# Full-Length Transcriptome Analysis Provides New Insights Into the Diversity of Immune-Related Genes in *Portunus trituberculatus*

**DOI:** 10.3389/fimmu.2022.843347

**Published:** 2022-04-07

**Authors:** Yi Zhang, Mengqi Ni, Yunhui Bai, Qiao Shi, Jinbin Zheng, Zhaoxia Cui

**Affiliations:** ^1^ School of Marine Sciences, Ningbo University, Ningbo, China; ^2^ Laboratory for Marine Biology and Biotechnology, Pilot Qingdao National Laboratory for Marine Science and Technology (Qingdao), Qingdao, China

**Keywords:** Portunus trituberculatus, full-length transcriptome, innate immune, transcript variants, c-type lectin

## Abstract

Generally, invertebrates were thought to solely rely on their non-specific innate immune system to fight against invading microorganisms. However, increasing studies have implied that the innate immune response of invertebrates displayed diversity and specificity owing to the hyper-variable immune molecules in organisms. In order to get an insight into the diversity of immune-related genes in *Portunus trituberculatus*, a full-length transcriptome analysis of several immune-related tissues (hemocytes, hepatopancreas and gills) in *P. trituberculatus* was performed and the diversity of several immune-related genes was analyzed. The full-length transcriptome analysis of *P. trituberculatus* was conducted using a combination of SMRT long-read sequencing and Illumina short-read sequencing. A total of 17,433 nonredundant full-length transcripts with average length of 2,271 bp and N50 length of 2,841 bp were obtained, among which 13,978 (80.18%) transcripts were annotated. Moreover, numerous transcript variants of various immune-related genes were identified, including pattern recognition receptors, antimicrobial peptides, heat shock proteins (HSPs), antioxidant enzymes and vital molecules in prophenoloxidase (proPO)-activating system. Based on the full-length transcriptome analysis, open reading frames (ORFs) of four C-type lectins (CTLs) were cloned, and tissue distributions showed that the four CTLs were ubiquitously expressed in all the tested tissues, and mainly expressed in hepatopancreas and gills. The transcription of the four CTLs significantly increased in several immune-related tissues (hemocytes, hepatopancreas and gills) of *P. trituberculatus* challenged with *Vibrio alginolyticus* and displayed different profiles. Moreover, the four CTLs displayed distinct bacterial binding and antibacterial activities. The recombinant protein PtCTL-1 (rPtCTL-1) and rPtCTL-3 displayed bacterial binding and antibacterial activities against all tested bacteria. rPtCTL-2 only showed bacterial binding and antibacterial activities against *V. alginolyticus*. No obvious bacterial binding or antibacterial activities for PtCTL-4 was observed against the tested bacteria. This study enriches the transcriptomic information on *P*. *trituberculatus* and provides new insights into the innate immune system of crustaceans. Additionally, our study provided candidates of antibiotic agents for the prevention and treatment of bacteriosis.

## Introduction

The swimming crab, *Portunus trituberculatus*, which is extensively distributed in East and Southeast Asia, is one of the most important commercial crabs in China (1). In the recent decade, the farming industry of *P*. *trituberculatus* has developed rapidly in China, and the annual production of *P*. *trituberculatus* in China is remain at approximately 100,000 tons (1). However, frequent outbreaks of diseases have resulted in severe economic losses and impede the sustainable development of the *P*. *trituberculatus* aquaculture industry. Mass mortalities of crustaceans attributed to outbreaks of vibriosis have been recorded from many regions (2–4). The vibriosis caused by *Vibrio alginolyticus* and *V*. *parahaemolyticus* has been responsible for the costliest disease (4–6). In recent years, in addition to *Vibrio* species, pathogenic bacteria such as *Staphylococcus aureus* has also become a ubiquitous pathogen in crab culture system (7). Coinfection with various pathogen seem to become a problematic for the crab farming industry. Since the abuse of antibiotics in aquaculture have resulted in serious antibiotics contamination and antibiotic resistance (8, 9), it is urgent to get a better understanding of the host immune system, which would be benefit for developing effective methods to control diseases.

Identification and functional characterization of immune molecules is basic for investigating immune characteristics of organisms. Moreover, several immune effector molecules are promising candidates of therapeutic agents (10–15). Recently, with the development of third-generation sequencing (TGS) technique, full-length transcriptome analysis has become an attractive high-throughput approach to excavate transcripts at isoform-level (16, 17). To date, an increasing number of full-length transcriptome analysis have been conducted in a variety of commercially important aquatic organisms using the TGS technique (18–22), which greatly facilitate the transcriptome research of aquatic animals lacking high-quality reference genome and provide technology for the development and utilization of antibacterial substances. However, the investigation of the full-length transcriptome has not yet been performed in *P. trituberculatus*.

In this context, with the purpose to get new insights into the innate immune system of *P. trituberculatus* and excavate candidate agents to control and cure the bacteriosis, the full-length transcriptome analysis of several immune-related tissues (hemocytes, hepatopancreas and gills) in *P. trituberculatus* was conducted. The tremendous transcript variants of immune-related molecules identified in this study provide new insight into the immune characteristics of *P. trituberculatus*. The data of full-length transcripts obtained in this study provided valuable genomic resources for further research into the new gene discovery and genomic research on *P. trituberculatus*. Moreover, this study provides candidates of antibiotic agents for the prevention and treatment of bacteriosis.

## Materials and Methods

### Animals and Samples

P. *trituberculatus* were purchased from a local seafood market in Ningbo (Zhejiang Province, China). The crabs were anesthetized by chilling on ice before dissection. For the full-length transcriptome analysis, three immune-related tissues (hemocytes, hepatopancreas and gills) were collected from *P. trituberculatus* with a body weight of 262.5 g. Crabs with a body weight of 87.72 ± 8.96 g were used for tissue distributions and immune response analyses. Crabs were acclimated in rectangular tanks (60 cm × 40 cm × 25 cm) with aerated seawater (25‰), each tank containing 8 individuals, and the seawater was renewed daily. Hemocytes, hepatopancreas, gills, stomach, intestine, heart, muscle and eyestalk were harvested from five crabs used for tissue distributions. For the immune challenge experiment, crabs were injected with 100 μL *V*. *alginolyticus* (2 × 10^8^ CFU/mL) resuspended in 0.1 M phosphate buffered saline (PBS) into the arthrodial membrane of the last walking leg. All of the bacterial strains used in this study were provided by Xiamen University (Xiamen, China). Crabs injected with 0.1 M PBS served as controls. At 3 h and 6 h post injection (hpi), hemocytes, hepatopancreas and gills were collected from five randomly selected crabs from each group. All samples were immediately frozen in liquid nitrogen and stored at −80°C until the extraction of total RNA for analysis.

### RNA Extraction and Qualification

Total RNA was extracted using RNAiso Plus (TaKaRa, Japan) according to the manufacturer’s instructions. RNA degradation was assessed on 1% agarose gels. The NanoPhotometer^®^ spectrophotometer (IMPLEN, CA, USA), Qubit^®^2.0 Fluorometer (Life Technologies, CA, USA) and Agilent Bioanalyzer 2100 system (Agilent Technologies, CA, USA) were used to detect the purity, concentration and integrity of the RNA samples.

### PacBio and Illumina Library Construction and Sequencing

Equal amounts of total RNA extracted from the three tissues were mixed to construct the library for PacBio and Illumina sequencing. The Isoform Sequencing (Iso-Seq) library was prepared according to the Iso-Seq protocol using the SMARTer PCR cDNA Synthesis Kit (Takara, Japan) and the BluePippin Size Selection System protocol as described by Pacific Biosciences (Sage science, USA). The qualified libraries were sequenced on a Pacbio Sequel platform.

Illumina sequencing library was constructed by pooling equal amounts of total RNA extracted from the three tissues. Sequencing libraries were generated using NEBNext^®^ Ultra™ RNA Library Prep Kit for Illumina^®^ (NEB, USA) following manufacturer’s recommendations and library quality was assessed on the Agilent Bioanalyzer 2100 system. The qualified libraries were sequenced on an Illumina Novaseq platform and 150 bp paired-end reads were generated.

### Data Processing

Raw polymerase reads were processed using the SMRTlink 5.0 software to obtain sub-reads. Circular consensus (CCS) reads were generated from the sub-reads *via* self-correction, and CCS reads contained the 5’ primer, 3’ primer and polyA tail were considered as full-length transcript sequence. And then, full-length non-chimeric (FLNC) reads were subjected to cluster and non-redundant treatment using hierarchical n*log(n) algorithm and Arrow polishing, and polished consensus reads were obtained. Finally, LoRDEC was used to correct these polished consensus reads with short reads yielded by Illumina sequencing, and CD-HIT program was further used to remove redundancy. Through above data processing procedure, non-redundant and accuracy full-length transcripts were produced for following analysis.

### Functional Annotation

For functional annotation, all transcripts were searched against the databases NCBI non-redundant nucleotide sequences (NT), NCBI non-redundant protein sequences (NR), Protein family (Pfam), Clusters of Orthologous Groups of proteins (COG), Swiss-Prot, Gene Ontology (GO), Kyoto Encyclopedia of Genes and Genomes (KEGG) and KEGG Ortholog database (KO). The BLAST programs with an E-value threshold of 10^−10^ were used in the NT database analysis. The Diamond BLASTX programs with an E-value threshold of 10^−10^ was used in the NR, KOG, Swiss-Prot and KEGG databases analysis. The Hmmscan programs was used in the Pfam database analysis.

### Prediction of Alternative Splicing (AS) Events

Alternative splicing analysis of non-redundant transcripts were processed with the Coding GENome reconstruction Tool (Cogent v3.1, https://github.com/Magdoll/Cogent).

### Sequence Analysis

Identities of deduced aa sequences were determined using BioEdit software (v7.1.3). Functional domains were predicted by SMART program (http://smart.embl-heidelberg.de/).

### Cloning of the Open Reading Frames (ORFs) Sequences of CTLs

Four CTLs, named PtCTL-1, PtCTL-2, PtCTL-3 and PtCTL-4, were selected for the immune function analysis. Extraction and qualification of total RNA was conducted as above mentioned. Reverse transcription to synthesize first-strand cDNA was carried out using PrimeScript™ RT reagent Kit with gDNA Eraser (TaKaRa, Japan) following the manufacturer’s protocols. The primers (**Table 1**) used for cloning were designed based on the sequence of the CTLs identified from the full-length transcriptome of *P*. *trituberculatus*. The amplification products were ligated into the pMD19-T vector (Takara, Japan) and sequenced (Sangon, China).

**Table 1:** Primers used in this study.

Primer name	Primer sequence (5’-3’)	Used for	Amplification efficiencies (%)
CTL-1-F	ATGAAGTGGGCCGCGCAT	ORF amplification	
CTL-1-R	TTAAGGACTTCTCTGACAGATAGCA		
CTL-2-F	ATGTTGGGGCTGGCCATG		
CTL-2-R	TCAATGCTGTCTATTTCGCATGA		
CTL-3-F	ATGAAGGTTTCTACAATTCCACTCC		
CTL-3-R	TCATTGTTCATTTGAGTCACTAACA		
CTL-4-F	ATGGGCGGGAGAGTGGC		
CTL-4-R	TTATAATGAGTCAGGGAAGAGTTGA		
CTL-1-F-*BamH*I	CGCGGATCCTGTCCCACTAACTTCATTCTCGTG	Vector construction	
CTL-1-R-*EcoR*I	CCGGAATTCTTAAGCACATATTTCAGCTTCTTTTTCG		
CTL-2-F-*EcoR*I	CCGGAATTCCCCGAGCCCTTCATGAAC		
CTL-2-R-*Hind*III	CCCAAGCTTTTATTCGCAGATGGAGCGTCG		
CTL-3-F-*EcoR*I	CCGGAATTCTGTCCTCCAGACTTTATCCATCTAG		
CTL-3-R-*Hind*III	CCCAAGCTTTTATTCACAGAGGTAGTGCATAGAGGTG		
CTL-4-F-*EcoR*I	CCGGAATTCATGGGCGGGAGAGTGGC		
CTL-4-R-*Hind*III	CCCAAGCTTTTATTATAATGAGTCAGGGAAGAGTTGA		
M13-47	CGCCAGGGTTTTCCCAGTCACGAC		
RV-M	GAGCGGATAACAATTTCACACAGG		
T7	TAATACGACTCACTATAGGG		
SP6	TGCTAGTTATTGCTCAGCGG		
q-CTL-1-F	TCTGGCTCGGAGGAAGTGA	Real-time PCR	98.870
q-CTL-1-R	TGATTGGGTTGATCGTTGC		
q-CTL-2-F	CATCCCAAAGAACCTGACTGA		99.615
q-CTL-2-R	GCGTCTCCAGATAGCCACTC		
q-CTL-3-F	GCCAGATAGAAGTCCAACACAA		104.847
q-CTL-3-R	CCCACCTTGTTTCTAACTCAGC		
q-CTL-4-F	CAGCGGTAGGTCACCTCACA		96.858
q-CTL-4-R	CAAGTCCAGTCCCATATCTTGG		
*Ptβ-actin* F	TCACACACTGTCCCCATCTACG		96.623
*Ptβ-actin* R	ACCACGCTCGGTCAGGATTTTC		

### Quantitative Real-Time RT-PCR

Quantitative real-time PCR (qRT-PCR) was carried out on an ABI 7500 real-time PCR detection system (Applied Biosystems, USA) using TB Green Premix Dimer Eraser kit (Takara, Japan) according to the manufacturer’s instructions. The specific primers used for qRT-PCR were designed based on the ORF sequence of the CTLs and listed in **Table 1**. The *β*-actin gene of *P. trituberculatus* were served as the internal standardization. The melting curve was recorded to confirm that only a single PCR product was amplified. Three technical replicates were conducted. The relative gene expression levels were determined by the 2^-△△Ct^ method (23).

### Expression and Purification of Recombinant Proteins

The cDNA fragments encoding the mature peptides of the four PtCTLs were amplified using primers with the restriction sites (**Table 1**). The target cDNA fragments were ligated into the expression vector pET-32a and confirmed by sequencing. The recombinant plasmids were transformed into *Escherichia coli* BL21 (DE3) pLysS Chemically Competent Cells (TransGen Biotech, China) and induced at 16°C for 12 h with isopropyl-β-thiogalactopyranoside (IPTG) at a final concentration of 0.5 mM. The induced bacterial pellets were collected after centrifugation (10,000 ×g) for 5 min at 4°C and then suspended in Tris-HCl buffer (50 mM Tris-HCl, 500 mM NaCl, 20 mM imidazole, pH 7.8) and sonicated on ice for 30 min with burst duration of 3 s. The supernatants were collected after centrifugation (10,000 ×g) for 25 min at 4°C. The purification of recombinant proteins was conducted using the high affinity Ni-NTA resin (GenScript, China) according to the manufacturer’s protocols. The purity of the recombinant proteins was analyzed by sodium dodecyl sulfate-polyacrylamide gel electrophoresis (SDS-PAGE) and Coomassie Blue staining, and the recombinant proteins were further confirmed by western blot analysis as previously described (24). The concentrations of recombinant proteins were examined using BCA Protein Assay Kit (Beyotimie, China).

### Antibacterial and Bacterial Binding Activities Assay

Gram-negative bacteria (*V*. *alginolyticus* and *V*. *parahaemolyticus*) and Gram-positive bacteria (*S*. *aureus*) were tested in the antibacterial and bacterial binding activities assay. *V. alginolyticus*, *V. parahaemolyticus* and *S. aureus* were suspended in TBS (50 mM Tris-HCl, 50 mM NaCl, 10 mM CaCl2, pH 7.5) at a concentration of 6.5 × 10^7^ CFU/mL, 6.8 × 10^7^ CFU/mL and 6.0 × 10^7^ CFU/mL, respectively. 50 μL of recombinant PtCTL proteins (rPtCTLs) (500 μg/mL) were mixed with the same volume of bacteria suspension and incubated at 37°C (*V. alginolyticus* and *S. aureus*) or 30°C (*V*. *parahaemolyticus*) for 2 h under slight rotation. The mixtures were diluted 10^4^-fold and spread on Luria-Bertani (*S. aureus*) or 2216E (*V. alginolyticus* and *V*. *parahaemolyticus*) agar plates, and the CFU were calculated after culturing for 8 h at 37°C/30°C. In the binding assay, the rPtCTLs and bacteria suspension were mixed and incubated as above mentioned. The bacterial pellets were harvested by centrifugation at 5,000 ×g for 3 min and washed five times with TBS. Bindings between rPtCTLs and bacteria was confirmed by western blot analysis.

### Statistical Analysis

All data were shown as the mean ± standard deviation (SD) and analyzed by the one-way analysis of variance (ANOVA) using the SPSS 17.0 software. 0.01 < *p* < 0.05 was considered statistically significant; *p* < 0.01 was considered to be extremely significant.

## Results

### Overview of the Sequencing Data

In total, 1,172,590 polymerase reads were obtained. The average length of polymerase reads was 75,948 bp, and the N50 length of polymerase reads was 141,113 bp (**Supplementary Table 1**). A total of 64,159,133 sub-reads with the average length of 1,314 bp and N50 length of 1,900 bp were generated from polymerase reads (**Supplementary Table 1**). Based on self-correction among sub-reads, 1,061,265 CCS reads were obtained, and the average length and N50 length of CCS reads were 2,006 bp and 2,471 bp, respectively (**Supplementary Table 1**). Subsequently, among the CCS reads, 668,918 full-length non-chimeric (FLNC) reads with average length of 1,760 bp and N50 length of 2,266 bp were identified (**Supplementary Table 1**). And then, 43,748 polished consensus reads with N50 length of 2,522 bp were produced from the FLNC reads (**Supplementary Table 1**). Finally, the polished consensus reads were subjected to correction using short reads produced by Illumina sequencing and removing redundancy. In total, 17,433 nonredundant full-length transcripts with average length of 2,271 bp and N50 length of 2,841 bp were obtained (**Supplementary Table 1**). Raw PacBio and Illumina sequencing reads are available at NCBI GenBank under accession numbers SRR12442560 and SRR12442561.

### Functional Annotation of Transcripts

In total, 13,978 (80.18%) transcripts were annotated in at least one database. A total of 12,972 (74.41%), 6,371 (36.55%), 10,172 (58.35%), 11,296 (64.80%), 10,172 (58.35%), 10,126 (58.09%), 12,343 (70.80%) transcripts were annotated in the NR, NT, Pfam, SwissProt, GO, KOG and KEGG database, respectively (**Supplementary Table 2**). For main species distribution matched against NR database, most of the matched transcripts were similar with *Hyalella azteca* (4,612), followed by *Zootermopsis nevadensis* (649), *Portunus trituberculatus* (506), *Scylla paramamosain* (473), *Limulus polyphemus* (344), *Daphnia magna* (332), *Eriocheir sinensis* (276) and *Litopenaeus vannamei* (209) (**Supplementary File 1**).

**Figure 1:**
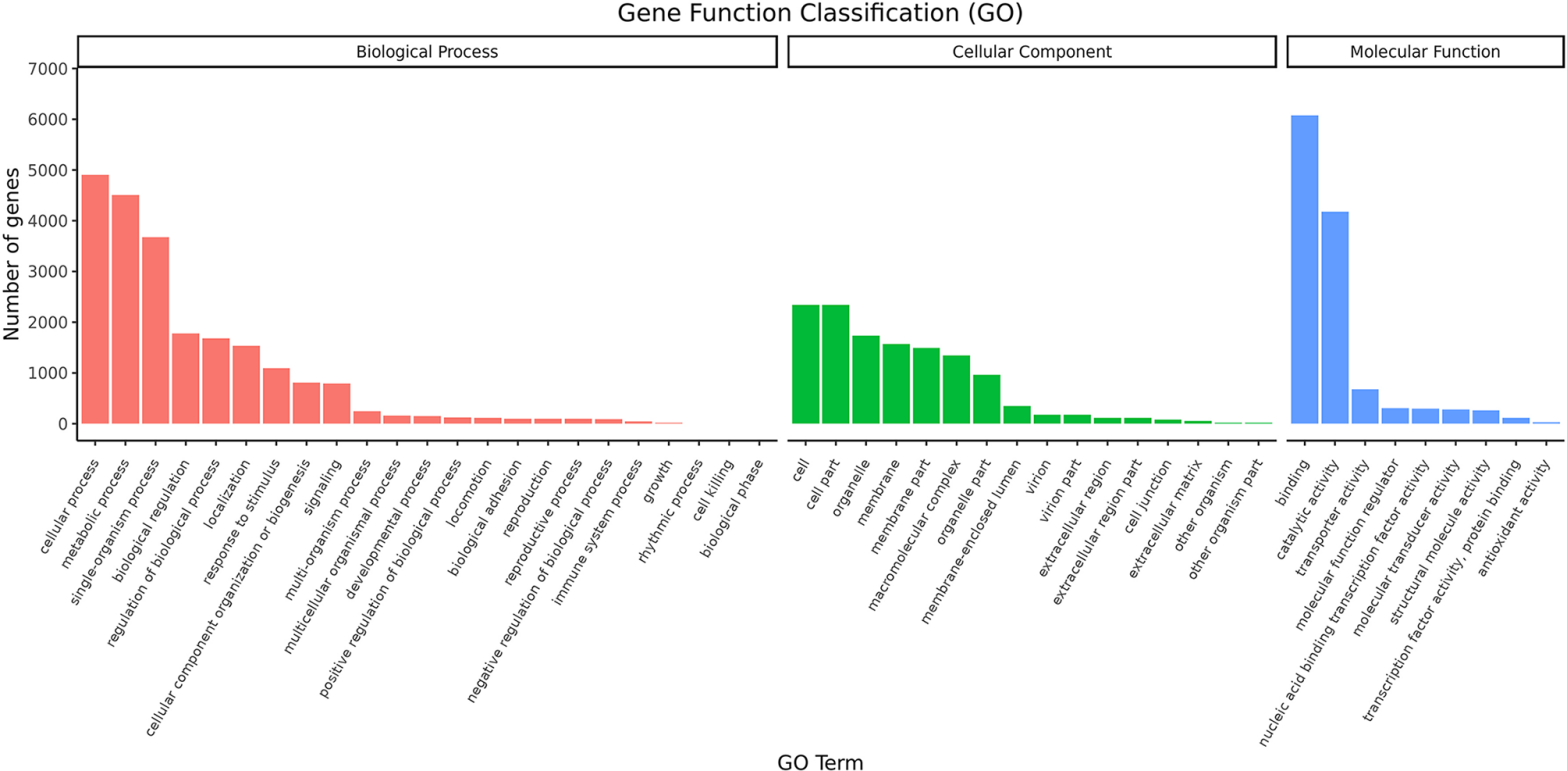
GO classification of the transcripts.

**Figure 2:**
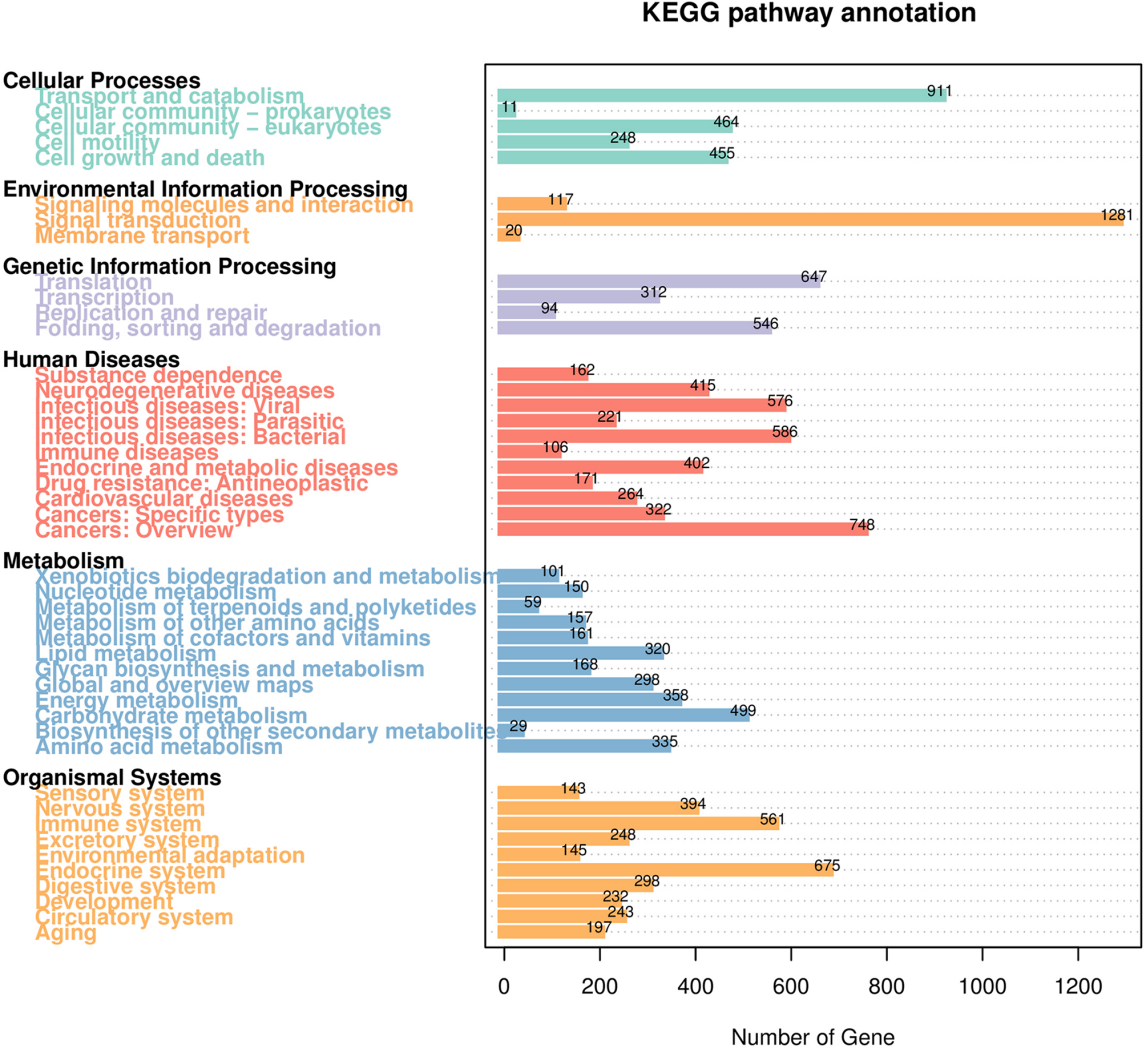
KEGG pathway classification of the transcripts. The number above the bars indicates the number of transcripts annotated in different KEGG pathways.

The distribution of GO second-level terms showed that cellular process (4,907), metabolic process (4,506) and single-organism process (3,678) were the most represented terms in the biological process category (**Figure 1** and **Supplementary File 2**). In the molecular function category, binding (6,075) and catalytic activity (4,173) comprised the large proportion (**Figure 1** and **Supplementary File 2**). As to cellular component category, most transcripts were associated with cell (2,344), cell parts (2,344) and organelle (1,739) (**Figure 1** and **Supplementary File 2**). The results of COG annotation showed that the cluster for general function prediction only (1,837), signal transduction mechanisms (1,368) and post-translation modification, protein turnover, chaperones (1,031) represented the top three class (**Supplementary File 3**). For the KEGG annotation, most transcripts were mapped to signal transduction (1,281) and transport and catabolism (911) related pathways (**Figure 2** and **Supplementary File 4**). Meanwhile, a total of 561 transcripts were mapped to immune system related pathways, such as chemokine signaling pathway, complement and coagulation cascades, Toll-like receptor signaling pathway, NOD-like receptor signaling pathway, RIG-I-like receptor signaling pathway and IMD signaling pathway (**Figure 2** and **Supplementary File 4**).

### Alternative Splicing Events Analysis

In total, 3,960 unique transcript models (UniTransModels) were reconstructed by 8,563 non-redundant full-length transcripts. 1,684 UniTransModels that have more than one isoform were used for alternative event analysis. As a result, a total of 315 AS events were predicted based on 244 UniTransModels and generate 483 full-length transcripts. The retained intron (RI) (167) is the most abundant type of AS events, followed by alternative 3’ splice sites (A3) (72), alternative 5’ splice sites (A5) (61), skipping exon (SE) (9), alternative first exons (AF) (4), alternative last exons (AL) (1) and mutually exclusive exons (MX) (1).

### Transcript Variants of Immune-Related Genes

**Figure 3:**
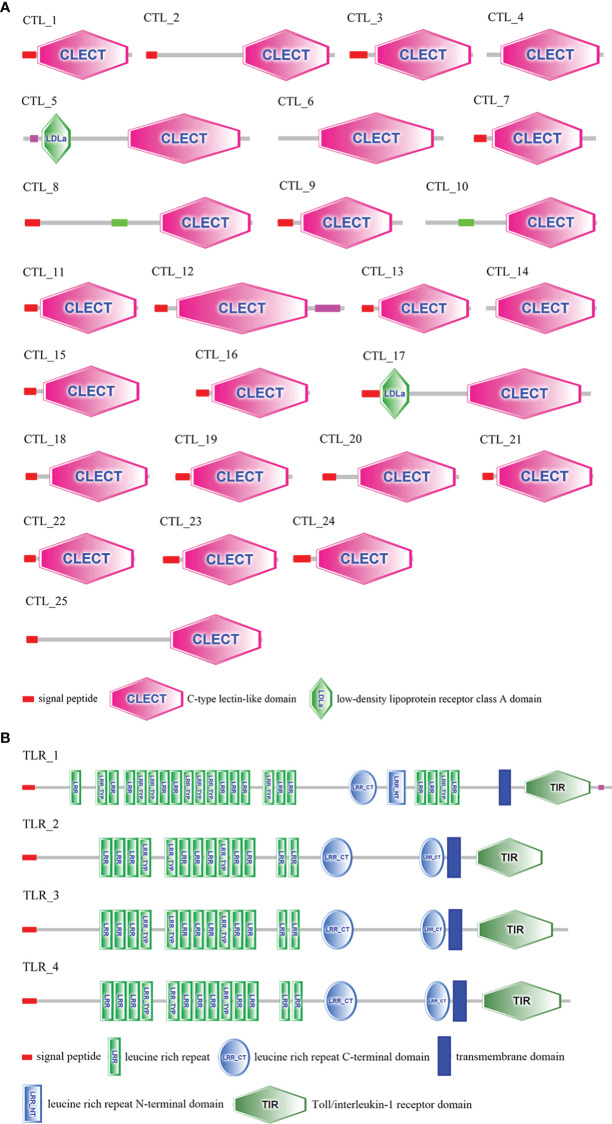
Domain architecture of CTLs **(A)** and TLRs **(B)** predicted by SMART. The sequences and transcript IDs are listed in **Supplementary File 5** and **Supplementary File 6**.

In this study, transcript variants of various immune-related genes such as pattern recognition receptors (PRRs), antimicrobial peptides (AMPs), vital molecules in prophenoloxidase (proPO)-activating system, antioxidant enzymes, and heat shock proteins (HSPs) were identified (**Table 2**). The ORFs and deduced amino acid (aa) sequences of these transcripts are listed in **Supplementary File 5**. A total of twenty-five C-type lectins (CTLs) were identified in this study. The functional domain analysis showed that the deduced aa sequences of the CTLs all contain a carbohydrate recognition domain (CRD), moreover, a signal peptide and a low-density lipoprotein receptor class A domain were also observed in some CTLs (**Figure 3A**). Homology comparisons showed that the deduced aa sequences of these CTLs shared 2.2% to 99.6% identity with each other (**Supplementary File 6**). In total, four Toll-like receptors (TLRs) were identified, and these TLRs showed 19.8% to 94.2% identity with each other (**Supplementary File 6**). These TLRs exhibited a typical domain architecture of their counterparts, including extracellular leucine-rich repeats (LRRs), a transmembrane domain and an intracellular Toll/interleukin-1 receptor (TIR) domain (**Figure 3B**). Herein, a total of twelve crustins, seven antilipopolysaccharide factors (ALFs) and fourteen hyastatin were identified. Homology comparisons showed that the deduced aa sequences of the crustins, ALFs and hyastatin shared 7.2% to 99%, 4% to 95.7% and 24% to 93.1% identity with each other, respectively (**Supplementary File 6**). In the current study, twenty-four proPO-activating factors (PPAFs) and twenty-one serine proteinases transcript variants were identified. Homology comparisons showed that the deduced aa sequences of the PPAFs and serine proteinases shared 10.1% to 98.6% and 7.1% to 96% identity with each other, respectively (**Supplementary File 6**). Moreover, several antioxidant enzymes, including twelve copper/zinc SODs (Cu/ZnSODs), two cytosolic manganese SODs (CytMnSODs), three CATs, three Gpxs and fifteen GSTs were identified. Additionally, we also identified several HSPs, including a HSP10, two HSP40, two HSP60, six HSP70 and two HSP90.

**Table 2:** Statistics of transcript variants of immune-related genes.

Immune-related genes	Transcript variants number
1. Antimicrobial peptides	
Antilipopolysaccharide factors	7
Hyastatin	14
Crustin	12
2. ProPO system	
Prophenoloxidase	1
Prophenoloxidase activating factor	24
Serine proteinase	21
Proteinase inhibitor	1
3. Antioxidant system	
Copper/zinc superoxide dismutase	12
Cytosolic manganese superoxide dismutase	2
Catalase	3
Glutathione peroxidase	3
Glutathione-S-transferase	15
Thioredoxin	3
4. PRR	
C-type lectin	25
M-type lectin	1
Galectin	2
Toll-like receptor	4
Scavenger receptor B	3
5. Heat shock proteins	
Heat shock protein 10	1
Heat shock protein 40	2
Heat shock protein 60	2
Heat shock protein 70	6
Heat shock protein 90	2
6. Other immune molecules	
Alpha-2-macroglobulin	5
Thioester-containing protein	1
Spatzle	8

### Cloning of PtCTLs cDNA

**Figure 4:**
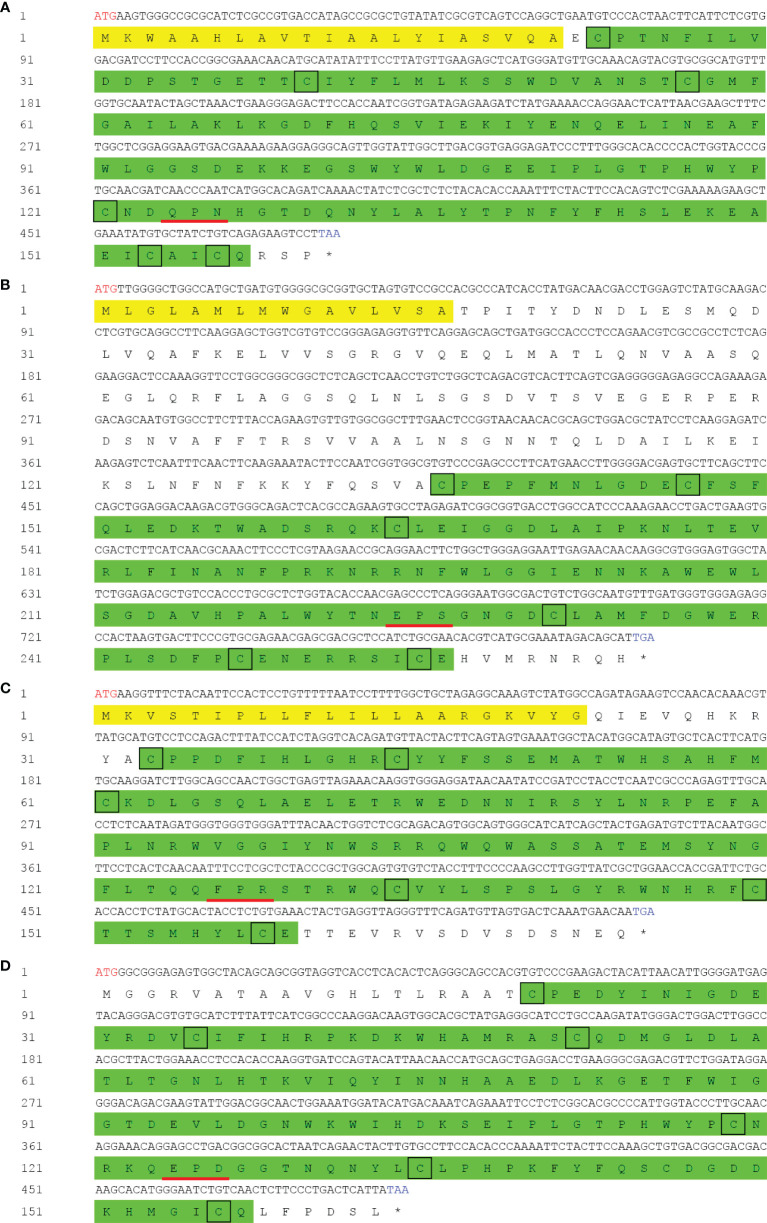
cDNA and deduced amino acid sequence of *PtCTLs*. Red letters indicate the start sites and blue letters represent the stop codons. The asterisk indicates the stop codon. The signal peptide is shaded in yellow and the CRD is highlighted in green. Highly conserved cysteine residues are shown by black boxes. The carbohydrate binding motif is underlined in red. **(A)** PtCTL-1; **(B)** PtCTL-2; **(C)** PtCTL-3; **(D)** PtCTL-4.

The cDNA sequences of PtCTL-1, PtCTL-2, PtCTL-3, and PtCTL-4 were deposited in GenBank with the accession number OL848444, OL848445, OL848446 and OL848447, respectively. The four PtCTLs encoded a polypeptide of 161 aa (18.08 kDa), 265 aa (29.68 kDa), 175 aa (20.62 kDa) and 164 aa (18.45 kDa), respectively. All of the four PtCTLs contained a single CRD with a characteristic carbohydrate binding motif (a QPN motif in PtCTL1, an EPS motif in PtCTL2, a FPR motif in PtCTL3 and an EPD motif in PtCTL4) and six cysteine residues (Cys^23^, Cys^40^, Cys^57^, Cys^121^, Cys^153^ and Cys^156^ in PtCTL1; Cys^136^, Cys^147^, Cys^164^, Cys^231^, Cys^247^ and Cys^255^ in PtCTL2; Cys^33^, Cys^44^, Cys^61^, Cys^134^, Cys^150^ and Cys^158^ in PtCTL3; Cys^20^, Cys^35^, Cys^52^, Cys^119^, Cys^135^ and Cys^156^ in PtCTL4) (**Figure 4**). Additionally, the PtCTL1, PtCTL2 and PtCTL3 also contained a signal peptide (Met^1^-Ala^21^ in PtCTL1, Met^1^-Ala^16^ in PtCTL2 and Met^1^-Gly^22^ in PtCTL3) in the N-terminus (**Figures 4A–C**).

### Tissue Distribution and Expression Profiles of the Four CTLs in *P. trituberculatus* Challenged With *V. alginolyticus*

**Figure 5:**
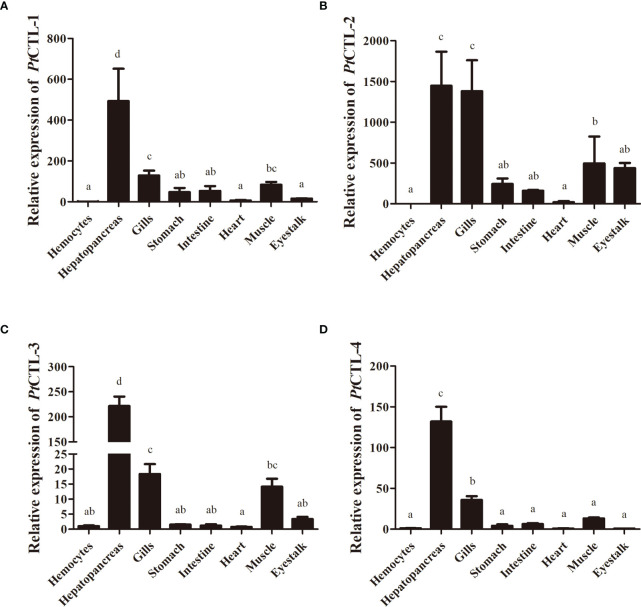
Spatial expression analysis of PtCTLs in different tissues. **(A)** PtCTL-1; **(B)** PtCTL-2; **(C)** PtCTL-3; **(D)** PtCTL-4. Expression levels in hepatopancreas, gills, stomach, intestine, heart, muscle and eyestalk are normalized to those in the hemocytes, and *β-actin* is used as a reference gene. Vertical bars represent the means ± SD, n = 5. Results were analyzed by one-way ANOVA and different letters (a-d) above the bars presented significant differences between groups (p < 0.05).

**Figure 6:**
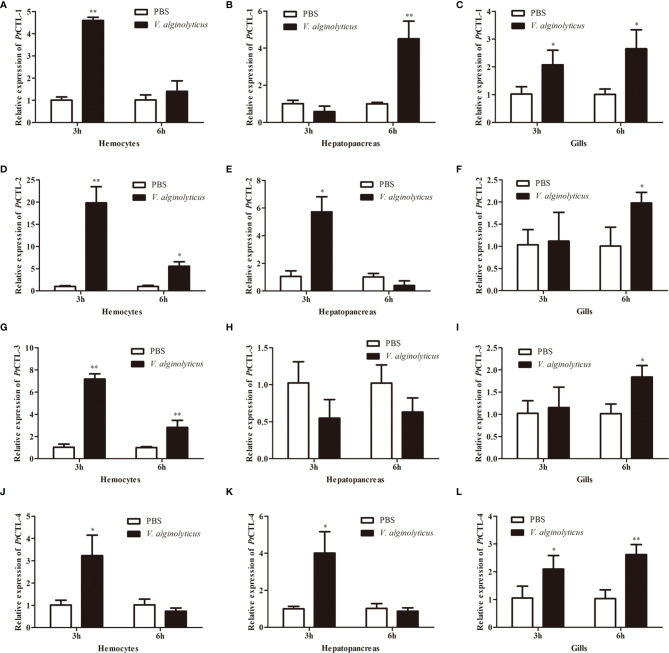
Expression profiles of *PtCTLs* in *V. alginolyticu*s-challenged *P. trituberculatus*. The relative expression of PtCTL-1 in hemocytes **(A)**, hepatopancreas **(B)** and gills **(C)** after *V. alginolyticus* injection; The relative expression of PtCTL-2 in hemocytes **(D)**, hepatopancreas **(E)** and gills **(F)** after *V. alginolyticus* injection; The relative expression of PtCTL-3 in hemocytes **(G)**, hepatopancreas **(H)** and gills **(I)** after *V. alginolyticus* injection; The relative expression of PtCTL-4 in hemocytes **(J)**, hepatopancreas **(K)** and gills **(L)** after *V. alginolyticus* injection. Each bar represents the mean ± SD (n = 5). Asterisks indicate statistically significant differences: *p < 0.05, **p < 0.01.

Tissue distribution analysis showed that the four *PtCTLs* were expressed in all tested tissues and showed relatively higher expression levels in the hepatopancreas and gills (**Figure 5**). Significant induction in the transcription of *PtCTL-1*, *PtCTL-2*, and *PtCTL-4* were observed in the hemocytes, hepatopancreas and gills of *P. trituberculatus* challenged with *V. alginolyticus*, and significant expression of *PtCTL-3* was detected in hemocytes and gills when *V. alginolyticus* challenged the *P. trituberculatus* (**Figure 6**). The transcriptional level of *PtCTL-1* in the hemocytes was up-regulated 4.59-fold (*P*<0.01) at 3 hpi (**Figure 6A**), and it was up-regulated 4.50-fold (*P*<0.01) at 6 hpi (**Figure 6B**) in the hepatopancreas. The transcription of *PtCTL-1* in the gills was up-regulated 2.07-fold and 2.65-fold (*P*<0.05) at 3 and 6 hpi (**Figure 6C**), respectively. The transcripts of *PtCTL-2* in hemocytes increased 19.81-fold and 5.57-fold (*P*<0.05) at 3 hpi and 6 hpi (**Figure 6D**), respectively. The transcripts of *PtCTL-2* in hepatopancreas increased 5.73-fold (*P*<0.05) at 3 hpi (**Figure 6E**), and it was up-regulated 1.98-fold (*P*<0.05) at 6 hpi in gills (**Figure 6F**). The mRNA expression of *PtCTL-3* in hemocytes was significantly up-regulated 7.18-fold and 2.83-fold (*P*<0.01) at 3 hpi and 6 hpi, respectively (**Figure 6G**). The expression level of *PtCTL-3* achieved a 1.84-fold (*P*<0.05) increment in gills (**Figure 6I**), while it was not changed in hepatopancreas (**Figure 6H**). The transcription of *PtCTL-4* in hemocytes (**Figure 6J**) and hepatopancreas (**Figure 6K**) increased 3.23-fold and 4.01-fold, respectively. The transcriptional level of *PtCTL-4* in gills was up-regulated 2.10-fold (*P*<0.05) and 2.62-fold (*P*<0.01) at 3 hpi and 6 hpi, respectively (**Figure 6L**).

### Bacterial Binding and Antibacterial Activities of rPtCTLs

**Figure 7:**
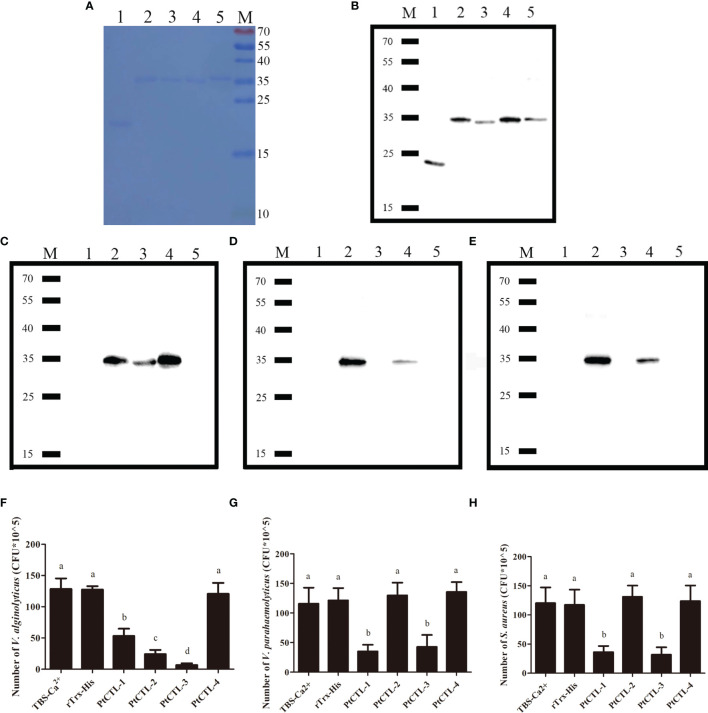
The bacteria binding activity and the antibacterial activities of recombinant CTLs. M, protein molecular standard (kDa); lane 1, rTrx-His; lane 2, PtCTL-1; lane 3, PtCTL-2; lane 4, PtCTL-3; lane 5, PtCTL-4. **(A)** SDS-PAGE analyses of purified recombinant proteins. **(B)** Western blot analyses of purified recombinant proteins. **(C–E)** Bacterial binding activity of recombinant PtCTLs proteins against *V*. *alginolyticus* **(C)**, *V*. *parahaemolyticus* **(D)** and *S*. *aureus* **(E)**. **(F–H)** Antibacterial activity of recombinant PtCTLs proteins against *V*. *alginolyticus* **(F)**, *V*. *parahaemolyticus* **(G)** and *S*. *aureus* **(H)**. Results were analyzed by one-way ANOVA and different letters (a-d) above the bars presented significant differences between groups at p < 0.05 (n = 3).

Recombinant proteins of the four PtCTLs were successfully produced and purified. Coomassie Blue staining (**Figure 7A**) and western blot (**Figure 7B**) showed that the recombinant proteins exhibited the expected sizes of approximately 23 kDa (His-Trx), 35 kDa (rPtCTL-1), 34 kDa (rPtCTL-2), 35 kDa (rPtCTL-3) and 36 kDa (rPtCTL-4). Bacterial binding activity assays showed that rPtCTL-1 and rPtCTL-3 could bind to *V. alginolyticus*, *V. parahaemolyticus* and *S. aureus* (**Figures 7C–E**). rPtCTL-2 only exhibited binding activity to *V. alginolyticus* (**Figures 7C–E**), while rPtCTL-4 could not bind to any tested bacteria (**Figures 7C–E**). The antibacterial activity assay showed that the four rPtCTLs displayed various antibacterial spectra against the tested bacteria. The rPtCTL-1 and rPtCTL-3 exhibited antibacterial activity against *V. alginolyticus*, *V. parahaemolyticus* and *S. aureus* (**Figures 7F–H**). The CFU of *V. alginolyticus* treated with rPtCTL-1, rPtCTL-2 and rPtCTL-3 significantly decreased 2.41-fold, 5.35-fold and 18.88-fold, respectively. Compared with the blank control group, the CFU of *V. parahaemolyticus* treated with rPtCTL-1 and rPtCTL-3 were significantly decreased by 3.29-fold and 2.72-fold, respectively. The CFU of *S. aureus* treated with rPtCTL-1 and rPtCTL-3 significantly decreased 3.32-fold and 3.79-fold, respectively, compared with the blank control group. No obvious antimicrobial activity of rPtCTL-4 was observed (**Figures 7F–H**).

## Discussion

Full-length cDNA sequence is basic for functional studies of genes. In the present study, to get an insight into the diversity of immune-related molecules in the *P. trituberculatus*, PacBio SMRT sequencing technology was used for identifying full-length transcripts in immune-related tissues of *P*. *trituberculatus*. In this study, a total of 17,433 nonredundant full-length transcripts with an average length of 2,271 bp and N50 length of 2,841 bp were obtained, which was longer than previously reported transcriptomes of *P*. *trituberculatus* obtained by NGS technologies (25–30). To obtain comprehensive information on gene function, transcripts were subjected to annotation analysis by searching against databases including NR, NT, Pfam, SwissProt, KOG, GO and KEGG. The results showed that the annotated rate (80.18%) of the full-length transcripts was much higher than that of transcripts obtained by next-generation sequencing (NGS) (25, 26, 29, 30).

Traditionally, be absent of adaptive immune system, invertebrates were thought to solely rely on their non-specific innate immune system to combat against invading microorganisms (31, 32). Recently, increasing studies revealed that the innate response of invertebrates displayed diversity and specificity (33–35). Unlike the antigen specificity and immunological memory of adaptive immunity, the hyper-variable immune molecules might be an underlying molecular mechanism of pathogen-specific innate immune responses in invertebrates (32, 36, 37). In this study, transcript variants of various immune-related genes were identified.

Pattern recognition is the primary step to implement an efficient immune response, in which PRRs sense the invading microorganisms and activate immune responses (38–40). To date, numerous PRRs have been identified and functionally studied in invertebrates, and PRRs have been found to display diverse and specific immune functions (41, 42). Herein, transcript variants of several PRRs, including CTL, TLR, galectin and scavenger receptor B (SRB), were identified. CTLs are a group of highly diverse PRRs that recognize various pathogens (41, 43). A total of 25 CTLs were identified in this study, most (20) of them contain a signal peptide and a CRD domain, and two CTLs with a low-density lipoprotein receptor class A domain (LDLa) were identified. The TLR-1 showed differences in the numbers and arrangement of LRRs compared with other three TLRs. Since the LRRs involved in the binding of microorganisms (44, 45), these TLRs may play diverse roles in recognizing invading pathogens. These highly abundant PRRs might contribute to the immune specificity of *P*. *trituberculatus*.

AMPs are effectors of the innate immune system to fight against invading microorganisms. Several AMPs in crab, such as crustin, ALF and hyastatin, have been identified and characterized in crab, and different types and isoforms of AMPs exhibit diverse and specific antimicrobial activities (25, 46–60). The massive variants of AMPs might be crucial for *P*. *trituberculatus* to eliminate diverse invading microorganisms.

ProPO-activating system is a crucial constituent of the immune system in invertebrates (51). To date, several genes associated with proPO-activating system, such as prophenoloxidase, PPAF, serine proteinase and its inhibitor, have been identified and characterized in crustacean species (52–57). In this study, numerous transcript variants of PPAFs and serine proteinases were identified. Considering the fact that proPO activation is mediated by PPAF and serine proteinase, the abundant variants of PPAFs and serine proteinases in *P*. *trituberculatus* may provide a molecular basis for the specific activation of proPO.

Reactive oxygen species (ROS) are considered as an important part of the innate immunity that participate in eliminating invading microorganisms (58). ROS can kill invading microorganisms; however, the overproduction of ROS will cause serious damage to cellular macromolecules (59). To maintain a balance of ROS, organisms have developed an antioxidant system to eliminate excessive ROS, which consist of various antioxidant enzymes such as superoxide dismutase (SOD), catalase (CAT), glutathione peroxidase (Gpx) and glutathione-S-transferase (GST) (60). In addition to antioxidant system, HSPs also play important roles in the maintenance of immune homeostasis by acting as molecular chaperones to maintain protein homeostasis (61). In the present study, various transcripts variants of antioxidant enzymes and HSPs were identified, which might equip *P*. *trituberculatus* to efficiently cope with adverse effects resulted from immune reaction.

Since abuse of antibiotics pose serious threats to the ecological environment and human health (8, 9), mining bioactive molecules to develop novel antimicrobial agents has attracted increasing attention of researchers. CTLs, which show high diversity in invertebrates, are promising candidates for antimicrobial agents for their antibacterial and antiviral activities (14, 15, 24, 35, 62). In this study, based on the full-length transcriptome analysis, the bacterial binding and antibacterial activities of four PtCTLs were investigated. The four PtCTLs were distinguished from previously identified CTLs in *P*. *trituberculatus* and shared less than 20.20% identity to CTLs identified in previous studies (GenBank: AGH68927.1, AHK59786.1, QEM39053.1, ATE51171.1, ATE51203.1, ACC86854.1, QIE05465.1, QGV11036.1). The four PtCTLs exhibited typical structures of CTLs, such as the CRD, a mannose-binding motif or a galactose-binding motif, four cysteine residues that were crucial for the formation of two disulfide bonds to stabilize the CRD structure. The tissue distribution showed that the four PtCTLs mainly expressed in the hepatopancreas and gills, which is consistent with the fact that the hepatopancreas and gills of crustaceans, two important tissues involved in immune response of crustaceans, are two main tissues that synthesizes CTLs (43, 63–66).

As multifunctional immune molecules in crustaceans, CTLs have been proven to be involved in diverse immune responses (43). In this study, to get a preliminary knowledge on the specific immune response of the four PtCTLs, the expression profiles of these PtCTLs in the hemocytes, hepatopancreas and gills of *P*. *trituberculatus* post-injection with *V. alginolyticus* were investigated. The transcriptional levels of the four PtCTLs showed different expression profiles after challenged with *V. alginolyticus*. The tissue-specific transcriptional profiles of the four PtCTLs implying the complicated and diverse innate immune response of *P*. *trituberculatus*.

T.he primary function of CTLs is act as PRRs that recognize and bind to the microorganisms (11, 15, 67–69). To further ascertain the roles of these PtCTLs, recombinant proteins of the PtCTLs were produced, and the bindings between the rPtCTLs and bacteria were analyzed. Bacterial binding assay showed that the four rPtCTLs displayed different bacterial binding activity. The rPtCTL-1 and rPtCTL-3 possessed the broadest spectrum of bacterial binding activity against the tested bacteria, including *V. alginolyticus*, *V. parahaemolyticus* and *S. aureus*, which was similar to previous studies (70). In addition to act as PRRs, CTLs also exhibit antibacterial activities (14, 24, 62, 64). Corresponding to the bacterial binding activities, antibacterial assays showed that rPtCTL-1 and rPtCTL-3 possessed the widest spectrum of antibacterial activity against the tested bacteria, which was similar with a CTL isolated from *P*. *trituberculatus* in previous study (70). The rPtCTL-2 only displayed bacterial binding and antibacterial activities against *V. alginolyticus* against the tested bacteria, whereas no bacterial binding and antibacterial activities of rPtCTL-4 was observed against the tested bacteria, which was different to previous studies (70–72). Differences in bacterial binding and antibacterial activities of the four rPtCTLs might due to the mutation of the carbohydrate binding motif (24, 73). Additionally, previous reports showed that the CRD recognized subtle differences in the arrangement and branching of the carbohydrate residues, which may result in specific sets of carbohydrate recognition profiles for CRD (74). These findings suggested that these diverse PtCTLs may play specific roles in the defense against bacteria, and the rPtCTL-1 and rPtCTL-3 might be a promising antibiotic agent.

## Conclusion

In conclusion, a full-length transcriptome analysis of several immune-related tissues in *P. trituberculatus* was performed. In total, 17,433 nonredundant full-length transcripts were obtained. Massive transcript variants of various immune-related genes, including CTLs, TLRs, crustins, ALFs, hyastatins, SODs, CATs, Gpxs, GSTs, HSP40, HSP60, HSP70, HSP90, PPAFs and serine proteinase, were identified. Moreover, the bacterial binding and antibacterial activities of four PtCTLs were investigated. The four PtCTLs exhibited diverse transcriptional profiles when challenged with *V. alginolyticus*, and displayed different bacterial binding and antibacterial activities against bacteria. These results will facilitate the excavation of immune molecules in *P. trituberculatus* and provide clues for further investigating the diversity and specificity of innate immune response in crustaceans. Furthermore, the widest antibacterial spectrum exhibited by rPtCTL-1 and rPtCTL-3 implied their promising application prospects in prevention and treatment of bacteriosis.

## Data Availability Statement

The original contributions presented in the study are included in the article/**Supplementary Material**. Further inquiries can be directed to the corresponding author.

## Ethics Statement

Ethical review and approval was not required for the animal study because Portunus trituberculatus is not an endangered or protected species, and there is no requirement for ethical approval to perform experiments involving this species in China.

## Author Contributions

Conceived and designed the experiments, ZC and JZ. Performed the experiments, YZ, MN, YB, and QS. Analyzed the data and wrote the paper, JZ and YZ. All authors contributed to the article and approved the submitted version.

## Funding

This research was supported by the National Key R&D Program of China (No. 2018YFD0900303), the National Natural Science Foundation of China (No. 32072964), the Ten Thousand Talents Program and the K. C. Wong Magna Fund of Ningbo University.

## Conflict of Interest

The authors declare that the research was conducted in the absence of any commercial or financial relationships that could be construed as a potential conflict of interest.

## Publisher’s Note

All claims expressed in this article are solely those of the authors and do not necessarily represent those of their affiliated organizations, or those of the publisher, the editors and the reviewers. Any product that may be evaluated in this article, or claim that may be made by its manufacturer, is not guaranteed or endorsed by the publisher.
