# Supplementary material for: Full-Length Transcriptome Analysis Provides New Insights Into the Diversity of Immune-Related Genes in Portunus trituberculatus
Source: Front Immunol. 2022 Apr 7;13:843347. doi: 10.3389/fimmu.2022.843347 (PMC9021376; doi:10.3389/fimmu.2022.843347)
Supplement: Supplementary file 5 [file DataSheet_5.docx]

transcript_transcript/11522_Pt_Mix_transcript11522/f10p0/2555

ATGAAGTGGGCCGCGCATCTCGCCGTGACCATAGCCGCGCTGTATATCGCGTCAGTCCAGGCTGAATGTCCCACTAACTTCATTCTCGTGGACGATCCTTCCACCGGCGAAACAACATGCATATATTTCCTTATGTTGAAGAGCTCATGGGATGTTGCAAACAGTACGTGCGGCATGTTTGGTGCAATACTAGCTAAACTGAAGGGAGACTTCCACCAATCGGTGATAGAGAAGATCTATGAAAACCAGGAACTCATTAACGAAGCTTTCTGGCTCGGAGGAAGTGACGAAAAGAAGGAGGGCAGTTGGTATTGGCTTGACGGTGAGGAGATCCCTTTGGGCACACCCCACTGGTACCCGTGCAACGATCAACCCAATCATGGCACAGATCAAAACTATCTCGCTCTCTACACACCAAATTTCTACTTCCACAGTCTCGAAAAAGAAGCTGAAATATGTGCTATCTGTCAGAGAAGTCCTTAA

MKWAAHLAVTIAALYIASVQAECPTNFILVDDPSTGETTCIYFLMLKSSWDVANSTCGMFGAILAKLKGDFHQSVIEKIYENQELINEAFWLGGSDEKKEGSWYWLDGEEIPLGTPHWYPCNDQPNHGTDQNYLALYTPNFYFHSLEKEAEICAICQRSP

transcript_transcript/26430_Pt_Mix_transcript26430/f2p0/1493

ATGTTGGGGCTGGCCATGCTGATGTGGGGCGCGGTGCTAGTGTCCGCCACGCCCATCACCTATGACAACGACCTGGAGTCTATGCAAGACCTCGTGCAGGCCTTCAAGGAGCTGGTCGTGTCCGGGAGAGGTGTTCAGGAGCAGCTGATGGCCACCCTCCAGAACGTCGCCGCCTCTCAGGAAGGACTCCAAAGGTTCCTGGCGGGCGGCTCTCAGCTCAACCTGTCTGGCTCAGACGTCACTTCAGTCGAGGGGGAGAGGCCAGAAAGAGACAGCAATGTGGCCTTCTTTACCAGAAGTGTTGTGGCGGCTTTGAACTCCGGTAACAACACGCAGCTGGACGCTATCCTCAAGGAGATCAAGAGTCTCAATTTCAACTTCAAGAAATACTTCCAATCGGTGGCGTGTCCCGAGCCCTTCATGAACCTTGGGGACGAGTGCTTCAGCTTCCAGCTGGAGGACAAGACGTGGGCAGACTCACGCCAGAAGTGCCTAGAGATCGGCGGTGACCTGGCCATCCCAAAGAACCTGACTGAAGTGCGACTCTTCATCAACGCAAACTTCCCTCGTAAGAACCGCAGGAACTTCTGGCTGGGAGGAATTGAGAACAACAAGGCGTGGGAGTGGCTATCTGGAGACGCTGTCCACCCTGCGCTCTGGTACACCAACGAGCCCTCAGGGAATGGCGACTGTCTGGCAATGTTTGATGGGTGGGAGAGGCCACTAAGTGACTTCCCGTGCGAGAACGAGCGACGCTCCATCTGCGAACACGTCATGCGAAATAGACAGCATTGA

MLGLAMLMWGAVLVSATPITYDNDLESMQDLVQAFKELVVSGRGVQEQLMATLQNVAASQEGLQRFLAGGSQLNLSGSDVTSVEGERPERDSNVAFFTRSVVAALNSGNNTQLDAILKEIKSLNFNFKKYFQSVACPEPFMNLGDECFSFQLEDKTWADSRQKCLEIGGDLAIPKNLTEVRLFINANFPRKNRRNFWLGGIENNKAWEWLSGDAVHPALWYTNEPSGNGDCLAMFDGWERPLSDFPCENERRSICEHVMRNRQH

transcript_transcript/29780_Pt_Mix_transcript29780/f5p0/1232

ATGAAGGTTTCTACAATTCCACTCCTGTTTTTAATCCTTTTGGCTGCTAGAGGCAAAGTCTATGGCCAGATAGAAGTCCAACACAAACGTTATGCATGTCCTCCAGACTTTATCCATCTAGGTCACAGATGTTACTACTTCAGTAGTGAAATGGCTACATGGCATAGTGCTCACTTCATGTGCAAGGATCTTGGCAGCCAACTGGCTGAGTTAGAAACAAGGTGGGAGGATAACAATATCCGATCCTACCTCAATCGCCCAGAGTTTGCACCTCTCAATAGATGGGTGGGTGGGATTTACAACTGGTCTCGCAGACAGTGGCAGTGGGCATCATCAGCTACTGAGATGTCTTACAATGGCTTCCTCACTCAACAATTTCCTCGCTCTACCCGCTGGCAGTGTGTCTACCTTTCCCCAAGCCTTGGTTATCGCTGGAACCACCGATTCTGCACCACCTCTATGCACTACCTCTGTGAAACTACTGAGGTTAGGGTTTCAGATGTTAGTGACTCAAATGAACAATGA

MKVSTIPLLFLILLAARGKVYGQIEVQHKRYACPPDFIHLGHRCYYFSSEMATWHSAHFMCKDLGSQLAELETRWEDNNIRSYLNRPEFAPLNRWVGGIYNWSRRQWQWASSATEMSYNGFLTQQFPRSTRWQCVYLSPSLGYRWNHRFCTTSMHYLCETTEVRVSDVSDSNEQ

transcript_transcript/35456_Pt_Mix_transcript35456/f13p0/724

ATGGGCGGGAGAGTGGCTACAGCAGCGGTAGGTCACCTCACACTCAGGGCAGCCACGTGTCCCGAAGACTACATTAACATTGGGGATGAGTACAGGGACGTGTGCATCTTTATTCATCGGCCCAAGGACAAGTGGCACGCTATGAGGGCATCCTGCCAAGATATGGGACTGGACTTGGCCACGCTTACTGGAAACCTCCACACCAAGGTGATCCAGTACATTAACAACCATGCAGCTGAGGACCTGAAGGGCGAGACGTTCTGGATAGGAGGGACAGACGAAGTATTGGACGGCAACTGGAAATGGATACATGACAAATCAGAAATTCCTCTCGGCACGCCCCATTGGTACCCTTGCAACAGGAAACAGGAGCCTGACGGCGGCACTAATCAGAACTACTTGTGCCTTCCACACCCAAAATTCTACTTCCAAAGCTGTGACGGCGACGACAAGCACATGGGAATCTGTCAACTCTTCCCTGACTCATTATAA

MGGRVATAAVGHLTLRAATCPEDYINIGDEYRDVCIFIHRPKDKWHAMRASCQDMGLDLATLTGNLHTKVIQYINNHAAEDLKGETFWIGGTDEVLDGNWKWIHDKSEIPLGTPHWYPCNRKQEPDGGTNQNYLCLPHPKFYFQSCDGDDKHMGICQLFPDSL

transcript_transcript/21750_Pt_Mix_transcript21750/f2p0/1786

ATGAAGACTTCACTTGGCTGCTTACACGCGGCCCTCGCTCTTGTCCTCGTCCTCGCCCTGCTTCAGCCAGCAGAGGGAGACTGTGGACGAAGTGACCAAATCAAGTGTAGGACTGGGAATCAATGTATCTATAAAAGCTACATTTGCAACAAACAATCAGAATGTCCAGACGGTTCAGATGAGGACCCTAAAATGTGTAAGTTTTGGCCGACGAAGAGTCAAGAATGCCGCTATGAAAGTAGCAGTTATATCTACCATAACGGAAACTGTCGAACAGTGGATTATATGTGCCAGAACAGCGATCGAGCCACGAACATTTATCACCAAGTTTGCAAGGTTATTCTTCGGCCAAAACTGGAGTTCGAGGACGAAGGAATGAGCACGAGTGGTGAGCTGCTCGCTTTGCTGAACTCAGCTGTTAACCACACTCTGAACCAAAAGTTACCAGACTGTCCCATGCTGTACTCCCGCGTTGGAAATGAATGCCTTTCCTTTTTCTCACCAGCCAGGGTGTCGTGGGCCGAAGCTCGACAGTTTTGTCTTAGCATCTACGGTGACTTGTGGCAAGCAAAGGATCTGGCAGCGTATGGGCGCCTAATGGAGTACATGCGGGAAGAAAAATTGACCGCTAATTACTGGATTGGTGGACGGTACGACATCGATACCAACGCATGGTCGTGGACGACGGATGACTCCACTATGCCCCTGGGCGTTCCATTCTGGGCAATTAGGTACGTGGACTCCTGCGTGCAAAGGGGACCTCCACACACCGACCCTTATTCCGCACCGCCCGCAGCTCTACCCGGGGCCCGCTGTTATCGAGCCGTCTTATATCCACAACAAAGATCTCCGGGCTGGTGTTCTGCAATGACCTACGAGCACTTCTACTACTGGACTGACGAGATGTGTGATGAAGCCTACAGCCCACTGTGCACCTTCACTGGCCCCGTCGCCGCCCCCATTGTGGCTGACGCTCATTAG

MKTSLGCLHAALALVLVLALLQPAEGDCGRSDQIKCRTGNQCIYKSYICNKQSECPDGSDEDPKMCKFWPTKSQECRYESSSYIYHNGNCRTVDYMCQNSDRATNIYHQVCKVILRPKLEFEDEGMSTSGELLALLNSAVNHTLNQKLPDCPMLYSRVGNECLSFFSPARVSWAEARQFCLSIYGDLWQAKDLAAYGRLMEYMREEKLTANYWIGGRYDIDTNAWSWTTDDSTMPLGVPFWAIRYVDSCVQRGPPHTDPYSAPPAALPGARCYRAVLYPQQRSPGWCSAMTYEHFYYWTDEMCDEAYSPLCTFTGPVAAPIVADAH

transcript_transcript/23436_Pt_Mix_transcript23436/f2p0/1667

ATGTGTCTGAACGCCAGAGAAACCAGTGAAATAAATTCGAGAATTTGTGAGATTATTCTTCAACCAAAACTGGACGTCAGGAAGCTAGATGGTATGAACATGAGCAGTGAGGTGATCTCCTTGCTGAATTCAGCTGTTAACTCCACCTTGAATTATAAGACGCCAGACTGCCCCATGCTGTACCTCCGCGTCGGAAATGATTGTCTCTCTTTCTTCTCACCAGCCAAGGTGTCGTGGGCAGAGGCTCGACAGTTCTGTCTTAGCATCTATGGTGACCTGTGGCATGCAAAGGATTTTGAAAGTTATGGCCGCCTAATGGAGTATATGAGAGAGGAAAAACTGACCTCTAATTACTGGATTGGCGGACGCTACGATATTGATACCAATGCATGGTCGTGGACGGCAGACGACTCTGCCATGCCCCTTGGCGCTCCATTCTGGTCGATGAAGTACGAGGACTCCTGCGTGCAAAGGGGACCTCCACACACCGACCCATATTCCGCACCGCCCGCAGCTCTACCCGGGGCCCGCTGTTATCGAGCCGTCTTATCTCCACAAAAAAGATCTCCGGGCTGGTGTTCTGCAATGACCTACGAGCACTTCTACTACTGGACTGACGAGATGTGTGATGAAGCCTTCAGCCCACTGTGCACCTTCACTGGCCCCGTCGCCGCCCCCGTTGTGGCTGACGCTCATTAG

MCLNARETSEINSRICEIILQPKLDVRKLDGMNMSSEVISLLNSAVNSTLNYKTPDCPMLYLRVGNDCLSFFSPAKVSWAEARQFCLSIYGDLWHAKDFESYGRLMEYMREEKLTSNYWIGGRYDIDTNAWSWTADDSAMPLGAPFWSMKYEDSCVQRGPPHTDPYSAPPAALPGARCYRAVLSPQKRSPGWCSAMTYEHFYYWTDEMCDEAFSPLCTFTGPVAAPVVADAH

transcript_transcript/26283_Pt_Mix_transcript26283/f11p0/1496

ATGCGGTGGCTGGTTCCAGGAGCGGTCTTGGTGCTTGTCTCACTCTCCGCAGGACAGCGAAGTGTAGAGACTGTTGACTTGGCAGGCTCTCGCTATTTCATCTCCCAGACCTCGCCTTACGTGCCCAGCCTCAACTGGTTCCTCGCCTACCAGTACTGTCGCACCATTGGCATGGAGCTTCTTAGCATAGGCACTGCTGAGGAGGCTGAGCTCATAAACTCCTATCTTGCTGCCAACCGTTTGAGTGACCGGGACTACTGGACGAGTGGTAACCAGCTGGGCTCTCACCTCTGGATGTGGATGGCCACTGGGCAGCGCTTCAACACCACCTTCAACTTCTGGGTGCATGACGAGTCTTTCACCAAGTCAACAGCTGCGTGTATGAGTGTAAATAATGGGGCTTGGGTGCCAGAGGACTGTATGCAGGAGAAATTCTTTATCTGTGAACTGACACGGTGCTTCTTCGTCAATTTTGTGAGCGCCAACCGGGGCTCATCTCAGGGGTAA

MRWLVPGAVLVLVSLSAGQRSVETVDLAGSRYFISQTSPYVPSLNWFLAYQYCRTIGMELLSIGTAEEAELINSYLAANRLSDRDYWTSGNQLGSHLWMWMATGQRFNTTFNFWVHDESFTKSTAACMSVNNGAWVPEDCMQEKFFICELTRCFFVNFVSANRGSSQG

transcript_transcript/14121_Pt_Mix_transcript14121/f2p0/2310

ATGCGCCCCACCGAGATGAGATGGTATGCGGCCGTCGTGCTGGTCCTCGCGGGTTTGGCAGCGGCGCAGGAGAATCTAGCAGGCCCCGTGGAGAGCCATAACGTGGGCCGCAAGAATGGAGCCACCAGTCCCGTGACTCCTTACGGCAAACGGTACGGCGGCTCAGCGAGGAATGACGTGACGGTGAGGCGGTACGGCGCGTCGAATCCCAACGGCAACCACAATATGCACGACGGCCACCGCATGATCACCCGCGCTCTCTGGAGGATCTTCGACGCCCTCATGCAGAAGAAGGACCACTCGCAGTTGTACGGCCGCATGGGCTTCGTGGAGGAGGCGCTCAAAAAGATGATCTCCGTGGATTCCCAGCTGGAGGGAGAGATCGAGAAGCTGAAGGAGAACATGGGCGTGTGCATGTCCCAGCTGTCCACGGTGATGGAGGAGCTGGGTCGCCTGAAGACCTTTAGCACGCGGCTGGAGATGCTGGAGCAGACTCGCCCTGAGATGCAGAACCAGGCCGACCAGGAACACGATGCTGTTTGGGGATCAGCCGCCGCATACTCCACCTGCCCGTCTCCGTTCACCCGCATCGCCAACGAATGCTTCTACGTGTCCACCGACGAGATGCTGGGCTGGGAGGACGCTAGGAGGGAGTGTGGTAGGCTGGGTGGGGACCTCGCCTCTCCACGTAACCTTACCGTGCTGAGAGAATTCCTCGGCAGTGTCCAGGATCCCCCCGAGTACGTGTGGGTGGGAGGCACCAAGCAGGACGAGGGAACGTGGGTGTGGACTTCAGGCCCTCAGGCTGGCGTGCCCATCGACATGAGCAAGAGCACCTGGAACGAGGAAGTTCCCTCCGGCTCCGGCAAGTGCATGGGACTGTTTGGCCAGAGCTCCTACCGCGCCTACAACTACGACTGCAAGGAGAGGGACTTCTTCGTGTGCCAGTACTTGTTCTAA

MRPTEMRWYAAVVLVLAGLAAAQENLAGPVESHNVGRKNGATSPVTPYGKRYGGSARNDVTVRRYGASNPNGNHNMHDGHRMITRALWRIFDALMQKKDHSQLYGRMGFVEEALKKMISVDSQLEGEIEKLKENMGVCMSQLSTVMEELGRLKTFSTRLEMLEQTRPEMQNQADQEHDAVWGSAAAYSTCPSPFTRIANECFYVSTDEMLGWEDARRECGRLGGDLASPRNLTVLREFLGSVQDPPEYVWVGGTKQDEGTWVWTSGPQAGVPIDMSKSTWNEEVPSGSGKCMGLFGQSSYRAYNYDCKERDFFVCQYLF

transcript_transcript/29393_Pt_Mix_transcript29393/f5p0/1274

ATGTCACCATGTGAGAGAGGCGCCGCGGCCCACGGCGTGTTCCTGATGTTGATGGGAAGCGTGGTCCTGGTAGCGGCGACGAGTGGAGCCTCTTCAATTGAGGTTGAGTGCCACAGCCCCTTCACAGAGGTGGGGGGCCGCTGCGTGCACCTCGACCACAGCGTATCCGGCACCTGGTTGGAAATGAGGCGGTTCTGCCAGATGCTTGGGGGAGACTTGATCAACCTCGAAGATGTACAGTTTTACGAAGACATTCTTACATACATACGAGTCTTGGATCTGCCCAAAGTCCACTTTTGGATCGGCGCGTCTGATCTGGAGCAGGAAGGCCACTGGATGTGGACAGACAGGACGCCTGTCAAGATGGGCACGCCCTTCTGGGCCAATTATGGAGCCGACAATATACAGATGCCTGCAGGAGGAAAAGGACAAAACTGTGCTCTACTCGATGTGAATTTGCACTACTACTTCAATGATTACGAATGCGAGAGTTCCACAAAATGTGCAATCTGTGAAAAGATATACTGA

MSPCERGAAAHGVFLMLMGSVVLVAATSGASSIEVECHSPFTEVGGRCVHLDHSVSGTWLEMRRFCQMLGGDLINLEDVQFYEDILTYIRVLDLPKVHFWIGASDLEQEGHWMWTDRTPVKMGTPFWANYGADNIQMPAGGKGQNCALLDVNLHYYFNDYECESSTKCAICEKIY

transcript_transcript/18432_Pt_Mix_transcript18432/f2p0/2007

ATGCACGACGGCCACCGCATGATCACCCGCGCTCTCTGGAGGATCTTCGACGCCCTCATGCAGAAGAAGGACCACTCGCAGTTGTACGGCCGCATGGGCTTCGTGGAGGAGGCGCTCAAAAAGATGATCTCCGTGGATTCCCAGCTGGAGGGAGAGATCGAGAAGCTGAAGGAGAACATGGGCGTGTGCATGTCCCAGCTGTCCACGGTGATGGAGGAGCTGGGTCGCCTGAAGACCTTTAGCACGCGGCTGGAGATGCTGGAGCAGACTCGCCCTGAGATGCAGAACCAGGCCGACCAGGAACACGATGCTGTTTGGGGATCAGCCGCCGCATACTCCACCTGCCCGTCTCCGTTCACCCGCATCGCCAACGAATGCTTCTACGTGTCCACCGACGAGATGCTGGGCTGGGAGGACGCTAGGAGGGAGTGTGGTAGGCTGGGTGGGGACCTCGCCTCTCCACGTAACCTTACCGTGCTGAGAGAATTCCTCGGCAGTGTCCAGGATCCCCCCGAGTACGTGTGGGTGGGAGGCACCAAGCAGGACGAGGGAACGTGGGTGTGGACTTCAGGCCCTCAGGCTGGCGTGCCCATCGACATGAGCAAGAGCACCTGGAACGAGGAAGTTCCCTCCGGCTCCGGCAAGTGCATGGGACTGTTTGGCCAGAGCTCCTACCGCGCCTACAACTACGACTGCAAGGAGAGGGACTTCTTCGTGTGCCAGTACTTGTTCTAA

MHDGHRMITRALWRIFDALMQKKDHSQLYGRMGFVEEALKKMISVDSQLEGEIEKLKENMGVCMSQLSTVMEELGRLKTFSTRLEMLEQTRPEMQNQADQEHDAVWGSAAAYSTCPSPFTRIANECFYVSTDEMLGWEDARRECGRLGGDLASPRNLTVLREFLGSVQDPPEYVWVGGTKQDEGTWVWTSGPQAGVPIDMSKSTWNEEVPSGSGKCMGLFGQSSYRAYNYDCKERDFFVCQYLF

transcript_transcript/29808_Pt_Mix_transcript29808/f46p0/1184

ATGAAGTGGATAGCGACACTTGCTGTGGCCGCAGCCGCTGTTGTCCTTTCCTCGGCACAGGATCCATGTCCTCCACCCTTCACTAACTATGATGACGGCACAGTGATACCCAAGTGCCTGCTGTTCCTCAAGGTCTTAGGCCCATGGGAGAACATGCTCAGTTTCTGCAATATGTTTAATGGTTCCCTCGCTATACTGACTGGAGACCTCCACAACATCGTCTACAACTACCTTACTGACACTGCAGACTTGAAGGATCACTGTTTCTGGATCGGAGGCACGGACCAAGAAGAGGAGGGGACCTGGGTGTGGGAGCATGATGGCTCAAGTATATCCATGCGCGGCCCTCACTGGAATCCCTGTGCTCCGGAACCCAACGGTGACACTAAAGAAAATTATATGGCCATCTGTCCTAAGAAGTTTTATTTCAGTGACTATCCAGGAGATGCTGAGCACTACGCCATCTGTCAGTACTTTCCTTAA

MKWIATLAVAAAAVVLSSAQDPCPPPFTNYDDGTVIPKCLLFLKVLGPWENMLSFCNMFNGSLAILTGDLHNIVYNYLTDTADLKDHCFWIGGTDQEEEGTWVWEHDGSSISMRGPHWNPCAPEPNGDTKENYMAICPKKFYFSDYPGDAEHYAICQYFP

transcript_transcript/30605_Pt_Mix_transcript30605/f10p0/1127

ATGCGAATCTACTTGCTTCTGGCGGTGGCGCTGGCGGTGATCTGCTCCGTAGCAGCCCAAGGCCGTGTGTTGGCGTTGCCGGAAATAGAACTATGTGATAATCGCCCAAAGCAGTGGAAGTTCCGCAACCACTATTATTTCTTCTCGTGGGACCAGGATGGCCCAGACTTCAAGGAAGTAAATCCTAAAACAGGACAACTGGAAGGTAGCAAGGTTGACTGGCTGAAAGCTCGCAACTTGTGCCGTCAGCGATGCATGGACGCTGTCGGCATGGAGAGCGAAGAGGAGAACAACATGATTTTCGACTTTATCAAAAGACGCAACATCACGTACATCTGGACGTCTGGCCGCCTCTGTGACTTCAAGGGGTGCGATGAGCGCGAGGACCTGAAGCCCATCAGTGTCAAGGGATGGTTCTTCTCCAACACCAACACTAAGATGGCCCCGACCAACGCATCGCCACCAGGCTGGAAGTACCAGCCATGGAGCGACAAGGGCCACACCGGTGGACCGCAGCCAGACAACGCTGAGTTCGATATCAACCAGACATCGGAGTCTTGTCTCGGCGTACTCAACAATCTGTACAATGACGGCATCAAATGGCACGACATTGCCTGCTACCACAAGAAGCCCTTCATTTGTGAGGACAGTGATGAACTACTTCGGTACATCGAGGGACAAAAGCAGCAACTGCAACAACAAAACCGCCCAGGGAACCAGGGACAGGGAAACCGTGGACAAGGAAACCGTGGACAAGGGAATAACGACAACGCCAACCAGCGCGCACGAGCACATTTTGGTTAA

MRIYLLLAVALAVICSVAAQGRVLALPEIELCDNRPKQWKFRNHYYFFSWDQDGPDFKEVNPKTGQLEGSKVDWLKARNLCRQRCMDAVGMESEEENNMIFDFIKRRNITYIWTSGRLCDFKGCDEREDLKPISVKGWFFSNTNTKMAPTNASPPGWKYQPWSDKGHTGGPQPDNAEFDINQTSESCLGVLNNLYNDGIKWHDIACYHKKPFICEDSDELLRYIEGQKQQLQQQNRPGNQGQGNRGQGNRGQGNNDNANQRARAHFG

transcript_transcript/31983_Pt_Mix_transcript31983/f5p0/1065

ATGAAAGTCTCCCTGGTCATCCTACTCGCGGCTGCTGCGTCTGTGGCACATGGGCTTGTGTACAGGAGTGACGGCTGCCCAGAACCATACGAGCGCCTGGATGACGCGCGCTGTATCCTGGCGGATCCCTTCAAGGCGAAGCAATACACTGAGGCCATCGACTACTGCAACAGTCATGGCGGGGACCTGCTCACTTACGACAACTGCGACGACTTGCTGCTCATATGGGACTTCATACACAGTGATGCGACTCTAGCGGCCAAATCATACTGGCTTGGGGCGACGGACCAGGCGGAGGAAGGCAAGTGGGTCTTCACACACAACCAAGCGGCCGTTCCGATGGGCAATCCTTTCTGGAAGCACGACCAGCCTTCCACCAGCACCTCCTACAACTGTGCCTCCCTTAGTTCTGCTGCTGATCACCGCTGGATCGACCTCTCCTGTACCTCCTCCGCCAACACCATCTGCCTGCGTGAAGGGTAG

MKVSLVILLAAAASVAHGLVYRSDGCPEPYERLDDARCILADPFKAKQYTEAIDYCNSHGGDLLTYDNCDDLLLIWDFIHSDATLAAKSYWLGATDQAEEGKWVFTHNQAAVPMGNPFWKHDQPSTSTSYNCASLSSAADHRWIDLSCTSSANTICLREG

transcript_transcript/34264_Pt_Mix_transcript34264/f2p0/871

ATGCCCTCTCCAGGTATCTCCGGTGACTCTTCGGGGGGCTGCAGCTCCCCGTACACTATGGTTGCTGGCCGCTGCTTATACATTAACTTCGCTACCTCTGGCACTTGGGACCGCTTCCACTCCATGTACTGCAAAGACACAGGAGGGAATGACCTGGTGACAGTGGACGACGCTAACTTCCTTGCCGACCTTGTTCAGTACATCACAAGTATTGGATGGCAAGACGGTTTGTTCTGGATCGGTGCCTCGGACAAGAGTCACGAGGGCCACTGGATATGGCCTAACGGCTCCCCCGTCAAATTGGGCACACCCTTCTGGGGCACGTACGGCTGCTACAACCAGCAGTATCCCGATGGAGGAACCAATGAAAACTGCGCAATACTAGATGGAAATGCAAATCTATATTTCAACGACTTTAAATGTGACACAGCATTTGCTGTCTTCGGTATCTGCGAGAAGAATATCTAG

MPSPGISGDSSGGCSSPYTMVAGRCLYINFATSGTWDRFHSMYCKDTGGNDLVTVDDANFLADLVQYITSIGWQDGLFWIGASDKSHEGHWIWPNGSPVKLGTPFWGTYGCYNQQYPDGGTNENCAILDGNANLYFNDFKCDTAFAVFGICEKNI

transcript_transcript/33261_Pt_Mix_transcript33261/f6p0/926

ATGACTGGCTTCAGGCTGCTGTCTTTAGTGGGAATCCTGGCCGTGGTGGCGGCGACGAACATCAAGCCAGCAAATACCAAGTGCCACAGTCCCTTCACGGAGATTAGTGGTCGCTGCGTGCATATTGATGTCTCCAAAACCGGCACGTGGCAAAATATGCGGAAGTTTTGTCAGCAACTTGGCGGTGACCTGGTCAATCTTTCTGATCTGCAATTCTACGGTGACATCCTTTTGTACATTGAAAGCTTACATTTGCCACACGCTAGTTTTTGGATCGGTGCTACGGACGAAGCGACGGAGGACGTCTGGATGTGGACAGATGGGACGCCAGTCAGGATGGGCACTCCTTTTTGGGCTAACTATGGCGATAATAATTATCAAATGCCTACTGGAGGAGAGAAACAGAACTGTGTTATGCTTGATGTAAACTTTCATTACTATTTCAATGATTTTATTTGTTCAAACACAGATGTAAGTCCGATTTGTGAGAAGTAA

MTGFRLLSLVGILAVVAATNIKPANTKCHSPFTEISGRCVHIDVSKTGTWQNMRKFCQQLGGDLVNLSDLQFYGDILLYIESLHLPHASFWIGATDEATEDVWMWTDGTPVRMGTPFWANYGDNNYQMPTGGEKQNCVMLDVNFHYYFNDFICSNTDVSPICEK

transcript_transcript/34078_Pt_Mix_transcript34078/f8p0/883

ATGAAGTGGATAACGACGCTTGCTGTGGCTGCAGCCGCTGTTGGCTTTTCTTCGACAGAGGATGTATGTCCGCCACCCTTCACTAACTATGATCCAGGCTCGGTGGTGCCCAAGTGCCTGACCTTCCTCTCCTCCGCGGGCACGTGGAGCAGCATGCTTGAGGTATGCAAAATGATGTCAGGTTCCCTCGCTGTGGTAAACGACGACCTCCACAACATCGTCTACAAGCACATCATTAACACTCCAGCCTTAACGGATAAATGCTTCTGGATCGGAGGCACGGACAAATTTCACGAAGGGACCTGGGTGTGGGAGCACGATGGCTCAGAGATACCCCTGGGCGGCCCTCACTGGGATCCCTGTGAACCGGAACCCAACGGAGACGAAAAGGAAAATTCCCTGGCCATCTGTCCGAGTAGATATTACTATAAGGACTATCCATCTGACCGCGAGCACCACGGCATCTGTCAGTACTTTCCTTAA

MKWITTLAVAAAAVGFSSTEDVCPPPFTNYDPGSVVPKCLTFLSSAGTWSSMLEVCKMMSGSLAVVNDDLHNIVYKHIINTPALTDKCFWIGGTDKFHEGTWVWEHDGSEIPLGGPHWDPCEPEPNGDEKENSLAICPSRYYYKDYPSDREHHGICQYFP

transcript_transcript/21663_Pt_Mix_transcript21663/f9p0/1777

ATGAAGACTTCACTTGGCTGCTTACACGCGGCCCTCGCTCTTGTCCTCGTCCTCGCCCTGCTTCAGCCAGCAGAGGGAGACTGTGGACGAAGTGACCAAATCAAGTGTAGGACTGGGAATCAATGTATCTATAAAAGCTACATTTGCAACAAACAATCAGAATGTCCAGACGGTTCAGATGAGGACCCTAAAATGTGTAAGTTTTGGCCGACGAAGAGTCAAGAATGCCGCTATGAAAGTAGCAGTTATATCTACCATAACGGAAACTGTCGAACAGTGGATTATATGTGCCAGAACAGCGATCGAGCCACGAACATTTATCACCAAGTTTGCAAGGTTATTCTTCGGCCAAAACTGGAGTTCGAGGACGAAGGAATGAGCACGAGTGGTGAGCTGCTCGCTTTGCTGAACTCAGCTGTTAACCACACTCTGAACCAAAAGTTACCAGACTGTCCCATGCTGTACTCCCGCGTTGGAAATGAATGCCTTTCCTTTTTCTCACCAGCCAGGGTGTCGTGGGCCGAAGCTCGACAGTTTTGTCTTAGCATCTACGGTGACTTGTGGCAAGCAAAGGATCTGGCAGCGTATGGGCGCCTAATGGAGTACATGCGGGAAGAAAAATTGACCGCTAATTACTGGATTGGTGGACGGTACGACATCGATACCAACGCATGGTCGTGGACGACGGATGACTCCACTATGCCCCTGGGCGTTCCATTCTGGGCAATTAGGTACGTGGACTCCTGCGTGCAAAGGGGACCTCCACACACCGACCCTTATTCCGCACCGCCCGCAGCTCTACCCGGGGCCCGCTGTTATCGAGCCGTCTTATCTCCACAACAAAGATCTCCGGGCTGGTGTTCTGCAATGACCTACGAGCACTTCTACTACTGGACTGACGAGATGTGTGATGAAGCCTACAGCCCACTGTGCACCTTCACTGGCCCCGTCGCCGCCCCCATTGTGGCTGACGCTCATTAG

MKTSLGCLHAALALVLVLALLQPAEGDCGRSDQIKCRTGNQCIYKSYICNKQSECPDGSDEDPKMCKFWPTKSQECRYESSSYIYHNGNCRTVDYMCQNSDRATNIYHQVCKVILRPKLEFEDEGMSTSGELLALLNSAVNHTLNQKLPDCPMLYSRVGNECLSFFSPARVSWAEARQFCLSIYGDLWQAKDLAAYGRLMEYMREEKLTANYWIGGRYDIDTNAWSWTTDDSTMPLGVPFWAIRYVDSCVQRGPPHTDPYSAPPAALPGARCYRAVLSPQQRSPGWCSAMTYEHFYYWTDEMCDEAYSPLCTFTGPVAAPIVADAH

transcript_transcript/35714_Pt_Mix_transcript35714/f33p0/682

ATGAGCCGCTACTTGCTCCTCGCATTCTTCGGAGCCTTGACTGTGGAGGCGACGACACATGTGAACTCATCTGACAGTATCTCCGGTGACTCTTCGGGGGGCTGCAGCTCCCCGTACACTATGGTTGCTGGCCGCTGCTTATACATTAACTTCGCTACCTCTGGCACTTGGGACCGCTTCCACTCCATGTACTGCAAAGACACAGGAGGGAATGACCTGGTGACAGTGGACGACGCTAACTTCCTTGCCGACCTTGTTCAGTACATCACAAGTATTGGATGGCAAGACGGTTTGTTCTGGATCGGTGCCTCGGACAAGGGTCACGAGGGCCACTGGATATGGCCTAACGGCTCCCCCGTCAAGATGGGCACACCCTTCTGGGGCACGTACGGCTGCTACAACCAGCAGTATCCCGATGGAGGAACCAATGAAAACTGCGCAATACTAGATGGAAATGCAAATCTATATTTCAACGACTTTAAATGTGACACAGCATTTGCTGTCTTCGGTATCTGCGAGAAGAATATCTAG

MSRYLLLAFFGALTVEATTHVNSSDSISGDSSGGCSSPYTMVAGRCLYINFATSGTWDRFHSMYCKDTGGNDLVTVDDANFLADLVQYITSIGWQDGLFWIGASDKGHEGHWIWPNGSPVKMGTPFWGTYGCYNQQYPDGGTNENCAILDGNANLYFNDFKCDTAFAVFGICEKNI

transcript_transcript/35852_Pt_Mix_transcript35852/f17p0/627

ATGAAGTGGGGCGTCTCCCTCGCCGTGGCTGCAGCCGCGCTCAGCCTCGCCTCAGTTGAGGCCACGTGTCCCGAAGACTACATTAACATTGGGGATGAGTACAGGGACGTGTGCATCTTTATTCATCGGCCCAAGGACAAGTGGCACGCTATGAGGGCATCCTGCCAAGATATGGGACTGGACTTGGCCACGCTTACTGGAAACCTCCACACCAAGGTGATCCAGTACATTAACAACCATGCAGCTGAGGACCTGAAGGGCGAGACGTTCTGGATAGGAGGGACAGACGAAGTATTGGACGGCAACTGGAAATGGATACATGACAAATCAGAAATTCCTCTCGGCACGCCCCATTGGTACCCTTGCAACAGGAAACAGGAGCCTGACGGCGGCACTAATCAGAACTACTTGTGCCTTCCACACCCAAAATTCTACTTCCAAAGCTGTGACGGCGACGACAAGCACATGGGAATCTGTCAACTCTTCCCTGACTCATTATAA

MKWGVSLAVAAAALSLASVEATCPEDYINIGDEYRDVCIFIHRPKDKWHAMRASCQDMGLDLATLTGNLHTKVIQYINNHAAEDLKGETFWIGGTDEVLDGNWKWIHDKSEIPLGTPHWYPCNRKQEPDGGTNQNYLCLPHPKFYFQSCDGDDKHMGICQLFPDSL

transcript_transcript/36166_Pt_Mix_transcript36166/f25p0/675

ATGACGCCACTCCACCTTCTCCTGTGCGTGGCGGCTGTGCTGCCTCTCACCACCACTGGAGATGAAGACAGTGAGAAGGATTGGTACACCACCTGGCCATGGGACACCTCCACCCCCTATCCTAATACTAGGACACCATCAACCAATGAATGCGACATGCCCTTCCTGCGTGTGGGGGATCGGTGCGTGTTGGTGGTTCCCTTCGTGACTGGGACGTGGGAGGAGTCGAGGTACTATTGCCACACACAGAACTCAGAGCTCGTGCAGACAGACAACATTAATTTCTTCTACAATCTGCTTAACTACCTCAGAGATGAAGGGCTGGAGCAACATAGCTACTGGCTGGGCGCGAGGGACATAGATGTAGAAGGAGAATTTCGCTGGACTATCGGAGACGGCTTGGTACCAATGGGCTCTCCTTTCTGGGCCATCAAATACTCCTCCAGTACCTACTACGACATTGAGCCGCTTGGGACCACCACCTCCAATTGTATCCGTATGGACCACGTGCGCCACTTGTATCTAGATGACGAAGACTGTGACCACGAGTACTCCTTCATGTGCGAGAAGAAGCTGAGGGTGTGA

MTPLHLLLCVAAVLPLTTTGDEDSEKDWYTTWPWDTSTPYPNTRTPSTNECDMPFLRVGDRCVLVVPFVTGTWEESRYYCHTQNSELVQTDNINFFYNLLNYLRDEGLEQHSYWLGARDIDVEGEFRWTIGDGLVPMGSPFWAIKYSSSTYYDIEPLGTTTSNCIRMDHVRHLYLDDEDCDHEYSFMCEKKLRV

transcript_transcript/36378_Pt_Mix_transcript36378/f2p0/669

ATGATGCTGCGATTGTTGCTCCTCACGCTCTCCACAGCCGTCCTGGCTGCTGGTCTGAATTGTTCCCGCCCATTCAATGATATTGGAGGGCGGTGCATCTTTATCGACCCCTGGGAGTTGGGCACCATGTCAGAAATCCGGGATTTCTGCAAAGCCTATGGCGGAGACCTCATCTGGTTTGACAACGACACAGATTGTGACTTCTACCGAGATCTTCTGACACACATCCATGAGAATAGCCTCAATGAGAGGGACTACTGGATGGGGATCACAGATGAGGGGCATAATGAAGTTTGGAAATACTTGAGAAATAATCAGGTGGTGCGTGATGGTCCCCCATACTGGGACACAGGCTTTCCTACTACCTCAACTACAGAGAACTGTGCCATCCTCGCAATGACAAGAGGCTACTACTGGGAAGAGGTCGCATGTGATTCCACATACTCAACTATCTGTCGTAAAACCTAG

MMLRLLLLTLSTAVLAAGLNCSRPFNDIGGRCIFIDPWELGTMSEIRDFCKAYGGDLIWFDNDTDCDFYRDLLTHIHENSLNERDYWMGITDEGHNEVWKYLRNNQVVRDGPPYWDTGFPTTSTTENCAILAMTRGYYWEEVACDSTYSTICRKT

transcript_transcript/37789_Pt_Mix_transcript37789/f10p0/527

ATGAAGATTGTCATAGCGTTGATATTGACTTTGCTCTCCGTGGCGACTGCCCAGAATAATGACTGCACAGATGGTTTTCAACAAGTGGGATCTGCCTGCTACATGATTCCTAGCGAATCTCATGGATGGTACGAGGCCATGCATCACTGCAAATCACTAACCCCATCCAACGGTAAGATGGCGGCGTTGGCGCGAGTGGAAACATGCACCCAGCTTCATTCAGTTTGGGAGTTCATCAATAAAGAGGGATATCCTATCGTCGACTACTGGCTTGGCGGGACCACATCATTGAATAAAAACACCTTCAGGTGGGACTCGACTGGTGAACTAGTGCCTATGGGCGTGCCCTTTTGGTTTCCGGGTCAGCCCGACTTTGCACCCGACGAAGAAAGAAGTCTCTCCCTTTCCAAGACCGGTTACTTCGCTGATGAAGAAGAACATATCAAACAAAAATTCATATGTCAGCTCTTGTAG

MKIVIALILTLLSVATAQNNDCTDGFQQVGSACYMIPSESHGWYEAMHHCKSLTPSNGKMAALARVETCTQLHSVWEFINKEGYPIVDYWLGGTTSLNKNTFRWDSTGELVPMGVPFWFPGQPDFAPDEERSLSLSKTGYFADEEEHIKQKFICQLL

transcript_transcript/30028_Pt_Mix_transcript30028/f3p0/1231

ATGGAAGACAGGAAGATGATAACAGCTCTGGTGGTGGTGTGGGCGGCTGCCCTCGTAACTCCTACTTCTGCCGTGAAAGTCTCATGCAATGGCGGATTCGTTCTCATCGGTGGCACCACCTGCATTAAAGTGTTCGAAAACCAAAAGTCATGGAAAGAAGCAAGTGATGCTTGTAGATCCTTAGAAAATTTTGCCTTTGGTTCCCCACACCTAGCCCGTATTAATGACTGCTCACTATTATCCAGCCTGTTCGATTACGTCTATTATCAGCTGAATATAACAGCAGACCTTTGGCTGGGAGGAACCGATTCCTTGAAAGAGGGTGAGTGGGAGTGGGAAAATGGTGATCCTGTACCAGTAGGAATTCCATTCTGGCACCCCCTTCAACCTGATGGAGGACTTAACGAAAACAAGCTGGTATTCGCTCATAATGGCTTCTTCGCAGATGGACACGAGGACAGGAAATACGGTTATATTTGCCAGTACCAGAGTTAA

MEDRKMITALVVVWAAALVTPTSAVKVSCNGGFVLIGGTTCIKVFENQKSWKEASDACRSLENFAFGSPHLARINDCSLLSSLFDYVYYQLNITADLWLGGTDSLKEGEWEWENGDPVPVGIPFWHPLQPDGGLNENKLVFAHNGFFADGHEDRKYGYICQYQS

transcript_transcript/32380_Pt_Mix_transcript32380/f5p0/1028

ATGGCCATCAACACAGGGACTCCTTTCCTGCTGCTGCTGTTGCAGCTGCTTCCATTACTGCCGTGTCTCCATGCAGCTCCAAACCGCGTCACAGAGTGCACGCCGCCCTTCACTGCGGCGGTGGGCGGGAAGTGCATCCTCGTGAGCTCCACAAGCAAGGGTTCCTGGTACAACATGAAAGACTTTTGTGAGACTCTTGGCGGTCGGCTGTTGACCATCAACACAGAGAACATTCACTACTACGTCGTCAAGTACTTGGAGAACAACGGATATACTGGCCGAGATTACTGGGTCGGCGCAAACAACGAGCACGCTGACGGGCAGTACCACTGGCTGGACGGGAGCAAGGTCAAGATGGGCACGCCTTTCTGGGGCATTCTAAACGACCATCAGGAGCCAGGCGGGGGAGAGTCCGAACACTGTGCTATCCTATTTAAAAGTGACTTTTATTTCATGCATGATTTAGCATGTAACAAAGAAGTGTACCCTATCTGCGAGGCTAAATAA

MAINTGTPFLLLLLQLLPLLPCLHAAPNRVTECTPPFTAAVGGKCILVSSTSKGSWYNMKDFCETLGGRLLTINTENIHYYVVKYLENNGYTGRDYWVGANNEHADGQYHWLDGSKVKMGTPFWGILNDHQEPGGGESEHCAILFKSDFYFMHDLACNKEVYPICEAK

transcript_transcript/11664_Pt_Mix_transcript11664/f2p0/2558

ATGACGGGCTGGTGGTGCACACTTCTGCTATTGGTGGTGGAAGTGATGGGGACAGGTCCTCCGTCAGATTTTGTGGGCCGACTGAATGCACTTGACTTTACGGATGGTAACTACTATATAATGGCCAGAGATGGGATGACAGCCTCGCTAGATGACCTGCAACGCGGCCAAGATGGACTCATAATAAAACTAAACAACGTTATCAGGACACTCAAGGAAGACGCATTAAAACTGAATAACATCTTCTACAAACTTGGCCGACATGACACCAACCTGGAAGATGTTGACTCCAACCTCCATCGGTTGGGTGACTCATCTCGACGAACTGAAGCCAAACTCAACAAGCACGCCTATAATGTTGATTTCCTTCGCCGTCGAGTAGAGGATCTTCATGGGCAGTGGCAAGGTGTAGCAGGCGTACAGATGGAAATGGCAGCAGTGCGTTCCATCACTGATCGTTTGGATGACCGACTGACGGATGCCATCGCCCGTTTGGGGTTGGTTGAAAACACTACTAAAACCCTGGAGAAAAGATCAACACCAGGTCACCTTGATGTTTTCCAAGCAGCTGAGAGGCCTCCTACCCTCACTGTGTACCCTTACCCTACCCGAGATTCATGTCGGCTGGATTTTGAGCGTGTTGGGAGTGACTGTTATTGGTTTGGTGGTGAGGAATTGACCTTCACAGAGGCCACAGCTGCCTGCTTTGGCCATGGAGGTAGCCTGTTAACGCTGCCTTCTCCTGGCACCCAGCTGACTTTGCTCCTTAACCGCCTCAAAAAAGATACCCTATATTGGACCGGTGGGACAGACGCCTTTCGTCACGGTTACTGGGTTTACCTTATGACAGCCCAGCCCGTCATGCCCTTACATTGGGCTGGAGAGAATGCATCCAATCCACGTAGCGTCGACAATCAACCTGATTCAGACATCCAACACTGTGCCGGTCTGACCTCCCACGGCCTCACACCGCGCCCATGCACCCAACGTCACCCTTATATATGCCACGTCATGTGA

MTGWWCTLLLLVVEVMGTGPPSDFVGRLNALDFTDGNYYIMARDGMTASLDDLQRGQDGLIIKLNNVIRTLKEDALKLNNIFYKLGRHDTNLEDVDSNLHRLGDSSRRTEAKLNKHAYNVDFLRRRVEDLHGQWQGVAGVQMEMAAVRSITDRLDDRLTDAIARLGLVENTTKTLEKRSTPGHLDVFQAAERPPTLTVYPYPTRDSCRLDFERVGSDCYWFGGEELTFTEATAACFGHGGSLLTLPSPGTQLTLLLNRLKKDTLYWTGGTDAFRHGYWVYLMTAQPVMPLHWAGENASNPRSVDNQPDSDIQHCAGLTSHGLTPRPCTQRHPYICHVM

transcript_transcript/32246_Pt_Mix_transcript32246/f7p0/1020

ATGGGGCAGCCAGTATATAATCCAGGTCAGCCATGCATGATCCCCATCGCTGGAGGTTTTGCTCCCGGCAAGATCCTCCACGTGACGGGCACCTTCACTCCCGCTGCTAACAGCTTTGTTATGAAACTTCAGTCAGGACAAGTTGGGGACCCAACTGATGAGATTGGCTTGTGTATCTACGGACGGGTGGCTGAGGGTGTGATTGGTCGCAATGCCTTCACCCGGGCCGCTGGCTGGGGGCAGGAGGAGGCCACCAGTTCTCCTGCCTTTGCTCGTGGCCAGAACTTTGACATCACGATCCTGTGTGACCCAGCACAGTTCAAGATTGCCCTCAACCAGAACCACTTTGCTGAGTTCAACCACCGCGCCAACCCTGCCAGCCTCACCTTCCTCAACATCTCCAGCACTAGCCAAGATGTCACTGTTGCCTGCGTTTGGGTTGAAGATGGGCCAGGGGCACCTCAGCCACAACCAGGCTTTGCCCAGCCCCCTGTCTCTGGCTTTGAACCCCCACCACCATACTCTGGAGGTCCTGGATTTGCCCCACAATACTCCAGTCCCCAAATGTACCAGCAGGCTGTCCCGAACTACCAGCAGCCAATCCCACCACAGCCGTACATGCCACCCGGAGCCCCGCCGCCGCAGTATGGTGTAGGTCCAAGGTTCTAG

MGQPVYNPGQPCMIPIAGGFAPGKILHVTGTFTPAANSFVMKLQSGQVGDPTDEIGLCIYGRVAEGVIGRNAFTRAAGWGQEEATSSPAFARGQNFDITILCDPAQFKIALNQNHFAEFNHRANPASLTFLNISSTSQDVTVACVWVEDGPGAPQPQPGFAQPPVSGFEPPPPYSGGPGFAPQYSSPQMYQQAVPNYQQPIPPQPYMPPGAPPPQYGVGPRF

transcript_transcript/30784_Pt_Mix_transcript30784/f3p0/1170

ATGATCCCCATCGCTGGAGGTTTTGCTCCCGGCAAGATCCTCCACGTGACGGGCACCTTCACTCCCGCTGCTAACAGCTTTGTTATGAAACTTCAGTCAGGACAAGTTGGGGACCCAACTGATGAGATTGGCTTGTGTATCTACGGACGGGTGGCTGAGGGTGTGATTGGTCGCAATGCCTTCACCCGGGCCGCTGGCTGGGGGCAGGAGGAGGCCACCAGTTCTCCTGCCTTTGCTCGTGGCCAGAACTTTGACATCACGATCCTGTGTGACCCAGCACAGTTCAAGATTGCCCTCAACCAGAACCACTTTGCTGAGTTCAACCACCGCGCCAACCCTGCCAGCCTCACCTTCCTCAACATCTCCAGCACTAGCCAAGATGTCACTGTTGCCTGCGTTTGGGTTGAAGATGGGCCAGGGGCACCTCAGCCACAACCAGGCTTTGCCCAGCCCCCTGTCTCTGGCTTTGAACCCCCACCACCATACTCTGGAGGTCCTGGATTTGCCCCACAATACTCCAGTCCCCAAATGTACCAGCAGGCTGTCCCGAACTACCAGCAGCCAATCCCACCACAGCCGTACATGCCACCCGGAGCCCCGCCGCCGCAGTATGGTGTAGGTCCAAGGTTCTAG

MIPIAGGFAPGKILHVTGTFTPAANSFVMKLQSGQVGDPTDEIGLCIYGRVAEGVIGRNAFTRAAGWGQEEATSSPAFARGQNFDITILCDPAQFKIALNQNHFAEFNHRANPASLTFLNISSTSQDVTVACVWVEDGPGAPQPQPGFAQPPVSGFEPPPPYSGGPGFAPQYSSPQMYQQAVPNYQQPIPPQPYMPPGAPPPQYGVGPRF

transcript_transcript/16321_Pt_Mix_transcript16321/f2p0/2175

ATGTACAGCGGCGGTGTCATGGAGGAACGGAGGGACTATGTGACTCTTTCCCTGCCGGCCGTACTATTGCCTCCTCCTGCTGCTGCTGCACCTTCCCGCTCCTGGAGGAGGCGATGGAAACAACTTCCACGTCTGCAGAGGGTGGTGGTGGTGATGGTTGCTGTAGGCCTACTGGTTGGAGTAGCACTGCTAGTGACTACCTACCTGTCAGGTCCTTCGCACTCATCCTCCCTCTCCCACACACCTCAACCTTCCCATTTCTCTTACAAGTTGGAGAATAATCTTACCCCACCTCCAGTAATGGTACATCTCCCCGAACCCCATCAAGATAAGGTGGAGACAGTACCAGAGGAGCAGGCCCTGAAGGATGATCCAGGACATGATGGCCGGGACCAGATAGTGCCACCACCAGGATTACCTCAAAAAAGATCAGCAAGGCAGGAGGCAGTAGTTGCAGCCATGAAGCATGCCTGGAAAGGATATAAGACTTATGCATGGGGCCATGATCACTTGAGACCTGTCTCACAGACTCGTAGTGATTGGCTCCGCCTTGGTCTCACCCTTGTGGATGCCCTTGACACTCTCTGGATCATGGACTTACGAGAAGAGTTCAGTGAGGCAAGAGAATGGGTAGCAACACAACTCAACTTTAATCTCAACCAGGATGTGAATCTCTTTGAGACAACCATCAGGGTTTTAGGTGGCCTTCTCTCCACCTATCACCTCACAAAGGAGCAACTTTTCCTTGACAAGGCTGTTGACCTTGGTGAGAGGCTGGTGTCAGGATTCAACAGTGGCTCTGGAGTTCCCTTTGCTGATGTAAATTTGTATTCACGTCGTGCCTCCAAGCCAAAGTGGGGGCCAGACTCCTCAACGTCTGAGGTCACTACCATACAGCTGGAGTTCCGAGACCTCTCTCGAATCACTGGCAATCCTATCTTTGAGGAGAAGGCCAGCTTTGTGAGTGAGCACATTCACAAACTTCCTAAGGTTGAAGGTTTGGTCCCCATCTTCATTAATGCCCAGACTGGCCAGTGGCGGTCCCACTCCACCATCACCCTTGGGGCACGAGGGGACTCTTACTATGAGTACCTCCTCAAACAGTGGATACAAACTGGACGGACTGTGGATTACCTGCGAATGGACTACAATGAAAGTGTATCAGGTATGGAGCATCTGCTGGCAGCACGTACACAACCTTCTAAGCTCCTCTTTTTTGGTGAACTTCACGGTAGCACCAAAAACTTTGTCAATAAGATGGATGAACTCACTTGCTACTTGCCAGGAACATTGGCCCTTGGTGTCCACTATGGAATGCCTCGACATCATCTGGACTTGGCACAGGACCTCATATACACCTGTGTTCAGACATGGCTCAGGCAGCCTACACATCTAGCTGCTGAGATCAGCTATTTCAACACCCAGCCCAATACCATGGATGGAGAAGACTTCTATGTGAAGAACAATGATGCCCACTATCTGCTGAGGCCGGAGACCATAGAGTCATTGTGGTACCTTTACCACTTAACGGGCAATACCACCTATCAAGACTGGGGATGGCAAATGTTTCAGGGTATTGAAAAATACTGCAAAGTGGAGAACGGTTACACTTCCATTGGCAACGTTCGCAGTGCTACAGATACCAAGCCAAAGGACAAAATGGAATCCTTTTTCCTTGGTGAAACTCTGAAGTACCTCTATTTGCTCTTCATGGAGGATCAGACTGCCTTCAGTGTGGATAAGTGGGTATTCAATAGTGAGGCACACCCACTTCCCATATATTCCCGCTAA

MYSGGVMEERRDYVTLSLPAVLLPPPAAAAPSRSWRRRWKQLPRLQRVVVVMVAVGLLVGVALLVTTYLSGPSHSSSLSHTPQPSHFSYKLENNLTPPPVMVHLPEPHQDKVETVPEEQALKDDPGHDGRDQIVPPPGLPQKRSARQEAVVAAMKHAWKGYKTYAWGHDHLRPVSQTRSDWLRLGLTLVDALDTLWIMDLREEFSEAREWVATQLNFNLNQDVNLFETTIRVLGGLLSTYHLTKEQLFLDKAVDLGERLVSGFNSGSGVPFADVNLYSRRASKPKWGPDSSTSEVTTIQLEFRDLSRITGNPIFEEKASFVSEHIHKLPKVEGLVPIFINAQTGQWRSHSTITLGARGDSYYEYLLKQWIQTGRTVDYLRMDYNESVSGMEHLLAARTQPSKLLFFGELHGSTKNFVNKMDELTCYLPGTLALGVHYGMPRHHLDLAQDLIYTCVQTWLRQPTHLAAEISYFNTQPNTMDGEDFYVKNNDAHYLLRPETIESLWYLYHLTGNTTYQDWGWQMFQGIEKYCKVENGYTSIGNVRSATDTKPKDKMESFFLGETLKYLYLLFMEDQTAFSVDKWVFNSEAHPLPIYSR

transcript_transcript/1834_Pt_Mix_transcript1834/f4p0/4383

ATGTGGAGTGCGAGATTGGGCGATGGCAGGTTGGCGCTGGTCTTGGTCGTGTGGCAGGTCGCGGCGTGCGGCGTGGCGTGCCAGGGTGTGGGGCGGTACAGCGGCGGCGACAAGTGTGAGAGCCGCGCCCTCAGTGCTCGAGAGCGCGCCCTCTCCTGCTCCCTCAAGGCGCTGGAGCAGGAGACGCGAGTGGCCAACCTGAGCCGAGTGGCGGCCGACAGTGTGGTGCGCCTCAGCCTCGGCTGTAACGCGATGTTCTACTTCCCCAGTGTGTTGTCCCCGCACACCCTGTCTGGCTTCGTCAGGGTGCGGGAGCTCAATGTGGAGTTCTGCAAACTCAGTGAACTGGAAGATAATGTGTTTATTAACCTAAAAAATCTTAGAAACCTAACCATACGGACTAGAAACTGGGACTGGCCGCGGCACAGCCTCGACCTGCGACCAGATGTGTTCCGTCCTCTGCACCAGCTACAGCGGCTCAACCTCAGCACCAACAGCATGTGGGAGCTGCCAGCTGGTGTCTTCTGCCACCTGGGCAGCCTCAAACAGCTCAATCTAAGTCATAACCACCTGCAACAAATGACACAGCTGGGGTTTGGAGAGAGCGGGGCTGGCAGGGGCGGCCCAACCTGCCGCTCTGGCCTGCGTTCCCTGGACCTCTCACACAACGACGTGACGGAGCTGGTGTCTGGCGCTCTGCAGGGCCTGTTCCGCTTGCAGGAGCTGTGGCTACACCACAACGATCTGGCCAAGCTGGACGACGACGCCTTCAAGGGCCTCTCTGGCCTGCACGCCCTCGACCTGTCTCATAACAGGCTGGTGGCGCTGCCCGAGGACGCCTTTGTACACACGCCGGAGCTGGAGTCGCTGCACGCCAGCAACAACTCGCTGGCCGTGCTGGCGCCGGGCATCTTCCGCTCCCTGAAGCACCTGGTGGAGCTCGACCTCTCCCACAACGAACTCAAGTCTGAGTGGCTCACGTCCTCCATCTTCGAGGGCCTCATCAGACTGCGGGTGCTGAGTCTCGCCCACAACAAGATCACGCAGCTCAGCCAACAGGTCTTCCACAATCTATACGGCCTGCAGGTGCTGCAGCTGTCCCACAACCAGCTGGCTACCATCCCTGCAGCTGCATTTGTTCGCTGTGTCAACCTGCACCAGCTGGATCTCTCTTACAACCAGCTGACGGCCATCCCTGACCGCGCCTTCCAGGGCGTGGACGTGCTCAGCTTTCTGGCACTGGACAACAATAGGATCAGCGAGGTGGCGGCTGACTCCCTCGACAACCTCACCAACCTGGCAGACCTCAATCTCAATGGCAACCAGCTGACAGCCATCCCTGATGTGGTGGGGCGCCTGGGGCTGCTCAAGACGCTAGACCTGGGAGAGAACCACATCACCTCGCTGGACAACATGCCGGTGAAGGGGTTGCAGTTCCTGTACGGTCTGCGCCTCGTCAATAACAGGATCTCAGGCAACATCTCCAAGGAGACGTTCAGTGACATCCCCTCCCTTAAGATACTCAATTTAGCAAAGAATTCCATTACCCACATTGAAAGTGGAACCTTTAATAATCATCACAACCTGCAGGCGGTGCGTCTCGATGCAAACAAGCTCACCAGTATTCATAATTTGTTTGAAAATCTTCAAAATTTGGTGTGGTTAAATGTGTCTGATAATAACATAGAAGTTTTTGATTACCACTTTATACCAGTGAGTCTCCAGTGGCTGGATTTACACAAAAACAGAATAAGTGAGTTAAGCAACTACAACGACAGGCAAGACCTCAACCTGCAGACGCTGGACGCGAGCTTCAACTCCCTGGAGTACATAAGCAACATCCAGATCCCGGATAGCGTGCAGCTGCTCTTCCTCAACGACAACAAGATCAACACCGTGGAGCCCTTCACCTTCTTTAAGAAGGAAAACCTTACCAGGGTGGATTTGTTCGCCAACCAGCTGTCCAAGATGGACATGTCTGCGCTGCGCCTCAGCGAGATTCCTGCCAACAGGTCCCTGCCTGAGTTTTATCTGGGTGGTAATCCGTTCGTGTGCGACTGCAAGATGGAATGGCTGCAGCGCATCAACGGCCTGGAGCACGAGAGGCAGAACCCAATCATCATGGACCTCGAGACCATCTACTGCCAGATGCCCTTCGCACGTACAGGTGCCTTCATCCCACTGGTGGAGGTCAATCCCTCGCAGTTCCTGTGTCAGTACGAAACTCACTGCTTCGCGCTGTGCCACTGCTGTGAGTTTGACGCCTGTGACTGCGAGATGACGTGCCCTGACGGCTGCGGCTGTTACCACGACCAATCGTGGCGTTCCAACATCGTGGACTGCTCCCTGCAGGACGTGCAACAAGTGCCTGAGCGGATCCCGATGGACGCCACGCAAGTGTACCTGGACGGCAACAACCTCAAAAACCTCTCCTCGCACTCTTTCATCGGCCGCAAGCACCTGCAGGTGCTGTACGTGAACGCGTCCAGCGTCAAGACGCTCGACAACGGCACCTTCAGCGGCCTGACGCGGCTCACGGCGCTACACCTTGAAGACAACCTGCTGGAAGCCCTGAGGGGTAACGAGTTCCAGGGGCTGCAGGGCGTGCGGGAGCTGTACCTGCACAACAACCACCTGCGCTACATCCACCAGCACACCTTCGCCACGCTCTTCCACCTGGAGACGCTCACGCTCCAGAACAATCTGCTTACCAACTTCCCCGTGTGGCGCCTGGTGGACAACCCATACCTGAGCCGCGTGTCGCTGGGCGCCAACCACTGGTCTTGCCAGTGCCAGTTTGTCGAGTCCTTCGGCATCTGGCTCAACGGCAACGACCGGAAGGTTGTGGACGGCAAGGACATCAAGTGCTACTCGGAGGAGGCAGACGAGGAGCCGGGCTCCTTCATCACGGACTTCAACGTCACCACGTGTATGAACAGCACGTCCACCTCCACCGTGATCCAGCCCATGGTGCTGGGCAACCTGCTGCACCCCGTCATCGCCACCTGCGCCTCCTTCGTGGTGGTGGTGCTGCTGGTGCTGTGCTTCCTGTACCGCGGCGCCCTGCGGGTCTGGGTGTTCTCGCGGTGCGGCTTCAGGATGTGCCACAAGGCCGCCACCACCGACGACCGCGACAAGCTGTTCGATGCCTTCGTGTCGTACAGCTCCAAGGACGAGGCGTGGGTCAACCAGGTGCTGGCAGGGGAGCTGGAGAGGGGCGAGAGGCCCTATAGGGTATGCCTGCACTACCGAGACTTCCCCGTCACTGCTTACATCGCCGACACCATCGTGGAGGCCGTGGAGTCTTCGCGCCGCACCATCATCGTGCTCTCCAAAAACTTTATTGAGAACGAATGGTGCCGCTTCCAGTTCAAGAGCGCCCATCACGAGGTGTTCAAGAAGCGGCGGCAGCGCCTCATCGTGGTGGTGCTGGGCGAGATTCCCTCGCGGGACCTGGACCCCGACCTGCGCCTGTACCTCAAGACCAACACCTGCATCAAGGCCAGCGACAGTCGCTTTTGGGAGAAGCTCAGGTTCGCCATGCCCGACGTGCAGAACAGCCAGAGAGTCGTCCACGCCTACAGCTCCATCCCGGAGAGGTCGTCCTCCTCCACCAACAAGTACAGCGTGAACAGTCCGGCGGGCGTCCACCACAACCTGCACGCCAGCTCGGACGCCTACTGGGCGTAG

MWSARLGDGRLALVLVVWQVAACGVACQGVGRYSGGDKCESRALSARERALSCSLKALEQETRVANLSRVAADSVVRLSLGCNAMFYFPSVLSPHTLSGFVRVRELNVEFCKLSELEDNVFINLKNLRNLTIRTRNWDWPRHSLDLRPDVFRPLHQLQRLNLSTNSMWELPAGVFCHLGSLKQLNLSHNHLQQMTQLGFGESGAGRGGPTCRSGLRSLDLSHNDVTELVSGALQGLFRLQELWLHHNDLAKLDDDAFKGLSGLHALDLSHNRLVALPEDAFVHTPELESLHASNNSLAVLAPGIFRSLKHLVELDLSHNELKSEWLTSSIFEGLIRLRVLSLAHNKITQLSQQVFHNLYGLQVLQLSHNQLATIPAAAFVRCVNLHQLDLSYNQLTAIPDRAFQGVDVLSFLALDNNRISEVAADSLDNLTNLADLNLNGNQLTAIPDVVGRLGLLKTLDLGENHITSLDNMPVKGLQFLYGLRLVNNRISGNISKETFSDIPSLKILNLAKNSITHIESGTFNNHHNLQAVRLDANKLTSIHNLFENLQNLVWLNVSDNNIEVFDYHFIPVSLQWLDLHKNRISELSNYNDRQDLNLQTLDASFNSLEYISNIQIPDSVQLLFLNDNKINTVEPFTFFKKENLTRVDLFANQLSKMDMSALRLSEIPANRSLPEFYLGGNPFVCDCKMEWLQRINGLEHERQNPIIMDLETIYCQMPFARTGAFIPLVEVNPSQFLCQYETHCFALCHCCEFDACDCEMTCPDGCGCYHDQSWRSNIVDCSLQDVQQVPERIPMDATQVYLDGNNLKNLSSHSFIGRKHLQVLYVNASSVKTLDNGTFSGLTRLTALHLEDNLLEALRGNEFQGLQGVRELYLHNNHLRYIHQHTFATLFHLETLTLQNNLLTNFPVWRLVDNPYLSRVSLGANHWSCQCQFVESFGIWLNGNDRKVVDGKDIKCYSEEADEEPGSFITDFNVTTCMNSTSTSTVIQPMVLGNLLHPVIATCASFVVVVLLVLCFLYRGALRVWVFSRCGFRMCHKAATTDDRDKLFDAFVSYSSKDEAWVNQVLAGELERGERPYRVCLHYRDFPVTAYIADTIVEAVESSRRTIIVLSKNFIENEWCRFQFKSAHHEVFKKRRQRLIVVVLGEIPSRDLDPDLRLYLKTNTCIKASDSRFWEKLRFAMPDVQNSQRVVHAYSSIPERSSSSTNKYSVNSPAGVHHNLHASSDAYWA

transcript_transcript/3140_Pt_Mix_transcript3140/f2p0/3854

ATGGCGGGTGAACATCAAGCCATGGCTGCCCTGTGGCTGTGGCTAGCCGTCCTGACGCTGGCCACCACACTCTCCTCTGCCTGCCCCAACTGCTTCAAAAATTTTAAGAGTACTTATTGCCCAATCCCATTTACACCTGAAGAACAGACGTACTCCTTAAAATTATCTAACTATAAAAATGCCGAAGAGAGTGCTCTGACCTACCAGTGTCACCACTTAGCTCCACAGATGTACTTCACCCACCACCAAGGATGCAATTTTTCCACAGTTCAATATGTGGCATTCCAGCGATGTCCGTTACCAAATGTCTCATTCAGTGAGATCTTTACTCAGCTTGGAGTACACCCAGAAAAGATTTTGGAAATGACTTTTAAAAATATTGGGACACGAAAAGATTTGGAACTTAAAACCTGGCACCTGGACGGACTTGTTAATCTTCAGATCTTACAATTAAAGCACAATTTGTTCACTTCATTACATCCAGACGTCCTTAAAGCAACTCCAAACCTGGAACACTTCCTTTTCTCACAAAATGCTATGCCTGCACTTCCTGAAGCTTTATTTGCACATACACCAAAGCTCAAAACAATTAATTTATTGAATAATAAATTTGAAAGCATACCTGAAAATATATTTTTTAATCTTTCAAATTTGACTGTCTTAAGATTGTATGGCAATAATCTAAAGGAGATCAACCCAAAGCTCTTTACTAATACACCTATGGTTTCCAAATTAGAGCTGTCATTTAATGGAATAACCAATGTGACTTCAGATACTTTTAAGTATCTTCCAAAACTACAACATTTATTCATGAAGTTTAATGATATTGAATCCTTACCACCAGACTTATTTCACAACTGCCCTGAACTGCAATCTTTCCATATACACTATAACAAACTTCAATCCCTTCCTTCAGAGCTTTTCAGTAAGTCAAAGAACATAAGTGATTTAGATTTTGATAAAAATGAAATAAAAGAGATTCCTGAAGAATTATTTCATGGGCAGGAGAACCTAACGATTCTAATGATGCAACAAAATGTTCTCAAGAATATTCCTGATGGTGCCTTTAAAGATCTAACAAACCTTGAGAAACTGTCACTGCAAAATAACCCTATCAAGAGTCTGCCACCTGGGAGCTTTGACCACCAGCACAAAATGAAGATACTTAACCTGGCAAACACCAGTCTTACTGATCTGCCTGACAAGATTTTCAAAAACTGTGAATCTTTAGTGGAAATTGACCTTTCAAATAATCATTTGAGTGAATTGAAGAGTACTGTTTTTCCTCATCCTGCTACAGCTCTAAGCATCCTAAAACTCAATAGAAATAGTCTTTCTCTCTCACCCAGAACAAGCGAGCCACAAGCACGGCAGATTCTGGTGGAACAGTTTCCTTTCTCAGACCAAGTCAACTTAACTTATCTTTTTCTACAGAGCAACAGAATCCAAAATATACCTCATGCTCTGAGAAACCTGAAGAAGTTGAAGCATCTGGATTTGAAGAATAATAGCATTGAATACTTAGATTATTATGGATTTTTATTTAGTTCTGATACTACTGCTGATCCAAGTGATGCTCCTTGGGCAAACCTACAAGAACTGCCACAAGCTTTACCCTCACAAGTCATTAAAATTGAATTAAAAGATAATCCTTTGATTTGTGACTGCAGTCTTTATAACTTTGCTCTGTGGTTGCAAGGCAAGATCTCTGAAGGTGATGTCCAGCTGAATGTTGTTGATAAAGCTGATGTCAAATGCTCTATGCCAGATGACAAAAGTGTCCAGAAATTTGTTATGACTGTAGACTTGAGCACACTTGTCTGTAACAGAGAGGAATGCCCTACATCCTGCACCTGTATTACAAGGCCCCATGACAATATGTTCTTTATGGAATGTATTCAGCAAAGATTACAGGCAATTCCTCCACTAAAAGCTTATTTACCACTAGGTAACTATTCAGTCACCCTTAACTTGAAGAACAACTTTATCGCCAGTCTGGAAGGTCTTCAGAGTCCTGAGTACTCCAATGTAGTGAATCTGACAGTGGCTCACAATGCCCTCAAATTCATCAATGAAAGTTACTTGCCAAGAAGACTAAAGGCTTTGGATCTTAGTGGCAATGCTCTCATTCATTCCTCTAAATCTCTTATAGCCTTTCTGAATGTAACCAATCCCACCTTGAGCCTTAGTGGGAATCCATGGGTCTGTGATTGCCAGCTGTTGGACCTCTACAACTTCTTGAGGGATCCCCTCAGAAAGATGGCTGATTCACATCCCATCATGTGTGACAATTCTGAGCCTCTCCTGAGCCTGACAGAGGAGCAGCTCTGCCCGACCATCCAGCAGCCCATGGTGGTTGTCACTATTGCTTCCACCACTGTCTTCTTGGTTTTGTTCTTTGTTTTAGGAACTGTAAGTGTGTACAAGTATCAACAAAACATCAAGGTATGGCTCTATACTCATCAAATGTGTCTGTGGATCATAGCCAAAGAAGAAGCAGACAAGAAAAAATATGATGCCTTCATCAGCTACTCTAACCAAGATGAGGAATATGTCAACAATGTGTTGGTTCCCGGGCTGGAGAGTGGAGAGCCTAAGTACCGCGTGTGCCTCCACTACCGAGACTGGGTGGCTGGAGATTATATCCAGAACCAGATTAACCAAAGCATTGAGGACAGCCACAGGACTATCGTTATACTGTCTTCCAGCTTCATAGAAAATGTTTGGGGACAGATTGAATTTAAAACTGCCCATTCTAAAGCACTTAAAGAAAAAAGCAAGAACATTATTGTCATTGTTTTGGGACAGAGTTGGCTGACTGCCAAGGAGCCCCAGCCTGATTACTCAGATTTATCAGCCCGCCAAGGATGTTCTAGATGA

MAGEHQAMAALWLWLAVLTLATTLSSACPNCFKNFKSTYCPIPFTPEEQTYSLKLSNYKNAEESALTYQCHHLAPQMYFTHHQGCNFSTVQYVAFQRCPLPNVSFSEIFTQLGVHPEKILEMTFKNIGTRKDLELKTWHLDGLVNLQILQLKHNLFTSLHPDVLKATPNLEHFLFSQNAMPALPEALFAHTPKLKTINLLNNKFESIPENIFFNLSNLTVLRLYGNNLKEINPKLFTNTPMVSKLELSFNGITNVTSDTFKYLPKLQHLFMKFNDIESLPPDLFHNCPELQSFHIHYNKLQSLPSELFSKSKNISDLDFDKNEIKEIPEELFHGQENLTILMMQQNVLKNIPDGAFKDLTNLEKLSLQNNPIKSLPPGSFDHQHKMKILNLANTSLTDLPDKIFKNCESLVEIDLSNNHLSELKSTVFPHPATALSILKLNRNSLSLSPRTSEPQARQILVEQFPFSDQVNLTYLFLQSNRIQNIPHALRNLKKLKHLDLKNNSIEYLDYYGFLFSSDTTADPSDAPWANLQELPQALPSQVIKIELKDNPLICDCSLYNFALWLQGKISEGDVQLNVVDKADVKCSMPDDKSVQKFVMTVDLSTLVCNREECPTSCTCITRPHDNMFFMECIQQRLQAIPPLKAYLPLGNYSVTLNLKNNFIASLEGLQSPEYSNVVNLTVAHNALKFINESYLPRRLKALDLSGNALIHSSKSLIAFLNVTNPTLSLSGNPWVCDCQLLDLYNFLRDPLRKMADSHPIMCDNSEPLLSLTEEQLCPTIQQPMVVVTIASTTVFLVLFFVLGTVSVYKYQQNIKVWLYTHQMCLWIIAKEEADKKKYDAFISYSNQDEEYVNNVLVPGLESGEPKYRVCLHYRDWVAGDYIQNQINQSIEDSHRTIVILSSSFIENVWGQIEFKTAHSKALKEKSKNIIVIVLGQSWLTAKEPQPDYSDLSARQGCSR

transcript_transcript/3576_Pt_Mix_transcript3576/f35p0/3715

ATGGCGGGTGAACATCAAGCCATGGCTGCCCTGTGGCTGTGGCTAGCCGTCCTGACGCTGGCCACCACACTCTCCTCTGCCTGCCCCAACTGCTTCAAAAATTTTAAGAGTACTTATTGCCCAATCCCATTTACACCTGAAGAACAGACGTACTCCTTAAAATTATCTAACTATAAAAATGCCGAAGAGAGTGCTCTGACCTACCAGTGTCACCACTTAGCTCCACAGATGTACTTCACCCACCACCAAGGATGCAATTTTTCCACAGTTCAATATGTGGCATTCCAGCGATGTCCGTTACCAAATGTCTCATTCAGTGAGATCTTTACTCAGCTTGGAGTACACCCAGAAAAGATTTTGGAAATGACTTTTAAAAATATTGGGACACGAAAAGATTTGGAACTTAAAACCTGGCACCTGGACGGACTTGTTAATCTTCAGATCTTACAATTAAAGCACAATTTGTTCACTTCATTACATCCAGACGTCCTTAAAGCAACTCCAAACCTGGAACACTTCCTTTTCTCACAAAATGCTATGCCTGCACTTCCTGAAGCTTTATTTGCACATACACCAAAGCTCAAAACAATTAATTTATTGAATAATAAATTTGAAAGCATACCTGAAAATATATTTTTTAATCTTTCAAATTTGACTGTCTTAAGATTGTATGGCAATAATCTAAAGGAGATCAACCCAAAGCTCTTTACTAATACACCTATGGTTTCCAAATTAGAGCTGTCATTTAATGGAATAACCAATGTGACTTCAGATACTTTTAAGTATCTTCCAAAACTACAACATTTATTCATGAAGTTTAATGATATTGAATCCTTACCACCAGACTTATTTCACAACTGCCCTGAACTGCAATCTTTCCATATACACTATAACAAACTTCAATCCCTTCCTTCAGAGCTTTTCAGTAAGTCAAAGAACATAAGTGATTTAGATTTTGATAAAAATGAAATAAAAGAGATTCCTGAAGAATTATTTCATGGGCAGGAGAACCTAACGATTCTAATGATGCAACAAAATGTTCTCAAGAATATTCCTGATGGTGCCTTTAAAGATCTAACAAACCTTGAGAAACTGTCACTGCAAAATAACCCTATCAAGAGTCTGCCACCTGGGAGCTTTGACCACCAGCACAAAATGAAGATACTTAACCTGGCAAACACCAGTCTTACTGATCTGCCTGACAAGATTTTCAAAAACTGTGAATCTTTAGTGGAAATTGACCTTTCAAATAATCATTTGAGTGAATTGAAGAGTACTGTTTTTCCTCATCCTGCTACAGCTCTAAGCATCCTAAAACTCAATAGAAATAGTCTTTCTCTCTCACCCAGAACAAGCGAGCCACAAGCACGGCAGATTCTGGTGGAACAGTTTCCTTTCTCAGACCAAGTCAACTTAACTTATCTTTTTCTACAGAGCAACAGAATCCAAAATATACCTCATGCTCTGAGAAACCTGAAGAAGTTGAAGCATCTGGATTTGAAGAATAATAGCATTGAATACTTAGATTATTATGGATTTTTATTTAGTTCTGATACTACTGCTGATCCAAGTGATGCTCCTTGGGCAAACCTACAAGAACTGCCACAAGCTTTACCCTCACAAGTCATTAAAATTGAATTAAAAGATAATCCTTTGATTTGTGACTGCAGTCTTTATAACTTTGCTCTGTGGTTGCAAGGCAAGATCTCTGAAGGTGATGTCCAGCTGAATGTTGTTGATAAAGCTGATGTCAAATGCTCTATGCCAGATGACAAAAGTGTCCAGAAATTTGTTATGACTGTAGACTTGAGCACACTTGTCTGTAACAGAGAGGAATGCCCTACATCCTGCACCTGTATTACAAGGCCCCATGACAATATGTTCTTTATGGAATGTATTCAGCAAAGATTACAGGCAATTCCTCCACTAAAAGCTTATTTACCACTAGGTAACTATTCAGTCACCCTTAACTTGAAGAACAACTTTATCGCCAGTCTGGAAGGTCTTCAGAGTCCTGAGTACTCCAATGTAGTGAATCTGACAGTGGCTCACAATGCCCTCAAATTCATCAATGAAAGTTACTTGCCAAGAAGACTAAAGGCTTTGGATCTTAGTGGCAATGCTCTCATTCATTCCTCTAAATCTCTTATAGCCTTTCTGAATGTAACCAATCCCACCTTGAGCCTTAGTGGGAATCCATGGGTCTGTGATTGCCAGCTGTTGGACCTCTACAACTTCTTGAGGGATCCCCTCAGAAAGATGGCTGATTCACATCCCATCATGTGTGACAATTCTGAGCCTCTCCTGAGCCTGACAGAGGAGCAGCTCTGCCCGACCATCCAGCAGCCCATGGTGGTTGTCACTATTGCTTCCACCACTGTCTTCTTGGTTTTGTTCTTTGTTTTAGGAACTGTAAGTGTGTACAAGTATCAACAAAACATCAAGGTATGGCTCTATACTCATCAAATGTGTCTGTGGATCATAGCCAAAGAAGAAGCAGACAAGAAAAAATATGATGCCTTCATCAGCTACTCTAACCAAGATGAGGAATATGTCAACAATGTGTTGGTTCCCGGGCTGGAGAGTGGAGAGCCTAAGTACCGCGTGTGCCTCCACTACCGAGACTGGGTGGCTGGAGATTATATCCAGAACCAGATTAACCAAAGCATTGAGGACAGCCACAGGACTATCGTTATACTGTCTTCCAGCTTCATAGAAAATGTTTGGGGACAGATTGAATTTAAAACTGCCCATTCTAAAGCACTTAAAGAAAAAAGCAAGAACATTATTGTCATTGTTTTGGGACAGGTGCCTCCAGCATCAGAGATGGATGAGGAACTCAAGCTGTACCTCTCTACCAGAACATACCTACAATCAGATCATCCCAGGTTTTGGGAGAACCTACGATATGCCATGCCACACCCACAGGAATTCTTATACAAGAAACGAAGAAAGACCAAGAAAATTGAGGGGCTACAAATGGTGCAAGCAAATGGTAAAGCAGGATCATAA

MAGEHQAMAALWLWLAVLTLATTLSSACPNCFKNFKSTYCPIPFTPEEQTYSLKLSNYKNAEESALTYQCHHLAPQMYFTHHQGCNFSTVQYVAFQRCPLPNVSFSEIFTQLGVHPEKILEMTFKNIGTRKDLELKTWHLDGLVNLQILQLKHNLFTSLHPDVLKATPNLEHFLFSQNAMPALPEALFAHTPKLKTINLLNNKFESIPENIFFNLSNLTVLRLYGNNLKEINPKLFTNTPMVSKLELSFNGITNVTSDTFKYLPKLQHLFMKFNDIESLPPDLFHNCPELQSFHIHYNKLQSLPSELFSKSKNISDLDFDKNEIKEIPEELFHGQENLTILMMQQNVLKNIPDGAFKDLTNLEKLSLQNNPIKSLPPGSFDHQHKMKILNLANTSLTDLPDKIFKNCESLVEIDLSNNHLSELKSTVFPHPATALSILKLNRNSLSLSPRTSEPQARQILVEQFPFSDQVNLTYLFLQSNRIQNIPHALRNLKKLKHLDLKNNSIEYLDYYGFLFSSDTTADPSDAPWANLQELPQALPSQVIKIELKDNPLICDCSLYNFALWLQGKISEGDVQLNVVDKADVKCSMPDDKSVQKFVMTVDLSTLVCNREECPTSCTCITRPHDNMFFMECIQQRLQAIPPLKAYLPLGNYSVTLNLKNNFIASLEGLQSPEYSNVVNLTVAHNALKFINESYLPRRLKALDLSGNALIHSSKSLIAFLNVTNPTLSLSGNPWVCDCQLLDLYNFLRDPLRKMADSHPIMCDNSEPLLSLTEEQLCPTIQQPMVVVTIASTTVFLVLFFVLGTVSVYKYQQNIKVWLYTHQMCLWIIAKEEADKKKYDAFISYSNQDEEYVNNVLVPGLESGEPKYRVCLHYRDWVAGDYIQNQINQSIEDSHRTIVILSSSFIENVWGQIEFKTAHSKALKEKSKNIIVIVLGQVPPASEMDEELKLYLSTRTYLQSDHPRFWENLRYAMPHPQEFLYKKRRKTKKIEGLQMVQANGKAGS

transcript_transcript/4908_Pt_Mix_transcript4908/f3p0/3431

ATGGCGGGTGAACATCAAGCCATGGCTGCCCTGTGGCTGTGGCTAGCCGTCCTGACGCTGGCCACCACACTCTCCTCTGCCTGCCCCAACTGCTTCAAAAATTTTAAGAGTACTTATTGCCCAATCCCATTTACACCTGAAGAACAGACGTACTCCTTAAAATTATCTAACTATAAAAATGCCGAAGAGAGTGCTCTGACCTACCAGTGTCACCACTTAGCTCCACAGATGTACTTCACCCACCACCAAGGATGCAATTTTTCCACAGTTCAATATGTGGCATTCCAGCGATGTCCGTTACCAAATGTCTCATTCAGTGAGATCTTTACTCAGCTTGGAGTACACCCAGAAAAGATTTTGGAAATGACTTTTAAAAATATTGGGACACGAAAAGATTTGGAACTTAAAACCTGGCACCTGGACGGACTTGTTAATCTTCAGATCTTACAATTAAAGCACAATTTGTTCACTTCATTACATCCAGACGTCCTTAAAGCAACTCCAAACCTGGAACACTTCCTTTTCTCACAAAATGCTATGCCTGCACTTCCTGAAGCTTTATTTGCACATACACCAAAGCTCAAAACAATTAATTTATTGAATAATAAATTTGAAAGCATACCTGAAAATATATTTTTTAATCTTTCAAATTTGACTGTCTTAAGATTGTATGGCAATAATCTAAAGGAGATCAACCCAAAGCTCTTTACTAATACACCTATGGTTTCCAAATTAGAGCTGTCATTTAATGGAATAACCAATGTGACTTCAGATACTTTTAAGTATCTTCCAAAACTACAACATTTATTCATGAAGTTTAATGATATTGAATCCTTACCACCAGACTTATTTCACAACTGCCCTGAACTGCAATCTTTCCATATACACTATAACAAACTTCAATCCCTTCCTTCAGAGCTTTTCAGTAAGTCAAAGAACATAAGTGATTTAGATTTTGATAAAAATGAAATAAAAGAGATTCCTGAAGAATTATTTCATGGGCAGGAGAACCTAACGATTCTAATGATGCAACAAAATGTTCTCAAGAATATTCCTGATGGTGCCTTTAAAGATCTAACAAACCTTGAGAAACTGTCACTGCAAAATAACCCTATCAAGAGTCTGCCACCTGGGAGCTTTGACCACCAGCACAAAATGAAGATACTTAACCTGGCAAACACCAGTCTTACTGATCTGCCTGACAAGATTTTCAAAAACTGTGAATCTTTAGTGGAAATTGACCTTTCAAATAATCATTTGAGTGAATTGAAGAGTACTGTTTTTCCTCATCCTGCTACAGCTCTAAGCATCCTAAAACTCAATAGAAATAGTCTTTCTCTCTCACCCAGAACAAGCGAGCCACAAGCACGGCAGATTCTGGTGGAACAGTTTCCTTTCTCAGACCAAGTCAACTTAACTTATCTTTTTCTACAGAGCAACAGAATCCAAAATATACCTCATGCTCTGAGAAACCTGAAGAAGTTGAAGCATCTGGATTTGAAGAATAATAGCATTGAATACTTAGATTATTATGGATTTTTATTTAGTTCTGATACTACTGCTGATCCAAGTGATGCTCCTTGGGCAAACCTACAAGAACTGCCACAAGCTTTACCCTCACAAGTCATTAAAATTGAATTAAAAGATAATCCTTTGATTTGTGACTGCAGTCTTTATAACTTTGCTCTGTGGTTGCAAGGCAAGATCTCTGAAGGTGATGTCCAGCTGAATGTTGTTGATAAAGCTGATGTCAAATGCTCTATGCCAGATGACAAAAGTGTCCAGAAATTTGTTATGACTGTAGACTTGAGCACACTTGTCTGTAACAGAGAGGAATGCCCTACATCCTGCACCTGTATTACAAGGCCCCATGACAATATGTTCTTTATGGAATGTATTCAGCAAAGATTACAGGCAATTCCTCCACTAAAAGCTTATTTACCACTAGGTAACTATTCAGTCACCCTTAACTTGAAGAACAACTTTATCGCCAGTCTGGAAGGTCTTCAGAGTCCTGAGTACTCCAATGTAGTGAATCTGACAGTGGCTCACAATGCCCTCAAATTCATCAATGAAAGTTACTTGCCAAGAAGACTAAAGGCTTTGGATCTTAGTGGCAATGCTCTCATTCATTCCTCTAAATCTCTTATAGCCTTTCTGAATGTAACCAATCCCACCTTGAGCCTTAGTGGGAATCCATGGGTCTGTGATTGCCAGCTGTTGGACCTCTACAACTTCTTGAGGGATCCCCTCAGAAAGATGGCTGATTCACATCCCATCATGTGTGACAATTCTGAGCCTCTCCTGAGCCTGACAGAGGAGCAGCTCTGCCCGACCATCCAGCAGCCCATGGTGGTTGTCACTATTGCTTCCACCACTGTCTTCTTGGTTTTGTTCTTTGTTTTAGGAACTGTAAGTGTGTACAAGTATCAACAAAACATCAAGGTATGGCTCTATACTCATCAAATGTGTCTGTGGATCATAGCCAAAGAAGAAGCAGACAAGAAAAAATATGATGCCTTCATCAGCTACTCTAACCAAGATGAGGAATATGTCAACAATGTGTTGGTTCCCGGGCTGGAGAGTGGAGAGCCTAAGTACCGCGTGTGCCTCCACTACCGAGACTGGGTGGCTGGAGATTATATCCAGAACCAGATTAACCAAAGCATTGAGGACAGCCACAGGACTATCGTTATACTGTCTTCCAGCTTCATAGAAAATGTTTGGGGACAGATTGAATTTAAAACTGCCCATTCTAAAGCACTTAAAGAAAAAAGCAAGAACATTATTGTCATTGTTTTGGGACAGGTGAGTATAAATCCTTATTATTTTATAAGAAATTTTTATATTATTTATGTTAATATTTTTCATGGTTTCTGGAGCCTAAGTATTCCTAGTCATTTGCAAATTATTATTATTATCTATGCTGTATTCCTAAGAATTCTAAAATACTTTATAATTGTTTGTAAGGGTTCTTCTTTAAATAAACAATAA

MAGEHQAMAALWLWLAVLTLATTLSSACPNCFKNFKSTYCPIPFTPEEQTYSLKLSNYKNAEESALTYQCHHLAPQMYFTHHQGCNFSTVQYVAFQRCPLPNVSFSEIFTQLGVHPEKILEMTFKNIGTRKDLELKTWHLDGLVNLQILQLKHNLFTSLHPDVLKATPNLEHFLFSQNAMPALPEALFAHTPKLKTINLLNNKFESIPENIFFNLSNLTVLRLYGNNLKEINPKLFTNTPMVSKLELSFNGITNVTSDTFKYLPKLQHLFMKFNDIESLPPDLFHNCPELQSFHIHYNKLQSLPSELFSKSKNISDLDFDKNEIKEIPEELFHGQENLTILMMQQNVLKNIPDGAFKDLTNLEKLSLQNNPIKSLPPGSFDHQHKMKILNLANTSLTDLPDKIFKNCESLVEIDLSNNHLSELKSTVFPHPATALSILKLNRNSLSLSPRTSEPQARQILVEQFPFSDQVNLTYLFLQSNRIQNIPHALRNLKKLKHLDLKNNSIEYLDYYGFLFSSDTTADPSDAPWANLQELPQALPSQVIKIELKDNPLICDCSLYNFALWLQGKISEGDVQLNVVDKADVKCSMPDDKSVQKFVMTVDLSTLVCNREECPTSCTCITRPHDNMFFMECIQQRLQAIPPLKAYLPLGNYSVTLNLKNNFIASLEGLQSPEYSNVVNLTVAHNALKFINESYLPRRLKALDLSGNALIHSSKSLIAFLNVTNPTLSLSGNPWVCDCQLLDLYNFLRDPLRKMADSHPIMCDNSEPLLSLTEEQLCPTIQQPMVVVTIASTTVFLVLFFVLGTVSVYKYQQNIKVWLYTHQMCLWIIAKEEADKKKYDAFISYSNQDEEYVNNVLVPGLESGEPKYRVCLHYRDWVAGDYIQNQINQSIEDSHRTIVILSSSFIENVWGQIEFKTAHSKALKEKSKNIIVIVLGQVSINPYYFIRNFYIIYVNIFHGFWSLSIPSHLQIIIIIYAVFLRILKYFIIVCKGSSLNKQ

transcript_transcript/12177_Pt_Mix_transcript12177/f2p0/2526

ATGCACAGATCATGTTGCTTAGTGTTCCTTGGTGTGGTGGGTGTGCTGCTTATTGTGTTTGGAGTGTTCATTACTGTGATGTTCGACCCTATGGTCAACGCCTTCATTTTCAAGGAACTTGTTATTCAGAATAATACCGATGTCTACAAAAACTGGAAGGATCCGCCCATTGTGCCTCACTTGAAAATATATTTCTTTAATGTTACCAATAAGGAGGAGTTTTTAGAGGGGAGTAAGCCAGTGCTCCAGGAGGTGGGGCCATACTGCTACAGAGAACACTGGAGGAAAGTAAACATATCATTTTATGACAATGGTACTGTTTCCTATGAGACCCAAAAGTTTTATTTCTTTGAGAGAAGTGCTTCTGTTGGATCAGAGGATGATCTCATCACCACCCTCAACATTCCAATGATGACTGCTGTGTCTCAGTGGAGATTTGCAGCCAGACTTGCTAAGCTGGCACTCTCATCCATGCTGGAGGTGTTGCGCACCAAGCCATTTGTGACGAAAACTGTTGGGCAGCTCATGTGGGGCTATGACGACCCGTTGCTCAAGATTGCCAAGGACATAATTCCCCCTGATAAACGTTTGCCACATGAACAGTTCGGGTTTTTCATTGAGAAAAACGGATCAACAGACGGATTACTCAATGTGTACACTGGTAGAGATGACATGACAAAGTACATGACAATCAACACCTTTGACTACAAGTGGGAGTTGCATTACTGGAAGACAGATGAGTGTAACAAAATTCACGGCACTGATGGCTCTTCCTTCCCTCCGGGTGTCAGGAAAGACTCAGTCCTCTACATGTTCAATGAAAATCTGTGCCGTGCAATTCCTCTCACTTACTGGCATGATGTGATGGACTTTGGTCTTGAGGGAATGAGGTTCTCTCCTCCACCCGATATCTTTGCCAATGTGTCTGTCAAGCCCGAGAATGACTGTTACTGTGCAGGTGGTCCCCCTTGCATTGGTGGAGGTGTATTCAATATTAGTTCATGTAAATTTGGTTCCCCAACATTAGTCTCCTGGCCACACTTCTATCAAGGAGATGCCAAATACCGTGACGCCCTTATTGGCATGAACCCTGACCCTGAGAAACATGCCATGTATATTGATATTGCTCCTCGTACCGGAACACCTTTGAGGGCCCAGGCTCGGCTCCAGATCAACATTGCTGTCCCACATGTCCCAGAAGTGTATCCTGCTGCCAAGCTCAGAGAAATGATCTTCCCAGTGATGTGGTTTGAAGATGGTGTGACAGAGCTTCCCGGTGATCTACTGGATTTGCTGAGGCTTGGTGAAAATACCCCACCGGTGGCCAAAATGAGTATGCTTTTAGCAGCATTCACCATTGGATTCACAACTGTCCTATTCGTGGTGGTTATATTCTTTGCCACTCACTACAACTTGCCCGTTCCTTACTTCAACCCGAACTACACTCACAAGGACGGCAAAACAACAAAGATCAGCAATGCACAACCTCAGCCACACAGACCAGAGTTGTCAGGTCATGTCAACACTATGGTGTCCAATAACCCAGAGAATGATGACGCCTGA

MHRSCCLVFLGVVGVLLIVFGVFITVMFDPMVNAFIFKELVIQNNTDVYKNWKDPPIVPHLKIYFFNVTNKEEFLEGSKPVLQEVGPYCYREHWRKVNISFYDNGTVSYETQKFYFFERSASVGSEDDLITTLNIPMMTAVSQWRFAARLAKLALSSMLEVLRTKPFVTKTVGQLMWGYDDPLLKIAKDIIPPDKRLPHEQFGFFIEKNGSTDGLLNVYTGRDDMTKYMTINTFDYKWELHYWKTDECNKIHGTDGSSFPPGVRKDSVLYMFNENLCRAIPLTYWHDVMDFGLEGMRFSPPPDIFANVSVKPENDCYCAGGPPCIGGGVFNISSCKFGSPTLVSWPHFYQGDAKYRDALIGMNPDPEKHAMYIDIAPRTGTPLRAQARLQINIAVPHVPEVYPAAKLREMIFPVMWFEDGVTELPGDLLDLLRLGENTPPVAKMSMLLAAFTIGFTTVLFVVVIFFATHYNLPVPYFNPNYTHKDGKTTKISNAQPQPHRPELSGHVNTMVSNNPENDDA

transcript_transcript/15196_Pt_Mix_transcript15196/f2p0/2254

ATGCAGCTGAAGGCTCAGGTGTGCGTGCTGGTGGTGGTGGGCGTGGTGCTAGTGGTGGGCAGCCTGGTGTTCCATCACTTCTTCCAGCGGGTGTTCGGCAGCATGCTGGAGCAGCGCCTCATGTTGGTGCCGGGCTCCAAGACGCTGGACAACTTCAAGGCGCCGCCCGTGCCCATCTTCATGCAGTTCTGGCTGTTCAATGTGACAAATCCCGAGGCCGTGATGATGGAGGGCGCCACGCCCGTGCTGCAGCAGGTGGGGCCTTACACGTACGAGGAGAAGCAGCTCAAGTACAACCTCACTTTCAATGACGAGGAGGGCACGGTCACCTACCTGCAGAACAAGACCTTCTTCTTCCGCCCCGAGCTGTCCCCCGGCGTGTCGCTCGCTGACCGCGTCACCACCGTCAACGCCGTCATGATCGGCCTAGGCAACCTGCTGGCCTCGCGCCCCGCACACTTCGCTGCCGTCGCGCAGCTCTGGTTCCACAGGTTCGGCGTGACGCCCCTCGTCACCAAGACGGTGCGCGAGCTGCTGTTTGACGGATACGAGGAGCCGCTGTTGCTGCAGCTGGCGGACCTGACGGGCAAGCCGGAGCACGCCACGGGCAAGTTTGGCTTCTATAGGAAGAACAACACCAACGACGGCACGTACACGGTGTACACTGGCGTGAAGGGCATGGACCGCTACCAGCACATCGCACGCTGGCGCGGCAAGTCCACCATTGACTTCTGGGAAAATGACACGGCTGGCGACAACACGTGCAACATGATCAACGGCACGGCGGGGTCACAGTTCCCGCGCCCCGTGACACGCCACACGCTGCTGCGCCTGTACGTGGCCGAGCTGTGCCGCTCCATCTACGCCGAGTACCAGCGCGACGTGCGACACGGCGCCCTCACGCTGTACCGCTTCGTGCTGCCGCCGCGCCTGCTGGCCAACTCGCCCGAGAACCAGTGCTACTGCCACGACCCCTTCACGTGCCGCGCCTCCATGATCAATGTAGCGCCGTGCAGGAAGGGCGCGCCCGTTGTTATGTCCACGCCACACTTCTACCAGGGCAGCGCGGAGGACGTGGCGGAGCTGCAGGGCCTGGCGCCGCACGCACTAGAGCACGAGACCTACCTGGACGTGGAGCCCAACACGGGCGTCACCTTCCGGGCAGCCAAGCGCATCCAGGTAAACCTCCCGCTGCGCCAGTACGCCGGCATACCCGCCTTCCGCAACGTGCCGGACGTGGTGATGCCCGTGTTTTGGGTCAACGAGAGCGCTGTGGTGCCCCTGGAGCGCACACACGCCCTTCACCGAACCCTCACGCTGCCTTTCACGCTGGTGACGGTGGGCGTGGGCGTACTGGTCACCCTGGGCGTGGTGCTGATGCTGGTGGCCGCCATCAAGGTGTGCCTGGCAGTGAGGGCGGCGAGCGGCGGCAAGAACAGGCGCCCACAGAGAGACAAGGACAAGACCGTCACTGAAAAACTCAACAGCAGGAACTACAGCTGA

MQLKAQVCVLVVVGVVLVVGSLVFHHFFQRVFGSMLEQRLMLVPGSKTLDNFKAPPVPIFMQFWLFNVTNPEAVMMEGATPVLQQVGPYTYEEKQLKYNLTFNDEEGTVTYLQNKTFFFRPELSPGVSLADRVTTVNAVMIGLGNLLASRPAHFAAVAQLWFHRFGVTPLVTKTVRELLFDGYEEPLLLQLADLTGKPEHATGKFGFYRKNNTNDGTYTVYTGVKGMDRYQHIARWRGKSTIDFWENDTAGDNTCNMINGTAGSQFPRPVTRHTLLRLYVAELCRSIYAEYQRDVRHGALTLYRFVLPPRLLANSPENQCYCHDPFTCRASMINVAPCRKGAPVVMSTPHFYQGSAEDVAELQGLAPHALEHETYLDVEPNTGVTFRAAKRIQVNLPLRQYAGIPAFRNVPDVVMPVFWVNESAVVPLERTHALHRTLTLPFTLVTVGVGVLVTLGVVLMLVAAIKVCLAVRAASGGKNRRPQRDKDKTVTEKLNSRNYS

transcript_transcript/7832_Pt_Mix_transcript7832/f4p0/2974

ATGGACGTGGAACCGCTGCTCAGGGGCGCCTCTTCAACCTCTGATTACGGAACTAACGACGGAAAGCGGAATGGTGGCTCCAGGAATGCCGCCGACGAGGATGACGAAGCGACTACCGTGGTGACTCCTGCAAGATGGTACCTGACTGGCTGCCAGATAGCGGTGTTGGTGCTGGCGGTGCTGGCGATTGTGCTGAGTGTGGCTATGCTTGCTGGGGGCTACAAGGCAATCCTCAACAATGCCCTTCATGAGCAACTGGTCATCAAGAAAGGCTCAAAGGCATACGATATGTGGAAGGCAACTCCTGTTCCTCTGTCCCTGAAACTTTACGTCTTCAACCTGACCAACCCTGAAGAATTCAAGAACGGGTCCAAGCCGGTGCTGGATGAGCTGGGCCCATATGTCTGGAGAGAGTACCACAAGAAGCAAAACGTGACCTTCCACCCCAACGACACCGTGACTTACCTGCAGCAGCGGTGGTGGGTGTGGGACCAGCATGCTTCGGGGAACCGCTCCCAGGACGACCTCATCATCACCCTCAACACCATCCCTGTGGCTGCAGCGTATGGCTTGAGGAACTCAATCCCCTTTCAGGCCTTCCTTGACTTGGGCTTGAAGAGTGTGGGTGAGCAGCTGACGGTCAGCACCACTGCCAGGAAGCTTGTGTTTGATGGAGTGAAGGACCCTCTGCTTGACTGGGTACAGAAAAACGTGGTGAACAAGAAAGGAAAGTACCACATACTTTATCCGTTCTTCGAGGGAACCGCAGTGGCTGAGTTTGAAAAGTTTGCCTGGTTTTTTAAGCGTAACCTGTCCCTAACGTATGACGGAGAGTTCAACATGATGACTGGGGAAGACACGCTGGATAATCTGGGGAGGATTGACAACTGGAACGGCCGTAACAGCACCAGCTTCTACACGCCGCCCTGCAATGAAGTGACGGGCTCAGCTGGGGAACTCTTCTCCCCTCACCTCAGCCGAAAGGATCTCATTTTCTTTAGCTCTGATCTCTGCATGAGTGCCAAACTGTTCTTCAAGGAGGAGGTGAATGACTACGGGGGCCTGACTACCTACCGGTACTGGGGCACCAACCACACCTTCGCTAATGGCACTACTGTCCCTGGCAATGAGTGCTACTGTGTTGGCAAGACATGTGCCCCGATGGGTCTCCTCAATGCCGAGTCTTGCCGCATGGGTGCCCCGGCGTTTGTATCCTTCCCTCACTTCTATGCCGCTGATCCCTTCCTTCTCAATGGTGTGGAGGGCCTGGCACCTGACGAGAAGAAACATTCCCTCTTCCTGGATGTTGTGCCAAACTTGGGTGTGCCTGCTGATGTATCCATCAAGCTACAAATCAACATCCACGTGACACCTTTCCCAAGTATCAGCCTGCTGGAACGTGTCCCTGACGTGTACCTGCCTATGCTGTGGTTTGAGGTGCAGGGCGGCATGACACCAAGCATTGCCTCTCAGATGAAGATGCTTGTCTACTTCATGAACTCCAGTGTGCCCAGTGTGGTGGTGTGGTCGGTGATCATCTGCCTGGCAGTGTTGGCGATGGTGGTGGTGCTGCTGGTCGCCTGGAGAAGGAATAGTAGCTATGACCTGGTGACCTAG

MDVEPLLRGASSTSDYGTNDGKRNGGSRNAADEDDEATTVVTPARWYLTGCQIAVLVLAVLAIVLSVAMLAGGYKAILNNALHEQLVIKKGSKAYDMWKATPVPLSLKLYVFNLTNPEEFKNGSKPVLDELGPYVWREYHKKQNVTFHPNDTVTYLQQRWWVWDQHASGNRSQDDLIITLNTIPVAAAYGLRNSIPFQAFLDLGLKSVGEQLTVSTTARKLVFDGVKDPLLDWVQKNVVNKKGKYHILYPFFEGTAVAEFEKFAWFFKRNLSLTYDGEFNMMTGEDTLDNLGRIDNWNGRNSTSFYTPPCNEVTGSAGELFSPHLSRKDLIFFSSDLCMSAKLFFKEEVNDYGGLTTYRYWGTNHTFANGTTVPGNECYCVGKTCAPMGLLNAESCRMGAPAFVSFPHFYAADPFLLNGVEGLAPDEKKHSLFLDVVPNLGVPADVSIKLQINIHVTPFPSISLLERVPDVYLPMLWFEVQGGMTPSIASQMKMLVYFMNSSVPSVVVWSVIICLAVLAMVVVLLVAWRRNSSYDLVT

transcript_transcript/20067_Pt_Mix_transcript20067/f3p0/1897

ATGTCATATCTCCTCCTCCCCTGCTGTGACAGACTGTGGCACAACGACTCGGTGGACTTCATGGGCCACATTTGCTACTTCCGCCGCCGCCCTAAGATCAGAAGATTTAAGCTGTACCACGAGGGCAAGTTTTGGTGTCCTGGTTGGGCGCCTTTCGAGGGCAGGTGTAAGTATTGTGTCGTCTTCTGA

MSYLLLPCCDRLWHNDSVDFMGHICYFRRRPKIRRFKLYHEGKFWCPGWAPFEGRCKYCVVF

transcript_transcript/21439_Pt_Mix_transcript21439/f3p0/1809

ATGCGGAAAGGAGTGGTGGCCGGCCTGTGCCTGGCACTGGTGGTGATGTGCCTGTACCTGCCCCAGCCTTGCGAGGCTCAGTATGAGGCTCTGGTAACTTCCATTCTTGGAAAACTCACTGGACTGTGGCACAACGACTCGGTGGACTTCATGGGCCACATTTGCTACTTCCGCCGCCGCCCTAAGATCAGAAGATTTAAGCTGTACCACGAGGGCAAGTTTTGGTGTCCTGGTTGGGCGCCTTTCGAGGGCAGGTGTAAGTATTGTGTCGTCTTCTGA

MRKGVVAGLCLALVVMCLYLPQPCEAQYEALVTSILGKLTGLWHNDSVDFMGHICYFRRRPKIRRFKLYHEGKFWCPGWAPFEGRCKYCVVF

transcript_transcript/30671_Pt_Mix_transcript30671/f4p0/1177

ATGGGCCACATTTGCTACTTCCGCCGCCGCCCTAAGATCAGAAGATTTAAGCTGTACCACGAGGGCAAGGTTTTGGTGTCCTGGTTGGGCGCCTTTCGAGGGCAGGTCGAGGACAAAGAGCAGGTCGGGGTCATCCAGGGAGGCCACCAAAGGACTTCGTGCGCAAAGCTTTACAGAACGGACTCGTCACACAGCAGGATGCTTCTCTGTGGCTGA

MGHICYFRRRPKIRRFKLYHEGKVLVSWLGAFRGQVEDKEQVGVIQGGHQRTSCAKLYRTDSSHSRMLLCG

transcript_transcript/31938_Pt_Mix_transcript31938/f3p0/1033

ATGGGCCACATTTGCTACTTCCGCCGCCGCCCTAAGATCAGAAGATTTAAGCTGTACCACGAAGGGCAAGTTTTGGTGTCCTGGTTGGGCGCCTTTCGAGGGCAGGTCGAGGACAAAGAGCAGGTCGGGGTCATCCAGGGAGGGCCACCAAGGACTTCGTGCGCAAAGCTTTACAGAACGGACTCGTCACACAGCAGGATGCTTCTCTGTGGCTGA

MGHICYFRRRPKIRRFKLYHEGQVLVSWLGAFRGQVEDKEQVGVIQGGPPRTSCAKLYRTDSSHSRMLLCG

transcript_transcript/35525_Pt_Mix_transcript35525/f2p0/756

ATGTGTGGTGCCCCGGCTGGACAGTCCATCAAGGGAGAATCCCTGACTCGCAGCAGGACTAGGGTGGTGAACAAGGCCGTCGCAGACTTCGCCCAGAAAGCTCTCGCCTCAGGGCCTCATCACGCAGGAGGACGCCCAACCCTTGCTAGAGTAGCTTGGACCTACAGGCGTGTGTGCCAAAGAGGTGCCAAAGACGGAATGATCTCTTCACTATTCATACTCCCGTGA

MCGAPAGQSIKGESLTRSRTRVVNKAVADFAQKALASGPHHAGGRPTLARVAWTYRRVCQRGAKDGMISSLFILP

transcript_transcript/35689_Pt_Mix_transcript35689/f2476p0/580

ATGTGTGGTGCCCCGGCTGGACAGTCATCAAGGGAGAATCCCCTGACTCGCAGCAGGACTAGGGTGGTGAACAAGGCCGTCGCAGACTTCGCCCAGAAAGCTCTCGCTCAGGGCCTCATCACGGCAGGAGGACGCCCAACCCTTGCTAGAGTAGCTTGGACCTACAGGCGTGTGTGCCAAAGAGGTGCAAAGACGGAATGA

MCGAPAGQSSRENPLTRSRTRVVNKAVADFAQKALAQGLITAGGRPTLARVAWTYRRVCQRGAKTE

transcript_transcript/35792_Pt_Mix_transcript35792/f2p0/720

ATGAGGAACGGAACCACTGAGATCCTTGACCGTGTATGCAACTACCGAGTAGTACCACGCTTAGAGAGCTTAGAGCTTTACTTTAAAGGGCGACGTGTGGTGCCCCGGCTGGACAGTCATCAAGGGAGAATCCCTGACTCGCAGCAGGACTAG

MRNGTTEILDRVCNYRVVPRLESLELYFKGRRVVPRLDSHQGRIPDSQQD

transcript_transcript/24866_Pt_Mix_transcript24866/f982p0/1465

ATGTCCTTCTCCTCTTGGCCTGCCTCGCGGCTGTGGGCAATGCTTTACAACGCCAAGGTACCAGTCCAGGTCCTGCCAGAGAGATTCGATACCTTCCCGGGAAGAAATCCATCCTTGACCAGACCAAGCAGTAGTGGGAGTTCAGACGCTCCCAGGAAGAGTGCCACCTCAGACTTTACCGGGAGTAGTTGGAGTCAGTCCCTTGGTTCTCCCCGGGCCGCCCAGGCATCACAGGCTCCGTCAGGCCCTTCCAACGTCCAGGCCAATACTCGTTCACCCGCTACAACTTGCAGGAGACAATGCCCGGGGTACGAAAGCGGTAATGCGAGGTGCTGCAGGCTGTCTGGTGACTGTTGCGGCAATTCCTATTCCAATCCCTTACAAAGGCTAGAGTGA

MSFSSWPASRLWAMLYNAKVPVQVLPERFDTFPGRNPSLTRPSSSGSSDAPRKSATSDFTGSSWSQSLGSPRAAQASQAPSGPSNVQANTRSPATTCRRQCPGYESGNARCCRLSGDCCGNSYSNPLQRLE

transcript_transcript/27530_Pt_Mix_transcript27530/f2p0/1425

ATGTCCTTCTCCTCTTGGCCTGCCTCGCGGCTGTGGGCAATGCTTTACAACGCCAAGGTACCAGTCCAGGTCCTGCCAGAGAGATTCGATACCTTCCCAGGAAGAAATCCATCCTTGACCAGACCCAGCAGTAGTGGGAGTTCAGACGCTCCCAGGAAGAGTGCCACCTCAGCCTTTACCGGGAGTAGTTGGAGTCAGTCCCTTGGGTCTCCCCGGGCCGCCCAGGCATCACAGGCTCCGGGTACGAAAGCCGTAATGCGAGGTGCTGCAGGCTGTCTGGTGACTGTTTGCGGCAATTCCTATCCAATCCCTTACAAAGGCTAG

MSFSSWPASRLWAMLYNAKVPVQVLPERFDTFPGRNPSLTRPSSSGSSDAPRKSATSAFTGSSWSQSLGSPRAAQASQAPGTKAVMRGAAGCLVTVCGNSYPIPYKG

transcript_transcript/28185_Pt_Mix_transcript28185/f79p0/1336

ATGTCCTTCTCCTCTTGGCCTGCCTCGCGGCTGTGGGCAATGCTTTACAACGCCAAGGTACCAGTCCAGGTCCTGCCAGAGAGATTCGATACCTTCCCGGGAAGAAATCCATCCTTGACCAGACCCAGCAGTAGTGGGAGTTCAGACGCTCCCAGGAAGAGTGCCACCTCAGACTTTACCGGGAGTAGTTGGAGTCAGTCCCTTGGGTCTCCCCGGGCCGCCCAGGCATCACAGGCTCCATCAGGCCCTTCCAACGTCCAGGCCAATACTCGTTCACCCGCTACAACCTGCAGGAGACAATGCCCGGGGTACGAAAGCGGTAATGCGAGGTGCTGCAGGCTGTCTGGTGACTGTTGCGGCAATTCCTAA

MSFSSWPASRLWAMLYNAKVPVQVLPERFDTFPGRNPSLTRPSSSGSSDAPRKSATSDFTGSSWSQSLGSPRAAQASQAPSGPSNVQANTRSPATTCRRQCPGYESGNARCCRLSGDCCGNS

transcript_transcript/32833_Pt_Mix_transcript32833/f2p0/1016

ATGTCCTTCTCCTCTTGGCCTGCCTCGCGGCTGTGGGCCAATGCTTACAACGCCAAGGTACCAGTCCAGGTCCTGCCAGAGAGATTCGATACCTTCCCGGGAAGAAATCCATCCTTGACCCAGACCAGCAGTAGTGGGAGTTCAGACGCTCCCAGGAAGAGTGCCACCTCAGACTTTACCGGGAGTAGTTGGAGTCAGTCCCCTTGGTCTCCCCGGGCCGCCCAGGCATCACAGGCTCCATCAGGCCCTTCCAACGTCCAGGCCAATACTCGTTCACCCGCCTACAACTGCAGGAGACAATGCCCGGGGTACGAAAGCGGTAATGCGAGGTGCTGCAGGCTGTCTGGTGACTGTTGCGGCAAATTCCTATCCAATCCCTTACAAAGGCTAGAGTGGACGCAACACCTTTCATACACCAAATGA

MSFSSWPASRLWANAYNAKVPVQVLPERFDTFPGRNPSLTQTSSSGSSDAPRKSATSDFTGSSWSQSPWSPRAAQASQAPSGPSNVQANTRSPAYNCRRQCPGYESGNARCCRLSGDCCGKFLSNPLQRLEWTQHLSYTK

transcript_transcript/33272_Pt_Mix_transcript33272/f3p0/963

ATGTCCTTCTCCTCTTGGCTTGCCTCGCGGCTGTGGGCAAATGTTTACAACGCCAAGGTACCAGTCAAGTTCCTGCCAGAGAGATTCGATACTTTCCCGGGAAGAAATCCATCCTTAACCAAGACCAGCAGTAGTGGGAGTTCAGACGCCCCCAGGAAGAGTGCCACCTCAGAATTTACCGGGAGTAGTTGGAGTCAATCCCCCCCCTTGGTCCCCTCCCCCCAGGGCCGCCCAGGCTTCACAGGCTCCGTCAGGCCCTTCTAA

MSFSSWLASRLWANVYNAKVPVKFLPERFDTFPGRNPSLTKTSSSGSSDAPRKSATSEFTGSSWSQSPPLVPSPQGRPGFTGSVRPF

transcript_transcript/33457_Pt_Mix_transcript33457/f2p0/932

ATGTCCTACTCCTCTTGGCCTGCCTCGCGGCTGTGGGCAAATGCTTACAACGCCAAGGTACCAGTCCAGGTCCTGCCAGAGAGATTCGATACCTTCCCGGGAAGAAATCCATCCTTGACCAAGACCAGCAGTAGTGGGAGTTCAGACGCTCCCAGGAAGAGTGCCACCTCAGACTTTACCGGGAGTAGTTGGAGTCAGTCCCCTTGGTCTCCCGGGCCGCCCGCCGCCCGTCCCAGCCATCACAGGCTCCATCAGGCCCTTCCAACTTCCAGGCCAATACTCGGTTCACCGCTACAACTGCAGGAGACAATGCCCGGGGTACGAAAGCGGTAA

MSYSSWPASRLWANAYNAKVPVQVLPERFDTFPGRNPSLTKTSSSGSSDAPRKSATSDFTGSSWSQSPWSPGPPAARPSHHRLHQALPTSRPILGSPLQLQETMPGVRKR

transcript_transcript/33937_Pt_Mix_transcript33937/f2p0/927

ATGTCCGTCTCCTCTTGGCCTGCCTCGCGGCTGTGGGCCATGCTTTACAACGCCAAGGTACCAGACCAGGTCCTGCCAGAGAGATTCGATACCTTCCCGGGAAGAAGTCCATCCTTGACCAGACCCAGCAGTAGTCGGAGTTCAGACGCTCCCAGGAAGAGTGCCACCTCAGACTTTACCAGGAGTAGTTGGAGTCAGTCCCTTGGGTCTCCCCGGGGCGCCCAGGCATCACAGGCTCCATCAGGCCCTTCCAACGTCCAGGCCAATACTCGTACACCCGCTACAACCTGCAGGAGAAAATGCCCGGGGTACGAAAGCGGAAATGCGAGGTGCTGCAGGCTGTATGGTGACTGTTGCGGCAATTCCAAATCCAATCCCGCACACCCATTGTTGTTGATTTGGAGAGCTATGACAATCTGCTTTCAAAAGGGCTTAGAGTGA

MSVSSWPASRLWAMLYNAKVPDQVLPERFDTFPGRSPSLTRPSSSRSSDAPRKSATSDFTRSSWSQSLGSPRGAQASQAPSGPSNVQANTRTPATTCRRKCPGYESGNARCCRLYGDCCGNSKSNPAHPLLLIWRAMTICFQKGLE

transcript_transcript/34254_Pt_Mix_transcript34254/f2p0/859

ATGTCCTTCTCCTCTTGGCCTGCCCTCGCGGCTGTGGGCAATGCTTACAACGCCAAGGTACCAGTCCAGGTCCTGTCAGAGAGATTCGATACCTTCCCGGAAGAAAATCCATCCTTGACCATACCAGCAGTAGTGGGAGTTCAGACGCTCCCAGGAAGAGTGCCACCTCAGACTTTACCGGGAGTAAGTTGGAGTCAGTCCCTTGGTCTCCCCGGGCCGCCCAGGCATCACAGGCTCCAGCAGGCCCTTCCAACGTCCAGGCCAATAACTCGTTCACCCGCTACAACTGCAGGAGACAATGCCCGGGGTACGAAAGCGGTAATGCGAGGTGCTGCAGGCTGTCTGGTGGACTGTTGCGGCAAATTCCTATCCAATCCCTTACAAAGGCTAGAGTGA

MSFSSWPALAAVGNAYNAKVPVQVLSERFDTFPEENPSLTIPAVVGVQTLPGRVPPQTLPGVSWSQSLGLPGPPRHHRLQQALPTSRPITRSPATTAGDNARGTKAVMRGAAGCLVDCCGKFLSNPLQRLE

transcript_transcript/34327_Pt_Mix_transcript34327/f14p0/821

ATGTCCTTCTCCTCTTGGCCTGCCTCGCGGCTGTGGGCAATGCTTTACAACGCCAAGGTACCAGTCCAGGTCCTGCCAGAGAGATTCGATACCTTCCCGGGAAGAAATCCATCCTTGACCAGACCAAGCAGTAGTGGGAGTTCAGACGCTCCCAGGAAGAGTGCCACCTCAGTCCCTTGGTCTCCCCGGGCCGCCCAGGCATCACAGGGCTCCATCAGGCCCTTCCAACGTCCAGGCCAATACTCGTTCACCCGCTACAACTGCAGGAGACAATGCCCGGGGTACGAAAAGCGGTAA

MSFSSWPASRLWAMLYNAKVPVQVLPERFDTFPGRNPSLTRPSSSGSSDAPRKSATSVPWSPRAAQASQGSIRPFQRPGQYSFTRYNCRRQCPGYEKR

transcript_transcript/34772_Pt_Mix_transcript34772/f29p0/777

ATGTCCTTCTCCTCTTGGCCTGCCTCGCGGCTGTGGGCAATGCTTTACAACGCCAAGGTACCAGTCCAGGTCCTGCCAGAGAGATTCGATACCTTCCCGGGAAGAAATCCATCCTTGACCAGACCCAGCAGTAGTGGGAGTTCAGACGCTCCCAGGAAGAGTGCCACCTCAGACTTTACCGGGAGTAGTTGGAGTCAGTCCCTTGGGTCTCCCCGGGCCGCCCAGGCATCACAGGCTCCGTCAGGCCCTTCCAACGTCCAGGCCAATACTCGTTCACCCGCTACAACCTGCAGGAGACAATGCCCGGGGTACGAAAGCGGTAATGCGAGGTGCTGCAGGCTGTCTGGTGACTGTTGCGGCAATTCCTAA

MSFSSWPASRLWAMLYNAKVPVQVLPERFDTFPGRNPSLTRPSSSGSSDAPRKSATSDFTGSSWSQSLGSPRAAQASQAPSGPSNVQANTRSPATTCRRQCPGYESGNARCCRLSGDCCGNS

transcript_transcript/35306_Pt_Mix_transcript35306/f8p0/745

ATGTCCTTCTCCTCTTGGCCTGCCTCGCGGCTGTGGGCAATGCTTTACAACGCCAAGGTACCAGTCCAGGTCCTGCCAGAGAGATTCGATACCTTCCCGGGAAGAAATCCATCCTTGACCAGACCAAGCAGTAGTGGGAGTTCAGACGCTCCCAGGAAGAGTGCCACCTCAGGCCAATACTCGTTCACCCGCTACAACTGCAGGAGAACAATGCCCGGGGTACGAAAGCGGTAA

MSFSSWPASRLWAMLYNAKVPVQVLPERFDTFPGRNPSLTRPSSSGSSDAPRKSATSGQYSFTRYNCRRTMPGVRKR

transcript_transcript/36207_Pt_Mix_transcript36207/f4p0/683

ATGTCCTTCTCCTCTTGGCCTGCCTCGCGGCTGTGGGCAATGCTTTACAACGCCAAGGTACCAGTCCAGGTCCTGCCAGAGAGATTCGATACCTTCCCGGGAAGAAATCCATCCTTGACCAGACCCAGCAGTAGTGGGAGTTCAGACGCTCCCAGGAAGAGTGCCACCTCAGACTTTACCGGGAGTAGTTGGAGTCAGTCCCTTGGGTCTCCCCGGGCCGCCCAGGCATCACAGGCTCCGTCAGGCCCTTCCAACGTCCAGGCCAATACTCGTTCACCCGCTACACTTCCTGATGACGACACATGA

MSFSSWPASRLWAMLYNAKVPVQVLPERFDTFPGRNPSLTRPSSSGSSDAPRKSATSDFTGSSWSQSLGSPRAAQASQAPSGPSNVQANTRSPATLPDDDT

transcript_transcript/36611_Pt_Mix_transcript36611/f6p0/623

ATGTCCTTCTCCTCTTGGCCTGCCTCGCGGCTGTGGGCAATGCTTTACAACGCCAAGGTACCAGTCCAGGTCCTGCCAGAGAGATTCGATACCTTCCCGGGTACGAAAGCGGTAATGCGAGGTGCCTGCAGGCTGTCTGGTGACTGTTGCGGCAATTCCTATCCAATCCCTTACAAAGGCTAG

MSFSSWPASRLWAMLYNAKVPVQVLPERFDTFPGTKAVMRGACRLSGDCCGNSYPIPYKG

transcript_transcript/37576_Pt_Mix_transcript37576/f4p0/529

ATGTCCTTCTCCTCTTGGCCTGCCTCGCGGCTGTGGGCAATGCTTTACAACGCCAAGGTACCAGTCCAGGTCCTGCCAGAGAGATTCGATACCTTCCCGGGAAGAAATCCATCCTTGACCAGACCCAGCAGTAGTGGAAGTTCAGACGCTCAGCCGACCATCTTCACCTGGTAA

MSFSSWPASRLWAMLYNAKVPVQVLPERFDTFPGRNPSLTRPSSSGSSDAQPTIFTW

transcript_transcript/19818_Pt_Mix_transcript19818/f3p0/1926

ATGATGGTGCGGTGGTTGCTGATGGCGGCAGTGGGCGTGCTGGCGGCGACTGCTGCGGCGCAGAACACCGATACTCCCTCCGACGAGAAGAGTTCCTCCCCAGGACAGACTCGCCTGGGGAATGCGGGCGGCGGTCAGGGCGGGTCAGAAGTGAACACCAGGTTCCTGTCACTGGGTCAGCTGGCGAACGTCGGCCAGACTCTGGTGAACGGCTTCCTTGGCAACAATCGCCCCAACAGACCCGGAGGAGGCTTCGGTAACCGACCCGGTTTTGGCGGCAACTTCGGAAATAGACCAGGCTTCGGTGGAGGCTTCGGGAACAGGCCAGGCTTTGGTGGAGGTTTTGGCGGCAACCAAGGTTTCGGTGGCGGCTTTGGTGGTAGCCAAGGTTTTGGTGGCGGCTTTGGTGGTAACCAAGGTTTTGGTGGCGGCTTCGGTAACAGGCCTGGTTTTGGTGGTGGTTTTGGTGGCGGTCAAGGCTTCGGTGGAGGTTTTGGTGGTTTTGGAAACAAGCCAGGGCGGTGCCCCCCTGTGCGGCCCCAATGTCCCCCAACAAGAAGCTTCTCCCCTCCGTCGCCGTGCAGAAATGACCGGCAGTGCAGCGGCTTCGACAAGTGCTGCTTTGACACCTGCCTCAGGGATCGCGTGTGCAAACCTGCCCAATGA

MMVRWLLMAAVGVLAATAAAQNTDTPSDEKSSSPGQTRLGNAGGGQGGSEVNTRFLSLGQLANVGQTLVNGFLGNNRPNRPGGGFGNRPGFGGNFGNRPGFGGGFGNRPGFGGGFGGNQGFGGGFGGSQGFGGGFGGNQGFGGGFGNRPGFGGGFGGGQGFGGGFGGFGNKPGRCPPVRPQCPPTRSFSPPSPCRNDRQCSGFDKCCFDTCLRDRVCKPAQ

transcript_transcript/22671_Pt_Mix_transcript22671/f5p0/1720

ATGAAGATGCAGACTGTAATAGCCATGGCAGTTGTGGCTACCATTGTGGCCATGACAGAAGCATCCCTAGTACTTCCATACCCAGGTCTGGATTGTAAGTACTGGTGCAAAGACAACTACGATAAACACTACTGCTGTGGCCCACCAGGACGTACCTATCCACCTTATACCGAGCGCTCTGGTAAATGTCCTCCGGTCCGTGCTACATGTACTGGTGTCAGGTCACGCCTACCAAAGTTGTGTCCCCATGATGGTGCTTGTGACTTTCCAAGCAAGTGCTGTTATGACGCCTGTGTGGAGCACCACGTATGCAAGACTCCTGATTTCTACTAA

MKMQTVIAMAVVATIVAMTEASLVLPYPGLDCKYWCKDNYDKHYCCGPPGRTYPPYTERSGKCPPVRATCTGVRSRLPKLCPHDGACDFPSKCCYDACVEHHVCKTPDFY

transcript_transcript/27210_Pt_Mix_transcript27210/f9p0/1421

ATGATGGTGCGGTGGTTGCTGATGGCGGCAGTGGGCGTGCTGGCGGCGACTGCTGCGGCGCAGAACACCGATACTCCCTCCGACGAGAAGAGTTCCTCCCCAGGACAGACTCGCCTGGGGAATGCGGGCGGCGGTCAGGGCGGGTCAGATGTGAACACCAGGTTCCTGTCACTGGGTCAGCTGGCGAACGTCGGCCAGACTCTGGTGAACAGCTTCCTTGGCAACAATCGCCCCAACAGACCCGGAGGAGGCTTCGGTAACCGACCCGGTTTTGGCGGCAACTTCGGAAATAGACCAGGCTTCGGTGGAGGCTTCGGGAACAGGCCAGGCTTTGGTGGAGGTTTTGGCGGCAACCAAGGTTTCGGTGGCGGCTTTGGTGGTAGCCAAGGTTTTGGTGGCGGCTTTGGTGGTAACCAAGGTTTTGGTGGCGGCTTCGGTAACAGGCCTGGTTTTGGTGGTGGTTTTGGTGGCGGTCAAGGCTTCGGTGGAGGTTTTGGTGGTTTTGGAAACAAGCCAGGGCGGTGCCCCCCTGTGCGGCCCCAATGTCCCCCAACAAGAAGCTTCTCCCCTCCGTCGCCGTGCAGAAATGACCGGCAGTGCAGCGGCTTCGACAAGTGCTGCTTTGACACCTGCCTCAGGGATCGCGTGTGCAAACCTGCCCAATGA

MMVRWLLMAAVGVLAATAAAQNTDTPSDEKSSSPGQTRLGNAGGGQGGSDVNTRFLSLGQLANVGQTLVNSFLGNNRPNRPGGGFGNRPGFGGNFGNRPGFGGGFGNRPGFGGGFGGNQGFGGGFGGSQGFGGGFGGNQGFGGGFGNRPGFGGGFGGGQGFGGGFGGFGNKPGRCPPVRPQCPPTRSFSPPSPCRNDRQCSGFDKCCFDTCLRDRVCKPAQ

transcript_transcript/28909_Pt_Mix_transcript28909/f21p0/1307

ATGAAGGTCCTGTGTCTCGTGGCGGCAGTCTGCGTGGTGGCGGTGGCGGGGGACGACACCAGGGGAGCATTTGGGAGCTTTAACAGACCTGGCAGACCTGGGAGACCTGGGAGTGGTATTCTGGGTAGCATTGTGGACGGTCTAACTGGACACGGCGGGGTACATGGGGGCGGTCATGGGGGTGTACATGGGGGCGGTCATGGGGGTGTACATGGGGGAATACAAGGAGGGCACGGAGGAATACACGGTGGCATTCACGGAGGCGGCCACGGCGGTCATGGAGGCATCGGGGGTCATATAGGTGGCCAGTGTGGTACCAATGTCCCCACGCACATAAGGGCTGGATGCAAGCACTTCACCAGGGATCGCTACGGCCAATACGTGTGTGACAGAAACCAGAAGCCAGCTTACCGGTGCCCGCCTGTCCGTCCCGAGTGCCCTCTTAACACCAGGTTCGGCCCGCCTATTCAGTGCACGTTGGATACAGACTGCCCTGGAATCTCGGACAAGTGCTGCTGTGACGCTTGCCTCAGCCATCCCGTGTGCAAACCTTCAGCCTAA

MKVLCLVAAVCVVAVAGDDTRGAFGSFNRPGRPGRPGSGILGSIVDGLTGHGGVHGGGHGGVHGGGHGGVHGGIQGGHGGIHGGIHGGGHGGHGGIGGHIGGQCGTNVPTHIRAGCKHFTRDRYGQYVCDRNQKPAYRCPPVRPECPLNTRFGPPIQCTLDTDCPGISDKCCCDACLSHPVCKPSA

transcript_transcript/28979_Pt_Mix_transcript28979/f44p0/1287

ATGAAGGTCCTGTGTCTCGTGGCGGCAGTCTGCGTGGTGGCGGTGGCGGGGGACGACACCAGGGGAGCATTTGGGAGCTTTAACAGACCTGGCAGACCTGGGAGACCTGGGAGTGGTATTCTGGGTAGCATTGTGGACGGTCTAACTGGACACGGCGGGGTACATGGGGGCGGTCATGGGGGTGTACATGGGGGAATACAAGGAGGGCACGGAGGAATACACGGTGGCATTCACGGAGGCGGCCACGGCGGTCATGGAGGCATCGGGGGTCATATAGGTGGCCAGTGTGGTACCAATGTCCCCACGCACATAAGGGCTGGATGCAAGCACTTCACCAGGGATCGCTACGGCCAATACGTGTGTGACAGAAACCAGAAGCCAGCTTACCGGTGCCCGCCTGTCCGTCCCGAGTGCCCTCTTAACACCAGGTTCGGCCCGCCTATTCAGTGCACGTTGGATACAGACTGCCCTGGAATCTCGGACAAGTGCTGCTGTGACGCTTGCCTCAGCCATCCCGTGTGCAAACCTTCAGCCTAA

MKVLCLVAAVCVVAVAGDDTRGAFGSFNRPGRPGRPGSGILGSIVDGLTGHGGVHGGGHGGVHGGIQGGHGGIHGGIHGGGHGGHGGIGGHIGGQCGTNVPTHIRAGCKHFTRDRYGQYVCDRNQKPAYRCPPVRPECPLNTRFGPPIQCTLDTDCPGISDKCCCDACLSHPVCKPSA

transcript_transcript/35324_Pt_Mix_transcript35324/f3p0/766

ATGAAGATGCAGACTGTAATAGCCATGGCAGTTGTGGCTACCATTGTGGCCATGACAGAAGCATCCCTAGTACTTCCATACCCAGGTCTGGATTGTAAGTACTGGTGCAAAGACAACTACGATAAAAACTACTGCTGTGGCCCACCAGGACGTACCTATCCACCTTATACCGAGCGCTCTGGTAAATGTCCTCCGGTCCGTGCTACATGTACTGGTGTCAGGTCACTCCTACCAAAGTTGTGTCCCCATGATGGTGCTTGTGACTTTCCAAGCAAGTGCTGTTATGACGCCTGTGTGGAGCACCACGTATGCAAGACTCCTGATTTCTACTAA

MKMQTVIAMAVVATIVAMTEASLVLPYPGLDCKYWCKDNYDKNYCCGPPGRTYPPYTERSGKCPPVRATCTGVRSLLPKLCPHDGACDFPSKCCYDACVEHHVCKTPDFY

transcript_transcript/35396_Pt_Mix_transcript35396/f6p0/746

ATGATGGTGCGGTGGTTGCTGATGGCGGCAGTGGGGGCGCAGAACACCGATACTCCCTCCGACGAGAAGAGTTCCTCCCCAGGACAGACTCGCCTGGGGAATGCGGGCGGCGGTCAGGGCGGGTCAGAAGTGAACACCAGGTTCCTGTCACTGGGTCAGCTGGCGAACGTCGGCCAGACTCTGGTGAACGGCTTCCTTGGCAACAATCGCCCCAACAGACCCGGAGGAGGCTTCGGTAACCGACCCGGTTTTGGCGGCAACTTCGGAAATAGACCAGGCTTCGGTGGAGGCTTCGGGAACAGGCCAGGCTTTGGTGGAGGTTTTGGCGGCAACCAAGGTTTCGGTGGCGGCTTTGGTGGTAGCCAAGGTTTTGGTGGCGGCTTTGGTGGTAACCAAGGTTTTGGTGGCGGCTTCGGTAACAGGCCTGGTTTTGGTGGTGGTTTTGGTGGCGGTCAAGGCTTCGGTGGAGGTTTTGGTGGTTTTGGAAACAAGCCAGGGCGGTGCCCCCCTGTGCGGCCCCAATGTCCCCCAACAAGAAGCTTCTCCCCTCCGTCGCCGTGCAGAAATGACCGGCAGTGCAGCGGCTTCGACAAGTGCTGCTTTGACACCTGCCTCAGGGATCGCGTGTGCAAACCTGCCCAATGA

MMVRWLLMAAVGAQNTDTPSDEKSSSPGQTRLGNAGGGQGGSEVNTRFLSLGQLANVGQTLVNGFLGNNRPNRPGGGFGNRPGFGGNFGNRPGFGGGFGNRPGFGGGFGGNQGFGGGFGGSQGFGGGFGGNQGFGGGFGNRPGFGGGFGGGQGFGGGFGGFGNKPGRCPPVRPQCPPTRSFSPPSPCRNDRQCSGFDKCCFDTCLRDRVCKPAQ

transcript_transcript/36826_Pt_Mix_transcript36826/f2p0/646

ATGACAGGAGCATCCCTAGTACTTCCATACTCAGTTCTGGATTGTAAGTACTGGTGCACAGACAACTACGATAAAAACTACTGCTGTGGCCCACCGGGACGTACCTATCCACCTTATACAGAGCGATCTGGTAAATGTCCTCCTGTCCGTGCTTCATGTACTGGTGTCAGGTCACTACTACCAAAGTTGTGTCCACATGATTGTGCTTGTGACTTTCCTAGCAAGTGCTTTTATGACGCCTGTGTGGAGCACTACGTATGCAAGACTCCTGATTTTTACTAA

MTGASLVLPYSVLDCKYWCTDNYDKNYCCGPPGRTYPPYTERSGKCPPVRASCTGVRSLLPKLCPHDCACDFPSKCFYDACVEHYVCKTPDFY

transcript_transcript/36834_Pt_Mix_transcript36834/f21p0/608

ATGACGTCACTGCGGATAGCGTTCCTGGTGTTGGTGGGATGCGTGGTGCCAGCTTACATGCAGTTTGGATCTAATTGTGTTCACTGGTGCCGGACACCTGAAAATCAAGTGTACTGTTGCAAGGATACACATGACACCCCGGCCTCTCCCATCCAAGTGGTTAATCATGGACGCTGCCCTCCTGTTCGTCCTGTATGTCCACCTGTCAGATCCTTCAGTCCGCCCCAGAGTTGCTCCAGCGACAGCGACTGCTACGGCCAGAAGTGCTGCTATGACAGATGTCTTGAGGAACACGTGTGCAAACCTCAGCATTATGGACATGGTGGGCACGGCGGACACGGCGGTCACGGTGGGCACGGAGGCTTTGGTGGGTTCGGCGGCGGATTTGGTCGCTAA

MTSLRIAFLVLVGCVVPAYMQFGSNCVHWCRTPENQVYCCKDTHDTPASPIQVVNHGRCPPVRPVCPPVRSFSPPQSCSSDSDCYGQKCCYDRCLEEHVCKPQHYGHGGHGGHGGHGGHGGFGGFGGGFGR

transcript_transcript/37084_Pt_Mix_transcript37084/f5p0/593

ATGCTTTGCATAGTGACAGTCGCCTTGTTGGTGCTCGTGGCGCTGAAAGATGCCGAGGCGGGGACGCCTCCTATATCTGACAGCTGCAGGCACTGGTGTCCAAGAGCAGGATATCCTCATGGCGAAGCGTTTTACTGTTGTGATCGTGGAATTGGGACTATAGGGGACGAGTTCGACTCGCACCCTGGCAAATGTCCCGACCGTCCTTTCTGCCCCAAGAATGAGTATCTCAGAATCGGACCAAGGCCCACGGTATGTGCTCATGACGGCCAGTGCAAACGTCACGAGAAGTGTTGTACTGATGCCTGCCTTAACCACCACACCTGCCTGATTGCTGACCCACATTAA

MLCIVTVALLVLVALKDAEAGTPPISDSCRHWCPRAGYPHGEAFYCCDRGIGTIGDEFDSHPGKCPDRPFCPKNEYLRIGPRPTVCAHDGQCKRHEKCCTDACLNHHTCLIADPH

transcript_transcript/37703_Pt_Mix_transcript37703/f15p0/516

ATGACAGAAGCATCCCTAGTACTTCCATACCCAGGTCTGGATTGTAAGTACTGGTGCAAAGACAACTACGATAAAAACTACTGCTGTGGCCCACCAGGACGTACCTATCCACCTTATACCGAGCGCTCTGGTAAATGTCCTCCGGTCCGTGCTACATGTACTGGTGTCAGGTCACTCCTACCAAAGTTGTGTCCCCATGATGGTGCTTGTGACTTTCCAAGCAAGTGCTGTTATGACGCCTGTGTGGAGCACCACGTATGCAAGACTCCTGATTTCTACTAA

MTEASLVLPYPGLDCKYWCKDNYDKNYCCGPPGRTYPPYTERSGKCPPVRATCTGVRSLLPKLCPHDGACDFPSKCCYDACVEHHVCKTPDFY

transcript_transcript/38303_Pt_Mix_transcript38303/f7p0/477

ATGAACCGAGTCGTGTGCGTGATGGTGGTGGTGGTGGCGGCGGCGGCGCTGTCATCTGCCAGCTCCCATTCCTGCAATACCTTCTGCGATGACCCAGACATTGACGCCGAGGGGCATTACGTGTGTTGTGACAGTTACCCCGGCACGTGTCCACCTATCGTTTTCTGTAACTACGGCCAAAGAAGGTGTCACTACGATGGTCAGTGCGGGCCGGGCTCCAAGTGCTGTGACTCTCGCTGTGGAAAGAGGTGCATTGCTTATCCCCCCCTCAAGTAA

MNRVVCVMVVVVAAAALSSASSHSCNTFCDDPDIDAEGHYVCCDSYPGTCPPIVFCNYGQRRCHYDGQCGPGSKCCDSRCGKRCIAYPPLK

transcript_transcript/3111_Pt_Mix_transcript3111/f3p0/3846

ATGGAGAACGAACAGAAGCAAGTGTTAGACATGCTCCAGCGACCCTTTCGTCTGCCAACTCGAAAAGAGGAGGATCATCCCATTGTGTTTGAAGTGGGTACCCGTGTGGCAGGAAGCCAACCACCACCTCAGCCCCTTGCTCTTGCTCGCTCCATTCCCAGAGGCACCGTCTTCTCTATTTTCGTGAAGAGCCATCGTCTGGCCGCTAAGGAACTTTGTGACTACTTCATGGAGACAAGCAGTGTGACGGAGCTCAAGCAAAGGGTGGATGAGATGCAAGGATTGGTCAATGAGAGTCTTTTCATCTTTGCCCTATCCTTTGTAATCATCAGGAAGCCGGAGATGAGACATCTGCGCCTGCCTAGCATTGTGGAAATCTTCCCCAGCATGTTTGTGCCAGTGACGACCGTGTCAGAGATGGAACACGAGGCCAAGAAATCCACCCCTGACCAAGAGATCATAGTAACAGAATATGGACCAGAGTTCTCCAGCACTCACCTCAAGCCTGAGCACCGAGTAGCCTACTGGCGTGAGGACTTTGGTATTAATTCCCACCACTGGCACTGGCATCTTGTCTACCCTGTGGACCTTGGTGTGAGTAGGGACCGCAAGGGGGAGCTTTTCTTCTACATGCACCAACAAATGCTTGCTCGTTATGACATGGATCGGCTGAGTGTCGGCCTGAACAGGGTGCAAAAACTGAGCAACTGGCGCATCCCCATCCCTGATGGCTACTTCCCCAAGCTGACCATCAACAACTCTGGCCAGACATGGGGCTCCCGGCAGGACAATACATTAATACAGGAATATAAGCGTGAAGAATTTGGGTTGCTGCCATTTGATGTCTCTACCCTTGAGTTGTGGCACAGGAGGATCATGGATGCCATTCATCAGGGCTATCTTATCAATCGTGATGATAACCTGACAAGGCTCACAGACAATGTCAAACCACCTGAGAAGCGTGGAATTGACCTTCTTGGTGACATAGTGGAAGCAGACAGCTACTTCAGCCTCAACAGTCTCTTCTACGGTGACCTGCACAACATGGGGCATGTCATCATTTCAGCCGCCCATGACCCAGACAATGCTCATCGAGAGAACCTTGGAGTTATGTCTCAGACAGCCACAGCCATGAGGGACCCTGTGTTCTATCGCTGGCACAAGTACATTGATGACATCTTCCAGGAGTACAAGCTCACTCAGCCACCATACACTGCTGAGGAACTGTCCCTGTCATCAGTTGAGGTGGTGTCAGTGGCTGTAGAGAGCCGAGGCCAGAAAAACCAGCTTATTACTGGCTGGAGCACTCGTGATTTTGAGGCGAGTCGTGGACTGGACTTCAATGCAGATAAGCCAGTAATAGTGCGACTCACCCATCTCAACCATCATCCCTTTGTCTATAGCATCAAGGTTGTCAACAGTGGCAGTGTATCAAAGGAAGTGACAGTGAGGATCTTCATGGCACCCAAACTTAATGAAAGAGGAGTGGAGATGAACTTCATGGAACAGCGTCTCTTCTGGGCTGAGATGGACCGCTTCACTCACGATTTGAAGCCTGGCCCAAACCACATTCTACGTTCCTCCACCTCCTCATCTATCACAAACTCAAATGACTTCACCTTCCGTGACCTGGAGAAGCAGCCGAACCCAGGCGAACCAGATGCTCCTGAAAACACCCTCTTTAATTTCTGTGGGTGTGGCTGGCCCCAACACATGCTACTGCCACGTGGGAAGCAAGAGGGAATGTCCTTTGAGCTCTTTGTGATGGTCACTGATTGGAGTCAGGACAAGGTGGCACAGCCTGATGGTGCCTGTTCCTGTTCTGCTGCTGCATCATTCTGTGGTATTCTGGATGCCCTCTACCCTGATGCCCAGCCCATGGGTTTCCCCTTCGACCGCCGCCCCTTGCCCATGTTACTCAACAGACACGTGGAAAGGACCTCAGATCTGACACGTCTCAGCAACATAGCCATGCAAGATATCACCATCACCTTCACCAATGCTAAGATCACCCAGTAA

MENEQKQVLDMLQRPFRLPTRKEEDHPIVFEVGTRVAGSQPPPQPLALARSIPRGTVFSIFVKSHRLAAKELCDYFMETSSVTELKQRVDEMQGLVNESLFIFALSFVIIRKPEMRHLRLPSIVEIFPSMFVPVTTVSEMEHEAKKSTPDQEIIVTEYGPEFSSTHLKPEHRVAYWREDFGINSHHWHWHLVYPVDLGVSRDRKGELFFYMHQQMLARYDMDRLSVGLNRVQKLSNWRIPIPDGYFPKLTINNSGQTWGSRQDNTLIQEYKREEFGLLPFDVSTLELWHRRIMDAIHQGYLINRDDNLTRLTDNVKPPEKRGIDLLGDIVEADSYFSLNSLFYGDLHNMGHVIISAAHDPDNAHRENLGVMSQTATAMRDPVFYRWHKYIDDIFQEYKLTQPPYTAEELSLSSVEVVSVAVESRGQKNQLITGWSTRDFEASRGLDFNADKPVIVRLTHLNHHPFVYSIKVVNSGSVSKEVTVRIFMAPKLNERGVEMNFMEQRLFWAEMDRFTHDLKPGPNHILRSSTSSSITNSNDFTFRDLEKQPNPGEPDAPENTLFNFCGCGWPQHMLLPRGKQEGMSFELFVMVTDWSQDKVAQPDGACSCSAAASFCGILDALYPDAQPMGFPFDRRPLPMLLNRHVERTSDLTRLSNIAMQDITITFTNAKITQ

transcript_transcript/12284_Pt_Mix_transcript12284/f3p0/2372

ATGCCCACCACCACCACTATCACGACTACTGAGACCACGCCCCCCACCACCACCATCACTACCAGTGAGACCACGCCCACCACCACTACGGCCATAGACACTACGACCACCATCACGACCACTGAGGCCACGACCACCACGAGTTGCACAGGATCGTGTCCTTGCGGTCTTGCCAATCTAGCGAGGGTCGTGGGCGGGGAGGATGTAGATCCTGCCTACAAGTACCCGTGGCATGTGGGAATAAAGTATGTATGGAACAGAAAGTACTGGTGCGGCGGCTCCATCATTAACGACCGGTATATCCTGACAGCCGCCCATTGTGTTAAAAACAGATTTAGACGGTGGCTGGTGGTGGGCGTGGCCGACCACGACATGACGTCCACCGACGGGGTGACCCAACTGGTACGCGTTCAGGAGATTATCGTTCATCCTGACTATAATATCAACACCCTTGACAGTGATATCGCGCTGCTCAAGCTGTCACAACCACTGGACCTGACTCAAGTCGAGCACATTCGCCCCGTGTGTCTGCCCGCCGATGACTCCAACACCTATGCCGGCGAAGATGCCACAGCGACAGGCTGGGGCATACTTCAGTCCTGGGGAAGTCAACCAGCAATACTGCAGGAAGTCACAGTGCCTATTCTGGATCCCTCCTGCCCGGGACAGATATCGAATTATATCACTGAAAACATGCTGTGTGCTGGCCTTGAAGAAGGTGGCAAGGACACCTGCCAGGGCGACGCGGGCGGTCCGCTTACTGTGCAAAATGACTGTTCCAAATATGAGCAAGTTGGCATCACCTCCTGGGGCTTTGGATGTGCCGACCCTGGCAGGCCCGGTGTATACACCAGGGTGAGCAGATTCCTTAACTGGATCTACGCCAACACTGTTGACGCCACCTACTGCCAGTAG

MPTTTTITTTETTPPTTTITTSETTPTTTTAIDTTTTITTTEATTTTSCTGSCPCGLANLARVVGGEDVDPAYKYPWHVGIKYVWNRKYWCGGSIINDRYILTAAHCVKNRFRRWLVVGVADHDMTSTDGVTQLVRVQEIIVHPDYNINTLDSDIALLKLSQPLDLTQVEHIRPVCLPADDSNTYAGEDATATGWGILQSWGSQPAILQEVTVPILDPSCPGQISNYITENMLCAGLEEGGKDTCQGDAGGPLTVQNDCSKYEQVGITSWGFGCADPGRPGVYTRVSRFLNWIYANTVDATYCQ

transcript_transcript/12712_Pt_Mix_transcript12712/f7p0/2329

ATGGACGTGAGGCTCTGTTGCCTCCTGCTGTTTGTCGCCGCATCCTGGATGCCCGGCCTGGCCAATGACTTTATGATAAGCCCCACTCACAGAGAGCTGCCTGCGCTTCACAAATCAGGACAAAACGAAAGGCGCCCTAAAGATGAAGTAGCCATTGTTGTGCCCAAAGCGAAAGAACAGCCGGTAGATGCCATCCAAGAAGGAACCATTGAGACTAAGCTGGGAGGACACCCCGTCGTTCACAAACAAATAGATATCTCTGGGAGGCCCAAGCAGGATGGAGACCCAGTGGAATGTGAAGGAAGAAAGGCAGTGGAGGTGGGCGAGGAGGGGCGCATCATCACCAGCCCGAATTATCCAGACAATTATTCCGACGAAGAAAAATGTGCCGTGAGGATTATCGCCGAGAACAGGGAATGCACGCTGGAGATTGACTGCTATGACTTCCACGTGCAGGACTCAGAGGATTGCAGAAAAGACTACCTGATGATTAAGGAGAATAGCGAGACGAAGGAGAAATTCTGCGGTGACGAGGGATTTATATATGAGAGTAGCAAAAATGCCGTAACGCTCAAGTTCAGATCCATCCGGCGGGTAACGGACAAAGGATTCTCCTGCTTAGCTCGCTCCGTATGTCCAACCACCACCACCACCACCACTTCCACTGAGACCACGCCTACCACCATCACGACTACTGAGACCACGCCCACTACCACCACGACCATGACCACTGAGACCACGCCCATCACCACCACTACCGCTGAGACCACGCCCATCACCACCACTACCGCTGAGACCACGCCCATCACCACCACTACCACTGAGACCACGCCCATCACCACCACTACCGCTGAGACCACGCCCATCACCACCACTACCACTGAGACCACGCCCATCACCACCACTACCACTGAGACCACGCCCACCACCACCACGACCACTGAGACCACGCCCACCACCACCACTATCACGACTACTGAGACCACGCCCACCACCACCACCATCACTACCACTGAGACCACGCCCACCACCACTACGGCCATAGACACTACGACCACCATCACGACCACTGAGGCCACGACCACCACGAGTTGCACAGGATCGTGTCCTTGCGGTCTTGCCAATCTAGCGAGGGTTGTGGGCGGGGAGGATGTAGATCCTGCCTACAAGTACCCGTGGCATGTGGGAATAAAGTATGTATGGAACAGAAAGTACTGGTGCGGCGGCTCCATCATTAACGACCGGTATATCCTGACAGCCGCCCATTGTGTTAAAAACAGATTTACGAGTAGACGGTGGCTGGTGGTGGGCGTGGCCGACCACGACATGACGTCCACCAACGGGGTGACCCAACTGGTACGCGTTCAGGAGATTATCGTTCATCCTGACTATAATATCAACACCCTTGACAGTGATATCGCGCTGCTCAAGCTGTCACAACCACTGGACCTGACTCAAGTCGAGCACATTCGCCCCGTGTGTCTGCCCGCCGATGACTCCAACACCTATGCCGGCGAAGATGCCACAGCGACAGGCTGGGGCATACTTCAGTCCTGGGGAAGTCAACCAGCAATACTGCAGGAAGTCACAGTGCCTATTCTGGATCCCTCCTGCCCGGGACAGATATCGAATTATATCACTGAAAACATGCTGTGTGCTGGCCTTGAAGAAGGTGGCAAGGACACCTGCCAGGGCGACGCGGGCGGTCCGCTTACTGTGCAAAATGACTGTTCCAAGTATGAGCAAGTTGGCATCACCTCCTGGGGCTTTGGATGTGCCGACCCTGGCAGGCCCGGTGTATACACCAGGGTGAGCAGATTCCTTAACTGGATCTACGCCAACACTGTTGACGCCACCTACTGCCAGTAG

MDVRLCCLLLFVAASWMPGLANDFMISPTHRELPALHKSGQNERRPKDEVAIVVPKAKEQPVDAIQEGTIETKLGGHPVVHKQIDISGRPKQDGDPVECEGRKAVEVGEEGRIITSPNYPDNYSDEEKCAVRIIAENRECTLEIDCYDFHVQDSEDCRKDYLMIKENSETKEKFCGDEGFIYESSKNAVTLKFRSIRRVTDKGFSCLARSVCPTTTTTTTSTETTPTTITTTETTPTTTTTMTTETTPITTTTAETTPITTTTAETTPITTTTTETTPITTTTAETTPITTTTTETTPITTTTTETTPTTTTTTETTPTTTTITTTETTPTTTTITTTETTPTTTTAIDTTTTITTTEATTTTSCTGSCPCGLANLARVVGGEDVDPAYKYPWHVGIKYVWNRKYWCGGSIINDRYILTAAHCVKNRFTSRRWLVVGVADHDMTSTNGVTQLVRVQEIIVHPDYNINTLDSDIALLKLSQPLDLTQVEHIRPVCLPADDSNTYAGEDATATGWGILQSWGSQPAILQEVTVPILDPSCPGQISNYITENMLCAGLEEGGKDTCQGDAGGPLTVQNDCSKYEQVGITSWGFGCADPGRPGVYTRVSRFLNWIYANTVDATYCQ

transcript_transcript/13274_Pt_Mix_transcript13274/f5p0/2404

ATGATTAAGGAGAATAGCGAGACGAAGGAGAAATTCTGCGGTGACGAGGGATTTATATATGAGAGTAGCAAAAATGCCGTAACGCTCAAGTTCAGATCCATCCGGCGGGTAACGGACAAAGGATTCTCCTGCTTAGCTCGCTCCGTATGTCCAACCACCACCACCACCACCACTTCCACTGAGACCACGCCTACCACCATCACGACTACTGAGACCACGCCCACTACCACCACGACCATGACCACTGAGACCACGCCCATCACCACCACTACCGCTGAGACCACGCCCATCACCACCACTACCGCTGAGACCACGCCCATCACCACCACTACCACTGAGACCACGCCCATCACCACCACTACCGCTGAGACCACGCCCATCACCACCACTACCACTGAGACCACGCCCATCACCACCACTACCACTGAGACCACGCCCACCACCACCACGACCACTGAGACCACGCCCACCACCACCACTATCACGACTACTGAGACCACGCCCACCACCACCACCATCACTACCACTGAGACCACGCCCACCACCACTACGGCCATAGACACTACGACCACCATCACGACCACTGAGGCCACGACCACCACGAGTTGCACAGGATCGTGTCCTTGCGGTCTTGCCAATCTAGCGAGGGTTGTGGGCGGGGAGGATGTAGATCCTGCCTACAAGTACCCGTGGCATGTGGGAATAAAGTATGTATGGAACAGAAAGTACTGGTGCGGCGGCTCCATCATTAACGACCGGTATATCCTGACAGCCGCCCATTGTGTTAAAAACAGATTTACGAGTAGACGGTGGCTGGTGGTGGGCGTGGCCGACCACGACATGACGTCCACCAACGGGGTGACCCAACTGGTACGCGTTCAGGAGATTATCGTTCATCCTGACTATAATATCAACACCCTTGACAGTGATATCGCGCTGCTCAAGCTGTCACAACCACTGGACCTGACTCAAGTCGAGCACATTCGCCCCGTGTGTCTGCCCGCCGATGACTCCAACACCTATGCCGGCGAAGATGCCACAGCGACAGGCTGGGGCATACTTCAGTCCTGGGGAAGTCAACCAGCAATACTGCAGGAAGTCACAGTGCCTATTCTGGATCCCTCCTGCCCGGGACAGATATCGAATTATATCACTGAAAACATGCTGTGTGCTGGCCTTGAAGAAGGTGGCAAGGACACCTGCCAGGGCGACGCGGGCGGTCCGCTTACTGTGCAAAATGACTGTTCCAAGTATGAGCAAGTTGGCATCACCTCCTGGGGCTTTGGATGTGCCGACCCTGGCAGGCCCGGTGTATACACCAGGGTGAGCAGATTCCTTAACTGGATCTACGCCAACACTGTTGACGCCACCTACTGCCAGTAG

MIKENSETKEKFCGDEGFIYESSKNAVTLKFRSIRRVTDKGFSCLARSVCPTTTTTTTSTETTPTTITTTETTPTTTTTMTTETTPITTTTAETTPITTTTAETTPITTTTTETTPITTTTAETTPITTTTTETTPITTTTTETTPTTTTTTETTPTTTTITTTETTPTTTTITTTETTPTTTTAIDTTTTITTTEATTTTSCTGSCPCGLANLARVVGGEDVDPAYKYPWHVGIKYVWNRKYWCGGSIINDRYILTAAHCVKNRFTSRRWLVVGVADHDMTSTNGVTQLVRVQEIIVHPDYNINTLDSDIALLKLSQPLDLTQVEHIRPVCLPADDSNTYAGEDATATGWGILQSWGSQPAILQEVTVPILDPSCPGQISNYITENMLCAGLEEGGKDTCQGDAGGPLTVQNDCSKYEQVGITSWGFGCADPGRPGVYTRVSRFLNWIYANTVDATYCQ

transcript_transcript/13609_Pt_Mix_transcript13609/f2p0/2396

ATGTCAGACGCCACGACAACCAACCGTGACTGGCGTAGGTTGAATATAACACTGTCTGTCATTCCACAGCTGTTTGTCGCCGCATCCTGGATGCCCGGCCTGGCCAATGACTTTATGATAAGCCCCACTCACAGAGAGCTGCCTGCGCTTCACAAATCAGGACAAAACGAAAGGCGCCCCAAAGATGAAGTAGCCATTGTTGTGCCCAAAGCGAAAGAACAGCCGGTAGATGCCATCCAAAAAGGAACCATTGAGACTAAGCTGGGAGGACACCCCGTCGTTCACAAACAAATAGATATCTCTGGGAGGCCCAAGCAGGATGGAGACCCAGTGGAATGTGAAGGAAGAAAGGCAGTGGTGGTGGGCGAGGAGGGGCGCATCATCACCAGCCCGAATTATCCAGACAATTATTCCGACGAAGAAATATGTGCCGTGAGGATTATCGCCGAGAACAGGGAATGCACGCTGGAGATTGACTGCTCCGACTTCCACGTGCAGGACTCAGAGGATTGCAGAAAAGACTACCTGATGATTAAGGAGAATAGCGAGACGAAGGAGAAATTCTGCGGTGACGAGGGATTTATATATGAGAGTAGCAAAAATGCCGTAACGCTCAAGTTCAAATCCAACCGGCGGGTAACGGACAAAGGATTCTCCTGCTTAGCTCGCTCCGTATGTCCAACCACCACCACCACCACCACTTCCACTGAGACCACGCCTACCACCATTACGACTACTGAGACCACGCCCACTACCACCACGACCATGACCACTGAGACCACGCCCATCACCACCACTACCGCTGAGACCACGCCCATCACCACCACTACCGCTGAGACCACGCCCATCACCACCACTACCACTGAGACCACGCCCATCACCACCACTACCGCTGAGACCACGCCCATCACCACCACTACCACTGAGACCACGCCCATCACCACCACTACCACTGAGACCACGCCCACCACCACCACGACCACTGAGACCACGCCCACCACCACCACTATCACGACTACTGAGACCACGCCCACCACCACCACCATCACTACCAGTGAGACCACGCCCACCACCACTACGGCCATAGACACTACGACCACCATCACGACCACTGAGGCCACGACCACCACGAGTTGCACAGGATCGTGTCCTTGCGGTCTTGCCAATCTAGCGAGGGTCGTGGGCGGGGAGGATGTAGATCCTGCCTACAAGTACCCGTGGCATGTGGGAATAAAGTATGTATGGAACAGAAAGTACTGGTGCGGCGGCTCCATCATTAACGACCGGTATATCCTGACAGCCGCCCATTGTGTTAAAAACAGATTTAGACGGTGGCTGGTGGTGGGCGTGGCCGACCACGACATGACGTCCACCGACGGGGTGACCCAACTGGTACGCGTTCAGGAGATTATCGTTCATCCTGACTATAATATCAACACCCTTGACAGTGATATCGCGCTGCTCAAGCTGTCACAACCACTGGACCTGACTCAAGTCGAGCACATTCGCCCCGTGTGTCTGCCCGCCGATGACTCCAACACCTATGCCGGCGAAGATGCCACAGCGACAGGCTGGGGCATACTTCAGTCCTGGGGAAGTCAACCAGCAATACTGCAGGAAGTCACAGTGCCTATTCTGGATCCCTCCTGCCCGGGACAGATATCGAATTATATCACTGAAAACATGCTGTGTGCTGGCCTTGAAGAAGGTGGCAAGGACACCTGCCAGGGCGACGCGGGCGGTCCGCTTACTGTGCAAAATGACTGTTCCAAGTATGAGCAAGTTGGCATCACCTCCTGGGGCTTTGGATGTGCCGACCCTGGCAGGCCCGGTGTATACACCAGGGTGAGCAGATTCCTTAACTGGATCTACGCCAACACTGTTGACGCCACCTACTGCCAGTAG

MSDATTTNRDWRRLNITLSVIPQLFVAASWMPGLANDFMISPTHRELPALHKSGQNERRPKDEVAIVVPKAKEQPVDAIQKGTIETKLGGHPVVHKQIDISGRPKQDGDPVECEGRKAVVVGEEGRIITSPNYPDNYSDEEICAVRIIAENRECTLEIDCSDFHVQDSEDCRKDYLMIKENSETKEKFCGDEGFIYESSKNAVTLKFKSNRRVTDKGFSCLARSVCPTTTTTTTSTETTPTTITTTETTPTTTTTMTTETTPITTTTAETTPITTTTAETTPITTTTTETTPITTTTAETTPITTTTTETTPITTTTTETTPTTTTTTETTPTTTTITTTETTPTTTTITTSETTPTTTTAIDTTTTITTTEATTTTSCTGSCPCGLANLARVVGGEDVDPAYKYPWHVGIKYVWNRKYWCGGSIINDRYILTAAHCVKNRFRRWLVVGVADHDMTSTDGVTQLVRVQEIIVHPDYNINTLDSDIALLKLSQPLDLTQVEHIRPVCLPADDSNTYAGEDATATGWGILQSWGSQPAILQEVTVPILDPSCPGQISNYITENMLCAGLEEGGKDTCQGDAGGPLTVQNDCSKYEQVGITSWGFGCADPGRPGVYTRVSRFLNWIYANTVDATYCQ

transcript_transcript/13869_Pt_Mix_transcript13869/f27p0/2345

ATGGACGTGAGGGTCTGTTGCCTCCTGCTGTTTGTCGCCGCATCCTGGGTGCCCGGCCTAGCCAATGACTTTGTGATAAGCCCTACCCACGGAGAGCTCCCAGCGCTTCACAAACGAGAACAAAACGCCAGGGGCCCAAGAGATGAAGAAACCATTCAGTCCAAGGCGGGAGAACAGCCGGTAAATGCCATTCATGAAGGAACCATTGAGCCTAAGCTGGGAGGTAACCCCGTGGTTCACAAACAACCAGAAATCTTTGGAAGGCCCAAGCAGGATGGAGACCCCGTGGAATGTGAAGGAAGAAAAAGAGTGGTGGTGGGCGAGGAGGGGCGCATCATCACCAGCCCGAATTATCCAGACAATTATCCCAACCGAGAAGAATGTGCCGTGAGGATTGTCGCCGAGAACAGGGAATGCACGCTGGAGATTGACTGCTATGACTTCCACGTGCAGGACTCAGAGGATTGCAGAAAAGACTACCTGATGATTAAGGAGAATAGGTGGACGAAGGAGAAATTCTGCGGTGACGAGGGATTTATTTACGAGAGTAGCAAAAGAGCCGTAACACTCAAGTTCAAATCCAACCGGCGGGTAACGGACGAAGGATTCTCCTGCTTAGCTCGCTCCGTATGTCCAACCACCACCACCACCACCACCACCGAGACCACGCCCACCACCACCACGACCACTGAAACCACCACGCCCACCACCACCATCACGACCACTGAAACCACCACGCCCACCACCACCACCACCACGACCACTGAGGTCACGACCACCACGAGTAGCACAGGATCGTGTCAGTGCGGTGTTGCCAATCTAGCGAGGATCGTGGGCGGGGAGGAGGTAGATCCTGCCCACAAGTACCCGTGGCATGTGGGAATAAAGTATGTATGGAACAGTAATTACTGGTGCGGCGGCTCCATCATTAACGACCGGTATATCCTGACAGCCGCCCACTGTGTTGACGACATAACATCTGTACAAGGGCTGGTGGTGGGCGTGGCTGACCACGATATGACGTCCACCGACGATGTCACCCAACTGGTACCCGTTCAGGAGATTATCGTTCATCCTGACTATAATTCCAACACCCTTGACAGTGATATCGCGCTGCTCAAGCTGTCACAACCACTGGACCTGACTCAAGTCGAGCACATTCGCCCCGTGTGTTTACCCGCCGATGACTCCAACACCTATGCCGGCGAAGATGCCACAGCGACAGGCTGGGGCACACTTCAGTCCGGGGGAAGTCAACCAGCAATACTGCAGGAAGTCACAGTGCCTATTCTGGATCCCTCCTGCCCGGGACACAAACCGAGTCATATCACTGAAAACATGCTGTGTGCTGGCCTTGAAGAAGGTGGCAAGGACACCTGCCAGGGCGACTCGGGCGGTCCGCTTACCGTGCAAAATGACTCTTCCAAGTATGAGCAAATTGGCATCACCTCCTGGGGCTTAGGATGTGCCGACCCTGGCAGCCCCGGTGTGTACACCAGGGTGAGCAACTTCCTTGAGTGGATAGAGGAGAACACTACTGACGCCACCTACTGCCAGTAG

MDVRVCCLLLFVAASWVPGLANDFVISPTHGELPALHKREQNARGPRDEETIQSKAGEQPVNAIHEGTIEPKLGGNPVVHKQPEIFGRPKQDGDPVECEGRKRVVVGEEGRIITSPNYPDNYPNREECAVRIVAENRECTLEIDCYDFHVQDSEDCRKDYLMIKENRWTKEKFCGDEGFIYESSKRAVTLKFKSNRRVTDEGFSCLARSVCPTTTTTTTTETTPTTTTTTETTTPTTTITTTETTTPTTTTTTTTEVTTTTSSTGSCQCGVANLARIVGGEEVDPAHKYPWHVGIKYVWNSNYWCGGSIINDRYILTAAHCVDDITSVQGLVVGVADHDMTSTDDVTQLVPVQEIIVHPDYNSNTLDSDIALLKLSQPLDLTQVEHIRPVCLPADDSNTYAGEDATATGWGTLQSGGSQPAILQEVTVPILDPSCPGHKPSHITENMLCAGLEEGGKDTCQGDSGGPLTVQNDSSKYEQIGITSWGLGCADPGSPGVYTRVSNFLEWIEENTTDATYCQ

transcript_transcript/13889_Pt_Mix_transcript13889/f13p0/2326

ATGATTAAGGAGAATAGCGAGACGAAGGAGAAATTCTGCGGTGACGAGGGATTTATATATGAGAGTAGCAAAAATGCCGTAACGCTCAAGTTCAAATCCAACCGGCGGGTAACGGACAAAGGATTCTCCTGCTTAGCTCGCTCCGTATGTCCAACCACCACCACCACCACCACTTCCACTGAGACCACGCCTACCACCATTACGACTACTGAGACCACGCCCACTACCACCACGACCATGACCACTGAGACCACGCCCATCACCACCACTACCGCTGAGACCACGCCCATCACCACCACTACCGCTGAGACCACGCCCATCACCACCACTACCACTGAGACCACGCCCATCACCACCACTACCGCTGAGACCACGCCCATCACCACCACTACCACTGAGACCACGCCCATCACCACCACTACCACTGAGACCACGCCCACCACCACCACGACCACTGAGACCACGCCCACCACCACCACTATCACGACTACTGAGACCACGCCCACCACCACCACCATCACTACCAGTGAGACCACGCCCACCACCACTACGGCCATAGACACTACGACCACCATCACGACCACTGAGGCCACGACCACCACGAGTTGCACAGGATCGTGTCCTTGCGGTCTTGCCAATCTAGCGAGGGTCGTGGGCGGGGAGGATGTAGATCCTGCCTACAAGTACCCGTGGCATGTGGGAATAAAGTATGTATGGAACAGAAAGTACTGGTGCGGCGGCTCCATCATTAACGACCGGTATATCCTGACAGCCGCCCATTGTGTTAAAAACAGATTTAGACGGTGGCTGGTGGTGGGCGTGGCCGACCACGACATGACGTCCACCGACGGGGTGACCCAACTGGTACGCGTTCAGGAGATTATCGTTCATCCTGACTATAATATCAACACCCTTGACAGTGATATCGCGCTGCTCAAGCTGTCACAACCACTGGACCTGACTCAAGTCGAGCACATTCGCCCCGTGTGTCTGCCCGCCGATGACTCCAACACCTATGCCGGCGAAGATGCCACAGCGACAGGCTGGGGCATACTTCAGTCCTGGGGAAGTCAACCAGCAATACTGCAGGAAGTCACAGTGCCTATTCTGGATCCCTCCTGCCCGGGACAGATATCGAATTATATCACTGAAAACATGCTGTGTGCTGGCCTTGAAGAAGGTGGCAAGGACACCTGCCAGGGCGACGCGGGCGGTCCGCTTACTGTGCAAAATGACTGTTCCAAGTATGAGCAAGTTGGCATCACCTCCTGGGGCTTTGGATGTGCCGACCCTGGCAGGCCCGGTGTATACACCAGGGTGAGCAGATTCCTTAACTGGATCTACGCCAACACTGTTGACGCCACCTACTGCCAGTAG

MIKENSETKEKFCGDEGFIYESSKNAVTLKFKSNRRVTDKGFSCLARSVCPTTTTTTTSTETTPTTITTTETTPTTTTTMTTETTPITTTTAETTPITTTTAETTPITTTTTETTPITTTTAETTPITTTTTETTPITTTTTETTPTTTTTTETTPTTTTITTTETTPTTTTITTSETTPTTTTAIDTTTTITTTEATTTTSCTGSCPCGLANLARVVGGEDVDPAYKYPWHVGIKYVWNRKYWCGGSIINDRYILTAAHCVKNRFRRWLVVGVADHDMTSTDGVTQLVRVQEIIVHPDYNINTLDSDIALLKLSQPLDLTQVEHIRPVCLPADDSNTYAGEDATATGWGILQSWGSQPAILQEVTVPILDPSCPGQISNYITENMLCAGLEEGGKDTCQGDAGGPLTVQNDCSKYEQVGITSWGFGCADPGRPGVYTRVSRFLNWIYANTVDATYCQ

transcript_transcript/14674_Pt_Mix_transcript14674/f3p0/2296

ATGGACGTGAGGCTCTGTTGCCTCCTGCTGTTTGTCGCCGCATCCTGGATGCCCGGCCTGGCCAATGACTTTATGATAAGCCCCACTCACAGAGAGCTGCCTGCGCTTCACAAATCAGGACAAAACGAAAGGCGCCCTAAAGATGAAGTAGCCATTGTTGTGCCCAAAGCGAAAGAACAGCCGGTAGATGCCATCCAAGAAGGAACCATTGAGACTAAGCTGGGAGGACACCCCGTCGTTCACAAACAAATAGATATCTCTGGGAGGCCCAAGCAGGATGGAGACCCAGTGGAATGTGAAGGAAGAAAGGCAGTGGAGGTGGGCGAGGAGGGGCGCATCATCACCAGCCCGAATTATCCAGACAATTATTCCGACGAAGAAAAATGTGCCGTGAGGATTATCGCCGAGAACAGGGAATGCACGCTGGAGATTGACTGCTATGACTTCCACGTGCAGGACTCAGAGGATTGCAGAAAAGACTACCTGATGATTAAGGAGAATAGCGAGACGAAGGAGAAATTCTGCGGTGACGAGGGATTTATATATGAGAGTAGCAAAAATGCCGTAACGCTCAAGTTCAGATCCATCCGGCGGGTAACGGACAAAGGATTCTCCTGCTTAGCTCGCTCCGTATGTCCAACCACCACCACCACCACCACTTCCACTGAGACCACGCCTACCACCATCACGACTACTGAGACCACGCCCACTACCACCACGACCATGACCACTGAGACCACGCCCATCACCACCACTACCGCTGAGACCACGCCCATCACCACCACTACCACTGAGACCACGCCCATCACCACCACTACCGCTGAGACCACGCCCATCACCACCACTACCACTGAGACCACGCCCATCACCACCACTACCACTGAGACCACGCCCACCACCACCACGACCACTGAGACCACGCCCACCACCACCACTATCACGACTACTGAGACCACGCCCACCACCACCACCATCACTACCACTGAGACCACGCCCACCACCACTACGGCCATAGACACTACGACCACCATCACGACCACTGAGGCCACGACCACCACGAGTTGCACAGGATCGTGTCCTTGCGGTCTTGCCAATCTAGCGAGGGTTGTGGGCGGGGAGGATGTAGATCCTGCCTACAAGTACCCGTGGCATGTGGGAATAAAGTATGTATGGAACAGAAAGTACTGGTGCGGCGGCTCCATCATTAACGACCGGTATATCCTGACAGCCGCCCATTGTGTTAAAAACAGATTTACGAGTAGACGGTGGCTGGTGGTGGGCGTGGCCGACCACGACATGACGTCCACCAACGGGGTGACCCAACTGGTACGCGTTCAGGAGATTATCGTTCATCCTGACTATAATATCAACACCCTTGACAGTGATATCGCGCTGCTCAAGCTGTCACAACCACTGGACCTGACTCAAGTCGAGCACATTCGCCCCGTGTGTCTGCCCGCCGATGACTCCAACACCTATGCCGGCGAAGATGCCACAGCGACAGGCTGGGGCATACTTCAGTCCTGGGGAAGTCAACCAGCAATACTGCAGGAAGTCACAGTGCCTATTCTGGATCCCTCCTGCCCGGGACAGATATCGAATTATATCACTGAAAACATGCTGTGTGCTGGCCTTGAAGAAGGTGGCAAGGACACCTGCCAGGGCGACGCGGGCGGTCCGCTTACTGTGCAAAATGACTGTTCCAAGTATGAGCAAGTTGGCATCACCTCCTGGGGCTTTGGATGTGCCGACCCTGGCAGGCCCGGTGTATACACCAGGGTGAGCAGATTCCTTAACTGGATCTACGCCAACACTGTTGACGCCACCTACTGCCAGTAG

MDVRLCCLLLFVAASWMPGLANDFMISPTHRELPALHKSGQNERRPKDEVAIVVPKAKEQPVDAIQEGTIETKLGGHPVVHKQIDISGRPKQDGDPVECEGRKAVEVGEEGRIITSPNYPDNYSDEEKCAVRIIAENRECTLEIDCYDFHVQDSEDCRKDYLMIKENSETKEKFCGDEGFIYESSKNAVTLKFRSIRRVTDKGFSCLARSVCPTTTTTTTSTETTPTTITTTETTPTTTTTMTTETTPITTTTAETTPITTTTTETTPITTTTAETTPITTTTTETTPITTTTTETTPTTTTTTETTPTTTTITTTETTPTTTTITTTETTPTTTTAIDTTTTITTTEATTTTSCTGSCPCGLANLARVVGGEDVDPAYKYPWHVGIKYVWNRKYWCGGSIINDRYILTAAHCVKNRFTSRRWLVVGVADHDMTSTNGVTQLVRVQEIIVHPDYNINTLDSDIALLKLSQPLDLTQVEHIRPVCLPADDSNTYAGEDATATGWGILQSWGSQPAILQEVTVPILDPSCPGQISNYITENMLCAGLEEGGKDTCQGDAGGPLTVQNDCSKYEQVGITSWGFGCADPGRPGVYTRVSRFLNWIYANTVDATYCQ

transcript_transcript/15402_Pt_Mix_transcript15402/f2p0/2289

ATGGGTCACGTGAATGTGGCGGTGGTGGTAGTGGCGGCGATGGCGGCGCTGGTGGGTTGCCAGACTCAGCAGGGCAGCAGCTGCGTGGACGGGAACGGACAAGCGGGAACCTGCATCAGCATCCGCTCCTGCCAGCCACTGCAGCAGCTCGTGCAGGCCTTAAGAACTAACACAGGACCTCCCAACGGCTTCCAAATTCTGCGTCGGTCAGTATGTAACATACAAATTAACAGAGAGCCACTGGTGTGCTGTGCCAGCAGTGGTGGTACTGGGCTTGACTTGTTGCCCAAGCGCTGTGGAGTGACGGGTCTGGTGGACCGCATTGTTGACGGGGAAGACGCTCCGCTGCTCGCCTGGCCTTGGATGGCGCTGCTCAGAGGAAAAGCTCGTGGTCAGCCAAGCACTTGGATCTGCGGTGGTGTGCTCATAAACGCTCGCTACGTCCTCACTGCAGCCCATTGCTTCAAGTCCATCTTCCAAATTGAACTGGAGTTCGTAAGGATTGGCGAGCACACGCTGAGCACGGTGCAGGACTGTGAGAATGGTTTGTGTGCTCCACTTGCCCAGGATATCTTGGTGGAGCAAATAATCATGCATCCAGAGTATGGATCTCCATGCAAGGAGTGCAACGACATTGCGCTGCTCAGGCTGTCCCATCCCGCCCAGCTGAACCCTATTCATGTGGTGCCAATTTGCCTTCCCATCAACGAGGGAAACGTTTGTAATCAATTGAAAAGTGGCTATCCGAATCGTCGATGGACTCTGTGTGCTGGCGGGGAGGGCAAGGACACCTGCAAGGGAGATTCAGGCGGACCACTCATACTGAGTAATAAAATTGGAACCAAAGGATTTGTGGTGGGCATCACAAGCGTGGGACCAAAGGTGTGTGGCAGGCAGAAGACTCAGGCCCTTTACACCAATGTTCACTTTTACGTGCCGTGGATCCTCAAGAACCTGCGGCCTTAG

MGHVNVAVVVVAAMAALVGCQTQQGSSCVDGNGQAGTCISIRSCQPLQQLVQALRTNTGPPNGFQILRRSVCNIQINREPLVCCASSGGTGLDLLPKRCGVTGLVDRIVDGEDAPLLAWPWMALLRGKARGQPSTWICGGVLINARYVLTAAHCFKSIFQIELEFVRIGEHTLSTVQDCENGLCAPLAQDILVEQIIMHPEYGSPCKECNDIALLRLSHPAQLNPIHVVPICLPINEGNVCNQLKSGYPNRRWTLCAGGEGKDTCKGDSGGPLILSNKIGTKGFVVGITSVGPKVCGRQKTQALYTNVHFYVPWILKNLRP

transcript_transcript/15460_Pt_Mix_transcript15460/f25p0/2178

ATGGACGTGAGGCTCTGTTGCCTCCTGCTGTTTGTCGCCGCATCCTGGATGCCCGGCCTGGCCAATGACTTTATGATAAGCCCCACTCACAGAGAGCTGCCTGCGCTTCACAAATCAGGACAAAACGAAAGGCGCCCCAAAGATGAAGTAGCCATTGTTGTGCCCAAAGCGAAAGAACAGCCGGTAGATGCCATCCAAAAAGGAACCATTGAGACTAAGCTGGGAGGACACCCCGTCGTTCACAAACAAATAGATATCTCTGGGAGGCCCAAGCAGGATGGAGACCCAGTGGAATGTGAAGGAAGAAAGGCAGTGGTGGTGGGCGAGGAGGGGCGCATCATCACCAGCCCGAATTATCCAGACAATTATTCCGACGAAGAAATATGTGCCGTGAGGATTATCGCCGAGAACAGGGAATGCACGCTGGAGATTGACTGCTCCGACTTCCACGTGCAGGACTCAGAGGATTGCAGAAAAGACTACCTGATGATTAAGGAGAATAGCGAGACGAAGGAGAAATTCTGCGGTGACGAGGGATTTATATATGAGAGTAGCAAAAATGCCGTAACGCTCAAGTTCAAATCCAACCGGCGGGTAACGGACAAAGGATTCTCCTGCTTAGCTCGCTCCGTATGTCCAACCACCACCACCACCACCACTTCCACTGAGACCACGCCTACCACCATCACGACTACTGAGACCACGCCCACTACCACCACGACCATGACCACTGAGACCACGCCCATCACCACCACTACCGCTGAGACCACGCCCATCACCACCACTACCGCTGAGACCACGCCCATCACCACCACTACCACTGAGACCACGCCCATCACCACCACTACCACTGAGACCACGCCCACCACCACCACGACCACTGAGACCACGCCCACCACCACCACTATCACGACTACTGAGACCACGCCCACCACCACCACCATCACTACCAGTGAGACCACGCCCACCACCACTACGGCCATAGACACTACGACCACCATCACGACCACTGAGGCCACGACCACCACGAGTTGCACAGGATCGTGTCCTTGCGGTCTTGCCAATCTAGCGAGGGTCGTGGGCGGGGAGGATGTAGATCCTGCCTACAAGTACCCGTGGCATGTGGGAATAAAGTATGTATGGAACAGAAAGTACTGGTGCGGCGGCTCCATCATTAACGACCGGTATATCCTGACAGCCGCCCATTGTGTTAAAAACAGATTTAGACGGTGGCTGGTGGTGGGCGTGGCCGACCACGACATGACGTCCACCGACGGGGTGACCCAACTGGTACGCGTTCAGGAGATTATCGTTCATCCTGACTATAATATCAACACCCTTGACAGTGATATCGCGCTGCTCAAGCTGTCACAACCACTGGACCTGACTCAAGTCGAGCACATTCGCCCCGTGTGTCTGCCCGCCGATGACTCCAACACCTATGCCGGCGAAGATGCCACAGCGACAGGCTGGGGCATACTTCAGTCCTGGGGAAGTCAACCAGCAATACTGCAGGAAGTCACAGTGCCTATTCTGGATCCCTCCTGCCCGGGACAGATATCGAATTATATCACTGAAAACATGCTGTGTGCTGGCCTTGAAGAAGGTGGCAAGGACACCTGCCAGGGCGACGCGGGCGGTCCGCTTACTGTGCAAAATGACTGTTCCAAATATGAGCAAGTTGGCATCACCTCCTGGGGCTTTGGATGTGCCGACCCTGGCAGGCCCGGTGTATACACCAGGGTGAGCAGATTCCTTAACTGGATCTACGCCAACACTGTTGACGCCACCTACTGCCAGTAG

MDVRLCCLLLFVAASWMPGLANDFMISPTHRELPALHKSGQNERRPKDEVAIVVPKAKEQPVDAIQKGTIETKLGGHPVVHKQIDISGRPKQDGDPVECEGRKAVVVGEEGRIITSPNYPDNYSDEEICAVRIIAENRECTLEIDCSDFHVQDSEDCRKDYLMIKENSETKEKFCGDEGFIYESSKNAVTLKFKSNRRVTDKGFSCLARSVCPTTTTTTTSTETTPTTITTTETTPTTTTTMTTETTPITTTTAETTPITTTTAETTPITTTTTETTPITTTTTETTPTTTTTTETTPTTTTITTTETTPTTTTITTSETTPTTTTAIDTTTTITTTEATTTTSCTGSCPCGLANLARVVGGEDVDPAYKYPWHVGIKYVWNRKYWCGGSIINDRYILTAAHCVKNRFRRWLVVGVADHDMTSTDGVTQLVRVQEIIVHPDYNINTLDSDIALLKLSQPLDLTQVEHIRPVCLPADDSNTYAGEDATATGWGILQSWGSQPAILQEVTVPILDPSCPGQISNYITENMLCAGLEEGGKDTCQGDAGGPLTVQNDCSKYEQVGITSWGFGCADPGRPGVYTRVSRFLNWIYANTVDATYCQ

transcript_transcript/16405_Pt_Mix_transcript16405/f2p0/2167

ATGGAGACCCAGTGGAATGTGAAGGAAGAAATATGTGCCGTGAGGATTATCGCCGAGAACAGGGAATGCACGCTGGAGATTGACTGCTCCGACTTCCACGTGCAGGACTCAGAGGATTGCAGAAAAGACTACCTGATGATTAAGGAGAATAGCGAGACGAAGGAGAAATTCTGCGGTGACGAGGGATTTATATATGAGAGTAGCAAAAATGCCGTAACGCTCAAGTTCAAATCCAACCGGCGGGTAACGGACAAAGGATTCTCCTGCTTAGCTCGCTCCGTATGTCCAACCACCACCACCACCACCACTTCCACTGAGACCACGCCTACCACCATCACGACTACTGAGACCACGCCCACTACCACCACGACCATGACCACTGAGACCACGCCCATCACCACCACTACCGCTGAGACCACGCCCATCACCACCACTACCGCTGAGACCACGCCCATCACCACCACTACCACTGAGACCACGCCCATCACCACCACTACCGCTGAGACCACGCCCATCACCACCACTACCACTGAGACCACGCCCATCACCACCACTACCACTGAGACCACGCCCACCACCACCACGACCACTGAGACCACGCCCACCACCACCACTATCACGACTACTGAGACCACGCCCACCACCACCACCATCACTACCAGTGAGACCACGCCCACCACCACTACGGCCATAGACACTACGACCACCATCACGACCACTGAGGCCACGACCACCACGAGTTGCACAGGATCGTGTCCTTGCGGTCTTGCCAATCTAGCGAGGGTCGTGGGCGGGGAGGATGTAGATCCTGCCTACAAGTACCCGTGGCATGTGGGAATAAAGTATGTATGGAACAGAAAGTACTGGTGCGGCGGCTCCATCATTAACGACCGGTATATCCTGACAGCCGCCCATTGTGTTAAAAACAGATTTAGACGGTGGCTGGTGGTGGGCGTGGCCGACCACGACATGACGTCCACCGACGGGGTGACCCAACTGGTACGCGTTCAGGAGATTATCGTTCATCCTGACTATAATATCAACACCCTTGACAGTGATATCGCGCTGCTCAAGCTGTCACAACCACTGGACCTGACTCAAGTCGAGCACATTCGCCCCGTGTGTCTGCCCGCCGATGACTCCAACACCTATGCCGGCGAAGATGCCACAGCGACAGGCTGGGGCATACTTCAGTCCTGGGGAAGTCAACCAGCAATACTGCAGGAAGTCACAGTGCCTATTCTGGATCCCTCCTGCCCGGGACAGATATCGAATTATATCACTGAAAACATGCTGTGTGCTGGCCTTGAAGAAGGTGGCAAGGACACCTGCCAGGGCGACGCGGGCGGTCCGCTTACTGTGCAAAATGACTGTTCCAAATATGAGCAAGTTGGCATCACCTCCTGGGGCTTTGGATGTGCCGACCCTGGCAGGCCCGGTGTATACACCAGGGTGAGCAGATTCCTTAACTGGATCTACGCCAACACTGTTGACGCCACCTACTGCCAGTAG

METQWNVKEEICAVRIIAENRECTLEIDCSDFHVQDSEDCRKDYLMIKENSETKEKFCGDEGFIYESSKNAVTLKFKSNRRVTDKGFSCLARSVCPTTTTTTTSTETTPTTITTTETTPTTTTTMTTETTPITTTTAETTPITTTTAETTPITTTTTETTPITTTTAETTPITTTTTETTPITTTTTETTPTTTTTTETTPTTTTITTTETTPTTTTITTSETTPTTTTAIDTTTTITTTEATTTTSCTGSCPCGLANLARVVGGEDVDPAYKYPWHVGIKYVWNRKYWCGGSIINDRYILTAAHCVKNRFRRWLVVGVADHDMTSTDGVTQLVRVQEIIVHPDYNINTLDSDIALLKLSQPLDLTQVEHIRPVCLPADDSNTYAGEDATATGWGILQSWGSQPAILQEVTVPILDPSCPGQISNYITENMLCAGLEEGGKDTCQGDAGGPLTVQNDCSKYEQVGITSWGFGCADPGRPGVYTRVSRFLNWIYANTVDATYCQ

transcript_transcript/16534_Pt_Mix_transcript16534/f13p0/2150

ATGGACGTGAGGCTCTGTTGCCTCCTGCTGTTTGTCGCCGCATCCTGGATGCCCGGCCTGGCCAATGACTTTATGATAAGCCCCACTCACAGAGAGCTGCCTGCGCTTCACAAATCAGGACAAAACGAAAGGCGCCCCAAAGATGAAGTAGCCATTGTTGTGCCCAAAGCGAAAGAACAGCCGGTAGATGCCATCCAAAAAGGAACCATTGAGACTAAGCTGGGAGGACACCCCGTCGTTCACAAACAAATAGATATCTCTGGGAGGCCCAAGCAGGATGGAGACCCAGTGGAATGTGAAGGAAGAAAGGCAGTGGTGGTGGGCGAGGAGGGGCGCATCATCACCAGCCCGAATTATCCAGACAATTATTCCGACGAAGAAATATGTGCCGTGAGGATTATCGCCGAGAACAGGGAATGCACGCTGGAGATTGACTGCTCCGACTTCCACGTGCAGGACTCAGAGGATTGCAGAAAAGACTACCTGATGATTAAGGAGAATAGCGAGACGAAGGAGAAATTCTGCGGTGACGAGGGATTTATATATGAGAGTAGCAAAAATGCCGTAACGCTCAAGTTCAAATCCAACCGGCGGGTAACGGACAAAGGATTCTCCTGCTTAGCTCGCTCCGTATGTCCAACCACCACCACCACCACCACTTCCACTGAGACCACGCCTACCACCATCACGACTACTGAGACCACGCCCACTACCACCACGACCATGACCACTGAGACCACGCCCATCACCACCACTACCGCTGAGACCACGCCCATCACCACCACTACCGCTGAGACCACGCCCATCACCACCACTACCACTGAGACCACGCCCACCACCACCACGACCACTGAGACCACGCCCACCACCACCACTATCACGACTACTGAGACCACGCCCACCACCACCACCATCACTACCAGTGAGACCACGCCCACCACCACTACGGCCATAGACACTACGACCACCATCACGACCACTGAGGCCACGACCACCACGAGTTGCACAGGATCGTGTCCTTGCGGTCTTGCCAATCTAGCGAGGGTCGTGGGCGGGGAGGATGTAGATCCTGCCTACAAGTACCCGTGGCATGTGGGAATAAAGTATGTATGGAACAGAAAGTACTGGTGCGGCGGCTCCATCATTAACGACCGGTATATCCTGACAGCCGCCCATTGTGTTAAAAACAGATTTAGACGGTGGCTGGTGGTGGGCGTGGCCGACCACGACATGACGTCCACCGACGGGGTGACCCAACTGGTACGCGTTCAGGAGATTATCGTTCATCCTGACTATAATATCAACACCCTTGACAGTGATATCGCGCTGCTCAAGCTGTCACAACCACTGGACCTGACTCAAGTCGAGCACATTCGCCCCGTGTGTCTGCCCGCCGATGACTCCAACACCTATGCCGGCGAAGATGCCACAGCGACAGGCTGGGGCATACTTCAGTCCTGGGGAAGTCAACCAGCAATACTGCAGGAAGTCACAGTGCCTATTCTGGATCCCTCCTGCCCGGGACAGATATCGAATTATATCACTGAAAACATGCTGTGTGCTGGCCTTGAAGAAGGTGGCAAGGACACCTGCCAGGGCGACGCGGGCGGTCCGCTTACTGTGCAAAATGACTGTTCCAAATATGAGCAAGTTGGCATCACCTCCTGGGGCTTTGGATGTGCCGACCCTGGCAGGCCCGGTGTATACACCAGGGTGAGCAGATTCCTTAACTGGATCTACGCCAACACTGTTGACGCCACCTACTGCCAGTAG

MDVRLCCLLLFVAASWMPGLANDFMISPTHRELPALHKSGQNERRPKDEVAIVVPKAKEQPVDAIQKGTIETKLGGHPVVHKQIDISGRPKQDGDPVECEGRKAVVVGEEGRIITSPNYPDNYSDEEICAVRIIAENRECTLEIDCSDFHVQDSEDCRKDYLMIKENSETKEKFCGDEGFIYESSKNAVTLKFKSNRRVTDKGFSCLARSVCPTTTTTTTSTETTPTTITTTETTPTTTTTMTTETTPITTTTAETTPITTTTAETTPITTTTTETTPTTTTTTETTPTTTTITTTETTPTTTTITTSETTPTTTTAIDTTTTITTTEATTTTSCTGSCPCGLANLARVVGGEDVDPAYKYPWHVGIKYVWNRKYWCGGSIINDRYILTAAHCVKNRFRRWLVVGVADHDMTSTDGVTQLVRVQEIIVHPDYNINTLDSDIALLKLSQPLDLTQVEHIRPVCLPADDSNTYAGEDATATGWGILQSWGSQPAILQEVTVPILDPSCPGQISNYITENMLCAGLEEGGKDTCQGDAGGPLTVQNDCSKYEQVGITSWGFGCADPGRPGVYTRVSRFLNWIYANTVDATYCQ

transcript_transcript/17017_Pt_Mix_transcript17017/f11p0/2113

ATGGACGTGAGGCTCTGTTGCCTCCTGCTGTTTGTCGCCGCATCCTGGATGCCCGGCCTGGCCAATGACTTTATGATAAGCCCCACTCACAGAGAGCTGCCTGCGCTTCACAAATCAGGACAAAACGAAAGGCGCCCCAAAGATGAAGTAGCCATTGTTGTGCCCAAAGCGAAAGAACAGCCGGTAGATGCCATCCAAAAAGGAACCATTGAGACTAAGCTGGGAGGACACCCCGTCGTTCACAAACAAATAGATATCTCTGGGAGGCCCAAGCAGGATGGAGACCCAGTGGAATGTGAAGGAAGAAAGGCAGTGGTGGTGGGCGAGGAGGGGCGCATCATCACCAGCCCGAATTATCCAGACAATTATTCCGACGAAGAAATATGTGCCGTGAGGATTATCGCCGAGAACAGGGAATGCACGCTGGAGATTGACTGCTCCGACTTCCACGTGCAGGACTCAGAGGATTGCAGAAAAGACTACCTGATGATTAAGGAGAATAGCGAGACGAAGGAGAAATTCTGCGGTGACGAGGGATTTATATATGAGAGTAGCAAAAATGCCGTAACGCTCAAGTTCAAATCCAACCGGCGGGTAACGGACAAAGGATTCTCCTGCTTAGCTCGCTCCGTATGTCCAACCACCACCACCACCACCACTTCCACTGAGACCACGCCTACCACCATCACGACTACTGAGACCACGCCCATCACCACCACTACCGCTGAGACCACGCCCATCACCACCACTACCACTGAGACCACGCCCATCACCACCACTACCACTGAGACCACGCCCACCACCACCACGACCACTGAGACCACGCCCACCACCACCACTATCACGACTACTGAGACCACGCCCACCACCACCACCATCACTACCAGTGAGACCACGCCCACCACCACTACGGCCATAGACACTACGACCACCATCACGACCACTGAGGCCACGACCACCACGAGTTGCACAGGATCGTGTCCTTGCGGTCTTGCCAATCTAGCGAGGGTCGTGGGCGGGGAGGATGTAGATCCTGCCTACAAGTACCCGTGGCATGTGGGAATAAAGTATGTATGGAACAGAAAGTACTGGTGCGGCGGCTCCATCATTAACGACCGGTATATCCTGACAGCCGCCCATTGTGTTAAAAACAGATTTAGACGGTGGCTGGTGGTGGGCGTGGCCGACCACGACATGACGTCCACCGACGGGGTGACCCAACTGGTACGCGTTCAGGAGATTATCGTTCATCCTGACTATAATATCAACACCCTTGACAGTGATATCGCGCTGCTCAAGCTGTCACAACCACTGGACCTGACTCAAGTCGAGCACATTCGCCCCGTGTGTCTGCCCGCCGATGACTCCAACACCTATGCCGGCGAAGATGCCACAGCGACAGGCTGGGGCATACTTCAGTCCTGGGGAAGTCAACCAGCAATACTGCAGGAAGTCACAGTGCCTATTCTGGATCCCTCCTGCCCGGGACAGATATCGAATTATATCACTGAAAACATGCTGTGTGCTGGCCTTGAAGAAGGTGGCAAGGACACCTGCCAGGGCGACGCGGGCGGTCCGCTTACTGTGCAAAATGACTGTTCCAAATATGAGCAAGTTGGCATCACCTCCTGGGGCTTTGGATGTGCCGACCCTGGCAGGCCCGGTGTATACACCAGGGTGAGCAGATTCCTTAACTGGATCTACGCCAACACTGTTGACGCCACCTACTGCCAGTAG

MDVRLCCLLLFVAASWMPGLANDFMISPTHRELPALHKSGQNERRPKDEVAIVVPKAKEQPVDAIQKGTIETKLGGHPVVHKQIDISGRPKQDGDPVECEGRKAVVVGEEGRIITSPNYPDNYSDEEICAVRIIAENRECTLEIDCSDFHVQDSEDCRKDYLMIKENSETKEKFCGDEGFIYESSKNAVTLKFKSNRRVTDKGFSCLARSVCPTTTTTTTSTETTPTTITTTETTPITTTTAETTPITTTTTETTPITTTTTETTPTTTTTTETTPTTTTITTTETTPTTTTITTSETTPTTTTAIDTTTTITTTEATTTTSCTGSCPCGLANLARVVGGEDVDPAYKYPWHVGIKYVWNRKYWCGGSIINDRYILTAAHCVKNRFRRWLVVGVADHDMTSTDGVTQLVRVQEIIVHPDYNINTLDSDIALLKLSQPLDLTQVEHIRPVCLPADDSNTYAGEDATATGWGILQSWGSQPAILQEVTVPILDPSCPGQISNYITENMLCAGLEEGGKDTCQGDAGGPLTVQNDCSKYEQVGITSWGFGCADPGRPGVYTRVSRFLNWIYANTVDATYCQ

transcript_transcript/17309_Pt_Mix_transcript17309/f6p0/2087

ATGGACGTGAGGCTCTGTTGCCTCCTGCTGTTTGTCGCCGCATCCTGGATGCCCGGCCTGGCCAATGACTTTATGATAAGCCCCACTCACAGAGAGCTGCCTGCGCTTCACAAATCAGGACAAAACGAAAGGCGCCCCAAAGATGAAGTAGCCATTGTTGTGCCCAAAGCGAAAGAACAGCCGGTAGATGCCATCCAAAAAGGAACCATTGAGACTAAGCTGGGAGGACACCCCGTCGTTCACAAACAAATAGATATCTCTGGGAGGCCCAAGCAGGATGGAGACCCAGTGGAATGTGAAGGAAGAAAGGCAGTGGTGGTGGGCGAGGAGGGGCGCATCATCACCAGCCCGAATTATCCAGACAATTATTCCGACGAAGAAATATGTGCCGTGAGGATTATCGCCGAGAACAGGGAATGCACGCTGGAGATTGACTGCTCCGACTTCCACGTGCAGGACTCAGAGGATTGCAGAAAAGACTACCTGATGATTAAGGAGAATAGCGAGACGAAGGAGAAATTCTGCGGTGACGAGGGATTTATATATGAGAGTAGCAAAAATGCCGTAACGCTCAAGTTCAAATCCAACCGGCGGGTAACGGACAAAGGATTCTCCTGCTTAGCTCGCTCCGTATGTCCAACCACCACCACCACCACCACTTCCACTGAGACCACGCCTACCACCATCACGACTACTGAGACCACGCCCACTACCACCACGACCATGACCACTGAGACCACGCCCATCACCACCACTACCACTGAGACCACGCCCACCACCACCACGACCACTGAGACCACGCCCACCACCACCACTATCACGACTACTGAGACCACGCCCACCACCACCACCATCACTACCAGTGAGACCACGCCCACCACCACTACGGCCATAGACACTACGACCACCATCACGACCACTGAGGCCACGACCACCACGAGTTGCACAGGATCGTGTCCTTGCGGTCTTGCCAATCTAGCGAGGGTCGTGGGCGGGGAGGATGTAGATCCTGCCTACAAGTACCCGTGGCATGTGGGAATAAAGTATGTATGGAACAGAAAGTACTGGTGCGGCGGCTCCATCATTAACGACCGGTATATCCTGACAGCCGCCCATTGTGTTAAAAACAGATTTAGACGGTGGCTGGTGGTGGGCGTGGCCGACCACGACATGACGTCCACCGACGGGGTGACCCAACTGGTACGCGTTCAGGAGATTATCGTTCATCCTGACTATAATATCAACACCCTTGACAGTGATATCGCGCTGCTCAAGCTGTCACAACCACTGGACCTGACTCAAGTCGAGCACATTCGCCCCGTGTGTCTGCCCGCCGATGACTCCAACACCTATGCCGGCGAAGATGCCACAGCGACAGGCTGGGGCATACTTCAGTCCTGGGGAAGTCAACCAGCAATACTGCAGGAAGTCACAGTGCCTATTCTGGATCCCTCCTGCCCGGGACAGATATCGAATTATATCACTGAAAACATGCTGTGTGCTGGCCTTGAAGAAGGTGGCAAGGACACCTGCCAGGGCGACGCGGGCGGTCCGCTTACTGTGCAAAATGACTGTTCCAAATATGAGCAAGTTGGCATCACCTCCTGGGGCTTTGGATGTGCCGACCCTGGCAGGCCCGGTGTATACACCAGGGTGAGCAGATTCCTTAACTGGATCTACGCCAACACTGTTGACGCCACCTACTGCCAGTAG

MDVRLCCLLLFVAASWMPGLANDFMISPTHRELPALHKSGQNERRPKDEVAIVVPKAKEQPVDAIQKGTIETKLGGHPVVHKQIDISGRPKQDGDPVECEGRKAVVVGEEGRIITSPNYPDNYSDEEICAVRIIAENRECTLEIDCSDFHVQDSEDCRKDYLMIKENSETKEKFCGDEGFIYESSKNAVTLKFKSNRRVTDKGFSCLARSVCPTTTTTTTSTETTPTTITTTETTPTTTTTMTTETTPITTTTTETTPTTTTTTETTPTTTTITTTETTPTTTTITTSETTPTTTTAIDTTTTITTTEATTTTSCTGSCPCGLANLARVVGGEDVDPAYKYPWHVGIKYVWNRKYWCGGSIINDRYILTAAHCVKNRFRRWLVVGVADHDMTSTDGVTQLVRVQEIIVHPDYNINTLDSDIALLKLSQPLDLTQVEHIRPVCLPADDSNTYAGEDATATGWGILQSWGSQPAILQEVTVPILDPSCPGQISNYITENMLCAGLEEGGKDTCQGDAGGPLTVQNDCSKYEQVGITSWGFGCADPGRPGVYTRVSRFLNWIYANTVDATYCQ

transcript_transcript/17417_Pt_Mix_transcript17417/f2p0/2094

ATGGACGTGAGGCTCTGTTGCCTCCTGCTGTTTGTCGCCGCATCCTGGATGCCCGGCCTGGCCAATGACTTTATGATAAGCCCCACTCACAGAGAGCTGCCTGCGCTTCACAAATCAGGACAAAACGAAAGGCGCCCCAAAGATGAAGTAGCCATTGTTGTGCCCAAAGCGAAAGAACAGCCGGTAGATGCCATCCAAAAAGGAACCATTGAGACTAAGCTGGGAGGACACCCCGTCGTTCACAAACAAATAGATATCTCTGGGAGGCCCAAGCAGGATGGAGACCCAGTGGAATGTGAAGGAAGAAAGGCAGTGGTGGTGGGCGAGGAGGGGCGCATCATCACCAGCCCGAATTATCCAGACAATTATTCCGACGAAGAAATATGTGCCGTGAGGATTATCGCCGAGAACAGGGAATGCACGCTGGAGATTGACTGCTCCGACTTCCACGTGCAGGACTCAGAGGATTGCAGAAAAGACTACCTGATGATTAAGGAGAATAGCGAGACGAAGGAGAAATTCTGCGGTGACGAGGGATTTATATATGAGAGTAGCAAAAATGCCGTAACGCTCAAGTTCAAATCCAACCGGCGGGTAACGGACAAAGGATTCTCCTGCTTAGCTCGCTCCGTATGTCCAACCACCACCACCACCACCACTTCCACTGAGACCACGCCTACCACCATCACGACTACTGAGACCACGCCCACTACCACCACGACCATGACCACTGAGACCACGCCCATCACCACCACTACCGCTGAGACCACGCCCATCACCACCACTACCGCTGAGACCACGCCCATCACCACCACTACCACTGAGACCACGCCCATCACCACCACTACCGCTGAGACCACGCCCATCACCACCACTACCACTGAGACCACGCCCATCACCACCACTACCACTGAGACCACGCCCACCACCACCACGACCACTGAGACCACGCCCACCACCACCACTATCACGACTACTGAGACCACGCCCACCACCACCACCATCACTACCAGTGAGACCACGCCCACCACCACTACGGCCATAGACACTACGACCACCATCACGACCACTGAGGCCACGACCACCACGAGTTGCACAGGATCGTGTCCTTGCGGTCTTGCCAATCTAGCGAGGGTCGTGGGCGGGGAGGATGTAGATCCTGCCTACAAGTACCCGTGGCATGTGGGAATAAAGTATGTATGGAACAGAAAGTACTGGTGCGGCGGCTCCATCATTAACGACCGGTATATCCTGACAGCCGCCCATTGTGTTAAAAACAGATTTAGACGGTGGCTGGTGGTGGGCGTGGCCGACCACGACATGACGTCCACCGACGGGGTGACCCAACTGGTACGCGTTCAGGAGATTATCGTTCATCCTGACTATAATATCAACACCCTTGACAGTGATATCGCGCTGCTCAAGCTGTCACAACCACTGGACCTGACTCAAGTCGAGCACATTCGCCCCGTGTGTCTGCCCGCCGATGACTCCAACACCTATGCCGGCGAAGATGCCACAGCGACAGGCTGGGGCATACTTCAGTCCTGGGGAAGTCAACCAGCAATACTGCAGGAAGTCACAGTGCCTATTCTGGATCCCTCCTGCCCGGGACAGATATCGAATTATATCACTGAAAACATGCTGTGTGCTGGCCTTGAAGAAGGTGGCAAGGACACCTGCCAGGGCGACGCGGGCGGTCCGCTTACTGTGCAAAATGACTGTTCCAAATATGAGCAAGTTGGCATCACCTCCTGGGGCTTTGGATGTGCCGACCCTGGCAGGCCCGGTGTATACACCAGGGTGAGCAGATTCCTTAACTGGATCTACGCCAACATTGCTGACGCTGCCAGTAGTAGTGCCAACACTGCTGACACCACCTACTGCCAGTAG

MDVRLCCLLLFVAASWMPGLANDFMISPTHRELPALHKSGQNERRPKDEVAIVVPKAKEQPVDAIQKGTIETKLGGHPVVHKQIDISGRPKQDGDPVECEGRKAVVVGEEGRIITSPNYPDNYSDEEICAVRIIAENRECTLEIDCSDFHVQDSEDCRKDYLMIKENSETKEKFCGDEGFIYESSKNAVTLKFKSNRRVTDKGFSCLARSVCPTTTTTTTSTETTPTTITTTETTPTTTTTMTTETTPITTTTAETTPITTTTAETTPITTTTTETTPITTTTAETTPITTTTTETTPITTTTTETTPTTTTTTETTPTTTTITTTETTPTTTTITTSETTPTTTTAIDTTTTITTTEATTTTSCTGSCPCGLANLARVVGGEDVDPAYKYPWHVGIKYVWNRKYWCGGSIINDRYILTAAHCVKNRFRRWLVVGVADHDMTSTDGVTQLVRVQEIIVHPDYNINTLDSDIALLKLSQPLDLTQVEHIRPVCLPADDSNTYAGEDATATGWGILQSWGSQPAILQEVTVPILDPSCPGQISNYITENMLCAGLEEGGKDTCQGDAGGPLTVQNDCSKYEQVGITSWGFGCADPGRPGVYTRVSRFLNWIYANIADAASSSANTADTTYCQ

transcript_transcript/17579_Pt_Mix_transcript17579/f6p0/2080

ATGGACGTGAGGGTCTGTTGCCTCCTGCTGTTTGTCGCCGCATCCTGGGTGCCCGGCCTGGCCAATGACTTTGTGATAAGCCCTACCCACGGAGAGCTCCCAGCGCTTCACAAACGAGAACAAAACGCCAGGGGCTCAAGAGATGAAGAAACCATTCAGCCCAAGGCGGGAGAACAGCCGGTAAATGCCATTCATGAAGAAACCATTGAGACTAAGCTGGGAGGTAACCCCGTGGTTCACAAACAACCAGGAATCTTTGGAAGGCCCAAGCAGGATGGAGACCCCGTGGAATGTGAAGGAAAAAAAAGAGTGGTGGTGGGCGAGGAGGGGCGCATCATTACCAGCCCGAATTATCCAGACAATTATCCCAACCGAGAAGAATGTGCCGTGAGGATTGTCGCCGAGAACAGGGAATGCACGCTGGAGATTGACTGCTATGACTTCCACGTGCAGGACTCAGAGGATTGCAGAAAAGACTACCTGATGATTAAGGAGAATAAGTGGACGAAGGAGAAATTCTGCGGTGACGAGGGATTTATTTACGAGAGTAGCAAAAGAGCCGTAACACTCAAGTTCAAATCCAACCGGCGGGTAACGGACGAAGGATTCTCCTGCTTAGCTCGCTCCGTATGTCCAACCACCACCACCACCACCACCACCGAGACCACGCCCACCACCACCACGACCACTGAAACCACCACGCCCACCACCACCACTGAAACCACCACGCCCACCACCACCATCACGACCACTGAAACCACCACGCCCACCACCACCACCACCACGACCACTGAGGTCACGACCACCACGAGTAGCACAGGATCGTGTCAGTGCGGTGTTGCCAATCTAGCGAGGATCGTGGGCGGGGAGGAGGTAGATCCTGCCCACAAGTACCCGTGGCATGTGGGAATAAAGTATGTATGGAACAGTAATTACTGGTGCGGCGGCTCCATCATTAACGACCGGTATATCCTGACAGCCGCCCACTGTGTTGACGACATAACATCTGTACAAGGGCTGGTGGTGGGCGTGGCTGACCACGATATGACGTCCACCGACGATGTCACCCAACTGGTACCCGTTCAGGAGATTATCGTTCATCCTGACTATAATTCCAACACCCTTGACAGTGATATCGCGCTGCTCAAGCTGTCACAACCACTGGACCTGACTCAAGTCGAGCACATTCGCCCCGTGTGTTTGCCCGCCGATGACTCCAACACCTATGCCGGCGAAGATGCCACAGCGACAGGCTGGGGCACACTTCAGTCCGGGGGAAGTCAACCAGCAATACTGCAGGAAGTCACAGTGCCTATTCTGGATCCCTCCTGCCCGGGACACAAACCGAGTCATATCACTGAAAACATGCTGTGTGCTGGCCTTGAAGAAGGTGGCAAGGACACCTGCCAGGGCGACTCGGGCGGTCCGCTTACCGTGCAAAATGACTCTTCCAAGTATGAGCAAATTGGCATCACCTCCTGGGGCTTTGGATGTGCCGACCCTGGCAGCCCCGGTGTGTACACCAGGGTGAGCAACTTCCTTGAGTGGATAGAGGAGAACACTACTGACGCCACCTACTGCCACTAG

MDVRVCCLLLFVAASWVPGLANDFVISPTHGELPALHKREQNARGSRDEETIQPKAGEQPVNAIHEETIETKLGGNPVVHKQPGIFGRPKQDGDPVECEGKKRVVVGEEGRIITSPNYPDNYPNREECAVRIVAENRECTLEIDCYDFHVQDSEDCRKDYLMIKENKWTKEKFCGDEGFIYESSKRAVTLKFKSNRRVTDEGFSCLARSVCPTTTTTTTTETTPTTTTTTETTTPTTTTETTTPTTTITTTETTTPTTTTTTTTEVTTTTSSTGSCQCGVANLARIVGGEEVDPAHKYPWHVGIKYVWNSNYWCGGSIINDRYILTAAHCVDDITSVQGLVVGVADHDMTSTDDVTQLVPVQEIIVHPDYNSNTLDSDIALLKLSQPLDLTQVEHIRPVCLPADDSNTYAGEDATATGWGTLQSGGSQPAILQEVTVPILDPSCPGHKPSHITENMLCAGLEEGGKDTCQGDSGGPLTVQNDSSKYEQIGITSWGFGCADPGSPGVYTRVSNFLEWIEENTTDATYCH

transcript_transcript/18141_Pt_Mix_transcript18141/f3p0/2041

ATGGACGTGAGGCTCTGTTGCCTCCTGGAGGTGGGCGAGGAGGGGCGCATCATCACCAGCCCGAATTATCCAGACAATTATTCCGACGAAGAAAAATGTGCCGTGAGGATTATCGCCGAGAACAGGGAATGCACGCTGGAGATTGACTGCTATGACTTCCACGTGCAGGACTCAGAGGATTGCAGAAAAGACTACCTGATGATTAAGGAGAATAGCGAGACGAAGGAGAAATTCTGCGGTGACGAGGGATTTATATATGAGAGTAGCAAAAATGCCGTAACGCTCAAGTTCAGATCCATCCGGCGGGTAACGGACAAAGGATTCTCCTGCTTAGCTCGCTCCGTATGTCCAACCACCACCACCACCACCACTTCCACTGAGACCACGCCTACCACCATCACGACTACTGAGACCACGCCCACTACCACCACGACCATGACCACTGAGACCACGCCCATCACCACCACTACCGCTGAGACCACGCCCATCACCACCACTACCGCTGAGACCACGCCCATCACCACCACTACCACTGAGACCACGCCCATCACCACCACTACCGCTGAGACCACGCCCATCACCACCACTACCACTGAGACCACGCCCATCACCACCACTACCACTGAGACCACGCCCACCACCACCACGACCACTGAGACCACGCCCACCACCACCACTATCACGACTACTGAGACCACGCCCACCACCACCACCATCACTACCACTGAGACCACGCCCACCACCACTACGGCCATAGACACTACGACCACCATCACGACCACTGAGGCCACGACCACCACGAGTTGCACAGGATCGTGTCCTTGCGGTCTTGCCAATCTAGCGAGGGTTGTGGGCGGGGAGGATGTAGATCCTGCCTACAAGTACCCGTGGCATGTGGGAATAAAGTATGTATGGAACAGAAAGTACTGGTGCGGCGGCTCCATCATTAACGACCGGTATATCCTGACAGCCGCCCATTGTGTTAAAAACAGATTTACGAGTAGACGGTGGCTGGTGGTGGGCGTGGCCGACCACGACATGACGTCCACCAACGGGGTGACCCAACTGGTACGCGTTCAGGAGATTATCGTTCATCCTGACTATAATATCAACACCCTTGACAGTGATATCGCGCTGCTCAAGCTGTCACAACCACTGGACCTGACTCAAGTCGAGCACATTCGCCCCGTGTGTCTGCCCGCCGATGACTCCAACACCTATGCCGGCGAAGATGCCACAGCGACAGGCTGGGGCATACTTCAGTCCTGGGGAAGTCAACCAGCAATACTGCAGGAAGTCACAGTGCCTATTCTGGATCCCTCCTGCCCGGGACAGATATCGAATTATATCACTGAAAACATGCTGTGTGCTGGCCTTGAAGAAGGTGGCAAGGACACCTGCCAGGGCGACGCGGGCGGTCCGCTTACTGTGCAAAATGACTGTTCCAAGTATGAGCAAGTTGGCATCACCTCCTGGGGCTTTGGATGTGCCGACCCTGGCAGGCCCGGTGTATACACCAGGGTGAGCAGATTCCTTAACTGGATCTACGCCAACACTGTTGACGCCACCTACTGCCAGTAG

MDVRLCCLLEVGEEGRIITSPNYPDNYSDEEKCAVRIIAENRECTLEIDCYDFHVQDSEDCRKDYLMIKENSETKEKFCGDEGFIYESSKNAVTLKFRSIRRVTDKGFSCLARSVCPTTTTTTTSTETTPTTITTTETTPTTTTTMTTETTPITTTTAETTPITTTTAETTPITTTTTETTPITTTTAETTPITTTTTETTPITTTTTETTPTTTTTTETTPTTTTITTTETTPTTTTITTTETTPTTTTAIDTTTTITTTEATTTTSCTGSCPCGLANLARVVGGEDVDPAYKYPWHVGIKYVWNRKYWCGGSIINDRYILTAAHCVKNRFTSRRWLVVGVADHDMTSTNGVTQLVRVQEIIVHPDYNINTLDSDIALLKLSQPLDLTQVEHIRPVCLPADDSNTYAGEDATATGWGILQSWGSQPAILQEVTVPILDPSCPGQISNYITENMLCAGLEEGGKDTCQGDAGGPLTVQNDCSKYEQVGITSWGFGCADPGRPGVYTRVSRFLNWIYANTVDATYCQ

transcript_transcript/18866_Pt_Mix_transcript18866/f8p0/1889

ATGGACGTGAGGCTCTGTTGCCTCCTGCTGTTTGTCGCCGCATCCTGGATGCCCGGCCTGGCCAATGACTTTATGATAAGCCCCACTCACAGAGAGCTGCCTGCGCTTCACAAATCAGGACAAAACGAAAGGCGCCCCAAAGATGAAGTAGCCATTGTTGTGCCCAAAGCGAAAGAACAGCCGGTAGATGCCATCCAAAAAGGAACCATTGAGACTAAGCTGGGAGGACACCCCGTCGTTCACAAACAAATAGATATCTCTGGGAGGCCCAAGCAGGATGGAGACCCAGTGGAATGTGAAGGAAGAAAGGCAGTGGTGGTGGGCGAGGAGGGGCGCATCATCACCAGCCCGAATTATCCAGACAATTATTCCGACGAAGAAATATGTGCCGTGAGGATTATCGCCGAGAACAGGGAATGCACGCTGGAGATTGACTGCTCCGACTTCCACGTGCAGGACTCAGAGGATTGCAGAAAAGACTACCTGATGATTAAGGAGAATAGCGAGACGAAGGAGAAATTCTGCGGTGACGAGGGATTTATATATGAGAGTAGCAAAAATGCCGTAACGCTCAAGTTCAAATCCAACCGGCGGGTAACGGACAAAGGATTCTCCTGCTTAGCTCGCTCCGTATGTCCAACCACCACCACCACCACCACTTCCACTGAGACCACGCCTACCACCATCACGACTACTGAGACCACGCCCACTACCACCACGACCATGACCACTGAGACCACGCCCATCACCACCACTACCGCTGAGACCACGCCCATCACCACCACTACCGCTGAGACCACGCCCATCACCACCACTACCACTGAGACCACGCCCATCACCACCACTACCGCTGAGACCACGCCCATCACCACCACTACCACTGAGACCACGCCCATCACCACCACTACCACTGAGACCACGCCCACCACCACCACGACCACTGAGACCACGCCCACCACCACCACTATCATGACGTCCACCGACGGGGTGACCCAACTGGTACGCGTTCAGGAGATTATCGTTCATCCTGACTATAATATCAACACCCTTGACAGTGATATCGCGCTGCTCAAGCTGTCACAACCACTGGACCTGACTCAAGTCGAGCACATTCGCCCCGTGTGTCTGCCCGCCGATGACTCCAACACCTATGCCGGCGAAGATGCCACAGCGACAGGCTGGGGCATACTTCAGTCCTGGGGAAGTCAACCAGCAATACTGCAGGAAGTCACAGTGCCTATTCTGGATCCCTCCTGCCCGGGACAGATATCGAATTATATCACTGAAAACATGCTGTGTGCTGGCCTTGAAGAAGGTGGCAAGGACACCTGCCAGGGCGACGCGGGCGGTCCGCTTACTGTGCAAAATGACTGTTCCAAATATGAGCAAGTTGGCATCACCTCCTGGGGCTTTGGATGTGCCGACCCTGGCAGGCCCGGTGTATACACCAGGGTGAGCAGATTCCTTAACTGGATCTACGCCAACACTGTTGACGCCACCTACTGCCAGTAG

MDVRLCCLLLFVAASWMPGLANDFMISPTHRELPALHKSGQNERRPKDEVAIVVPKAKEQPVDAIQKGTIETKLGGHPVVHKQIDISGRPKQDGDPVECEGRKAVVVGEEGRIITSPNYPDNYSDEEICAVRIIAENRECTLEIDCSDFHVQDSEDCRKDYLMIKENSETKEKFCGDEGFIYESSKNAVTLKFKSNRRVTDKGFSCLARSVCPTTTTTTTSTETTPTTITTTETTPTTTTTMTTETTPITTTTAETTPITTTTAETTPITTTTTETTPITTTTAETTPITTTTTETTPITTTTTETTPTTTTTTETTPTTTTIMTSTDGVTQLVRVQEIIVHPDYNINTLDSDIALLKLSQPLDLTQVEHIRPVCLPADDSNTYAGEDATATGWGILQSWGSQPAILQEVTVPILDPSCPGQISNYITENMLCAGLEEGGKDTCQGDAGGPLTVQNDCSKYEQVGITSWGFGCADPGRPGVYTRVSRFLNWIYANTVDATYCQ

transcript_transcript/20658_Pt_Mix_transcript20658/f8p0/1860

ATGGACGTGAGGCTCTGTTGCCTCCTGCTGTTTGTCGCCGCATCCTGGATGCCCGGCCTGGCCAATGACTTTATGATAAGCCCCACTCACAGAGAGCTGCCTGCGCTTCACAAATCAGGACAAAACGAAAGGCGCCCCAAAGATGAAGTAGCCATTGTTGTGCCCAAAGCGAAAGAACAGCCGGTAGATGCCATCCAAAAAGGAACCATTGAGACTAAGCTGGGAGGACACCCCGTCGTTCACAAACAAATAGATATCTCTGGGAGGCCCAAGCAGGATGGAGACCCAGTGGAATGTGAAGGAAGAAAGGCAGTGGTGGTGGGCGAGGAGGGGCGCATCATCACCAGCCCGAATTATCCAGACAATTATTCCGACGAAGAAATATGTGCCGTGAGGATTATCGCCGAGAACAGGGAATGCACGCTGGAGATTGACTGCTCCGACTTCCACGTGCAGGACTCAGAGGATTGCAGAAAAGACTACCTGATGATTAAGGAGAATAGCGAGACGAAGGAGAAATTCTGCGGTGACGAGGGATTTATATATGAGAGTAGCAAAAATGCCGTAACGCTCAAGTTCAAATCCAACCGGCGGGTAACGGACAAAGGATTCTCCTGCTTAGCTCGCTCCGTATGTCCAACCACCACCACCACCACCACTTCCACTGAGACCACGCCTACCACCATCACGACTACTGAGACCACGCCCACTACCACCACGACCATGACCACTGAGACCACGCCCATCACCACCACTACCGCTGAGACCACGCCCATCACCACCACTACCGCTGAGACCACGCCCATCACCACCACTACCACTGAGACCACGCCCATCACCACCACTACCGCTGAGACCACGCCCATCACCACCACTACCACTGAGACCACGCCCATCACCACCACTACCACTGAGACCACGCCCACCACCACCACGACCACTGACCCGTCCACCGACGGGGTGACCCAACTGGTACGCGTTCAGGAGATTATCGTTCATCCTGACTATAATATCAACACCCTTGACAGTGATATCGCGCTGCTCAAGCTGTCACAACCACTGGACCTGACTCAAGTCGAGCACATTCGCCCCGTGTGTCTGCCCGCCGATGACTCCAACACCTATGCCGGCGAAGATGCCACAGCGACAGGCTGGGGCATACTTCAGTCCTGGGGAAGTCAACCAGCAATACTGCAGGAAGTCACAGTGCCTATTCTGGATCCCTCCTGCCCGGGACAGATATCGAATTATATCACTGAAAACATGCTGTGTGCTGGCCTTGAAGAAGGTGGCAAGGACACCTGCCAGGGCGACGCGGGCGGTCCGCTTACTGTGCAAAATGACTGTTCCAAATATGAGCAAGTTGGCATCACCTCCTGGGGCTTTGGATGTGCCGACCCTGGCAGGCCCGGTGTATACACCAGGGTGAGCAGATTCCTTAACTGGATCTACGCCAACACTGTTGACGCCACCTACTGCCAGTAG

MDVRLCCLLLFVAASWMPGLANDFMISPTHRELPALHKSGQNERRPKDEVAIVVPKAKEQPVDAIQKGTIETKLGGHPVVHKQIDISGRPKQDGDPVECEGRKAVVVGEEGRIITSPNYPDNYSDEEICAVRIIAENRECTLEIDCSDFHVQDSEDCRKDYLMIKENSETKEKFCGDEGFIYESSKNAVTLKFKSNRRVTDKGFSCLARSVCPTTTTTTTSTETTPTTITTTETTPTTTTTMTTETTPITTTTAETTPITTTTAETTPITTTTTETTPITTTTAETTPITTTTTETTPITTTTTETTPTTTTTTDPSTDGVTQLVRVQEIIVHPDYNINTLDSDIALLKLSQPLDLTQVEHIRPVCLPADDSNTYAGEDATATGWGILQSWGSQPAILQEVTVPILDPSCPGQISNYITENMLCAGLEEGGKDTCQGDAGGPLTVQNDCSKYEQVGITSWGFGCADPGRPGVYTRVSRFLNWIYANTVDATYCQ

transcript_transcript/23871_Pt_Mix_transcript23871/f2p0/1639

ATGACCACTGAGACCACGCCCATCACCACCACTACCGCTGAGACCACGCCCATCACCACCACTACCGCTGAGACCACGCCCATCACCACCACTACCACTGAGACCACGCCCATCACCACCACTACCGCTGAGACCACGCCCATCACCACCACTACCACTGAGACCACGCCCATCACCACCACTACCACTGAGACCACGCCCACCACCACCACGACCACTGAGACCACGCCCACCACCACCACTATCACGACTACTGAGACCACGCCCACCACCACCACCATCACTACCAGTGAGACCACGCCCACCACCACTACGGCCATAGACACTACGACCACCATCACGACCACTGAGGCCACGACCACCACGAGTTGCACAGGATCGTGTCCTTGCGGTCTTGCCAATCTAGCGAGGGTCGTGGGCGGGGAGGATGTAGATCCTGCCTACAAGTACCCGTGGCATGTGGGAATAAAGTATGTATGGAACAGAAAGTACTGGTGCGGCGGCTCCATCATTAACGACCGGTATATCCTGACAGCCGCCCATTGTGTTAAAAACAGATTTAGACGGTGGCTGGTGGTGGGCGTGGCCGACCACGACATGACGTCCACCGACGGGGTGACCCAACTGGTACGCGTTCAGGAGATTATCGTTCATCCTGACTATAATATCAACACCCTTGACAGTGATATCGCGCTGCTCAAGCTGTCACAACCACTGGACCTGACTCAAGTCGAGCACATTCGCCCCGTGTGTCTGCCCGCCGATGACTCCAACACCTATGCCGGCGAAGATGCCACAGCGACAGGCTGGGGCATACTTCAGTCCTGGGGAAGTCAACCAGCAATACTGCAGGAAGTCACAGTGCCTATTCTGGATCCCTCCTGCCCGGGACAGATATCGAATTATATCACTGAAAACATGCTGTGTGCTGGCCTTGAAGAAGGTGGCAAGGACACCTGCCAGGGCGACGCGGGCGGTCCGCTTACTGTGCAAAATGACTGTTCCAAATATGAGCAAGTTGGCATCACCTCCTGGGGCTTTGGATGTGCCGACCCTGGCAGGCCCGGTGTATACACCAGGGTGAGCAGATTCCTTAACTGGATCTACGCCAACACTGTTGACGCCACCTACTGCCAGTAG

MTTETTPITTTTAETTPITTTTAETTPITTTTTETTPITTTTAETTPITTTTTETTPITTTTTETTPTTTTTTETTPTTTTITTTETTPTTTTITTSETTPTTTTAIDTTTTITTTEATTTTSCTGSCPCGLANLARVVGGEDVDPAYKYPWHVGIKYVWNRKYWCGGSIINDRYILTAAHCVKNRFRRWLVVGVADHDMTSTDGVTQLVRVQEIIVHPDYNINTLDSDIALLKLSQPLDLTQVEHIRPVCLPADDSNTYAGEDATATGWGILQSWGSQPAILQEVTVPILDPSCPGQISNYITENMLCAGLEEGGKDTCQGDAGGPLTVQNDCSKYEQVGITSWGFGCADPGRPGVYTRVSRFLNWIYANTVDATYCQ

transcript_transcript/26660_Pt_Mix_transcript26660/f10p0/1457

ATGATGTGTCACGTGAGTGTGGCGATGGTGACGGTAGCGACGCTAGCGGCATTAATGGGCTGTCAGGCCCAGGAGGGCGGAAAGTGCGTGGACGGGAACGGCCAGGCTGGAACCTGCATCAACATCCGCTCATGCCAGCCGCAAAGAGAACTCCTGCAGGCGGTCAGGGAAAATAGAGCGCCTCCTAACGGCCTCAGGATTTTGCGGCAGTCTGTGTGTGAAATCCAGAGCAATGGAAGACTACTGGTGTGCTGCGCCCAGAGCACAAGCACAGGGACAACTACAAGTGCAAGTACAGGTAGTGGCCGTGACCTGTTGCCCAAAAATTGTGGAAGGGTTGATCTGACCGACCGTATCATCGATGGAGAAGACTCTCCGCTGCTCGCCTGGCCCTGGATGGCGCTTCTCAGGGGCACAGGTCGAGGACAGCCGGCTAGCTGGTTCTGCGGTGGAGTCCTCATCAACGATCGCTATGTGCTCACTGCGGCCCACTGCTTCAATGAACACGAGTTGGAGTTTGTGAGGCTGGGCGAACACACACTCTCCACAGTAGAGGACTGCCAATCAGGTGTGTGCGCCCCGCCCCCTCAGGATATCACGGCAGAGCAGATCATCATTCATCCGCAGTATAAATCATCGTGTCGGGAGTGTAACGATATCGCTCTCCTCAGGCTGTCTACTCCCGTCCAGCTGCACCCTGTTCACGTGTTGCCAATCTGCGTCCCTGTCGACGTAGTGAGGGACATGGGGTTCTCGGAGGCAGACTTCCAGGGAAAACGCGCCTGGGCCGCCGGTTGGGGCTCCACCTCCAGGAGTCCCTTGAGGGTGACAACGCCTGACACGCTGCAGCAGGTGTTTCTCCCCATCCGAGAAGACGCAGTCTGCCCGCTGCTAAAGAGAAATTACCCCGACCCCCGCATGGTACTGTGTGCTGGAGGGGACGGCAAGGACACGTGCAGGGGAGACTCAGGCGGCCCACTCCAGCTGAGCAACAGGGCCGAAACAAGAAGGTTCGTGGTTGGCATTACCAGCGTTGGTCCCGAGGTGTGCGGTAGACAGAACACCCAGGCACTGTACACCAATGTTCACTTCTACGTTCAGTGGATCCTGGAAACTCTACGACCTTGA

MMCHVSVAMVTVATLAALMGCQAQEGGKCVDGNGQAGTCINIRSCQPQRELLQAVRENRAPPNGLRILRQSVCEIQSNGRLLVCCAQSTSTGTTTSASTGSGRDLLPKNCGRVDLTDRIIDGEDSPLLAWPWMALLRGTGRGQPASWFCGGVLINDRYVLTAAHCFNEHELEFVRLGEHTLSTVEDCQSGVCAPPPQDITAEQIIIHPQYKSSCRECNDIALLRLSTPVQLHPVHVLPICVPVDVVRDMGFSEADFQGKRAWAAGWGSTSRSPLRVTTPDTLQQVFLPIREDAVCPLLKRNYPDPRMVLCAGGDGKDTCRGDSGGPLQLSNRAETRRFVVGITSVGPEVCGRQNTQALYTNVHFYVQWILETLRP

transcript_transcript/26912_Pt_Mix_transcript26912/f6p0/1445

ATGAAGGCCTGCAGTGTGTTGGTGTTGGTCGCCGTGGCCTGCGTGCCCGCCCTGGCCCAGGAAGGCGGAGAATATGTGGACCCCAAGCAGGGAGAATATGTGGACCCCAAGCAGTCATGTGGAGGAACAATGACTGTGGGCGCGGAGCCGGTGGTGATCTCCAGCCCTAACCACCCCAACGACTACGCCAACAGGCAGAGGTGCAAGTGGAGGTTTACGGCGGAGGACCCTGACGACGCACTGTTCCTCAGCTGCGAGGAGTTCAGTGTGCTCTGCAAGGGAGACAGGCTGAACATCATCGAGGGAGGCGTTGTGCAGGAGAGGCTGTGTGGCAGTGACCCGGTGGAGTACGCCTCTGAAGGAAACGTCATGGTACTCAAGTTCCGCACCAACAGGCGTGGCACCAGTTCAGGCTTCTCCTGCACGGTGACCTCCTCCTCCTACTACGAGGGCTCCTCCTCCTCCTCTTCTTCCTCCACCGAGGAGGGCGAGGAGAACACCACGCCCTCCAGCAACACTGGCTCCTGCAAGTGCGGCGTGCCTAACCTCAACAGGATCGTGGGCGGTCAGGAGGTGAATCCCAAGAACAAGTACCCGTGGCAGGTGGGACTCAAGATGAGTAACGGCCGCAACTACTGGTGCGGCGGCTCCATCATCAACGACAGGTACGTCATGACCGCCGCCCACTGCATCTACGGCATGAGCTCCACCAACAGTGGCTTGATGGTGGGCGTGGGCGACCACAACATGTACCAGACCAGCGACGACGTGTCGGGAGCCACGCGCCTGGTGGCCGTGGAGCGAATCATCCAACACCCTGACTACAACACACGCACCCTGGACAACGACATCGCGCTGCTCAAGCTGTCCGAGACGCTGGACCTCACCCAGTACAAGGAGGTGGGCGCCGTGTGTCTGCCCGCCGACGACTCCAAGACTTACGCCGGGGAGTTGGCCACTGCCTCTGGCTGGGGCACAACCAGTTCCGGCGGCTCACAGCCCTCCACGCTCTACGAGGTGGTGGTGCCCATCCTGGAGCCTTCCTGCTGGGGCATGTCCGTCACCGCCAACATGCTGTGTGCCGGCCTGGAGGAGGGCGGCAAGGACACCTGCCAGGGAGACTCCGGCGGCCCTCTCTACGTGGAGGAGAACTCCGTGCGCGTGCAGGTCGGTATCACCTCGTGGGGCTACGGCTGTGCTGACGCCAACAGCCCCGGCGTGTACGCCAGGGTCAGCAAGTACGTGTCCTGGATCCAGCAGAACACCCAGGACGCCACCTACTGCCAGTAG

MKACSVLVLVAVACVPALAQEGGEYVDPKQGEYVDPKQSCGGTMTVGAEPVVISSPNHPNDYANRQRCKWRFTAEDPDDALFLSCEEFSVLCKGDRLNIIEGGVVQERLCGSDPVEYASEGNVMVLKFRTNRRGTSSGFSCTVTSSSYYEGSSSSSSSSTEEGEENTTPSSNTGSCKCGVPNLNRIVGGQEVNPKNKYPWQVGLKMSNGRNYWCGGSIINDRYVMTAAHCIYGMSSTNSGLMVGVGDHNMYQTSDDVSGATRLVAVERIIQHPDYNTRTLDNDIALLKLSETLDLTQYKEVGAVCLPADDSKTYAGELATASGWGTTSSGGSQPSTLYEVVVPILEPSCWGMSVTANMLCAGLEEGGKDTCQGDSGGPLYVEENSVRVQVGITSWGYGCADANSPGVYARVSKYVSWIQQNTQDATYCQ

transcript_transcript/6164_Pt_Mix_transcript6164/f2p0/3211

ATGGACGTGAGGCTCTGTTGCCTCCTGCTGTTTGTCGCCGCATCCTGGATGCCCGGCCTGGCCAATGACTTTATGATAAGCCCCACTCACAGAGAGCTGCCTGCGCTTCACAAATCAGGACAAAACGAAAGGCGCCCCAAAGATGAAGTAGCCATTGTTGTGCCCAAAGCGAAAGAACAGCCGGTAGATGCCATCCAAAAAGGAACCATTGAGACTAAGCTGGGAGGACACCCCGTCGTTCACAAACAAATAGATATCTCTGGGAGGCCCAAGCAGGATGGAGACCCAGTGGAATGTGAAGGAAGAAAGGCAGTGGTGGTGGGCGAGGAGGGGCGCATCATCACCAGCCCGAATTATCCAGACAATTATTCCGACGAAGAAATATGTGCCGTGAGGATTATCGCCGAGAACAGGGAATGCACGCTGGAGATTGACTGCTCCGACTTCCACGTGCAGGACTCAGAGGATTGCAGAAAAGACTACCTGATGATTAAGGAGAATAGCGAGACGAAGGAGAAATTCTGCGGTGACGAGGGATTTATATATGAGAGTAGCAAAAATGCCGTAACGCTCAAGTTCAAATCCAACCGGCGGGTAACGGACAAAGGATTCTCCTGCTTAGCTCGCTCCGTATGTCCAACCACCACCACCACCACCACTTCCACTGAGACCACGCCTACCACCATCACGACTACTGAGACCACGCCCACTACCACCACGACCATGACCACTGAGACCACGCCCATCACCACCACTACCGCTGAGACCACGCCCATCACCACCACTACCGCTGAGACCACGCCCATCACCACCACTACCACTGAGACCACGCCCATCACCACCACTACCGCTGAGACCACGCCCATCACCACCACTACCACTGAGACCACGCCCATCACCACCACTACCACTGAGACCACGCCCACCACCACCACGACCACTGAGACCACGCCCACCACCACCACTATCACGACTACTGAGACCACGCCCACCACCACCACCATCACTACCAGTGAGACCACGCCCACCACCACTACGGCCATAGACACTACGACCACCATCACGACCACTGAGGCCACGACCACCACGAGTTGCACAGGATCGTGTCCTTGCGGTCTTGCCAATCTAGCGAGGGTCGTGGGCGGGGAGGATGTAGATCCTGCCTACAAGTACCCGTGGCATGTGGGAATAAAGTATGTATGGAACAGAAAGTACTGGTGCGGCGGCTCCATCATTAACGACCGGTATATCCTGACAGCCGCCCATTGTGTTAAAAACAGATTTAGACGGTGGCTGGTGGTGGGCGTGGCCGACCACGACATGACGTCCACCGACGGGGTGACCCAACTGGTACGCGTTCAGGAGATTATCGTTCATCCTGACTATAATATCAACACCCTTGACAGTGATATCGCGCTGCTCAAGCTGTCACAACCACTGGACCTGACTCAAGTCGAGCACATTCGCCCCGTGTGTCTGCCCGCCGATGACTCCAACACCTATGCCGGCGAAGATGCCACAGCGACAGGCTGGGGCATACTTCAGTCCTGGGGAAGTCAACCAGCAATACTGCAGGAAGTCACAGTGCCTATTCTGGATCCCTCCTGCCCGGGACAGATATCGAATTATATCACTGAAAACATGCTGTGTGCTGGCCTTGAAGAAGGTGGCAAGGACACCTGCCAGGGCGACGCGGGCGGTCCGCTTACTGTGCAAAATGACTGTTCCAAATATGAGCAAGTTGGCATCACCTCCTGGGGCTTTGGATGTGCCGACCCTGGCAGGCCCGGTGTATACACCAGGGTGAGCAGATTCCTTAACTGGATCTACGCCAACACTGTTGACGCCACCTACTGCCAGTAG

MDVRLCCLLLFVAASWMPGLANDFMISPTHRELPALHKSGQNERRPKDEVAIVVPKAKEQPVDAIQKGTIETKLGGHPVVHKQIDISGRPKQDGDPVECEGRKAVVVGEEGRIITSPNYPDNYSDEEICAVRIIAENRECTLEIDCSDFHVQDSEDCRKDYLMIKENSETKEKFCGDEGFIYESSKNAVTLKFKSNRRVTDKGFSCLARSVCPTTTTTTTSTETTPTTITTTETTPTTTTTMTTETTPITTTTAETTPITTTTAETTPITTTTTETTPITTTTAETTPITTTTTETTPITTTTTETTPTTTTTTETTPTTTTITTTETTPTTTTITTSETTPTTTTAIDTTTTITTTEATTTTSCTGSCPCGLANLARVVGGEDVDPAYKYPWHVGIKYVWNRKYWCGGSIINDRYILTAAHCVKNRFRRWLVVGVADHDMTSTDGVTQLVRVQEIIVHPDYNINTLDSDIALLKLSQPLDLTQVEHIRPVCLPADDSNTYAGEDATATGWGILQSWGSQPAILQEVTVPILDPSCPGQISNYITENMLCAGLEEGGKDTCQGDAGGPLTVQNDCSKYEQVGITSWGFGCADPGRPGVYTRVSRFLNWIYANTVDATYCQ

transcript_transcript/6925_Pt_Mix_transcript6925/f37p0/3042

ATGGGTCACGTGAATGTGGCGGTGGTGGTAGTGGCGGCGATGGCGGCGCTGGTGGGTTGCCAGACTCAGCAGGGCAGCAGCTGCGTGGACGGGAACGGACAAGCGGGAACCTGCATCAGCATCCGCTCCTGCCAGCCACTGCAGCAGCTCGTGCAGGCCTTAAGAACTAACACAGGACCTTCCAACGGCTTCCAAATTCTGCGTCGGTCAGTATGTAACATACAAATTAACAGAGAGCCACTGGTGTGCTGTGCCAGCAGTGGTGGTACTGGGCTTGACTTGTTGCCCAAGCGCTGTGGAGTGACGGGTCTGGTGGACCGCATTGTTGACGGGGAAGACGCTCCGCTGCTCGCCTGGCCTTGGATGGCGCTGCTCAGAGGAAAAGCTCGTGGTCAGCCAAGCACTTGGATCTGCGGTGGTGTGCTCATAAACGCTCGCTACGTCCTCACTGCAGCCCATTGCTTCAAGTCCATCTTCCAAATTGAACTGGAGTTCGTAAGGATTGGCGAGCACACGCTGAGCACGGTGCAGGACTGTGAGAATGGTTTGTGTGCTCCACTTGCCCAGGATATCTTGGTGGAGCAAATAATCATGCATCCAGAGTATGGATCTCCATGCAAGGAGTGCAACGACATTGCGCTGCTCAGGCTGTCCCATCCCGCCCAGCTGAACCCTATTCATGTGGTGCCAATTTGCCTTCCTGTTGACCCACCCAAAGACATGGAATTCTCTGAGGAGGAGTTCCAAGGGAAGGTTGCTTATGCTGCTGGTTGGGGATCTACTTCCCGGAGTCCCCTCAATCCTGTCACTCCAGATGTACTACAGCAGGTTCTCCTTCCCATCAACGAGGGAAACGTTTGTAATCAATTGAAAAGTGGCTATCCGAATCGTCGATGGACTCTGTGTGCTGGCGGGGAGGGCAAGGACACCTGCAAGGGAGATTCAGGCGGACCACTCATACTGAGTAATAAAATTGGAACCAAAGGATTTGTGGTGGGCATCACAAGCGTGGGACCAAAGGTGTGTGGCAGGCAGAAGACTCAGGCCCTTTACACCAATGTTCACTTTTACGTGCCGTGGATCCTCAAGAACCTGCGGCCTTAG

MGHVNVAVVVVAAMAALVGCQTQQGSSCVDGNGQAGTCISIRSCQPLQQLVQALRTNTGPSNGFQILRRSVCNIQINREPLVCCASSGGTGLDLLPKRCGVTGLVDRIVDGEDAPLLAWPWMALLRGKARGQPSTWICGGVLINARYVLTAAHCFKSIFQIELEFVRIGEHTLSTVQDCENGLCAPLAQDILVEQIIMHPEYGSPCKECNDIALLRLSHPAQLNPIHVVPICLPVDPPKDMEFSEEEFQGKVAYAAGWGSTSRSPLNPVTPDVLQQVLLPINEGNVCNQLKSGYPNRRWTLCAGGEGKDTCKGDSGGPLILSNKIGTKGFVVGITSVGPKVCGRQKTQALYTNVHFYVPWILKNLRP

transcript_transcript/8208_Pt_Mix_transcript8208/f3p0/2934

ATGGGTCACGTGAATGTGGCGGTGGTGGTAGTGGCGGCGATGGCGGCGCTGGTGGGTTGCCAGACTCAGCAGGGCAGCAGCTGCGTGGACGGGAACGGACAAGCGGGAACCTGCATCAGCATCCGCTCCTGCCAGCCACTGCAGCAGCTCGTGCAGGCCTTAAGAACTAACACAGGACCTTCCAACGGCTTCCAAATTCTGCGTCGGTCAGTATGTAACATACAAATTAACAGAGAGCCACTGGTGTGCTGTGCCAGCAGTGGTGGTACTGGGCTTGACTTGTTGCCCAAGCGCTGTGGAGTGACGGGTCTGGTGGACCGCATTGTTGACGGGGAAGACGCTCCGCTGCTCGCCTGGCCTTGGATGGCGCTGCTCAGAGGAAAAGCTCGTGGTCAGCCAAGCACTTGGATCTGCGGTGGTGTGCTCATAAACGCTCGCTACGTCCTCACTGCAGCCCATTGCTTCAAGTCCATCTTCCAAATTGAACTGGAGTTCGTAAGGATTGGCGAGCACACGCTGAGCACGGTGCAGGACTGTGAGAATGGTTTGTGTGCTCCACTTGCCCAGGATATCTTGGTGGAGCAAATAATCATGCATCCAGAGTATGGATCTCCATGCAAGGAGTGCAACGACATTGCGCTGCTCAGGCTGTCCCATCCCGCCCAGCTGAACCCTAGTCCCCTCAATCCTGTCACTCCAGATGTACTACAGCAGGTTCTCCTTCCCATCAACGAGGGAAACGTTTGTAATCAATTGAAAAGTGGCTATCCGAATCGTCGATGGACTCTGTGTGCTGGCGGGGAGGGCAAGGACACCTGCAAGGGAGATTCAGGCGGACCACTCATACTGAGTAATAAAATTGGAACCAAAGGATTTGTGGTGGGCATCACAAGCGTGGGACCAAAGGTGTGTGGCAGGCAGAAGACTCAGGCCCTTTACACCAATGTTCACTTTTACGTGCCGTGGATCCTCAAGAACCTGCGGCCTTAG

MGHVNVAVVVVAAMAALVGCQTQQGSSCVDGNGQAGTCISIRSCQPLQQLVQALRTNTGPSNGFQILRRSVCNIQINREPLVCCASSGGTGLDLLPKRCGVTGLVDRIVDGEDAPLLAWPWMALLRGKARGQPSTWICGGVLINARYVLTAAHCFKSIFQIELEFVRIGEHTLSTVQDCENGLCAPLAQDILVEQIIMHPEYGSPCKECNDIALLRLSHPAQLNPSPLNPVTPDVLQQVLLPINEGNVCNQLKSGYPNRRWTLCAGGEGKDTCKGDSGGPLILSNKIGTKGFVVGITSVGPKVCGRQKTQALYTNVHFYVPWILKNLRP

transcript_transcript/18687_Pt_Mix_transcript18687/f2p0/2042

ATGAAGTGGAGAGTGTGTTGCAGCCTGGTGGTGGTGGCGGCGGTGGTGGCGATATGTGGGCAAGACAACGGTGGCTTCAGAGTGTGCTGCCCAGACTCTGACCCCGGCTCCAACAACCCCGTCGTCTCCGGCAGCAGCTCCACCACTTCCCAGCCTACTGTGGACGGAGAAACACTGCTGCCGAAGGGGAGTGAGTGCGGGCAATCCGGTAACCACAGGGTCGTGTTCGGGGAAGATGCTCCTCTCTATGCCTATCCTTGGATGGTGCTGCTCGGATACAGAGACAGAGCCAACCCATCCTGGAAGTGTGGCGGCGCCCTCATCAACGACCGCTACGTGCTCACCGCTGCTCACTGCGTCCACCGTAACTTCACCATCCCCTCTGGCAACGGCGATGTAGTGGCCCTGCGAGTGGGAGAACACACCATATCCATCGACCCAGACTGCGCCCTGACCGATGCCGTGCCGTGTTCCTCCCCCGAGGACTTCGACCCAGAAGAGGTGATCGTTCACCCTCAGTTCAACAAGCGAGCTCCTGTGAGTGACGACATCGCCCTCATTCGCCTCAACAAGAAGGTCACGTTTGGCTTTTCCGCTAAGCCAGTGTGCCTTCCCGCTGCGGGCTTGGACGTGAAGAGCTTCCTGGGTGCGCGCGACGCCGTGGTGGCAGGTTGGGGCGCCACAGAGACCACCTCCACCTCTGACGTCCTGCAGGCTGCGAAGGTTCCATTCGCAGAGAAGTCCACGTGCGAGCCTTTCTATCGCAACCAGCTGGTGGACGAGCAGGTGTGCTTCGGCGGGAGAGGCAACGTAGACTCCTGCTTCGGAGATTCTGGTGGCCCAGTCTTCCAAACCCACAACGAACTCCCTCGCTTCACCGTGCTGGGCATCGTGTCCCGCGGCGTGCGTGAGTGCGGGACTCCTGGAGTTCCTGCTGTCTACACCAACGTCGCCTTCTACAGGCAGTGGATAGCAAGCAGCATCAAGCCCTAG

MKWRVCCSLVVVAAVVAICGQDNGGFRVCCPDSDPGSNNPVVSGSSSTTSQPTVDGETLLPKGSECGQSGNHRVVFGEDAPLYAYPWMVLLGYRDRANPSWKCGGALINDRYVLTAAHCVHRNFTIPSGNGDVVALRVGEHTISIDPDCALTDAVPCSSPEDFDPEEVIVHPQFNKRAPVSDDIALIRLNKKVTFGFSAKPVCLPAAGLDVKSFLGARDAVVAGWGATETTSTSDVLQAAKVPFAEKSTCEPFYRNQLVDEQVCFGGRGNVDSCFGDSGGPVFQTHNELPRFTVLGIVSRGVRECGTPGVPAVYTNVAFYRQWIASSIKP

transcript_transcript/22205_Pt_Mix_transcript22205/f4p0/1750

ATGTGGTACGCATGTGTGGAAGCGGTATTATTGCTGGGACTGTGCTTCGCCGCTATTGCAGAAGCCGCCAGCATATTTTTCCCAGACCAGCTAAATCACGCTGAACATGAGTCCTGCAGGACACATTTAAGCACTGATGGTACGTGTGAGCGCACTTGTGGCGTCTCCTTTAACAGCAGAGAGCTTAGCAAGTGTGGCATCAAGGATTCTGCATTCCTCTATTGTTGCGACCCAAACCATGTCAGGACCACCGACCGCGTGGTGGACATCTCTCCTCCATTCACAGAGTTTCAATGTGGAAGAAATTCAAGGAATAGTTTTTTCTTTCCCCCGTTTTACGATCCGCCAGCTGACTTCCTACGTCCCGAAGCTGACTTGCCTTCCCCAGATGTTTTGGCAGCGGCCATTCCACCTTCTCATACACCGGGTATTCCTGAAGAGGCAATAGCAGAGTATGAAGATTTTACTGTCCCTCCTGGTGTGTACGAAACAATTGGGGGAATCGTAGCTGAGAAGAACGCTTGGCCTTGGATGGCGCTGGTGGGGAAAAACACCACACACGGTCCTAATTGGTTCTGCGGCGGAGTGTTGATCAACGAGCAGTGGGTCCTGTCAGCTTACCACTGTTTCATCTACCACGTTGCCGAAGTAGTACGTTTAGGCGAGCATGATTATAACGATGACTTTGAAGGAGCCCATCACGAGGACTTCGATGTTTTGGAGACAGTACTCTACCCTGACTACAAACACCCTCAGGCTTACCACGACATAGCCCTCCTTAAGCTGGCCTCCAAGGTTACCATTCAGGATTACATCAGACCTGTTTGTCTGCCCTGGGGAAGCGCGAGCGACAGTGTAGTGACGGACCACATTGCAACGCTAACTGGCTGGGGTGACACCGAGTACGGTGGATTTCCCAGCCCGATCCTACAGGAGGTGAACGTCACAGTATTCTCTGTTGGCCAGTGTGACAGCAGCTACTCAAAACTGCTTCACTACGGCGCCACCTGGCCTCAGGGCATTGGAGAGGAGACCCTGTGTGCCGGAGACCCTAATGGAGGGCGGGACGCGTGTCAGGGCGACTCGGGCGGACCTCTGGTGTCTCAGGACGAGCATGGAGCTTACATATTGGCTGGTATCGTTTCAAGAGGTTACGGCTGCGGCCACAAGGACTACCCAGGACTGTATGCCAACATGCGTCACCCTGCATATCTCACATGGATTAAAAAGATCGCCTTTGCGTCTCCTTGA

MWYACVEAVLLLGLCFAAIAEAASIFFPDQLNHAEHESCRTHLSTDGTCERTCGVSFNSRELSKCGIKDSAFLYCCDPNHVRTTDRVVDISPPFTEFQCGRNSRNSFFFPPFYDPPADFLRPEADLPSPDVLAAAIPPSHTPGIPEEAIAEYEDFTVPPGVYETIGGIVAEKNAWPWMALVGKNTTHGPNWFCGGVLINEQWVLSAYHCFIYHVAEVVRLGEHDYNDDFEGAHHEDFDVLETVLYPDYKHPQAYHDIALLKLASKVTIQDYIRPVCLPWGSASDSVVTDHIATLTGWGDTEYGGFPSPILQEVNVTVFSVGQCDSSYSKLLHYGATWPQGIGEETLCAGDPNGGRDACQGDSGGPLVSQDEHGAYILAGIVSRGYGCGHKDYPGLYANMRHPAYLTWIKKIAFASP

transcript_transcript/23467_Pt_Mix_transcript23467/f73p0/1655

ATGGAGCGGGTGTGTGCGCTAGTACTACTGATGATGCTCTCCCAAGCCACAGAGGCCAGTGCCATTATATTTCCCGGTCAACTGGATCATGCTGAAGGGGATGACTGCCCCACTAGCTCTGGCGGGCCGGGCAAGTGTTCCCGGTCCTGCGAACACTCTGTTGGCCCAGGGGAGCCTTCCAGGTGTGGCATTAAGGACTCCGCCTTCCTCGTTTGCTGCGACATACCCTCGAATCCGGGTGTAGTCACTGTTGCCCTCACGGACATAGACCCTCCTAAAGTCGCCTTTGAATGTGGGAAAAATGTACAAAACTTTCTAAATTCGCGTGGGCCTTCTCGTGGCAATGTACTACGCCCTGAGCCACCAACACCTCCAAGCCCGATTATAGAAATTGCAGCAAGTCGGCCTGCTCGGTCTGCCCCTGGTGGATTTGTGCCTTCGGGTGGAAGATCTGAAACAGCTGAAGGAATAGGTGGAGTTAACACAAAAAGTGCCTGGCCATGGATGACACTGCTCGGGGAAACCAGTAATGCGGGAATTAGATGGTTCTGTGGTGGAGTGTTGATCAACGAGCAGTGGATCCTGTCAGCCCTCCATTGCTTCTTCCATAACTCGGCAGATGTAGTTCGTCTTGGTGAACACAACTACAACGACGACAACGATGGGGCGATCCACGAGGACTTTGGTGTGACGGAGACGGTGCTTTATCCTGACTTTACGTTTGGAGAGGGTTATCATGACCTGGCACTCCTCAAGCTGGACAAACCAGTCGCAGTACAGGAGTTCATCAGTCCTGTTTGCTTGCCATGGGGAACGGAAAGTGACAGTGATGTTGCCTTCCGATCGGCAACACTTACTGGCTGGGGTGACACTGAGCGAGGTGGGTTTCCTACCTCTATCTTACAAGAGATCAGCGTGACAGTGTTCCCGTCTGCTATGTGTAACGATAGCTACTCTACATTACGTAACTTTGAAGACACCTGGCCTCGAGGCATCGGGGACGAGATCCTGTGTGCTGGGGATCTTAATGGAGGGCGCGACGCTTGCCAGGGTGATTCGGGTGGACCACTTGTGACTGAAGAAGCCAATGGACTCTTCGTGCTGGCGGGCATTGTGTCGCAAGGATACGGCTGTGGCCACAAGGACTATCCAGGACTGTACGTCAACATCCGCCATAAGGCTTACCTTGCCTGGATCAAGAAGGTCGCATTCACTTCACCCTGA

MERVCALVLLMMLSQATEASAIIFPGQLDHAEGDDCPTSSGGPGKCSRSCEHSVGPGEPSRCGIKDSAFLVCCDIPSNPGVVTVALTDIDPPKVAFECGKNVQNFLNSRGPSRGNVLRPEPPTPPSPIIEIAASRPARSAPGGFVPSGGRSETAEGIGGVNTKSAWPWMTLLGETSNAGIRWFCGGVLINEQWILSALHCFFHNSADVVRLGEHNYNDDNDGAIHEDFGVTETVLYPDFTFGEGYHDLALLKLDKPVAVQEFISPVCLPWGTESDSDVAFRSATLTGWGDTERGGFPTSILQEISVTVFPSAMCNDSYSTLRNFEDTWPRGIGDEILCAGDLNGGRDACQGDSGGPLVTEEANGLFVLAGIVSQGYGCGHKDYPGLYVNIRHKAYLAWIKKVAFTSP

transcript_transcript/23931_Pt_Mix_transcript23931/f2p0/1639

ATGCAGCGAGTGAGTGCGGTGGCGGTGGTGGTGGTGGCGGTGGTGCTGGGTGGAGGCGAGGTGGAAGCAGCGCATCGGTCAATCAGACAATTGTTCCCTGAGTCCGGTCCAGATTGCAGCAACGGGCGACAGTGCATTCCCATCAGATCTTGCCCCGTGTTCGTGAGACTCCTTGAGAATCCCACGCCTGAGGGAATCAAGATGCTGCGAGAGAGTCACTGTGGCTTCGCTGGTGACCGGAGTCCCTTGACTTGCTGCCCAGATGGTGACTCCGGGAAACCCACAAGCTCAAACATCGACTTCAGACCCACAGTGAGCCCAAGCAGACCAATACCGATCCCTACCAGCTCCCCCACTTCCCCACCTACTCGTCCACCTACACGCCCTCCCACCCAGCCGACCACCACTCAACTTCCGGCGTCTGGTGGTGATCTGCTGCCTAGTGAATGTGGGTTCTCCTCCGCTGGTCAAACGAGGATATTCTTTGGTGAGGACAGCCCACTAGGAGCCTACCCCTGGATCGCCCTGCTTGGATATACCTCACGGTTCCAGCCTCAGGTGGTGTGGGGCTGCGGCGGGTCTCTCATCAACTCTCGCTACGTGGTCACAGCTAGCCATTGCACCGCCGAGGAGTTCACTTTCAATAGAGATCTGACCGTGATTCGCCTGGGAGAGCACAACCTATCCACGGAGATCGACTGTGAGAGCAGAGGTGGGAGAAGGACCTGCGCCCCGCCCCATCAGACCTTCACGCCCGTTGAGATCATCCGCCACTCAGATTTCAACAGCCGAGGCACCGTAAGCGACGATATAGCGCTGATAAGACTCGATAAGGAGGTTGAGTTTAATGCTTTCGTGGGGCCCATCTGCATCCCGCCGCCCACCACTGACCTCACCACCTTCCTCGGGAATCGCCAGGCCTTCGTTGCAGGCTGGGGCGCTACCGAGAGGGGCCCAGACACCCAGATTCTGCAACAAGTCCGCATTCCCTTCGTCAGTAGAGATGAGTGCAACCCTCACTACAACAACGCCCTTCTGCCGGAACAGGTGTGCTTCGGAGGTGACGGGCGACGTGACTCCTGCTTCGGTGACTCAGGGGGTCCCGTGGTGGCGCCTGCACCTGGGGGAGGGTCCTTCCTGCTGCTCGCTCTGGTGTCGTTCGGTCAGCCCTCGTGCGGTGTGGAGGGCGTGCCTGCTGTCTACACCTCCATGGCCGCCTACAGGAGCTGGATACTGGAGAATATAAAGCCTTAA

MQRVSAVAVVVVAVVLGGGEVEAAHRSIRQLFPESGPDCSNGRQCIPIRSCPVFVRLLENPTPEGIKMLRESHCGFAGDRSPLTCCPDGDSGKPTSSNIDFRPTVSPSRPIPIPTSSPTSPPTRPPTRPPTQPTTTQLPASGGDLLPSECGFSSAGQTRIFFGEDSPLGAYPWIALLGYTSRFQPQVVWGCGGSLINSRYVVTASHCTAEEFTFNRDLTVIRLGEHNLSTEIDCESRGGRRTCAPPHQTFTPVEIIRHSDFNSRGTVSDDIALIRLDKEVEFNAFVGPICIPPPTTDLTTFLGNRQAFVAGWGATERGPDTQILQQVRIPFVSRDECNPHYNNALLPEQVCFGGDGRRDSCFGDSGGPVVAPAPGGGSFLLLALVSFGQPSCGVEGVPAVYTSMAAYRSWILENIKP

transcript_transcript/24153_Pt_Mix_transcript24153/f4p0/1622

ATGGAGAAGCTTTGCCTCCTCTTGCTGCTCGTCACCGCGGAGGCACAAGTTATATTCCCTGGTGAGGACGTCAGGGTCACCTCCGCAGCGCCTGCAGTGATCAACCCTTCTGACAACCGCAACACTGCTCTTGAGGATGATAGGATATATTTTCCGGATGTTATAATTACCCGACCCCCTGCTATCACCTCAACCACCACCACTACCACCACCCCCACCACCACCACTGCTACCACTACCACCACCACAAGGCCTTCAGGGGACGGGCTTCAACCCGTTCGCAGCCCTAATCCCAGCCCTCGTCCTAGCCACAGCCCAGTTTCCAGCCACAACCAGAGCGTGCCATCGAGTGTTCCTTCCTCCATCATTCCTAGCGAGTGCGGCAAATCGATGTTTGACTCTCGGGTCGTTGGAGGAACAGTCACAGAGCCTGGGGAATGGCCGTGGTTGGCAGCGCTGGGCCGGATGAGCAGAGGAAGATTTTCTAATTTATGTGGCGGCTCTCTCTTCACTGTTCGCCACGTCCTGAGTGCTGAACACTGCTTCATTAGTCTTTTCGTCCCGAACATAGTGAGATTGGGAGAATATGACGTGACTCGCTTTGATGAAGTGCCGGGCACCCAGGACTTCGGCATCCTGGAGCGTCACAACAAGCAGTACAAATCACAGACGTATGAGAATGACATTGTCATTATCGTCTTGGATCGCGATGTTGTCTTTACAGATTACATTCGACCGGTGTGTCTTCCATTTAACGAGCGAGGGAACAATTTTGCAAACGAGAAACTGATAATAGTTGGCTGGGGGAAGACTGACTATGAGACCTCCAGCTACACAGACGTGCCCTTTGATGCCATTGTGCCGGTGGTGGACAGAGAGCAGTGTGCCCAGAGTTACAAGCGAGCAGGGAACAGACGTGTCAAACCTGTGGTGGACGAGAGACATCTGTGTGCTGGTAACGGGACGAAGGACTCCTGCAGTGGTGACAGCGGCGGCCCTCTGCACCACGTGTCCCTTAACGACGGCCGGTTCTACATTGTTGGAATAGTGTCCTTCGGCTTCGAGTGTGCCAGTGCTGATTACCCCGGGGTGTACACGCGGGTCACCACCTTCCTGGATTGGATCATGGAAGTCGTGCAGTAA

MEKLCLLLLLVTAEAQVIFPGEDVRVTSAAPAVINPSDNRNTALEDDRIYFPDVIITRPPAITSTTTTTTTPTTTTATTTTTTRPSGDGLQPVRSPNPSPRPSHSPVSSHNQSVPSSVPSSIIPSECGKSMFDSRVVGGTVTEPGEWPWLAALGRMSRGRFSNLCGGSLFTVRHVLSAEHCFISLFVPNIVRLGEYDVTRFDEVPGTQDFGILERHNKQYKSQTYENDIVIIVLDRDVVFTDYIRPVCLPFNERGNNFANEKLIIVGWGKTDYETSSYTDVPFDAIVPVVDREQCAQSYKRAGNRRVKPVVDERHLCAGNGTKDSCSGDSGGPLHHVSLNDGRFYIVGIVSFGFECASADYPGVYTRVTTFLDWIMEVVQ

transcript_transcript/24556_Pt_Mix_transcript24556/f27p0/1599

ATGGAGCGGGTGTGTGCGCTAGTACTACTGATGATGCTCTCCCAAGCCACAGAGGCCAGTGCCATTATATTTCCCGGTCAACTGGATCATGCTGAAGGGGATGACTGCCCCACTAGCTCTGGCGGGCCGGGCAAGTGTTCCCGGTCCTGCGAACACTCTGTTGGCCCAGGGGAGCCTTCCAGGTGTGGCATTAAGGACTCCGCCTTCCTCGTTTGCTGCGACATACCCTCGAAATGTGGGAAAAATGTACAAAACTTTCTAAATTCGCGTGGGCCTTCTCGTGGCAATGTACTACGCCCTGAGCCACCAACACCTCCAAGCCCGATTATAGAAATTGCAGCAAGTCGGCCTGCTCGGTCTGCCCCTGGTGGATTTGTGCCTTCGGGTGGAAGATCTGAAACAGCTGAAGGAATAGGTGGAGTTAACACAAAAAGTGCCTGGCCATGGATGACACTGCTCGGGGAAACCAGTAATGCGGGAATTAGATGGTTCTGTGGTGGAGTGTTGATCAACGAGCAGTGGATCCTGTCAGCCCTCCATTGCTTCTTCCATAACTCGGCAGATGTAGTTCGTCTTGGTGAACACAACTACAACGACGACAACGATGGGGCGATCCACGAGGACTTTGGTGTGACGGAGACGGTGCTTTATCCTGACTTTACGTTTGGAGAGGGTTATCATGACCTGGCACTCCTCAAGCTGGACAAACCAGTCGCAGTACAGGAGTTCATCAGTCCTGTTTGCTTGCCATGGGGAACGGAAAGTGACAGTGATGTTGCCTTCCGATCGGCAACACTTACTGGCTGGGGTGACACTGAGCGAGGTGGGTTTCCTACCTCTATCTTACAAGAGATCAGCGTGACAGTGTTCCCGTCTGCTATGTGTAACGATAGCTACTCTACATTACGTAACTTTGAAGACACCTGGCCTCGAGGCATCGGGGACGAGATCCTGTGTGCTGGGGATCTTAATGGAGGGCGCGACGCTTGCCAGGGTGATTCGGGTGGACCACTTGTGACTGAAGAAGCCAATGGACTCTTCGTGCTGGCGGGCATTGTGTCGCAAGGATACGGCTGTGGCCACAAGGACTATCCAGGACTGTACGTCAACATCCGCCATAAGGCTTACCTTGCCTGGATCAAGAAGGTCGCATTCACTTCACCCTGA

MERVCALVLLMMLSQATEASAIIFPGQLDHAEGDDCPTSSGGPGKCSRSCEHSVGPGEPSRCGIKDSAFLVCCDIPSKCGKNVQNFLNSRGPSRGNVLRPEPPTPPSPIIEIAASRPARSAPGGFVPSGGRSETAEGIGGVNTKSAWPWMTLLGETSNAGIRWFCGGVLINEQWILSALHCFFHNSADVVRLGEHNYNDDNDGAIHEDFGVTETVLYPDFTFGEGYHDLALLKLDKPVAVQEFISPVCLPWGTESDSDVAFRSATLTGWGDTERGGFPTSILQEISVTVFPSAMCNDSYSTLRNFEDTWPRGIGDEILCAGDLNGGRDACQGDSGGPLVTEEANGLFVLAGIVSQGYGCGHKDYPGLYVNIRHKAYLAWIKKVAFTSP

transcript_transcript/24684_Pt_Mix_transcript24684/f7p0/1591

ATGGACCACCAGACCTTCGCAATCTTCCTGTTTTTGTTGGCATCCACAGCAGAGAAGGCAACAGTGGTGGACACCACACCAAACGATAAGGTGGTGGACATTTCCCCTCCGTCAGGATACGACGCACTGGACTGCGGGAAAAGTGCGGTCAAAATCATCGAGACTTTTGGACCTAACTTCGGGTTAAGTGCAAGCGACGTCTCCTTGAGTCGAAAGAAGCGAAGTAATCCACCCTTTATGTCAGTGCCACTACCAAAGACGCAGCCCGGTGAGCTTTTACCAGTTGGAGTAGGTCATAACAAGCCTACAGTTGTTGGTGGAGAAAAATCGAGAAGAAACGCTTGGCCATGGATGGCACTGATTGGGAAGCGAGACCCAGCTGGCATCAACTGGTTCTGCGGCGGGGCACTAATTAATGAATACTGGGTCCTCACGGCGCTTCACTGCTTCCACAACAACAAGGCCGAGGTAGTGCGTCTTGGCGAGCACGACTATAACAATAATAATGATGGGGCTAACCACGAGGACATTCCAGTGGCGGATAAGATAGAGTATCCTGACTACATTTACCCACAGGGCTACCACGACCTAGCTCTGCTCAGGCTGGAACGCAAAGTCACACTGCAGAAATTCATCTTACCGGTGTGTCTTCCTTGGGGAAGTAAGATCGACGAGAACATCGTGGGTAGCAAGGCAACAGTGGTGGGTTGGGGCCACACATTATTCGGAGGGTTCTCAAGCTCTACGCTGATGGAGGTGGACGTGCAGGTGTTCCCTACCTCCAGATGCAACAGCAGCTACTCCTCTCTCCCTCCATACCCAGACCAGTGGCCACGCGGCATTGGGGAAGAGACACTGTGTGCCGGGGATGTCGAGGGTGGCAAGGACTCCTGCCAGGGGGACTCTGGCGGACCTCTGGTCACTCGGGACAGCCGGGGGCGCTACGTGGCGGCGGGCGTAGTGTCGCAAGGCTACGGGTGTGGCCATAAGGACTATCTCGGCCTGTACGTCAACCTGCGCTACCCAGCCTACCTTGCTTGGATTAAGAAAGTGGCTTTTGCTCCTTAG

MDHQTFAIFLFLLASTAEKATVVDTTPNDKVVDISPPSGYDALDCGKSAVKIIETFGPNFGLSASDVSLSRKKRSNPPFMSVPLPKTQPGELLPVGVGHNKPTVVGGEKSRRNAWPWMALIGKRDPAGINWFCGGALINEYWVLTALHCFHNNKAEVVRLGEHDYNNNNDGANHEDIPVADKIEYPDYIYPQGYHDLALLRLERKVTLQKFILPVCLPWGSKIDENIVGSKATVVGWGHTLFGGFSSSTLMEVDVQVFPTSRCNSSYSSLPPYPDQWPRGIGEETLCAGDVEGGKDSCQGDSGGPLVTRDSRGRYVAAGVVSQGYGCGHKDYLGLYVNLRYPAYLAWIKKVAFAP

transcript_transcript/25451_Pt_Mix_transcript25451/f2p0/1451

ATGAAGTGGGTGTGTGTGTTGGTGCTACTGCTGGCGCTCTCCAAAGCAACAGAAGCCAGTGCCATTGTATTTCCCGGCCAACTGGACCACGCTGAAGGGGATGACTGCCCCACTAGCTCTGGCGGGCCGGGCAAGTGTTCCCGGTCCTGCAAACACTCTGTTGGCCGTGGGGAGCCTTCTAAGTGCGGCATTAAGGACTCCGCTCTCCTCGTCTGCTGCGACATACCCTCGAATCGAGGTGCTGTCACTTTGGCACTTACGGACATCAGCGCCCCTAGCGTCACCTTCCAATGTGGAAAAAATGCAGAAAACTTTTTATTTTTGTTTGGGCCTTCTGTCGGTGATGTGCTGCGCCCTGAGGAATTTACACCTGGCGAGATACCAGAGGTTGAGGGGAATAGGCCTGTTCGGTCTGTTCCTGGTGGATTGCACCCTAATGAATTTGCACGGCCAGAGTTTATAAACCCCCAAGCAGGAAGAGAAGCAGCAATAGGAGCAATTAACTCAAAGCGAAATGCGTGGCCATGGATGGCACTGATTGGAGAAAATGATGTAGTGGGAATCAGGTGGTTCTGCGGTGGAGCGTTGATCAACGAGCAGTGGGTCCTGACAGCCCTCCACTGCTTCTTCCAGAACACGGCAGAAGTGGTTCGTCTTGGTGAACACAACTACAACGACGACAACGATGGGGCGATCCACGAGGACTTTGGTGTGACGGAGACGGTGCTTTATCCTGACTTTACGTTTGGAGAGGGTTATCATGACCTGGCACTCCTCAAGCTGGACAAGCCAGTCGAAATACAGGAGTTCATCAGTCCTGTTTGCCTGCCGTGGGGAACGGAGAGCGACAATGATGTAACGACACGGAAGGCGACACTCACTGGTTGGGGCGACACTTTGCTGGGAGGGTTTCCAAGCTCCATCCTGCAAGAGGTCAACGTGACAGTGTTCCAGTCTGGCCAGTGTGACCGCAGCTACTCTACCTTGCCCGATTATCCAAACACCTGGCCTCAAGGAATTGGGAAGGAGATGCTGTGCGCTGGAGATCTTGATGGAGGGCGGGACGCTTGTCAGGGTGACTCAGGTGGACCTCTTGTGACTCGGGAAGCACGTGGACGCTTCGTGCTGGCGGGCGTCGTGTCGCAGGGGTACGGCTGTGGCCACAAGGACTATCCCGGACTGTACGTCAACATGCGCCATGCGCCCTATCTTGCCTGGATCAAGAAGGTTGCGTTCACCACATCTTGA

MKWVCVLVLLLALSKATEASAIVFPGQLDHAEGDDCPTSSGGPGKCSRSCKHSVGRGEPSKCGIKDSALLVCCDIPSNRGAVTLALTDISAPSVTFQCGKNAENFLFLFGPSVGDVLRPEEFTPGEIPEVEGNRPVRSVPGGLHPNEFARPEFINPQAGREAAIGAINSKRNAWPWMALIGENDVVGIRWFCGGALINEQWVLTALHCFFQNTAEVVRLGEHNYNDDNDGAIHEDFGVTETVLYPDFTFGEGYHDLALLKLDKPVEIQEFISPVCLPWGTESDNDVTTRKATLTGWGDTLLGGFPSSILQEVNVTVFQSGQCDRSYSTLPDYPNTWPQGIGKEMLCAGDLDGGRDACQGDSGGPLVTREARGRFVLAGVVSQGYGCGHKDYPGLYVNMRHAPYLAWIKKVAFTTS

transcript_transcript/26763_Pt_Mix_transcript26763/f88p0/1364

ATGATGAAGGTACTTGGCTTCTTCCTCGTGGTTCTGACGGTCGCAAAGGCTCAAATTATCTTCCCCAATGAAGAAGGTGCTCCTAATCCTGATCCTGTCGACACTGAACTCTTTGAGGCCCTGGAGGTGGACGACCGGCAGGCTTTCTCTTCCTGCCGGACGCCCAATGGGGAAATAGGCAGGTGTCAGTCATTCTCATCCTGCGGTGCATACAGCTCGCTGAGACGAAATCTGCATAGGCCGTCCGTCCTAAACTTCTTGAGAGGCCGCATATGCGGACGCCTGAGACACACAGTGCACCTTTGCTGTCCAGCCTCCAATATCCAACTGCCACCTTCGGAGCCCTCTGGCCCCTCCCGCCCATCATCCAGTTCTCTACCAGAGGAGTGTGGCAGCACGCCGCTGGTTATCAGAGTCGTTGGAGGACAGGCAACAGAGCCCGGAGAGTGGCCCTGGTTGGCTGCTCTCGGTACCCGCGTGGGAAACACCTTCACTTCGGGATGCGCAGGCACACTCATCACGCGACGACACGTCCTCACTGCCGGCCACTGCTTCGAACCCGGCCAGCCCCACCCGACAATGGTGCGTCTTGGGGCATACAGCCTGACGAGGAGTAGCACTTCGATAGCTCCCCAGGACTTCAATATCAGGGACCGCCGTGATGGAGGCTACGTCACCAGAACCAAAGAAAACGACATCTCCATCGTTATTTTGGACCGCGAAGCTGTGTTTAATGACTATGTCCAGCCAGCGTGTTTGCCTTTCAACTACAGGAATAGAGATTTTCGAGAGGAGAAACTTGTCGTCACCGGCTGGGGAAGGACTCGCGCTCATAGCTTCCGGACCTCCGATGTCCCGATGAAGGCCGTGGTTCCAGTGACAGACACAAACAGATGTGCCTTGAGCTACACCAGACTGGCAGCGAACAAGCGACCCGTGATAGACAACAGGTCTCTGTGTGCTGGGAACGGCACTACGGATGCCTGTCTTGGTGACAGCGGCGGGCCCCTTCACTACCTCGACCTGAACGACGGCAAGTACTACGTGGTGGGCGTGGTGTCCTTTGGCGTGGGCTGCGCTAATTCAGACTTCCCCGGGGTGTACACGAGAGTCACCAGCTTCCTGGACTGGATCGAGAGAAATGTATAA

MMKVLGFFLVVLTVAKAQIIFPNEEGAPNPDPVDTELFEALEVDDRQAFSSCRTPNGEIGRCQSFSSCGAYSSLRRNLHRPSVLNFLRGRICGRLRHTVHLCCPASNIQLPPSEPSGPSRPSSSSLPEECGSTPLVIRVVGGQATEPGEWPWLAALGTRVGNTFTSGCAGTLITRRHVLTAGHCFEPGQPHPTMVRLGAYSLTRSSTSIAPQDFNIRDRRDGGYVTRTKENDISIVILDREAVFNDYVQPACLPFNYRNRDFREEKLVVTGWGRTRAHSFRTSDVPMKAVVPVTDTNRCALSYTRLAANKRPVIDNRSLCAGNGTTDACLGDSGGPLHYLDLNDGKYYVVGVVSFGVGCANSDFPGVYTRVTSFLDWIERNV

transcript_transcript/27827_Pt_Mix_transcript27827/f5p0/1391

ATGACTGCCCCACTAGCTCTGGTTGGCCGTGGGGAGCCTTCTAAGTGCGGCATTAAGGACTCCGCTCTCCTCGTCTGCTGCGACATACCCTCGAATCGAGGTGCTGTCACTTTGGCACTTACGGACATCAGCGCCCCTAGCGTCACCTTCCAATGTGGAAAAAATGCAGAAAACTTTTTATTTTTGTTTGGGCCTTCTGTCGGTGATGTGCTGCGCCCTGAGGAATTTACACCTGGCGAGATACCAGAGGTTGAGGGGAATAGGCCTGTTCGGTCTGTTCCTGGTGGATTGCACCCTAATGAATTTGCACGGCCAGAGTTTATAAACCCCCAAGCAGGAAGAGAAGCAGCAATAGGAGCAATTAACTCAAAGCGAAATGCGTGGCCATGGATGGCACTGATTGGAGAAAATGATGTAGTGGGAATCAGGTGGTTCTGCGGTGGAGCGTTGATCAACGAGCAGTGGGTCCTGACAGCCCTCCACTGCTTCTTCCAGAACACGGCAGAAGTGGTTCGTCTTGGTGAACACAACTACAACGACGACAACGATGGGGCGATCCACGAGGACTTTGGTGTGACGGAGACGGTGCTTTATCCTGACTTTACGTTTGGAGAGGGTTATCATGACCTGGCACTCCTCAAGCTGGACAAGCCAGTCGAAATACAGGAGTTCATCAGTCCTGTTTGCCTGCCGTGGGGAACGGAGAGCGACAATGATGTAACGACACGGAAGGCGACACTCACTGGTTGGGGCGACACTTTGCTGGGAGGGTTTCCAAGCTCCATCCTGCAAGAGGTCAACGTGACAGTGTTCCAGTCTGGCCAGTGTGACCGCAGCTACTCTACCTTGCCCGATTATCCAAACACCTGGCCTCAAGGAATTGGGAAGGAGATGCTGTGCGCTGGAGATCTTGATGGAGGGCGGGACGCTTGTCAGGGTGACTCAGGTGGACCTCTTGTGACTCGGGAAGCACGTGGACGCTTCGTGCTGGCGGGCGTCGTGTCGCAGGGGTACGGCTGTGGCCACAAGGACTATCCCGGACTGTACGTCAACATGCGCCATGCGCCCTATCTTGCCTGGATCAAGAAGGTCGCGTTCACCACATCTTGA

MTAPLALVGRGEPSKCGIKDSALLVCCDIPSNRGAVTLALTDISAPSVTFQCGKNAENFLFLFGPSVGDVLRPEEFTPGEIPEVEGNRPVRSVPGGLHPNEFARPEFINPQAGREAAIGAINSKRNAWPWMALIGENDVVGIRWFCGGALINEQWVLTALHCFFQNTAEVVRLGEHNYNDDNDGAIHEDFGVTETVLYPDFTFGEGYHDLALLKLDKPVEIQEFISPVCLPWGTESDNDVTTRKATLTGWGDTLLGGFPSSILQEVNVTVFQSGQCDRSYSTLPDYPNTWPQGIGKEMLCAGDLDGGRDACQGDSGGPLVTREARGRFVLAGVVSQGYGCGHKDYPGLYVNMRHAPYLAWIKKVAFTTS

transcript_transcript/27949_Pt_Mix_transcript27949/f4p0/1379

ATGAAGTGGGTGTGTGTGTTGGTGCTACTGCTGGCGCTCTCCAAAGCAACAGAAGCCAGTGCCATTGTATTTCCCGGCCAACTGGACCACGCTGAAGGGGATGACTGCCCCACTAGCTCTGGCGGGCCGGGCAAGTGTTCCCGGTCCTGCAAACACTCTGTTGGCCGTGGGGAGCCTTCTAAGTGCGGCATTAAGGACTCCGCTCTCCTCGTCTGCTGCGACATACCCTCGAATCGAGGTGCTGTCACTTTGGCACTTACGGACATCAGCGCCCCTAGCGTCACCTTCCTCGGTGATGTGCTGCGCCCTGAGGAATTTACACCTGGCGAGATACCAGAGGTTGAGGGGAATAGGCCTGTTCGGTCTGTTCCTGGTGGATTGCACCCTAATGAATTTGCACGGCCAGAGTTTATAAACCCCCAAGCAGGAAGAGAAGCAGCAATAGGAGCAATTAACTCAAAGCGAAATGCGTGGCCATGGATGGCACTGATTGGAGAAAATGATGTAGTGGGAATCAGGTGGTTCTGCGGTGGAGCGTTGATCAACGAGCAGTGGGTCCTGACAGCCCTCCACTGCTTCTTCCAGAACACGGCAGAAGTGGTTCGTCTTGGTGAACACAACTACAACGACGACAACGATGGGGCGATCCACGAGGACTTTGGTGTGACGGAGACGGTGCTTTATCCTGACTTTACGTTTGGAGAGGGTTATCATGACCTGGCACTCCTCAAGCTGGACAAGCCAGTCGAAATACAGGAGTTCATCAGTCCTGTTTGCCTGCCGTGGGGAACGGAGAGCGACAATGATGTAACGACACGGAAGGCGACACTCACTGGTTGGGGCGACACTTTGCTGGGAGGGTTTCCAAGCTCCATCCTGCAAGAGGTCAACGTGACAGTGTTCCAGTCTGGCCAGTGTGACCGCAGCTACTCTACCTTGCCCGATTATCCAAACACCTGGCCTCAAGGAATTGGGAAGGAGATGCTGTGCGCTGGAGATCTTGATGGAGGGCGGGACGCTTGTCAGGGTGACTCAGGTGGACCTCTTGTGACTCGGGAAGCACGTGGACGCTTCGTGCTGGCGGGCGTCGTGTCGCAGGGGTACGGCTGTGGCCACAAGGACTATCCCGGACTGTACGTCAACATGCGCCATGCGCCCTATCTTGCCTGGATCAAGAAGGTCGCGTTCACCACATCTTGA

MKWVCVLVLLLALSKATEASAIVFPGQLDHAEGDDCPTSSGGPGKCSRSCKHSVGRGEPSKCGIKDSALLVCCDIPSNRGAVTLALTDISAPSVTFLGDVLRPEEFTPGEIPEVEGNRPVRSVPGGLHPNEFARPEFINPQAGREAAIGAINSKRNAWPWMALIGENDVVGIRWFCGGALINEQWVLTALHCFFQNTAEVVRLGEHNYNDDNDGAIHEDFGVTETVLYPDFTFGEGYHDLALLKLDKPVEIQEFISPVCLPWGTESDNDVTTRKATLTGWGDTLLGGFPSSILQEVNVTVFQSGQCDRSYSTLPDYPNTWPQGIGKEMLCAGDLDGGRDACQGDSGGPLVTREARGRFVLAGVVSQGYGCGHKDYPGLYVNMRHAPYLAWIKKVAFTTS

transcript_transcript/27983_Pt_Mix_transcript27983/f3p0/1386

ATGAAGTGGGTGTGTGTGTTGGTGCTACTGCTGGCGCTCTCCAAAGCAACAGAAGCCAGTGCCATTGTATTTCCCGGCCAACTGGACCACGCTGAAGGGGATGACTGCCCCACTAGCTCTGGCGGGCCGGGCAAGTGTTCCCGGTCCTGCAAACACTCTGTTGGCCGTGGGGAGCCTTCTAAGTGCGGCATTAAGGACTCCGCTCTCCTCGTCTGCTGCGACATACCCTCGAATCGAGGTGCTGTCACTTTGGCACTTACGGACATCAGCGCCCCTAGCGTCACCTTCCAATGTGGAAAAAATGCAGAAAACTTTTTATTTTTGTTTGGGCCTTCTGTCGGTGATGTGCTGCGCCCTGAGGAATTTACACCTGGCGAGATACCAGAGGTTGAGGGGAATAGGCCTGTTCGGTCTGTTCCTGGTGGATTGCACCCTAATGAATTTGCACGGCCAGAGTTTATAAACCCCCAAGCAGGAAGAGAAGCAGCAATAGGAGCAATTAACTCAAAGCGAAATGCGTGGCCATGGATGGCACTGATTGGAGAAAATGATGTAGTGGGAATCAGGTGGTTCTGCGGTGGAGCGTTGATCAACGAGCAGTGGGTCCTGACAGCCCTCCACTGCTTCTTCCAGAACACGGCAGAAGTGGTTCGTCTTGGTGAACACAACTACAACGACGACAACGATGGGGCGATCCACGAGGACTTTGGTGTGACGGAGACGGTGCTTTATCCTGACTTTACGTTTGGAGAGGGTTATCATGACCTGGCACTCCTCAAGCTGGACAAGCCAGTCGAAATACAGGAGTTCATCAGTCCTGTTTGCCTGCCGTGGGGAACGGAGAGCGACAATGATGTAACGACACGGAAGGCGACACTCACTGGTTGGGGCGACACTTTGCTGGGAGGGTTTCCAAGCTCCATCCTGCAAGAGGTCAACGTGACAGTGTTCCAGTCTGGCCAGTGTGACCGCAGCTACTCTACCTTGCGCTGGATATCAAACCCTGGCCATGGAGGGCGGGACGCTTGTCAGGGTGACTCAGGTGGACCTCTTGTGACTCGGGAAGCACGTGGACGCTTCGTGCTGGCGGGCGTCGTGTCGCAGGGGTACGGCTGTGGCCACAAGGACTATCCCGGACTGTACGTCAACATGCGCCATGCGCCCTATCTTGCCTGGATCAAGAAGGTCGCGTTCACCACATCTTGA

MKWVCVLVLLLALSKATEASAIVFPGQLDHAEGDDCPTSSGGPGKCSRSCKHSVGRGEPSKCGIKDSALLVCCDIPSNRGAVTLALTDISAPSVTFQCGKNAENFLFLFGPSVGDVLRPEEFTPGEIPEVEGNRPVRSVPGGLHPNEFARPEFINPQAGREAAIGAINSKRNAWPWMALIGENDVVGIRWFCGGALINEQWVLTALHCFFQNTAEVVRLGEHNYNDDNDGAIHEDFGVTETVLYPDFTFGEGYHDLALLKLDKPVEIQEFISPVCLPWGTESDNDVTTRKATLTGWGDTLLGGFPSSILQEVNVTVFQSGQCDRSYSTLRWISNPGHGGRDACQGDSGGPLVTREARGRFVLAGVVSQGYGCGHKDYPGLYVNMRHAPYLAWIKKVAFTTS

transcript_transcript/28327_Pt_Mix_transcript28327/f5p0/1326

ATGGAGCGTACTACGTTGCTGTTACTACTTGCCTTGTTTATTGCCGCAGAGGCCAGCGCCATTATATATCCCGGCCAGCTGGACCACACTGAAGGGGATGAGTGTCCTATTGACTCCGGCAGAACAGGCACATGTTCCCGGTCATGTGGAAACTTCCTCCGCCAGGAGCAGCCTCCCAGATGTGGCATCAAAGATTCTGCTTTCCTCGTCTGTTGTGACAAAATACAAATTCACAGTTCCCTGCCAATCACTGACGTAGCTCCTCCTGCAGTAACTTTCGAATGTGGAAGAAATGCAAGAAATATGTTGACGTTATCATTCAGCTACGTGGGTGAGAAGAAAATTTATGTCGAACTTGAAGATTACGATCCCATTAAAAGTTACACTGAGAAGACAATACTGGAAAATGGTACAGAGATTGTGACGGAATACGAGGTTGGAGGCGTAGGGGGTGAGATGGCAGAGAAAAACGCGTGGCCTTGGATGGCCCTGGTTGGTGAGCGGAACGGTCACGGCATAAATTGGTTCTGTGGGGGGGTGCTGATCAACGAACAGTGGGTTCTCTCTGCCCTACACTGCTTCTTATACAAGAAGGCTGAGACTGTTCGTCTTGGTGAACATAACTATAAAGACGACAACGACGGGGCACTTCACCAAGACTTTGACGTAGTAGAGACAGTTAATTATCCTGGGTATGCTTATCCGGAGGCATACCATGACCTGGCCTTGCTTAAATTGTCTTCGAGGGTGCATATACAGGAGTTCATCAGCCCTGTGTGTCTCCCATGGGCAGCGGAGAGCGAGGTGGATATCACAGGACACCCAGCTACACTCACTGGCTATGGCGATACTGAATTTCAAGGAATACCAACCTCCTACTTGCAGGAAATCAACATGACAGTGTTTCCATCGGTCCAGTGTGACCGCAGCTACTCTAATTTGCTCCAATATGCAAACACCTGGCCTAAGGGCATCGGACAGGAGACACTGTGTGCTGGAGATCCTAATGGAGGACGAGACGCTTGCCAGGGTGACTCTGGTGGACCCCTCGTGACTCAAGACGCTCAAGGACGTTTCGTGCTGGCGGGAATCGTGTCACGAGGATACGGCTGTGGCCACAAGGATTACCCAGGATTGTACGTAAACATACGCCACAAGCCCTACCTCACCTGGATAAAAGAGATAGCGTTCACTTCCTTATGA

MERTTLLLLLALFIAAEASAIIYPGQLDHTEGDECPIDSGRTGTCSRSCGNFLRQEQPPRCGIKDSAFLVCCDKIQIHSSLPITDVAPPAVTFECGRNARNMLTLSFSYVGEKKIYVELEDYDPIKSYTEKTILENGTEIVTEYEVGGVGGEMAEKNAWPWMALVGERNGHGINWFCGGVLINEQWVLSALHCFLYKKAETVRLGEHNYKDDNDGALHQDFDVVETVNYPGYAYPEAYHDLALLKLSSRVHIQEFISPVCLPWAAESEVDITGHPATLTGYGDTEFQGIPTSYLQEINMTVFPSVQCDRSYSNLLQYANTWPKGIGQETLCAGDPNGGRDACQGDSGGPLVTQDAQGRFVLAGIVSRGYGCGHKDYPGLYVNIRHKPYLTWIKEIAFTSL

transcript_transcript/28474_Pt_Mix_transcript28474/f4p0/1360

ATGAAGTGGGTGTGTGTGTTGGTGCTACTGCTGGCGCTCTCCAAAGCAACAGAAGCCAGTGCCATTGTATTTCCCGGCCAACTGGACCACGCTGAAGGGGATGACTGCCCCACTAGCTCTGGCGGGCCGGGCAAGTGTTCCCGGTCCTGCAAACACTCTGTTGGCCGTGGGGAGCCTTCTAAGTGCGGCATTAAGGACTCCGCTCTCCTCGTCTGCTGCGACATACCCTCGAATCGAGGTGCTGTCACTTTGGCACTTACGGACATCAGCGCCCCTAGCGTCACCTTCCAATGTGGAAAAAATGCAGAAAACTTTTTATTTTTGTTTGGGCCTTCTGTCGGTGATGTGCTGCGCCCTGAGGAATTTACACCTGGCGAGATACCAGAGGTTGAGGGGAATAGGCCTGTTCGGTCTGTTCCTGGTGGATTGCACCCTAATGAATTTGCACGGCCAGAGTTTATAAACCCCCAAGCAGGAAGAGAAGCAGCAATAGGAGCAATTAACTCAAAGCGAAATGCGTGGCCATGGATGGCACTGATTGGAGAAAATGATGTAGTGGGAATCAGGTGGTTCTGCGGTGGAGCGTTGATCAACGACAACTACAACGACGACAACGATGGGGCGATCCACGAGGACTTTGGTGTGACGGAGACGGTGCTTTATCCTGACTTTACGTTTGGAGAGGGTTATCATGACCTGGCACTCCTCAAGCTGGACAAGCCAGTCGAAATACAGGAGTTCATCAGTCCTGTTTGCCTGCCGTGGGGAACGGAGAGCGACAATGATGTAACGACACGGAAGGCGACACTCACTGGTTGGGGCGACACTTTGCTGGGAGGGTTTCCAAGCTCCATCCTGCAAGAGGTCAACGTGACAGTGTTCCAGTCTGGCCAGTGTGACCGCAGCTACTCTACCTTGCCCGATTATCCAAACACCTGGCCTCAAGGAATTGGGAAGGAGATGCTGTGCGCTGGAGATCTTGATGGAGGGCGGGACGCTTGTCAGGGTGACTCAGGTGGACCTCTTGTGACTCGGGAAGCACGTGGACGCTTCGTGCTGGCGGGCGTCGTGTCGCAGGGGTACGGCTGTGGCCACAAGGACTATCCCGGACTGTACGTCAACATGCGCCATGCGCCCTATCTTGCCTGGATCAAGAAGGTCGCGTTCACCACATCTTGA

MKWVCVLVLLLALSKATEASAIVFPGQLDHAEGDDCPTSSGGPGKCSRSCKHSVGRGEPSKCGIKDSALLVCCDIPSNRGAVTLALTDISAPSVTFQCGKNAENFLFLFGPSVGDVLRPEEFTPGEIPEVEGNRPVRSVPGGLHPNEFARPEFINPQAGREAAIGAINSKRNAWPWMALIGENDVVGIRWFCGGALINDNYNDDNDGAIHEDFGVTETVLYPDFTFGEGYHDLALLKLDKPVEIQEFISPVCLPWGTESDNDVTTRKATLTGWGDTLLGGFPSSILQEVNVTVFQSGQCDRSYSTLPDYPNTWPQGIGKEMLCAGDLDGGRDACQGDSGGPLVTREARGRFVLAGVVSQGYGCGHKDYPGLYVNMRHAPYLAWIKKVAFTTS

transcript_transcript/28483_Pt_Mix_transcript28483/f3p0/1345

ATGAAGTGGGTGTGTGTGTTGGTGCTACTGCTGGCGCTCTCCAAAGCAACAGAAGCCAGTGCCATTGTATTTCCCGGCCAACTGGACCACGCTGAAGGGGATGACTGCCCCACTAGCTCTGGCGGGCCGGGCAAGTGTTCCCGGTCCTGCAAACACTCTGTTGGCCGTGGGGAGCCTTCTAAGTGCGGCATTAAGGACTCCGCTCTCCTCGTCTGCTGCGACATACCCTCGAATCGAGGTGCTGTCACTTTGGCACTTACGGACATCAGCGCCCCTAGCGTCACCTTCCAATGTGGAAAAAATGCAGAAAACTTTTTATTTTTGTTTGGGCCTTCTGTCGGTGATGTGCTGCGCCCTGAGGAATTTACACCTGGCGAGATACCAGAGGTTGAGGGGAATAGGCCTGTTCGGTCTGTTCCTGGTGGATTGCACCCTAATGAATTTGCACGGCCAGAGTTTATAAACCCCCAAGCAGGAAGAGAAGCAGCAATAGGAGCAATTAACTCAAAGCGAAATGCGTGGCCATGGATGGCACTGATTGGAGAAAATGATGTAGTGGGAATCAGGTGGTTCTGCGGTGGAGCGTTGATCAACGAGCAGTGGGTCCTGACAGCCCTCCACTGCTTCTTCCAGAACACGGCAGAAGTGGTTCGTCTTGGTGAACACAACTACAACGACGACAACGATGGGGCGATCCACGAGGACTTTGGTGTGACGGAGACGGTGCTTTATCCTGACTTTACGTTTGGAGAGGGTTATCATGACCTGGCACTCCTCAAGCTGGACAAGCCAGTCGAAATACAGGAGTTCATCAGTCCTGTTTGCCTGCCGTGGGGAACGGAGAGCGACAATGATGTAACGACACGGAAGGCGACACTCACTGGTTGGGGCGACACTTTGCTGGGAGGGTTTCCAAGCTCCATCCTGCAAGAGGTCAACGTGACAGTGTTCCAGTCTGGAGATCTTGATGGAGGGCGGGACGCTTGTCAGGGTGACTCAGGTGGACCTCTTGTGACTCGGGAAGCACGTGGACGCTTCGTGCTGGCGGGCGTCGTGTCGCAGGGGTACGGCTGTGGCCACAAGGACTATCCCGGACTGTACGTCAACATGCGCCATGCGCCCTATCTTGCCTGGATCAAGAAGGTCGCGTTCACCACATCTTGA

MKWVCVLVLLLALSKATEASAIVFPGQLDHAEGDDCPTSSGGPGKCSRSCKHSVGRGEPSKCGIKDSALLVCCDIPSNRGAVTLALTDISAPSVTFQCGKNAENFLFLFGPSVGDVLRPEEFTPGEIPEVEGNRPVRSVPGGLHPNEFARPEFINPQAGREAAIGAINSKRNAWPWMALIGENDVVGIRWFCGGALINEQWVLTALHCFFQNTAEVVRLGEHNYNDDNDGAIHEDFGVTETVLYPDFTFGEGYHDLALLKLDKPVEIQEFISPVCLPWGTESDNDVTTRKATLTGWGDTLLGGFPSSILQEVNVTVFQSGDLDGGRDACQGDSGGPLVTREARGRFVLAGVVSQGYGCGHKDYPGLYVNMRHAPYLAWIKKVAFTTS

transcript_transcript/28952_Pt_Mix_transcript28952/f8p0/1289

ATGGATTGTTCATCCCTGTGGTTATTCTGCTTCCTCGTAGCTACCGCAAGTACACAGAGTATAAGGCTACCAGACATGCCAGCTGATCAAAGTGACTGCACCACATCGTCAGGAGTGCCTGGAGAGTGTGTCGACGTTCGCTTTTGCCCAAACACTGCTCGCAGCCTTGACCATGACACGCAGGCCGCCTGTGGCTTCAATGATACATTTCCAATCGTCTGCTGCACTACAGTTGCAGACAACAGCGTTGACCGTGTTCAGGACATCTCTCCACCCACAGTTGATTTCGAATGTGGGAACAATGTAGGCAGAGTTATACGATTGCAAGAAATTAGTCCTGGACCAGAAAAATTTCTGCGAGCGGACAATGTAATTGTCTTCCCGGAAGGCACTGAGGAAAGTTCCAGGAGACCTGTTACTCGAGCTGGCTTTCTGGCAGGCGAAATTGTAACCCACATCATAGGCGGCGCCATAGTAAAGAAACATTCTTGGCCTTGGATGGCACTGATAGGAGAGAGGGACGCTGCGGAGATCAGATGGTTCTGCGGCGGAGTGTTGATCAACCAGCAGTGGGTACTCACGGCGCAGCACTGCTTCTTCCAACACACAGCTGACATAGTCCGCCTTGGTGAACACGACTACAACGACGATAACGACGGGGCGGTTCATCAGGACATCAAGGTGGCTGACGCAGTGCCGTTCCCTGACTTCGCTCATCCTAAAGCTTATCACGATCTGGAACTTCTCAAGCTGGTCACAGCAGTCACCATACAGAAGTTCATCAGTCCCGTGTGTTTACCTTGGGGCAGCGAGAGCAGCATAGAGCTATTAGGTCAACAGGCAACACTGGCAGGCTGGGGAGATACGCTTTTTGGTGGGTATCCCAGCTCCATCCTGCAAGAGGTCAACGTGACAGTGTTCTCTTCTGACCAGTGTGACCGCAGTTACTCTATCTTGTCCCATTATGCACAAACCTGGCCTCAAGGCATCGGAGAGGAGACAATGTGTGCTGGAGACCCTGAGGGAGGGCGCGACGCTTGCCAGGGTGACTCAGGCGGACCGATTATGTCTCAGGATGCTCTTGGACGATTCATGTTGGCGGGTATCGTATCGCAAGGTTACGGCTGTGGCAACAAGGAATATCCAGGACTGTACGTTAATGTACGGAACCCTCTCTACCTCGCTTGGATAAAAAAAATTGCGTTTTCTTAA

MDCSSLWLFCFLVATASTQSIRLPDMPADQSDCTTSSGVPGECVDVRFCPNTARSLDHDTQAACGFNDTFPIVCCTTVADNSVDRVQDISPPTVDFECGNNVGRVIRLQEISPGPEKFLRADNVIVFPEGTEESSRRPVTRAGFLAGEIVTHIIGGAIVKKHSWPWMALIGERDAAEIRWFCGGVLINQQWVLTAQHCFFQHTADIVRLGEHDYNDDNDGAVHQDIKVADAVPFPDFAHPKAYHDLELLKLVTAVTIQKFISPVCLPWGSESSIELLGQQATLAGWGDTLFGGYPSSILQEVNVTVFSSDQCDRSYSILSHYAQTWPQGIGEETMCAGDPEGGRDACQGDSGGPIMSQDALGRFMLAGIVSQGYGCGNKEYPGLYVNVRNPLYLAWIKKIAFS

transcript_transcript/28981_Pt_Mix_transcript28981/f4p0/1312

ATGCCAGCTGATCAAAGTGACTGCACCACATCGTCAGGAGTGCCTGGAGAGTGTGTCGACGTTCGCTTTTGCCCAAACACTGCTCGCAGCCTTGACCATGACACGCAGGCCGCCTGTGGCTTCAATGATACATTTCCAATCGTCTGCTGCACTACAGTTGCAGACAACAGCGTTGACCGTGTTCAGGACATCTCTCCACCCACAGTTGATTTCGAATGTGGGAACAATGTAGGCAGAGTTATACGATTGCAAGAAATTAGTCCTGGACCAGAAAAATTTCTGCGAGCGGACAATGTAATTGTCTTCCCGGAAGGCACTGAGGAAAGTTCCAGGAGACCTGTTACTCGAGCTGGCTTTCTGGCAGGCGAAATTGTAACCCACATCATAGGCGGCGCCATAGTAAAGAAACATTCTTGGCCTTGGATGGCACTGATAGGAGAGAGGGACGCTGCGGAGATCAGATGGTTCTGCGGCGGAGTGTTGATCAACCAGCAGTGGGTACTCACGGCGCAGCACTGCTTCTTCCAACACACAGCTGACATAGTCCGCCTTGGTGAACACGACTACAACGACGATAACGACGGGGCGGTTCATCAGGACATCAAGGTGGCTGACGCAGTGCCGTTCCCTGACTTCGCTCATCCTAAAGCTTATCACGATCTGGAACTTCTCAAGCTGGTCACAGCAGTCACCATACAGAAGTTCATCAGTCCCGTGTGTTTACCTTGGGGCAGCGAGAGCAGCATAGAGCTATTAGGTCAACAGGCAACACTGGCAGGCTGGGGAGATACGCTTTTTGGTGGGTATCCCAGCTCCATCCTGCAAGAGGTCAACGTGACAGTGTTCTCTTCTGACCAGTGTGACCGCAGTTACTCTATCTTGTCCCATTATGCACAAACCTGGCCTCAAGGCATCGGAGAGGAGACAATGTGTGCTGGAGACCCTGAGGGAGGGCGCGACGCTTGCCAGGGTGACTCAGGCGGACCGATTATGTCTCAGGATGCTCTTGGACGATTCATGTTGGCGGGTATCGTATCGCAAGGTTACGGCTGTGGCAACAAGGAATATCCAGGACTGTACGTTAATGTACGGAACCCTCTCTACCTCGCTTGGATAAAAAAAATTGCGTTTTCTTAA

MPADQSDCTTSSGVPGECVDVRFCPNTARSLDHDTQAACGFNDTFPIVCCTTVADNSVDRVQDISPPTVDFECGNNVGRVIRLQEISPGPEKFLRADNVIVFPEGTEESSRRPVTRAGFLAGEIVTHIIGGAIVKKHSWPWMALIGERDAAEIRWFCGGVLINQQWVLTAQHCFFQHTADIVRLGEHDYNDDNDGAVHQDIKVADAVPFPDFAHPKAYHDLELLKLVTAVTIQKFISPVCLPWGSESSIELLGQQATLAGWGDTLFGGYPSSILQEVNVTVFSSDQCDRSYSILSHYAQTWPQGIGEETMCAGDPEGGRDACQGDSGGPIMSQDALGRFMLAGIVSQGYGCGNKEYPGLYVNVRNPLYLAWIKKIAFS

transcript_transcript/30029_Pt_Mix_transcript30029/f6p0/1228

ATGAAGTGGGTGTGTGTGTTGGTGCTACTGCTGGCGCTCTCCAAAGCAACAGAAGCCAGTGCCATTGTATTTCCCGGCCAACTGGACCACGCTGAAGGGGATGACTGCCCCACTAGCTCTGGCGGGCCGGGCAAGTGTTCCCGGTCCTGCAAACACTCTGTTGGCCGTGGGGAGCCTTCTAAGTGCGGCATTAAGGACTCCGCTCTCCTCGTCTGCTGCGACATACCCTCGAATCGAGGTGCTGTCACTTTGGCACTTACGGACATCAGCGCCCCTAGCGTCACCTTCCAATGTGGAAAAAATGCAGAAAACTTTTTATTTTTGTTTGGGCCTTCTGTCGGTGATGTGCTGCGCCCTGAGGAATTTACACCTGGCGAGATACCAGAGGTTGAGGGGAATAGGCCTGTTCGGTCTGTTCCTGGTGGATTGCACCCTAATGAATTTGCACGGCCAGAGTTTATAAACCCCCAAGCAGGAAGAGAAGCAGCAATAGGAGCAATTAACTCAAAGCGAAATGCGTGGCCATGGATGGCACTGATTGGAGAAAATGATGTAGTGGGAATCAGGTGGTTCTGCGGTGGAGCGTTGATCAACGAGCAGTGGGTCCTGACAGCCCTCCACTGCTTCTTCCAGAACACGGCAGAAGTGGTTCGTCTTGGTGAACACAACTACAACGACGACAACGATGGGGCGATCCACGAGGACTTTGGTGTGACGGAGACGGTGCTTTATCCTGACTTTACGTTTGGAGAGGGTTATCATGACCTGGCACTCCTCAAGCTGGACAAGCCAGTCGAAATACAGGAGTTCATCAGTCCTGTTTGCCTGCCGTGGGGAACGGAGAGCGACAATGATGTAACGACACGGAAGGCGACACTCACTGGTTGGGGCGACACTTTGCTGGGAGGGTTTCCAAGCTCCATCCTGCAAGAGGTCAACGTGACAGTGTTCCAGTCTGGCCACAAGGACTATCCCGGACTGTACGTCAACATGCGCCATGCGCCCTATCTTGCCTGGATCAAGAAGGTCGCGTTCACCACATCTTGA

MKWVCVLVLLLALSKATEASAIVFPGQLDHAEGDDCPTSSGGPGKCSRSCKHSVGRGEPSKCGIKDSALLVCCDIPSNRGAVTLALTDISAPSVTFQCGKNAENFLFLFGPSVGDVLRPEEFTPGEIPEVEGNRPVRSVPGGLHPNEFARPEFINPQAGREAAIGAINSKRNAWPWMALIGENDVVGIRWFCGGALINEQWVLTALHCFFQNTAEVVRLGEHNYNDDNDGAIHEDFGVTETVLYPDFTFGEGYHDLALLKLDKPVEIQEFISPVCLPWGTESDNDVTTRKATLTGWGDTLLGGFPSSILQEVNVTVFQSGHKDYPGLYVNMRHAPYLAWIKKVAFTTS

transcript_transcript/30263_Pt_Mix_transcript30263/f14p0/1186

ATGACTGCCCCACTAGCTCTGGCGGGCCGGGCAAGTGTTCCCGGTCCTGCAAACACTCTGTTGGCCGTGGGGAGCCTTCTAAGTGCGGCATTAAGGACTCCGCTCTCCTCGTCTGCTGCGACATACCCTCGAATCGAGGTGCTGTCACTTTGGCACTTACGGACATCAGCGCCCCTAGCGTCACCTTCCGCACTGATTGGAGAAAATGATGTAGTGGGAATCAGGTGGTTCTGCGGTGGAGCGTTGATCAACGAGCAGTGGGTCCTGACAGCCCTCCACTGCTTCTTCCAGAACACGGCAGAAGTGGTTCGTCTTGGTGAACACAACTACAACGACGACAACGATGGGGCGATCCACGAGGACTTTGGTGTGACGGAGACGGTGCTTTATCCTGACTTTACGTTTGGAGAGGGTTATCATGACCTGGCACTCCTCAAGCTGGACAAGCCAGTCGAAATACAGGAGTTCATCAGTCCTGTTTGCCTGCCGTGGGGAACGGAGAGCGACAATGATGTAACGACACGGAAGGCGACACTCACTGGTTGGGGCGACACTTTGCTGGGAGGGTTTCCAAGCTCCATCCTGCAAGAGGTCAACGTGACAGTGTTCCAGTCTGGCCAGTGTGACCGCAGCTACTCTACCTTGCCCGATTATCCAAACACCTGGCCTCAAGGAATTGGGAAGGAGATGCTGTGCGCTGGAGATCTTGATGGAGGGCGGGACGCTTGTCAGGGTGACTCAGGTGGACCTCTTGTGACTCGGGAAGCACGTGGACGCTTCGTGCTGGCGGGCGTCGTGTCGCAGGGGTACGGCTGTGGCCACAAGGACTATCCCGGACTGTACGTCAACATGCGCCATGCGCCCTATCTTGCCTGGATCAAGAAGGTCGCGTTCACCACATCTTGA

MTAPLALAGRASVPGPANTLLAVGSLLSAALRTPLSSSAATYPRIEVLSLWHLRTSAPLASPSALIGENDVVGIRWFCGGALINEQWVLTALHCFFQNTAEVVRLGEHNYNDDNDGAIHEDFGVTETVLYPDFTFGEGYHDLALLKLDKPVEIQEFISPVCLPWGTESDNDVTTRKATLTGWGDTLLGGFPSSILQEVNVTVFQSGQCDRSYSTLPDYPNTWPQGIGKEMLCAGDLDGGRDACQGDSGGPLVTREARGRFVLAGVVSQGYGCGHKDYPGLYVNMRHAPYLAWIKKVAFTTS

transcript_transcript/30414_Pt_Mix_transcript30414/f2p0/1206

ATGAAGTGGGTGTGTGTGTTGGTGCTACTGCTGGCGCTCTCCAAAGCAACAGAAGCCAGTGCCATTGTATTTCCCGGCCAACTGGACCACGCTGAAGGGGATGACTGCCCCACTAGCTCTGGCGGGCCGGGCAAGTGTTCCCGGTCCTGCAAACACTCTGTTGGCCGTGGGGAGCCTTCTAAGTGCGGCATTAAGGACTCCGCTCTCCTCGTCTGCTGCGACATACCCTCGAATCGAGGTGCTGTCACTTTGGCACTTACGGACATCAGCGCCCCTAGCGTCACCTTCCAATGTGGAAAAAATGCAGAAAACTTTTTATTTTTGTTTGGGCCTTCTGTCGGTGATGTGCTGCGCCCTGAGGAATTTACACCTGGCGAGATACCAGAGGTTGAGGGGAATAGGCCTGTTCGGTCTGTTCCTGGTGGATTGCACCCTAATGAATTTGCACGGCCAGAGTTTATAAACCCCCAAGCAGGAAGAGAAGCAGCAATAGGAGCAATTAACTCAAAGCGAAATGCGTGGCCATGGATGGCACTGATTGGAGAAAATGATGTAGTGGGAATCAGGTGGTTCTGCGGTGGAGCGTTGATCAACGAGCAGTGGGTCCTGACAGCCCTCCACTGCTTCTTCCAGAACACGGCAGAAGTGGTTCGTCTTGGTGAACACAACTACAACGACGACAACGATGGGGCGATCCACGAGGACTTTGGTGTGACGGAGACGGTGCTTTATCCTGACTTTACGTTTGGAGAGGGTTATCATGACCTGGCACTCCTCAAGCTGGACAAGCCAGTCGAAATACAGGAGATGCTGTGCGCTGGAGATCTTGATGGAGGGCGGGACGCTTGTCAGGGTGACTCAGGTGGACCTCTTGTGACTCGGGAAGCACGTGGACGCTTCGTGCTGGCGGGCGTCGTGTCGCAGGGGTACGGCTGTGGCCACAAGGACTATCCCGGACTGTACGTCAACATGCGCCATGCGCCCTATCTTGCCTGGATCAAGAAGGTCGCGTTCACCACATCTTGA

MKWVCVLVLLLALSKATEASAIVFPGQLDHAEGDDCPTSSGGPGKCSRSCKHSVGRGEPSKCGIKDSALLVCCDIPSNRGAVTLALTDISAPSVTFQCGKNAENFLFLFGPSVGDVLRPEEFTPGEIPEVEGNRPVRSVPGGLHPNEFARPEFINPQAGREAAIGAINSKRNAWPWMALIGENDVVGIRWFCGGALINEQWVLTALHCFFQNTAEVVRLGEHNYNDDNDGAIHEDFGVTETVLYPDFTFGEGYHDLALLKLDKPVEIQEMLCAGDLDGGRDACQGDSGGPLVTREARGRFVLAGVVSQGYGCGHKDYPGLYVNMRHAPYLAWIKKVAFTTS

transcript_transcript/31465_Pt_Mix_transcript31465/f2p0/1129

ATGACTGCCCCACTAGCTCTGGCGGGCCGGGCAAGTGTTCCCGGTCCTGCAAACACTCTGTTGGCCGTGGGGAGCCTTCTAAGTGCGGCATTAAGGACTCCGCTCTCCTCGTCTGCTGCGACATACCCTCGAATCGAGGTGCTGTCACTTTGGCACTTACGGACATCAGCGCCCCTAGCGTCACCTTCCAATGTGGAAAAAATGCAGAAAACTTTTTATTTTTGTTTGGGCCTTCTGTCGGTGATGTGCTGCGCCCTGAGGAATTTACACCTGGCGATCCACGAGGACTTTGATAAACCCCCCAAGCAGACGGAGACGGTGCTTTATCCTGACTTTACGTTTGGAGAGGGTTATCATGACCTGGCACTCCTCAAGCTGGACAAGCCAGTCGAAATACAGGAGTTCATCAGTCCTGTTTGCCTGCCGTGGGGAACGGAGAGCGACAATGATGTAACGACACGGAAGGCGACACTCACTGGTTGGGGCGACACTTTGCTGGGAGGGTTTCCAAGCTCCATCCTGCAAGAGGTCAACGTGACAGTGTTCCAGTCTGGCCAGTGTGACCGCAGCTACTCTACCTTGCCCGAGTATCCAAACACCTGGCCTCAAGGAATTGGGAAGGAGATGCTGTGCGCTGGAGATCTTGATGGAGGGCGGGACGCTTGTCAGGGTGACTCAGGTGGACCTCTTGTGACTCGGGAAGCACGTGGACGCTTCGTGCTGGCGGGCGTCGTGTCGCAGGGGTACGGCTGTGGCCACAAGGACTATCCCGGACTGTACGTCAACATGCGCCATGCGCCCTATCTTGCCTGGATCAAGAAGGTCGCGTTCACCACATCTTGA

MTAPLALAGRASVPGPANTLLAVGSLLSAALRTPLSSSAATYPRIEVLSLWHLRTSAPLASPSNVEKMQKTFYFCLGLLSVMCCALRNLHLAIHEDFDKPPKQTETVLYPDFTFGEGYHDLALLKLDKPVEIQEFISPVCLPWGTESDNDVTTRKATLTGWGDTLLGGFPSSILQEVNVTVFQSGQCDRSYSTLPEYPNTWPQGIGKEMLCAGDLDGGRDACQGDSGGPLVTREARGRFVLAGVVSQGYGCGHKDYPGLYVNMRHAPYLAWIKKVAFTTS

transcript_transcript/4784_Pt_Mix_transcript4784/f2p0/3456

ATGGGATCTGCGGGCCGCACTCGCCAGGAGCTTCTCCGTGGTCTCCACTTCGATGACGCTCACTCCGAGAATAATGTCCATCTCAGTTTTCAAAGGCTGATGGAAGACATCTCTAGTGACGGACCCGATGTTACTCTCAAAGTAGCCAACGGTCTCTTCCTGCAGAGAGGTGCGGGTGTCATGTACAATTTCACCCAAAAGGCTCGCGCCCATTACAACAGTGAAGTCAGCACGCTTGACTTCTTGAACTCCTCTCTGAAGTCCACGGAAACCATTAACAACTGGGTGAAGGAATCCACAAATGGTATGATCCCAAATCTCCTCACGCAGTCCCTTGATCCCTTCACCACCTTTGTTGCTGTAAACACTGTCTTCTTCAACGGTAAGTGGGTCACGTCCTTCGACCCGAAGATGACCAGAGAGAGGGATTTCGATACTGGGGCAGGAAAAATTCAAGTGCCCATAATGAGTGGGACGTTCACCGTGAACTACATCGACATCCCTGAACTGGAGGCCCACATGGCTGCCTTCCCTTACAAAGGCAACCGGCAAGCCATGTACGTCATTCTGCCAACAGGTCAACCTACAGCCAATCTCGAGCCACTGGAGCGAGAGCTGTCTGCTGAGAAAATCAACTCCCTCATTGGCAAGATGACACCACTGGAGATGAGGGTAGGATTGCCTAAAATGCGCCTTTCCTTTAAGTCAAGCCTGCGAAAAACACTCAAGAATCTGGATATGGGATCCATGTTCAATCCCGCTGCAGCGAACTTCTCCCGTCTGATAGCGCAACCTGTAAGGGTGGATGATGTATTGCACGAGACCGTCATCGAAGTCTCCGAGGAGGGCACTAAGGCTGCTGCTGCTACTGGAGCAATAACAGTTCGCAGTGGTTTCTTTCATTATTTTTATGTGAATCGGCCAGCAATAATCTTCATCAGGGACGAGGTGTCTGGCGTGCCGCTCTTCTGGGGCAAACTAGTGCGGCCTGAGCCCCTCAGGACGTAG

MGSAGRTRQELLRGLHFDDAHSENNVHLSFQRLMEDISSDGPDVTLKVANGLFLQRGAGVMYNFTQKARAHYNSEVSTLDFLNSSLKSTETINNWVKESTNGMIPNLLTQSLDPFTTFVAVNTVFFNGKWVTSFDPKMTRERDFDTGAGKIQVPIMSGTFTVNYIDIPELEAHMAAFPYKGNRQAMYVILPTGQPTANLEPLERELSAEKINSLIGKMTPLEMRVGLPKMRLSFKSSLRKTLKNLDMGSMFNPAAANFSRLIAQPVRVDDVLHETVIEVSEEGTKAAAATGAITVRSGFFHYFYVNRPAIIFIRDEVSGVPLFWGKLVRPEPLRT

transcript_transcript/12188_Pt_Mix_transcript12188/f4p0/2506

ATGAGGTCTTTACTAGTAACACTCGTGACGGTGGTGGCGGTGGCAACTGGGGCTCCTCAAGGGGATAACCAAGTGGTGGTGACAAACCCACACAACTACCCGTCTCTCCTCTACATCAACTCGGCTTCCGGACTGACCCGACACACCAATAGCAGCGGCTCCCCAGTCTTCGAGCTCATCCTCTATCCTGCCAATCCTAGCCCGCCCACTCCGAGGAAGGCCGTGGTGGTGCTGGGAGGAGGGGAAGCAACGGGCACTCTCATCCTGACCCAAGCCGCGCCGCCCACAGGGGAGGTGCACATCGAGGGTGTCGTCAGTAACCTAACGCCTGGTCTGCATGGCTTCCACATCCACGAGAAGGGAAGCCTCGAGAATGGCTGTATCTCGGCGGGAGGACATTACAACCCCTATATGCGCGACCACGGCTCTCCAATACACCTAGAGCGACATGTGGGTGACCTCGGGAATATTTTGGCTGATCAGAGCGGCGTCGCGCATGTCAACATCACTGATCCGCTCGTGACGTTGGTCGGGCCGAGGTCAGTCATCGGGAGAGCTATTGTTGTGCACGCCGGGGAGGACGATCTAGGCGGTGGTGGCCATCCCTCCAGCCTCAAGACAGGCAACGCCGGGGGTCGTGTTGGCTGCGGCGTGATTGGGATTGCCTAG

MRSLLVTLVTVVAVATGAPQGDNQVVVTNPHNYPSLLYINSASGLTRHTNSSGSPVFELILYPANPSPPTPRKAVVVLGGGEATGTLILTQAAPPTGEVHIEGVVSNLTPGLHGFHIHEKGSLENGCISAGGHYNPYMRDHGSPIHLERHVGDLGNILADQSGVAHVNITDPLVTLVGPRSVIGRAIVVHAGEDDLGGGGHPSSLKTGNAGGRVGCGVIGIA

transcript_transcript/18611_Pt_Mix_transcript18611/f13p0/1948

ATGGTGATGGCTGATGGTGATGGTGGCTTACCTCTCCGCAGGATGCATCGACACGTGCTGACGGGACTGCTGCTGTTGATGACGGCCTCCTGCATGGCCATCCCTGTGCCTGCGGAAGGTGACTATGACCTCAACTATGTACTGAGCAACCTGACGGCGCTGTGGGAGGAAGTGGCACAGCTGCGGCATGACGTGGAGGATCATCGACACGGCTACCCTCCCCACAGCCAGGTGACCCAGGAGGTGGACACGCTGCAACACGAGATGGTGGTACACGCCTACCAACGCCCACACGAGGGCCACAACGACCACCATGACCCTAACGCCCTCCAACACCTGGAGCACTTCCACGAGCATCACCACGACGACCACAACCACCACGATGCCGACGACCACCAAGGCCACGAACACGGCCACGACCACAGCCACGACCACGGCCATGACCACGATCACGGACATGATCACAGCCATGACCACGATCACGGACATGATCACAGCCATGACCACGACCACGGCCACGACCACGGCCACGAGCATGGCCATCACCACCACGATGGAGACCACGACCACACTCACGACCACAAACACGATGATAACGCCGACAACCAGAGTGATGAGGATAACAGCGGAGAAGACGACGACTCAGACAACAGTGGCGAGGCACACGACCATGATGGACACGACCACCACCATGGCGGCCATGATCACCACCACCGCCAACACGACCACCACCACCATGGTGGCCACGAAAATCACAACCATGGCAAAGGACACGACCACTCTGACGACACCCACGACCACAAAAGTCACCACCACAGCAAGGACACCCACCAACACGACAGCGACCACCACCACGATCACGACCACACAGGCGAAATCCACGACCACGAGGGACATGACCACAGCCAGGACCACAGTGAACATGGCGAAGGGCACGACCATTCTGGCGACGTGCACAGCCACGAGGGGCACGACCACAGCCAGGACACCCACCAACACGACAACGACCACCACCACCATGACGACCACCACCACCACCACCATGAGAAGGACGCCCATGACACACACGGCCTCCACGATCATTTTGAAGGAGCTCCTCCCCCCGCCAGCCGCCCCTCTCTGGTCGGCCCCGTGCTGGCCCCTAAGCTCGGCCTGGCTCTAGCCCCGGGGAGCGAACAGGCGGCGGCAGACCAGGGGGAGGAGTGGGCGCACGCCTCCTGCAAGCTGGAGCCCAACGCTGAGCTGGCGGATGGCACCGTGAGGGGAAATGTCATCATCTCGCGGAAGAAGGACGAGTCTGGCCCTGTCTTCTTCCACCTCGACCTCCAAGGCTTCCAACCCTCGCAAGGAAGAATACACGGGTTCCACATCCACTCCTTCCCCATCACTGACAACAAGTGCAGCACCGCCGGGGGACACTTTAACCCCTACGGCACCACCCACGGAGGCCCTGACAGTGACACAAGACACGTGGGCGATCTTGGTAACATCGAGGTGGACAGCCACGGCTACCTGGAGAACCACCTCTTGACTGATGATCGTGTGGCACTCAGCGGGCCAGCCAACATTGCCGCCAAATCTATCGTGATACACGCCGGTGAGGACGACCTAGGGCTTGGCGGGGACAGTGGCAGCCTCACTACGGGCAATGCTGGGGGTCGCCTCGCCTGCTGTACCATCCACATCCAGGCCGAGGGTTCCTACAGGTTCAAAGGATAA

MVMADGDGGLPLRRMHRHVLTGLLLLMTASCMAIPVPAEGDYDLNYVLSNLTALWEEVAQLRHDVEDHRHGYPPHSQVTQEVDTLQHEMVVHAYQRPHEGHNDHHDPNALQHLEHFHEHHHDDHNHHDADDHQGHEHGHDHSHDHGHDHDHGHDHSHDHDHGHDHSHDHDHGHDHGHEHGHHHHDGDHDHTHDHKHDDNADNQSDEDNSGEDDDSDNSGEAHDHDGHDHHHGGHDHHHRQHDHHHHGGHENHNHGKGHDHSDDTHDHKSHHHSKDTHQHDSDHHHDHDHTGEIHDHEGHDHSQDHSEHGEGHDHSGDVHSHEGHDHSQDTHQHDNDHHHHDDHHHHHHEKDAHDTHGLHDHFEGAPPPASRPSLVGPVLAPKLGLALAPGSEQAAADQGEEWAHASCKLEPNAELADGTVRGNVIISRKKDESGPVFFHLDLQGFQPSQGRIHGFHIHSFPITDNKCSTAGGHFNPYGTTHGGPDSDTRHVGDLGNIEVDSHGYLENHLLTDDRVALSGPANIAAKSIVIHAGEDDLGLGGDSGSLTTGNAGGRLACCTIHIQAEGSYRFKG

transcript_transcript/19443_Pt_Mix_transcript19443/f50p0/1891

ATGCATCGACACGTGCTGACGGGACTGCTGCTGTTGATGACGGCCTCCTGCATGGCCATCCCTGTGCCTGCGGAAGGTGACTATGACCTCAACTATGTACTGAGCAACCTGACGGCGCTGTGGGAGGAAGTGGCACAGCTGCGGCATGACGTGGAGGATCATCGACACGGCTACCCTCCCCACAGCCAGGTGACCCAGGAGGTGGACACGCTGCAACACGAGATGGTGGTACACGCCTACCAACGCCCACACGAGGGCCACAACGACCACCATGACCCTAACGCCCTCCAACACCTGGAGCACTTCCACGAGCATCACCACGACGACCACAACCACCACGATGCCGACGACCACCAAGGCCACGAACACGGCCACGACCACAGCCACGACCACGGCCATGACCACGATCACGGACATGATCACAGCCATGACCACGATCACGGACATGATCACAGCCATGACCACGACCACGGCCACGACCACGGCCACGAGCATGGCCATCACCACCACGATGGAGACCACGACCACACTCACGACCACAAACACGATGATAACGCCGACAACCAGAGTGATGAGGATAACAGCGGAGAAGACGACGACTCAGACAACAGTGGCGAGGCACACGACCATGATGGACACGACCACCACCATGGCGGCCATGATCACCACCACCGCCAACACGACCACCACCACCATGGTGGCCACGAAAATCACAACCATGGCAAAGGACACGACCACTCTGACGACACCCACGACCACAAAAGTCACCACCACAGCAAGGACACCCACCAACACGACAGCGACCACCACCACGATCACGACCACACAGGCGAAATCCACGACCACGAGGGACATGACCACAGCCAGGACCACAGTGAACATGGCGAAGGGCACGACCATTCTGGCGACGTGCACAGCCACGAGGGGCACGACCACAGCCAGGACACCCACCAACACGACAACGACCACCACCACCATGACGACCACCACCACCACCACCATGAGAAGGACGCCCATGACACACACGGCCTCCACGATCATTTTGAAGGAGCTCCTCCCCCCGCCAGCCGCCCCTCTCTGGTCGGCCCCGTGCTGGCCCCTAAGCTCGGCCTGGCTCTAGCCCCGGGGAGCGAACAGGCGGCGGCAGACCAGGGGGAGGAGTGGGCGCACGCCTCCTGCAAGCTGGAGCCCAACGCTGAGCTGGCGGATGGCACCGTGAGGGGAAATGTCATCATCTCGCGGAAGAAGGACGAGTCTGGCCCTGTCTTCTTCCACCTCGACCTCCAAGGCTTCCAACCCTCGCAAGGAAGAATACACGGGTTCCACATCCACTCCTTCCCCATCACTGACAACAAGTGCAGCACCGCCGGGGGACACTTTAACCCCTACGGCACCACCCACGGAGGCCCTGACAGTGACACAAGACACGTGGGCGATCTTGGTAACATCGAGGTGGACAGCCACGGCTACCTGGAGAACCACCTCTTGACTGATGATCGTGTGGCACTCAGCGGGCCAGCCAACATTGCCGCCAAATCTATCGTGATACACGCCGGTGAGGACGACCTAGGGCTTGGCGGGGACAGTGGCAGCCTCACTACGGGCAATGCTGGGGGTCGCCTCGCCTGCTGTACCATCCACATCCAGGCCGAGGGTTCCTACAGGTTCAAAGGATAA

MHRHVLTGLLLLMTASCMAIPVPAEGDYDLNYVLSNLTALWEEVAQLRHDVEDHRHGYPPHSQVTQEVDTLQHEMVVHAYQRPHEGHNDHHDPNALQHLEHFHEHHHDDHNHHDADDHQGHEHGHDHSHDHGHDHDHGHDHSHDHDHGHDHSHDHDHGHDHGHEHGHHHHDGDHDHTHDHKHDDNADNQSDEDNSGEDDDSDNSGEAHDHDGHDHHHGGHDHHHRQHDHHHHGGHENHNHGKGHDHSDDTHDHKSHHHSKDTHQHDSDHHHDHDHTGEIHDHEGHDHSQDHSEHGEGHDHSGDVHSHEGHDHSQDTHQHDNDHHHHDDHHHHHHEKDAHDTHGLHDHFEGAPPPASRPSLVGPVLAPKLGLALAPGSEQAAADQGEEWAHASCKLEPNAELADGTVRGNVIISRKKDESGPVFFHLDLQGFQPSQGRIHGFHIHSFPITDNKCSTAGGHFNPYGTTHGGPDSDTRHVGDLGNIEVDSHGYLENHLLTDDRVALSGPANIAAKSIVIHAGEDDLGLGGDSGSLTTGNAGGRLACCTIHIQAEGSYRFKG

transcript_transcript/19513_Pt_Mix_transcript19513/f20p0/1889

ATGGTGATGGCTGATGGTGATGGTGGCTTACCTCTCCGCAGGATGCATCGACACGTGCTGACGGGACTGCTGCTGTTGATGACGGCCTCCTGCATGGCCATCCCTGTGCCTGCGGAAGGTGACTATGACCTCAACTATGTACTGAGCAACCTGACGGCGCTGTGGGAGGAAGTGGCACAGCTGCGGCATGACGTGGAGGATCATCGACACGGCTACCCTCCCCACAGCCAGGTGACCCAGGAGGTGGACACGCTGCAACACGAGATGGTGGTACACGCCTACCAACGCCCACACGAGGGCCACAACGACCACCATGACCCTAACGCCCTCCAACACCTGGAGCACTTCCACGAGCATCACCACGACGACCACAACCACCACGATGCCGACGACCACCAAGGCCACGAACACGGCCACGACCACAGCCACGACCACGGCCATGACCACGATCACGGACATGATCACAGCCATGACCACGATCACGGACATGATCACAGCCATGACCACGACCACGGCCACGACCACGGCCACGAGCATGGCCATCACCACCACGATGGAGACCACGACCACACTCACGACCACAAACACGATGATAACGCCGACAACCAGAGTGATGAGGATAACAGCGGAGAAGACGACGACTCAGACAACAGTGGCGAGGCACACGACCATGATGGACACGACCACCACCATGGCGGCCATGATCACCACCACCGCCAACACGATCACCACCACCATGGTGGCCACGAAAATCACAACCATGGCAAAGGACACGACCACTCTGGCAACACCCACGACCACAAAAGTCACGACCACAGCAAGGACACCCACCAACACGACAGCGACCACCACCACGATCACGACCACACAGGCGAAATCCACAACCACGAGGGACATGACCACAGCCAGGACCACAGTGAACATGGCGAAGGGCACGACCATTCTGGCGACGTGCACAGCCACGAGGGGCACGACCATAGCCAGGACACCCACCAACACGACAACGACCACCACCACCACCACCATGAGAAGGACGCCCATGACACACACGGCCTTCACGATCATTTTGAAGGAGTTCCTCCCCCCGCCAGCCGCCCCTCTCTGGTCGGCCCCGTGCTGGCCCCTAAGCTCGGCCTGGCTCTAGCCCCGGGGAGCGAACAGGCGGCGGCACACCAGGGGGAGGAGTGGGCGCACGCCTCCTGCAAGCTGGAGCCCAACGCTGAGCTGGCGGATAGCACCGTGAGGGGAAATGTCATCATCTCGCGGAAGAAGGACGAGTCTGGCCCTGTCTTCTTCCACCTCGACCTCCAAGGCTTCCAACCCTCGCAAGGAAGAATACACGGGTTCCACATCCACTCCTTCCCCATCACTGACAACAAGTGCAGCACCGCCGGGGGACACTTTAACCCCTACGGCACCACCCACGGAGGCCCTGACAGTGACACAAGACACGTGGGCGACCTTGGTAACATCGAGGTGGACAGCCACGGCTACCTGGAGAACCACCTCTTGACTGATGATCGTGTGGCACTCAGCGGGCCAGCCAACATTGCCGCCAAATCTATCGTGATACACGCCGGTGAGGACGACCTAGGGCTTGGCGGGGACAGTGGCAGCCTCACTACGGGCAATGCTGGGGGTCGCCTCGCCTGCTGTACCATCCACATCCAGGCCGAGGTTTCCTACAGGTTCAAAGGATAA

MVMADGDGGLPLRRMHRHVLTGLLLLMTASCMAIPVPAEGDYDLNYVLSNLTALWEEVAQLRHDVEDHRHGYPPHSQVTQEVDTLQHEMVVHAYQRPHEGHNDHHDPNALQHLEHFHEHHHDDHNHHDADDHQGHEHGHDHSHDHGHDHDHGHDHSHDHDHGHDHSHDHDHGHDHGHEHGHHHHDGDHDHTHDHKHDDNADNQSDEDNSGEDDDSDNSGEAHDHDGHDHHHGGHDHHHRQHDHHHHGGHENHNHGKGHDHSGNTHDHKSHDHSKDTHQHDSDHHHDHDHTGEIHNHEGHDHSQDHSEHGEGHDHSGDVHSHEGHDHSQDTHQHDNDHHHHHHEKDAHDTHGLHDHFEGVPPPASRPSLVGPVLAPKLGLALAPGSEQAAAHQGEEWAHASCKLEPNAELADSTVRGNVIISRKKDESGPVFFHLDLQGFQPSQGRIHGFHIHSFPITDNKCSTAGGHFNPYGTTHGGPDSDTRHVGDLGNIEVDSHGYLENHLLTDDRVALSGPANIAAKSIVIHAGEDDLGLGGDSGSLTTGNAGGRLACCTIHIQAEVSYRFKG

transcript_transcript/20733_Pt_Mix_transcript20733/f12p0/1783

ATGACCCTAACGCCCTCCAACACCTGGAGCACTTCCACGAGCATCACCACGACGACCACAACCACCACGATGCCGACGACCACCAAGGCCACGAACACGGCCACGACCACAGCCACGACCACGGCCATGACCACGATCACGGACATGATCACAGCCATGACCACGATCACGGACATGATCACAGCCATGACCACGACCACGGCCACGACCACGGCCACGAGCATGGCCATCACCACCACGATGGAGACCACGACCACACTCACGACCACAAACACGATGATAACGCCGACAACCAGAGTGATGAGGATAACAGCGGAGAAGACGACGACTCAGACAACAGTGGCGAGGCACACGACCATGATGGACACGACCACCACCATGGCGGCCATGATCACCACCACCGCCAACACGACCACCACCACCATGGTGGCCACGAAAATCACAACCATGGCAAAGGACACGACCACTCTGACGACACCCACGACCACAAAAGTCACCACCACAGCAAGGACACCCACCAACACGACAGCGACCACCACCACGATCACGACCACACAGGCGAAATCCACGACCACGAGGGACATGACCACAGCCAGGACCACAGTGAACATGGCGAAGGGCACGACCATTCTGGCGACGTGCACAGCCACGAGGGGCACGACCACAGCCAGGACACCCACCAACACGACAACGACCACCACCACCATGACGACCACCACCACCACCACCATGAGAAGGACGCCCATGACACACACGGCCTCCACGATCATTTTGAAGGAGTGGGCGCACGCCTCCTGCAAGCTGGAGCCCAACGCTGAGCTGGCGGATGGCACCGTGAGGGGAAATGTCATCATCTCGCGGAAGAAGGACGAGTCTGGCCCTGTCTTCTTCCACCTCGACCTCCAAGGCTTCCAACCCTCGCAAGGAAGAATACACGGGTTCCACATCCACTCCTTCCCCATCACTGACAACAAGTGCAGCACCGCCGGGGGACACTTTAACCCCTACGGCACCACCCACGGAGGCCCTGACAGTGACACAAGACACGTGGGCGATCTTGGTAACATCGAGGTGGACAGCCACGGCTACCTGGAGAACCACCTCTTGACTGATGATCGTGTGGCACTCAGCGGGCCAGCCAACATTGCCGCCAAATCTATCGTGATACACGCCGGTGAGGACGACCTAGGGCTTGGCGGGGACAGTGGCAGCCTCACTACGGGCAATGCTGGGGGTCGCCTCGCCTGCTGTACCATCCACATCCAGGCCGAGGGTTCCTACAGGTTCAAAGGATAA

MHRHVLTGLLLLMTASCMAIPVPAEGDYDLNYVLSNLTALWEEVAQLRHDVEDHRHGYPPHSQVTQEVDTLQHEMVVHAYQRPHEGHNDHHDPNALQHLEHFHEHHHDDHNHHDADDHQGHEHGHDHSHDHGHDHDHGHDHSHDHDHGHDHSHDHDHGHDHGHEHGHHHHDGDHDHTHDHKHDDNADNQSDEDNSGEDDDSDNSGEAHDHDGHDHHHGGHDHHHRQHDHHHHGGHENHNHGKGHDHSDDTHDHKSHHHSKDTHQHDSDHHHDHDHTGEIHDHEGHDHSQDHSEHGEGHDHSGDVHSHEGHDHSQDTHQHDNDHHHHDDHHHHHHEKDAHDTHGLHDHFEGVGARLLQAGAQR

transcript_transcript/21834_Pt_Mix_transcript21834/f6p0/1718

ATGACCCTAACGCCCTCCAACACCTGGAGCACTTCCACGAGCATCACCACGACGACCACAACCACCACGATGCCGACGACCACCAAGGCCACGAACACGGCCACGACCACAGCCACGACCACGGCCATGACCACGATCACGGACATGATCACAGCCATGACCACGATCACGGACATGATCACAGCCATGACCACGACCACGGCCACGACCACGGCCACGAGCATGGCCATCACCACCACGATGGAGACCACGACCACACTCACGACCACAAACACGATGATAACGCCGACAACCAGAGTGATGAGGATAACAGCGGAGAAGACGACGACTCAGACAACAGTGGCGAGGCACACGACCATGATGGACACGACCACCACCATGGCGGCCATGATCACCACCACCGCCAACACGATCACCACCACCATGGTGGCCACGAAAATCACAACCATGGCAAAGGACACGACCACTCTGGCAACACCCACGACCACAAAAGTCACGACCACAGCAAGGACACCCACCAACACGACAGCGACCACCACCACGATCACGACCACACAGGCGAAATCCACAACCACGAGGGACATGACCACAGCCAGGACCACAGTGAACATGGCGAAGGGCACGACCATTCTGGCGACGTGCACAGCCACGAGGGGCACGACCATAGCCAGGACACCCACCAACACGACAACGACCACCACCACCACCACCATGAGAAGGACGCCCATGACACACACGGCCTTCACGATCATTTTGAAGGAGTGGGCGCACGCCTCCTGCAAGCTGGAGCCCAACGCTGAGCTGGCGGATAGCACCGTGAGGGGAAATGTCATCATCTCGCGGAAGAAGGACGAGTCTGGCCCTGTCTTCTTCCACCTCGACCTCCAAGGCTTCCAACCCTCGCAAGGAAGAATACACGGGTTCCACATCCACTCCTTCCCCATCACTGACAACAAGTGCAGCACCGCCGGGGGACACTTTAACCCCTACGGCACCACCCACGGAGGCCCTGACAGTGACACAAGACACGTGGGCGACCTTGGTAACATCGAGGTGGACAGCCACGGCTACCTGGAGAACCACCTCTTGACTGATGATCGTGTGGCACTCAGCGGGCCAGCCAACATTGCCGCCAAATCTATCGTGATACACGCCGGTGAGGACGACCTAGGGCTTGGCGGGGACAGTGGCAGCCTCACTACGGGCAATGCTGGGGGTCGCCTCGCCTGCTGTACCATCCACATCCAGGCCGAGGTTTCCTACAGGTTCAAAGGATAA

MTLTPSNTWSTSTSITTTTTTTTMPTTTKATNTATTTATTTAMTTITDMITAMTTITDMITAMTTTTATTTATSMAITTTMETTTTLTTTNTMITPTTRVMRITAEKTTTQTTVARHTTMMDTTTTMAAMITTTANTITTTMVATKITTMAKDTTTLATPTTTKVTTTARTPTNTTATTTTITTTQAKSTTTRDMTTARTTVNMAKGTTILATCTATRGTTIARTPTNTTTTTTTTTMRRTPMTHTAFTIILKEWAHASCKLEPNAELADSTVRGNVIISRKKDESGPVFFHLDLQGFQPSQGRIHGFHIHSFPITDNKCSTAGGHFNPYGTTHGGPDSDTRHVGDLGNIEVDSHGYLENHLLTDDRVALSGPANIAAKSIVIHAGEDDLGLGGDSGSLTTGNAGGRLACCTIHIQAEVSYRFKG

transcript_transcript/26748_Pt_Mix_transcript26748/f184p0/1401

ATGGGCAAGGGCGTGATGTACGTGGTGGCAGCCGTGGTGTTCATAGGCATCGGGGCACTGGTGGCCGGCCTCAGCATCTGGTACCATCACCCTAATCTCAACAAGGAACCCTTGATCTCTGAGATCCGGTACGCCCGGTGTGTTCTTCAGTCCCCTAACGGCATTGTGTCGGGAACTTTGTACCTGCAGCAGTCTCGTTCCTTCTCACCTGTCCTGATCAGTGGTAACATCACAGGCTTGAGTAAAGGCAAGCACGGCTTCCACATCCATCAGTGGGGAGTTGAGAACAATGATTGCAAATCCACCGGTGGTCATTACAATCCTCTTGGCTACAGCCACTCTGCCCCCAACGCAACAGAGCGGCACATGGGTGACCTCGGCAACGTGGAGGCAAAGGCTAGTAGCACCAAGGGGGACATCATCGCCAATGTAGGGATAACGGACAAAGTTTTGACATTGTCTGGCCAGTACTCCATCGTAGGGCGGGCCATCGTTGTGCATGAAGGCGAGGACGATCTCGGCTTGGGAGGCGACTCTGGATCACTTAAGACGGGCAACGCTGGGGGCAGGGTGGCATGCTGTACCTTGTATTTGTCCGCGGCACCTAAACCATAA

MGKGVMYVVAAVVFIGIGALVAGLSIWYHHPNLNKEPLISEIRYARCVLQSPNGIVSGTLYLQQSRSFSPVLISGNITGLSKGKHGFHIHQWGVENNDCKSTGGHYNPLGYSHSAPNATERHMGDLGNVEAKASSTKGDIIANVGITDKVLTLSGQYSIVGRAIVVHEGEDDLGLGGDSGSLKTGNAGGRVACCTLYLSAAPKP

transcript_transcript/27856_Pt_Mix_transcript27856/f8p0/1363

ATGGGCAAGGGCGTGATGTACGTGGTGGCAGCCGTGGTGTTCATAGGCATCGGGGCACTGGTGGCCGGCCTCAGCATCTGGTACCATCACCCTAATCTCAACAAGGAACCCTTGATCTCTGAGATCCGGTACGCCCGGTGTGTTCTTCAGTCCCCTAACGGCATTGTGTCGGGAACTTTGTACCTGCAGCAGTCTCGTTCCTTCTCACCTGTCCTGATCAGTGGTAACATCACAGGCTTGAGTAAAGGCAAGCACGGCTTCCACATCCATCAGTGGGGAGTTGAGAACAATGATTGCAAATCCACCGGTGGTCATTACAATCCTCTTGGCTACAGCCACACTGCCCCCAACGCACCAGAGCGGGACATCATCGCCAATGTAGGGATAACGGACAAAGTTTTGACATTGTCTGGCCAGTACTCCATCGTAGGGCGGGCCATCGTTGTGCATGAAGGCGAGGACGATCTCGGCTTGGGAGGCGACTCTGGATCACTCAAGACGGGCAACGCTGGGGGCAGGGTGGCATGCTGTACCTTGTATTTGTCCGCGGCACCTAAACCATAA

MGKGVMYVVAAVVFIGIGALVAGLSIWYHHPNLNKEPLISEIRYARCVLQSPNGIVSGTLYLQQSRSFSPVLISGNITGLSKGKHGFHIHQWGVENNDCKSTGGHYNPLGYSHTAPNAPERDIIANVGITDKVLTLSGQYSIVGRAIVVHEGEDDLGLGGDSGSLKTGNAGGRVACCTLYLSAAPKP

transcript_transcript/32942_Pt_Mix_transcript32942/f13p0/974

ATGAATCTGTATGAGTTTAACGTGGTAGAGGCCGGTAAAGGGTTGCTGATTAAAATGCAGCTTCTCCTACTAGTGTGTCTTGCAGTCAGTGCTTCCTGCGGCGCGGTAAGTAGACCAGATGCAAAGCTACCCCGAGTGACGCCGAGTGAAGTGCACGAAGTTCCTGACGCGGTAGTGAACTTTGTGCCTGGTAATAATAATGTGTACGGGGAACTGCATCTTTTCCGAACTCAATATTCTGGTGTCTTGATCCGAGGGAGAGTGTCCGGTCTGAGTCCAGGTCAACACGGCTTCCATGTGCATGCAGTCGGTGATCTCTCAGGGAAGTGCACGGCCGCAGGTGGTCACTTCAATCCCTACATGACTACCCACGGCTCCCCCTATGACGCTAACCGCCACGCTGGAGACCTTGGCAACATTCAGGCTGACCTTCATGGCAACGCTGACGTGTATATTCATGACTTGGTGATCTCCCTCGACCCTGCCTCCCCTGCGTACATCGGCAACCTGGCCATAGTGGTTCACCAGGGCGAGGATGATCTAGGACGTGGAGGCAATGCGGATAGCTTGAAAACTGGCAATGCTGGAGGACGTGCTGGGTGTGGACTCATCATACCTCTGTGA

MNLYEFNVVEAGKGLLIKMQLLLLVCLAVSASCGAVSRPDAKLPRVTPSEVHEVPDAVVNFVPGNNNVYGELHLFRTQYSGVLIRGRVSGLSPGQHGFHVHAVGDLSGKCTAAGGHFNPYMTTHGSPYDANRHAGDLGNIQADLHGNADVYIHDLVISLDPASPAYIGNLAIVVHQGEDDLGRGGNADSLKTGNAGGRAGCGLIIPL

transcript_transcript/33404_Pt_Mix_transcript33404/f52p0/914

ATGAATCTGTATGAGTTTAACGTGGTAGAGGCCGGTAAAGGGTTGCTGATTAAAATGCAGCTTCTCCTACTAGTGTGTCTTGCAGTCAGTGCTTCCTGCGGCGCGGTGCACGAAGTTCCTGACGCGGTAGTGAACTTTGTGCCTGGTAATAATAATGTGTACGGGGAACTGCATCTTTTCCGAACTCAATATTCTGGTGTCTTGATCCGAGGGAGAGTGTCCGGTCTGAGTCCAGGTCAACACGGCTTCCATGTGCATGCAGTCGGTGATCTCTCAGGGAAGTGCACGGCCGCAGGTGGTCACTTCAATCCCTACATGACTACCCACGGCTCCCCCTATGACGCTAACCGCCACGCTGGAGACCTTGGCAACATTCAGGCTGACCTTCATGGCAACGCTGACGTGTATATTCATGACTTGGTGATCTCCCTCGACCCTGCCTCCCCTGCGTACATCGGCAACCTGGCCATAGTGGTTCACCAGGGCGAGGATGATCTAGGACGTGGAGGCAATGCGGATAGCTTGAAAACTGGCAATGCTGGAGGACGTGCTGGGTGTGGACTCATCATACCTCTGTGA

MNLYEFNVVEAGKGLLIKMQLLLLVCLAVSASCGAVHEVPDAVVNFVPGNNNVYGELHLFRTQYSGVLIRGRVSGLSPGQHGFHVHAVGDLSGKCTAAGGHFNPYMTTHGSPYDANRHAGDLGNIQADLHGNADVYIHDLVISLDPASPAYIGNLAIVVHQGEDDLGRGGNADSLKTGNAGGRAGCGLIIPL

transcript_transcript/3437_Pt_Mix_transcript3437/f2p0/3765

ATGTGGAGACGAGGAGGAGGAAGAAGATGTGGAGGAGGAGGAGGAGGAGGAGGAAGAATGAGAAGAGGAAAAGGAGGAGGAGGATTACTAATGGCCTTGTGTTTTCTCTGCGGAGTGCAGACATCCTACACAACATCCCCTGTCCTCCTGTACTCCACTATGTCCTGGGGCGGCCTGCGGGGGAACATTACCTTCTCCTGGGGCGGCGAGGGCACCAATGTGACGGTGACGGCGGCGCTGGAGGAGGCGGGGACAGATCTTGAAGGGGAGACTAAAGAATATGACTGGGCTGTATATGATTGGCCCGTTCGCTTTGATACTAACAAAAGATGCGGTTTATCGGATCTTGGTACGAAAAAAAGGGACCTCTCTCGGCTCCTTGGCAAGCTATCCCTCCCGCAGGTGGATGGGCCCCAGGTGTTCGAGACTGACCAGCTGGCCTTGGTCGGGGAGGAGGCCATCTGGGGGCGGAGCATCAAGATTACTGGCCCGAAAACCACCTGCGCTAATTTACACGGAGTGGGCGGCGAGAGGACATACGAGGGGCGGTTCCAGGCTCCTGTGGGCGGGTCGGTGTGGCTGAGGACCTGGGCGTGGGACGTTGGGACAGGGAACAACTCAAAGAAGGGAATACAAACCACAATCTTTACTGACGTCTACCACACAGCAGCTGATGACCCTCCCAGCGGTGATCACAGCTGGTCCCTCTTCATCACCGATATCCTGGACGAGAAGGAGAACCGACCATCTTGCAATTTCTTGAGTCGCATTTATGATCCTGAGGGGAGAGAGAAGTGTGACGAGTCTGGCTGCCCAATGGGGGACTTAACGGCAGCTCATGGGCCCCTAAGAGTGTCCGCGGCCAGGTCACGATTCTCACGCAAGATGTACACCTCCACCAAGATGACCCTGCCTGACCTGACGGGGCCGCGGAAGCTGTACCTGGCTGTGATGGGCGCCCTTCACCCTGATCACCTGTGGGCGTGCACGAAGCTCAGACCAGTGCTGCCGAAGAAGGCCCGGGCGGTGTTCGATGCCCTGGGAGTGACTGGCAAAATCACACTCACACAGGCGTCACTTTTTGCCCCCACGGACGTCACCCTGGCTTTGGAGGGCCTGGGGGGAATGGCCGGGGGGTTCCACGTGCATGAGCTCCCTGCGTTACCTCAAAGGGACCCCGGGGTATCGCACTGCTCCGCCACAAAGGGCCACTATAACCCGTATGGTGTGGACGTGGCTACATCTCCTGAGCCTGGCCTCGGTGCGCACGATCAGTATGAATTGGGTGACTTGAGCGGGAAACACGGCATGCTACTGGGTCTAGAGGACGCTCAAGCTACTGTCACAGACCACAACTTGCCTCTCTTCGGCCCTCGTTCAGTGCTAGGTCGTGGTTTGGTCATACACAAGGCGGAGGGCGCGAGGTGGGTCTGTGCTAACCTCCGCCCCACTACACCACAGATCCGCGCCGCTGTGACCTTCAGATACCCGTTAGTTGGTGAGATGATATTTGAGCAAGAGGCAGATGACCCTCATTCCGATACCTCCGTGCTGGTCACCTACCTCGTGTACTCTGACGGCAGCAGGAACACCACAGGGGACCACCGCTGGCACGTGCACCTCCACCCCCCAGGCAGGGACTTCTATAACTGGACCAAACGTTGCGTCAGTGCTGGCCCGCGGTACAACCCTTTCAAGGTGAGCACACACGAGAGGCAATACAAGGGATGCAGCGTGGACACCCCTGCCAAATGTGAACTGGGGGACCTCTCTGGGCGTCACGGGAACGTCAGGGTCTCGGGGACAGTGAAGGGCGCCCCGGAGACACAAAGGATGATGACGGACACGAATCTTCCTCTCTCGGGGCCTCACAGCATCTTAGGACACTCCATTGTGATTCATGATGACTTTGCTCCAAAACATCGTGGTGATAGGATGGCCTGCATGAGCATCCACCGCATCTTCCGTCACAAGGGCGTGGTGAGCAAGTGGCACGCGACGAGGGGGGCGGGGTCGGTGGAGGGCAAGATTGAGTTCGTGCAGGAGTCAGAGTATGACCTGACCAACACTGAGGTGGAGCTGAGGGGCCTGGCGGGGATGGCCGGGGGGTACCACGTGCATATGGTCCCAGTAGAAGCAGAGTTGGAGTTCCCCTGTGAAGCCTCCACAACACTGGGGCACTACAACCCTCTGAACATAACGCCCAGTGACTCTCCAGCACCCACGAAGGGCACGGATGACCTGTACGAGATGGGCGACCTCTCTGGGAAATACGGGGGTCTTCATGGGCAAACTTGGCTGCACGAGTTTTACAATGACACGAATCTGCAACTGTTTGGGCCCACCTCGATCATGGGACGGTCCGTGGTGATTCATAAGATACAGCAGAATGCGAGGTGGTTCTGCGGCAGTATAGGATGGGGGTACTCACCTGCCGAGGCCCGCCAGGTGTCAGCCATCGCATCCTTCCACAACCCTCAAGGATACGCCGAAGGATACGTCAGGATGCGTCAGTTGGTGTACATGGACGGTTCCTTTGGCGAGACTTTCATCGAGGTGAACCTGAAACACCCTGGCAGTAACAACAGGAACGTGACGAGGCGACACAACTGGTCCGTGTACGTGAACCCTGTGGGAGTGGATGCAGGTGTGAAGTTTTTCCAATCCAGGTGTGTGGCTGCCGGGTACAGGTGGAATCCGTACCTCATCCACCTCGCCTTCCCTAATGACCGTGACTATTACGAGAGGGAGTGTGGACCTGATGTACCCTTGCGCTGTGATGTTGGAGACCTCTCTGGGCGCCTTGGAACCATCGATATTGGAGACAAGCGCTTCGTGTTCGTGGATCGTAACTTGCCACTGTCTGGTCCACACGGCATCATGAACCGGGCCATTATAATCCACAGGGAGAATGCAGGAGTGGAGCGGTTCGCCTGTGCCAATATAGAACCGGATGATGACATTATAAAGTGGGTCATTATCAGAAAGGCGCCCAAATTTTCCGTGCCCACCTTCATGATGGATATCCGGGAGGTTCTGGGGGCGCCGAAGTGGTTCCTGGCTGCTGACCTCCAAACAGTGACCTTTTCCAGTGACCAGCAGTGTGTGACCTTCGTGGTGCACTTCATGGGCCCCAGCGCAGGTCGCCTTGAGTTGGACTTTTCCCGTGTGCTGGCTGGCGGCATCCTGGACCATCCCACCATCAGCATCAGGGGCGTGTACCCTGACCCGGACCGCCCCAAGAAGCTCCCCTACAGGACGTGCGGGGGCCTGGAGGAGGAACTGCTGCTGGAGGAGTATAAGACCAACACCCTGTGGGATCTGATCAAGGGCAGTAGTGATGACGACGATGGTTATGGTGGGTACAGGGGAGCGGCGGCTGGGGCTGGCCTCACCCTGGCCCTGCTCTTCCTGCCTCTGCTTCACTTCCTGCTTTGA

MWRRGGGRRCGGGGGGGGRMRRGKGGGGLLMALCFLCGVQTSYTTSPVLLYSTMSWGGLRGNITFSWGGEGTNVTVTAALEEAGTDLEGETKEYDWAVYDWPVRFDTNKRCGLSDLGTKKRDLSRLLGKLSLPQVDGPQVFETDQLALVGEEAIWGRSIKITGPKTTCANLHGVGGERTYEGRFQAPVGGSVWLRTWAWDVGTGNNSKKGIQTTIFTDVYHTAADDPPSGDHSWSLFITDILDEKENRPSCNFLSRIYDPEGREKCDESGCPMGDLTAAHGPLRVSAARSRFSRKMYTSTKMTLPDLTGPRKLYLAVMGALHPDHLWACTKLRPVLPKKARAVFDALGVTGKITLTQASLFAPTDVTLALEGLGGMAGGFHVHELPALPQRDPGVSHCSATKGHYNPYGVDVATSPEPGLGAHDQYELGDLSGKHGMLLGLEDAQATVTDHNLPLFGPRSVLGRGLVIHKAEGARWVCANLRPTTPQIRAAVTFRYPLVGEMIFEQEADDPHSDTSVLVTYLVYSDGSRNTTGDHRWHVHLHPPGRDFYNWTKRCVSAGPRYNPFKVSTHERQYKGCSVDTPAKCELGDLSGRHGNVRVSGTVKGAPETQRMMTDTNLPLSGPHSILGHSIVIHDDFAPKHRGDRMACMSIHRIFRHKGVVSKWHATRGAGSVEGKIEFVQESEYDLTNTEVELRGLAGMAGGYHVHMVPVEAELEFPCEASTTLGHYNPLNITPSDSPAPTKGTDDLYEMGDLSGKYGGLHGQTWLHEFYNDTNLQLFGPTSIMGRSVVIHKIQQNARWFCGSIGWGYSPAEARQVSAIASFHNPQGYAEGYVRMRQLVYMDGSFGETFIEVNLKHPGSNNRNVTRRHNWSVYVNPVGVDAGVKFFQSRCVAAGYRWNPYLIHLAFPNDRDYYERECGPDVPLRCDVGDLSGRLGTIDIGDKRFVFVDRNLPLSGPHGIMNRAIIIHRENAGVERFACANIEPDDDIIKWVIIRKAPKFSVPTFMMDIREVLGAPKWFLAADLQTVTFSSDQQCVTFVVHFMGPSAGRLELDFSRVLAGGILDHPTISIRGVYPDPDRPKKLPYRTCGGLEEELLLEEYKTNTLWDLIKGSSDDDDGYGGYRGAAAGAGLTLALLFLPLLHFLL

transcript_transcript/34458_Pt_Mix_transcript34458/f75p0/845

ATGCAGCTTCTCCTACTAGTGTGTCTTGCAGTCAGTGCTTCCTGCGGCGCGGTGCACGAAGTTCCTGACGCGGTAGTGAACTTTGTGCCTGGTAATAATAATGTGTACGGGGAACTGCATCTTTTCCGAACTCAATATTCTGGTGTCTTGATCCGAGGGAGAGTGTCCGGTCTGAGTCCAGGTCAACACGGCTTCCATGTGCATGCAGTCGGTGATCTCTCAGGGAAGTGCACGGCCGCAGGTGGTCACTTCAATCCCTACATGACTACCCACGGCTCCCCCTATGACGCTAACCGCCACGCTGGAGACCTTGGCAACATTCAGGCTGATCTTCATGGCAACGCTGACGTGTATATTCATGACTTGGTGATCTCCCTCGACCCTGCCTCCCCTGCGTACATCGGCAACCTGGCCATAGTGGTTCACCAGGGCGAGGATGATCTAGGACGTGGAGGCAATGCGGATAGCTTGAAAACTGGCAATGCTGGAGGACGTGCTGGGTGTGGACTCATCATACCTCTGTGA

MQLLLLVCLAVSASCGAVHEVPDAVVNFVPGNNNVYGELHLFRTQYSGVLIRGRVSGLSPGQHGFHVHAVGDLSGKCTAAGGHFNPYMTTHGSPYDANRHAGDLGNIQADLHGNADVYIHDLVISLDPASPAYIGNLAIVVHQGEDDLGRGGNADSLKTGNAGGRAGCGLIIPL

transcript_transcript/24601_Pt_Mix_transcript24601/f2p0/1603

ATGGCAGAGAAGGACCTGTACATTGCTGCCCTTGAGAAGAAGCTGGCTGAGTTGTCTGGTATTGAAGTTGATCAGATCAAGAAAAACCAGCTAGCCAATGCTTCAAGTGAGGCACGTTCCATCCGTGAGATGGCCGAGTATGTGGAGGGCATCCAGGTGAAGCAAGCTGGACAGGTTATTACTGGTCAGGTGAATCCTCAGGTGGCTGCCATGTTTGCCCACATCAAGGCTGAACTTGGTGAGGAGCGTGGAGCACATTCCCTGCCACCTTTGAAATATGATTACAGTGCCCTGGAACCACATATTTGTACCACCATCATGCAGATCCATCACACCAAGCACCATCAAGGATACATCAACAACCTGAAGGCAGCTGTAGAAAAGCTTGCAGAAGCAGAGAAGGCTAATGATATAGCTGCCATAAATGCACTTTTTCCGGCTATGAAGTTCAATGGAGGTGGTCATTTAAACCATACCATCTTCTGGACCAACATGGCACCTGAAGCTGGAGGAGAACCCACAGGAGACATTGCTGAGGCCATCAACAAGGATTTTGGCTCATTCCAGTCCTTCAAGGAAAAGTTCTCAGGTGTCAGTGTTGCTGTGAAAGGATCCGGTTGGGGTTGGCTTGGCTACTGCCCAAAGGATGACAAGCTTGCTATTGCCTCGTGCCAGAATCAGGACCCCCTGCAGCTTACCCATGGTCTGGTGCCACTGCTGGGTTTGGATGTGTGGGAGCATGCCTATTATCTCCAATATAAGAACCTACGTGCTGATTATGTCAAAGCCTTCTTCAATGTGATCAATTGGGCCAACGTGAACGAGCGCTATGAAGTAGCTCGTAAGGCAGCTGGACACTGA

MAEKDLYIAALEKKLAELSGIEVDQIKKNQLANASSEARSIREMAEYVEGIQVKQAGQVITGQVNPQVAAMFAHIKAELGEERGAHSLPPLKYDYSALEPHICTTIMQIHHTKHHQGYINNLKAAVEKLAEAEKANDIAAINALFPAMKFNGGGHLNHTIFWTNMAPEAGGEPTGDIAEAINKDFGSFQSFKEKFSGVSVAVKGSGWGWLGYCPKDDKLAIASCQNQDPLQLTHGLVPLLGLDVWEHAYYLQYKNLRADYVKAFFNVINWANVNERYEVARKAAGH

transcript_transcript/29482_Pt_Mix_transcript29482/f11p0/1271

ATGGCAGAGAAGGACCTGTACATTGCTGCCCTTGAGAAGAAGCTGGCTGAGTTGTCTGGTATTGAAGTTGATCAGATCAAGAAAAACCAGCTAGCCAATGCTTCAAGTGAGGCACGTTCCATCCGTGAGATGGCCGAGTATGTGGAGGGCATCCAGGTGAAGCAAGCTGGACAGGTTATTACTGGTCAGGTGAATCCTCAGGTGGCTGCCATGTTTGCCCACATCAAGGCTGAACTTGGTGAGGAGCGTGGAGCACATTCCCTGCCACCTTTGAAATATGATTACAGTGCCCTGGAACCACATATTTGTACCACCATCATGCAGATCCATCACACCAAGCACCATCAAGGATACATCAACAACCTGAAGGCAGCTGTAGAAAAGCTTGCAGAAGCAGAGAAGGCTAATGATATAGCTGCCATAAATGCACTTTTTCCGGCTATGAAGTTCAATGGAGGTGGTCATTTAAACCATACCATCTTCTGGACCAACATGGCACCTGAAGCTGGAGGAGAACCCACAGGAGACATTGCTGAGGCCATCAACAAGGATTTTGGCTCATTCCAGTCCTTCAAGGAAAAGTTCTCAGGTGTCAGTGTTGCTGTGAAAGGATCCGGTTGGGGTTGGCTTGGCTACTGCCCAAAGGATGACAAGCTTGCTATTGCCTCGTGCCAGAATCAGGACCCCCTGCAGCTTACCCATGTCAGAGGATCCTGGTGGGGATGA

MAEKDLYIAALEKKLAELSGIEVDQIKKNQLANASSEARSIREMAEYVEGIQVKQAGQVITGQVNPQVAAMFAHIKAELGEERGAHSLPPLKYDYSALEPHICTTIMQIHHTKHHQGYINNLKAAVEKLAEAEKANDIAAINALFPAMKFNGGGHLNHTIFWTNMAPEAGGEPTGDIAEAINKDFGSFQSFKEKFSGVSVAVKGSGWGWLGYCPKDDKLAIASCQNQDPLQLTHVRGSWWG

transcript_transcript/4284_Pt_Mix_transcript4284/f4p0/3568

ATGCCGAGGGACAAGGCAGCCGAGCAACTCAATGAATTTAAGAAGAGCCAGACAAACCCAGATGTGCTCACCACTGGCCATGGCTGTCCATTGTCGGATAAGCTCAACTCCCTCACAGTGGGCCCACGAGGACCCATTCTTCTTCAGGACATTCAGCTGATAGATGAAATGGCCCACTTTGACCGAGAACGTATCCCTGAGAGGGTAGTCCATGCCAAAGGAGCAGGTGCATTTGGATACTTTGAAGTCACCCATGACATCTCACAGTATACTAAAGCCAAGATCTTCAGTGAGATTGGCAAACGCACACCTTTGGCTATTCGATTCTCCACTGTAGGTGGGGAGAGTGGATCTGCAGATACTGCCAGGGATCCTCGAGGTTTTGCTGTGAAATTTTATACTGAAGAAGGTAATTGGGATCTGGTAGGCAACAACACTCCCATCTTCTTTATCAGGGATCCTGTTCTCTTCCCTTCCTTCATCCACACCCAAAAGAGGAACCCAGCTACTCATCTTAAGGATGCAGACATGTTCTGGGATTTCATCACTCTGCGTCCAGAAACAACACATCAAGTGTCATTCCTCTTCTCTGATCGTGGTATCCCAGATGGCTACCGGCACATGAATGGTTATGGGTCTCACACCTTCAAGTTGGTAAACAAAGAAGGGAACCCTGTATACTGCAAGTTCCACTACAAGACTGACCAAGGCATCAAATGCCTTAGTGCAGAAAGAGCAGATTTCTTGGCAGGCTCTGACCCTGACTATGCTATCCGTGACCTGTACAATGCAATCAGTGAAGGCAACTATCCCTCCTACACCATGTATATCCAGGTAATGACCTATGAGCAGGCAGAGAAGTGGGAATTCAACCCATTTGACTTGACCAAGGTCTGGCCCCATGCTGATTTCCCGCTAATTCCTGTTGGTCGCATCACACTTGATCGCAATGCACAAAACTACTTTGCTGAGGTGGAGCAGCTGGCCTTCTCCCCTGCCAACCTTGTGCCAGGCATCGAGCCATCCCCAGACAAGATGCTTCAGGGTCGCCTCTTTTCATACAATGATACCCATCGGCATCGCCTGGGAGCCAACTACCATCAGATCCCAGTGAACTGCCCTTACCGTGCCCGCCCTAAGAACTATCAAAGGGATGGTCCCATGACAGTGAATGATAACCAGATGTGTGCACCCAACTACTTCCCCAACAGCTTCTCTGGACCTATGGACTGCAAACAATTTGAGGGACCCAAGGAGAAGCTTGCAGGGGATGTAATGCGCTACAATAGTGCTGATGAAGATAATTTCACACAAGTGTGCATCTTCTATAGAAATGTGCTGAATGAAGAGGAACGGCAACGATTGGTGAACAATATTGCTGGCCATCTTGTGAATGCTCAGGAGTTTTTGCAAGAGAGGGCCATAAAGAACTTCTCTCAAGCCTGCCCTGAGTATGGTGCTGGCATCAGGTCTGCTTTGAACCGCATCAAAGCAGCACAGTCATCAAATTCCTCTGCTATCCATGCAGTGGCAGCCTCCAATGCCAAGTTGTGA

MPRDKAAEQLNEFKKSQTNPDVLTTGHGCPLSDKLNSLTVGPRGPILLQDIQLIDEMAHFDRERIPERVVHAKGAGAFGYFEVTHDISQYTKAKIFSEIGKRTPLAIRFSTVGGESGSADTARDPRGFAVKFYTEEGNWDLVGNNTPIFFIRDPVLFPSFIHTQKRNPATHLKDADMFWDFITLRPETTHQVSFLFSDRGIPDGYRHMNGYGSHTFKLVNKEGNPVYCKFHYKTDQGIKCLSAERADFLAGSDPDYAIRDLYNAISEGNYPSYTMYIQVMTYEQAEKWEFNPFDLTKVWPHADFPLIPVGRITLDRNAQNYFAEVEQLAFSPANLVPGIEPSPDKMLQGRLFSYNDTHRHRLGANYHQIPVNCPYRARPKNYQRDGPMTVNDNQMCAPNYFPNSFSGPMDCKQFEGPKEKLAGDVMRYNSADEDNFTQVCIFYRNVLNEEERQRLVNNIAGHLVNAQEFLQERAIKNFSQACPEYGAGIRSALNRIKAAQSSNSSAIHAVAASNAKL

transcript_transcript/7018_Pt_Mix_transcript7018/f2p0/3092

ATGGCCCACTTTGACCGAGAACGTATCCCTGAGAGGGTAGTCCATGCCAAAGGAGCAGGTGCATTTGGATACTTTGAAGTCACCCATGACATCTCACAGTATACTAAAGCCAAGATCTTCAGTGAGATTGGCAAACGCACACCTTTGGCTATTCGATTCTCCACTGTAGGTGGGGAGAGTGGATCTGCAGATACTGCCAGGGATCCTCGAGGTTTTGCTGTGAAATTTTATACTGAAGAAGGTAATTGGGATCTGGTAGGCAACAACACTCCCATCTTCTTTATCAGGGATCCTGTTCTCTTCCCTTCCTTCATCCACACCCAAAAGAGGAACCCAGCTACTCATCTTAAGGATGCAGACATGTTCTGGGATTTCATCACTCTGCGTCCAGAAACAACACATCAAGTGTCATTCCTCTTCTCTGATCGTGGTATCCCAGATGGCTACCGGCACATGAATGGTTATGGGTCTCACACCTTCAAGTTGGTAAACAAAGAAGGGAACCCTGTATACTGCAAGTTCCACTACAAGACTGACCAAGGCATCAAATGCCTTAGTGCAGAAAGAGCAGATTTCTTGGCAGGCTCTGACCCTGACTATGCTATCCGTGACCTGTACAATGCAATCAGTGAAGGCAACTATCCCTCCTACACCATGTATATCCAGGTAATGACCTATGAGCAGGCAGAGAAGTGGGAATTCAACCCATTTGACTTGACCAAGGTCTGGCCCCATGCTGATTTCCCGCTAATTCCTGTTGGTCGCATCACACTTGATCGCAATGCACAAAACTACTTTGCTGAGGTGGAGCAGCTGGCCTTCTCCCCTGCCAACCTTGTGCCAGGCATCGAGCCATCCCCAGACAAGATGCTTCAGGGTCGCCTCTTTTCATACAATGATACCCATCGGCATCGCCTGGGAGCCAACTACCATCAGATCCCAGTGAACTGCCCTTACCGTGCCCGCCCTAAGAACTATCAAAGGGATGGTCCCATGACAGTGAATGATAACCAGATGTGTGCACCCAACTACTTCCCCAACAGCTTCTCTGGACCTATGGACTGCAAACAATTTGAGGGACCCAAGGAGAAGCTTGCAGGGGATGTAATGCGCTACAATAGTGCTGATGAAGATAATTTCACACAAGTGTGCATCTTCTATAGAAATGTGCTGAATGAAGAGGAACGGCAACGATTGGTGAACAATATTGCTGGCCATCTTGTGAATGCTCAGGAGTTTTTGCAAGAGAGGGCCATAAAGAACTTCTCTCAAGCCTGCCCTGAGTATGGTGCTGGCATCAGGTCTGCTTTGAACCGCATCAAAGCAGCACAGTCATCAAATTCCTCTGCTATCCATGCAGTGGCAGCCTCCAATGCCAAGTTGTGA

MAHFDRERIPERVVHAKGAGAFGYFEVTHDISQYTKAKIFSEIGKRTPLAIRFSTVGGESGSADTARDPRGFAVKFYTEEGNWDLVGNNTPIFFIRDPVLFPSFIHTQKRNPATHLKDADMFWDFITLRPETTHQVSFLFSDRGIPDGYRHMNGYGSHTFKLVNKEGNPVYCKFHYKTDQGIKCLSAERADFLAGSDPDYAIRDLYNAISEGNYPSYTMYIQVMTYEQAEKWEFNPFDLTKVWPHADFPLIPVGRITLDRNAQNYFAEVEQLAFSPANLVPGIEPSPDKMLQGRLFSYNDTHRHRLGANYHQIPVNCPYRARPKNYQRDGPMTVNDNQMCAPNYFPNSFSGPMDCKQFEGPKEKLAGDVMRYNSADEDNFTQVCIFYRNVLNEEERQRLVNNIAGHLVNAQEFLQERAIKNFSQACPEYGAGIRSALNRIKAAQSSNSSAIHAVAASNAKL

transcript_transcript/8029_Pt_Mix_transcript8029/f2p0/2965

ATGGGTGTCCCTAGATCAACAGTATCAATAATTTGGAAGAACAGGGACAAGTACCGTGAGACTGGTGTATCATGTGCATTTGGATACTTTGAAGTCACCCATGACATCTCACAGTATACTAAAGCCAAGATCTTCAGTGAGATTGGCAAACGCACACCTTTGGCTATTCGATTCTCCACTGTAGGTGGGGAGAGTGGATCTGCAGATACTGCCAGGGATCCTCGAGGTTTTGCTGTGAAATTTTATACTGAAGAAGGTAATTGGGATCTGGTAGGCAACAACACTCCCATCTTCTTTATCAGGGATCCTGTTCTCTTCCCTTCCTTCATCCACACCCAAAAGAGGAACCCAGCTACTCATCTTAAGGATGCAGACATGTTCTGGGATTTCATCACTCTGCGTCCAGAAACAACACATCAAGTGTCATTCCTCTTCTCTGATCGTGGTATCCCAGATGGCTACCGGCACATGAATGGTTATGGGTCTCACACCTTCAAGTTGGTAAACAAAGAAGGGAACCCTGTATACTGCAAGTTCCACTACAAGACTGACCAAGGCATCAAATGCCTTAGTGCAGAAAGAGCAGATTTCTTGGCAGGCTCTGACCCTGACTATGCTATCCGTGACCTGTACAATGCAATCAGTGAAGGCAACTATCCCTCCTACACCATGTATATCCAGGTAATGACCTATGAGCAGGCAGAGAAGTGGGAATTCAACCCATTTGACTTGACCAAGGTCTGGCCCCATGCTGATTTCCCGCTAATTCCTGTTGGTCGCATCACACTTGATCGCAATGCACAAAACTACTTTGCTGAGGTGGAGCAGCTGGCCTTCTCCCCTGCCAACCTTGTGCCAGGCATCGAGCCATCCCCAGACAAGATGCTTCAGGGTCGCCTCTTTTCATACAATGATACCCATCGGCATCGCCTGGGAGCCAACTACCATCAGATCCCAGTGAACTGCCCTTACCGTGCCCGCCCTAAGAACTATCAAAGGGATGGTCCCATGACAGTGAATGATAACCAGATGTGTGCACCCAACTACTTCCCCAACAGCTTCTCTGGACCTATGGACTGCAAACAATTTGAGGGACCCAAGGAGAAGCTTGCAGGGGATGTAATGCGCTACAATAGTGCTGATGAAGATAATTTCACACAAGTGTGCATCTTCTATAGAAATGTGCTGAATGAAGAGGAACGGCAACGATTGGTGAACAATATTGCTGGCCATCTTGTGAATGCTCAGGAGTTTTTGCAAGAGAGGGCCATAAAGAACTTCTCTCAAGCCTGCCCTGAGTATGGTGCTGGCATCAGGTCTGCTTTGAACCGCATCAAAGCAGCACAGTCATCAAATTCCTCTGCTATCCATGCAGTGGCAGCCTCCAATGCCAAGTTGTGA

MGVPRSTVSIIWKNRDKYRETGVSCAFGYFEVTHDISQYTKAKIFSEIGKRTPLAIRFSTVGGESGSADTARDPRGFAVKFYTEEGNWDLVGNNTPIFFIRDPVLFPSFIHTQKRNPATHLKDADMFWDFITLRPETTHQVSFLFSDRGIPDGYRHMNGYGSHTFKLVNKEGNPVYCKFHYKTDQGIKCLSAERADFLAGSDPDYAIRDLYNAISEGNYPSYTMYIQVMTYEQAEKWEFNPFDLTKVWPHADFPLIPVGRITLDRNAQNYFAEVEQLAFSPANLVPGIEPSPDKMLQGRLFSYNDTHRHRLGANYHQIPVNCPYRARPKNYQRDGPMTVNDNQMCAPNYFPNSFSGPMDCKQFEGPKEKLAGDVMRYNSADEDNFTQVCIFYRNVLNEEERQRLVNNIAGHLVNAQEFLQERAIKNFSQACPEYGAGIRSALNRIKAAQSSNSSAIHAVAASNAKL

transcript_transcript/19651_Pt_Mix_transcript19651/f2p0/1955

ATGGGTTACCAGCGGCAGGACGTGGCTCTGTTCTCTTGTCTCGTCCTCCTGGCAGCCCTATGCACTGCAGGCTGTGCTGGGGAAGACTTCTACAGCTTCTCCGTCAAGGACCACCAGGGGCAGGACGTGGCACTGGAGTCCTACAGAGGGAAGGTTACTCTGGTGGTGAATGTTGCTAGTTTATGTGGCTACACAGAGACCACATACCGAGCCCTCAAGAAGCTGCATGACATTCTTGGTTATGGTGGCCACTTCTCAGTGCTGGCTTTCCCATGCAATCAGTTTGGTGATCAGGAGCCACTTGACATCGAAGACATACTGGAACATGTCACCTCAGAGTATGAGGTGGAGTTCCCAGTATTCAACAAGATCGATGTGGTGGGACCAAACGCTGACCCAGCCTTCAGACAACTCATTGAGGATTCATCCATTGTGCCGGACTGGAACTTCTACAAATACTTGGTGAATGGGTCTGGCCGGGTGGTAGGGGCTTGGGGCACCCGCGTCACCATAGAGGAAATCTTTGATGAGGTCCAGGCTGAGGTGAAGAAGGCGAAGGAAGCAGCATCACCTGACAAGAAAGAAGCATCGCCAGTTGAAGGTGGTGGCAAAGATGAACTCTAG

MGYQRQDVALFSCLVLLAALCTAGCAGEDFYSFSVKDHQGQDVALESYRGKVTLVVNVASLCGYTETTYRALKKLHDILGYGGHFSVLAFPCNQFGDQEPLDIEDILEHVTSEYEVEFPVFNKIDVVGPNADPAFRQLIEDSSIVPDWNFYKYLVNGSGRVVGAWGTRVTIEEIFDEVQAEVKKAKEAASPDKKEASPVEGGGKDEL

transcript_transcript/26824_Pt_Mix_transcript26824/f2p0/1448

ATGGCCAGCCGTGTTAAGTCTTTCTACGAGCTCAGTGCTAAGGCTCTCTCCGGTGTGGAGGTACCGTTCAGCAAGTACCGTGGGAAGGTCGTACTGATCCAGAACACGGCGTCTCTCTGAGGTACGACCACGCGGGACTTCCTCGAGATGAACCAGCTGATCGAAAAGTTTGGTGACCGTCTCGCAGTCCTCGCGTTCCCCTGCAATCAGTTTGGTCACCAGGAAAACACCACTCACGAGGAGCTTCTGAGTTCCCTGCGCCACGTTCGTCCTGGTAACAACTTTGAGCCCAAGATGGAGATGTTTGGCAAGGTGGAGGTCAACGGAACGGGCACCCACCAGGTCTTCCAGCTACTGAAGGAGGCGCTGCCGTTGCCCGTGGACGACCCAGTGAGTCTAATGGCAGACCCCAAGTGTATCATCTGGAACCCCGTGACACGCTCTGATGTTGCCTGGAATTTTGAAAAGTTCCTTGTTGACTCCAGCGGCAAGCCAATTAAAAGATTCAGCAAGAAATTCCCAACCAAGGACCTGGACACCCACATAGAAAAACTGCTTGAGTAGGACCCCCGTAGGACTCCCTGAAGGACTCCACCCCGCCACAGGCCCCTCCTAGCATCCCTCAACCCCCCAGCCCCTCGCAGGACCCCACAGGACCCACACAGGACCCCCCAAAGGACTCTCTAATGAAGGACCCTCTTTAAACACTTCTACGTGAGAGAGAGGCTGGAAAAAATGAGTCTGGGGTGTTTTGTGTG

MASRVKSFYELSAKALSGVEVPFSKYRGKVVLIQNTASLUGTTTRDFLEMNQLIEKFGDRLAVLAFPCNQFGHQENTTHEELLSSLRHVRPGNNFEPKMEMFGKVEVNGTGTHQVFQLLKEALPLPVDDPVSLMADPKCIIWNPVTRSDVAWNFEKFLVDSSGKPIKRFSKKFPTKDLDTHIEKLLE

transcript_transcript/32938_Pt_Mix_transcript32938/f157p0/917

ATGCTGTGGGCGTGGCTGCTGCCCGTGGCCGCGGTGGGTGTGGGGGCCCAGGACAGGGTGCCGTCAAGGGCCTGCTACCACCATGAGAGTGACGGAGGCTCCATCTATGACTTCCTTGAGTCTGATCTGTTTGAGACAAGAAATATATCTCTGTCGGAGCACCAGGGGAAGGTGGTGCTGATCGTCAACGTGGCGACTTACTGAGGGTACACCCATCAGTACACCGACATGAATGTGCTGCAAGAGTCCTTCACTAATTTTGAAGTCCTGGCTTTCCCCTGCAACCAGTTTGGGAAGCAAGAGCCAGCAGGGACCCCAGAGGAGCTCCTTAACGGCGTGACCTACGTGCGTCCCGGGAATGGCTTCATCCCTAACTTCACCATGTTCAAGAAGATCGAGGTGAACGGCGAGAACGAACACCCGCTGTACACGTACCTCAAGGCGTACTGTCCCCCGACACGCCAGAGCTTCTCGGACGCCAACAAACTATACTACAAGCCACAGAAGAACAGCGACGTCCGCTGGAACTGGGAGAAGTTCCTCATCACTAAGAGTGGCAAGCCCTTCATGCGGTACGACCCGGGCACCAAACCTGATGAGATCCGCAACGACATCATGTTCCTGCTGCAGCAGAAGTCTTAGCTGGCCCCGCAAGGCCTGCTATTGCTGCTGCTCCCGCCAAGAGGGGCGGCGTCCTGACGGTGGCCTGGCCCGTTGTGATGAAGGCGTCGCCGGAAACCCAGCGGTGGGCGGCGCTGGAGGGCGGGACGGCCTGGGTTTGGT

MLWAWLLPVAAVGVGAQDRVPSRACYHHESDGGSIYDFLESDLFETRNISLSEHQGKVVLIVNVATYUGYTHQYTDMNVLQESFTNFEVLAFPCNQFGKQEPAGTPEELLNGVTYVRPGNGFIPNFTMFKKIEVNGENEHPLYTYLKAYCPPTRQSFSDANKLYYKPQKNSDVRWNWEKFLITKSGKPFMRYDPGTKPDEIRNDIMFLLQQKS

transcript_transcript/19628_Pt_Mix_transcript19628/f6p0/1896

ATGGCACCTGTACTGGCTTACTGGGATATTCGTGGGCTTGCCCAGCCCATCCGTCTCTTGCTGGAATACACAGGGACGGAGTTTGAGGACAAGTATTACAAATGTGGCCCAGCTCCTGATTACGACAAGTCTTGTTGGTTTGACATCAAGGAAACACTTGGATTTGATTTTCCTAATCTTCCCTATTACATTGATGGGGACATCAAGGTAACACAGAGCAATGCCATTATGCGCTACATTGCTCGCAAACACGACCTCTGTGGTAAGACTGAGGAGGAAAAAGTCCGTGTTGACATCCTGGAGAACCAAGCCATGGACTTCCGCAATGGTTTTGTTCGCTTGTGCTATGCGACATATGACACAGGGAAGGATGCCTATCTGCAGGCCCTGTCAAAGACCATCGAAAGGTTCTCAAAGTTTTTAGGGACACGCACTTGGTTTGCTGGTGATGAGATCACCTTTGTGGACTTTATCATGTATGAATTACTAGACCAACACCTGCAGTTAGACAAGGACTGTCTCAAGGATGCTCAGAACCTGCAAGATTACCAGAAGCGCTTTGAGGAGCTGGAGCCCATCAAGAAGTACATGGCCTCCACCAGGTTCATGAAGGCTCCCCTGAACAACAAGATGGCAAAGTTCGGTAACAAGTAG

MAPVLAYWDIRGLAQPIRLLLEYTGTEFEDKYYKCGPAPDYDKSCWFDIKETLGFDFPNLPYYIDGDIKVTQSNAIMRYIARKHDLCGKTEEEKVRVDILENQAMDFRNGFVRLCYATYDTGKDAYLQALSKTIERFSKFLGTRTWFAGDEITFVDFIMYELLDQHLQLDKDCLKDAQNLQDYQKRFEELEPIKKYMASTRFMKAPLNNKMAKFGNK

transcript_transcript/2154_Pt_Mix_transcript2154/f2p0/4027

ATGCCATACGGACATGTGCCTGTGCTGTTCGTGGACGGGAAGCCGCTGTGTCAGAGCGTCAGTATCTGCCGGTACCTGGGACGCATGCACGGCCTCTTCGTGGACGACCCTTGGGAGGCCGCCTTAGGGGACGAGGTGGCTGACGCCGTGAACGACCTTATTCAACCCGCCGGTCAGATCGCGTATGCCAGACTTGCGAACGAGACCGAGAAATACAAGATGCTGGCCACGGAGTTCCACACCACCACGCTGCCGCCAACCATAAGGGAACTGGACCGCAGACTGGACGGACGGGATTGGTTCTGCGGCTCCAAGATGACGTGGGTGGACCTGTTCGCCGCCTGTTACCTGAAGGAGATCACTTTGCAACACGAAGGCTCCTTGGACGCCGTCCCGCGCCTGAAGAAACACGTGGAGAAGATTGCCAAGCTGCCACAGATCGAGAAATGGCTAAAAGAGCGGCCAGATTCCATGCTGTAG

MPYGHVPVLFVDGKPLCQSVSICRYLGRMHGLFVDDPWEAALGDEVADAVNDLIQPAGQIAYARLANETEKYKMLATEFHTTTLPPTIRELDRRLDGRDWFCGSKMTWVDLFAACYLKEITLQHEGSLDAVPRLKKHVEKIAKLPQIEKWLKERPDSML

transcript_transcript/21822_Pt_Mix_transcript21822/f3p0/1790

ATGATGTACCTAATGAACAAATACGCCACGGAGGAGAACCAGTATCTGTACCCGAAGGAGCCTGAGGAGAGGGCGAAGGTGGACCGCATGTTATTCTTCGACATGGGAACGCTCTACCACTCGATCAAGGAGTACTTTGCCCCCAAGATATACGACGGCCTGCCCCCGGACCCAGAGAAGGAGAACCTGCTGAAGACAAGCCTGGATCACCTGGACCACTTCCTGGAGATCGGTGGCGTGCCTTACCTGTGCGGGGAGAGGATCACTCTCGCTGACGTGGCTGTCCTCGCCTCCGTCACTGAGCTGGACGCTATGGAATACAACTACAGGTGTTACGGGGAGTTCAACAGATGGGTGGAGCGAGTGAAGGCCGCCATCAAGCCTTACAAGGAGTGCTGCGAGGAGGGCGTGCGGATGACCAAAGACCGCACCAAGTTCCTGGAGACTGAGGCCAAGAAGCAGATGGAGAAGGAACGCAAGGCTTCCCTCCCCAAGAAGTAA

MMYLMNKYATEENQYLYPKEPEERAKVDRMLFFDMGTLYHSIKEYFAPKIYDGLPPDPEKENLLKTSLDHLDHFLEIGGVPYLCGERITLADVAVLASVTELDAMEYNYRCYGEFNRWVERVKAAIKPYKECCEEGVRMTKDRTKFLETEAKKQMEKERKASLPKK

transcript_transcript/22079_Pt_Mix_transcript22079/f4p0/1758

ATGGGCAGCGTTGGTCACTCTCCGGCTCTTCCCACGCCCTCCCCTGCCGCCCCAACGCTCACCCCTGACAGCATGGGGGTGGTGGGTGACGGGAGTGAAGGAGGGGATGCTGTGTGCGTGTATGTAAAGGCAGGAGTGGATGGGGAGAGGATGGGAGCATGTCCATTCTGTCAACGAATTTTCATGGTTTTGTTGATTAAGGCGAACCATGGATTGCTACGGTTTAAGGTGATCACAACCAACCCAGCCAAGCCACCGCCAGAATTCAAGATGTTAGGACTGAAGCACCTGCCGGCTCTCATTCACGGCGAGGAAGGCTTTGATGCCCTTGACGACATCATTCAGTACCTTGACACTAGGTTTCCAGGTGGAGGTCTGGAATACAACCACCCACACGCAGACCTGGCCACCAAAGACTTCTTCTCCAGGTTCTGCTTCTACATCAAGGCGGTGAACTTAGACTCGACCAAGCTGGACCAAGCACTATGTAGCCTCGACGCTTTCCTCCAGCGCCCTGTCTTCCCCTCACCTGACGCTGGACTAACTAACGGCCAGCATGAGGCACCAGAATCACCAGGTGAGGGCCCTCAGCCTCCCCAGTACCTGTGTGGTGACTCCCTAACACACCTGGACTGCGAGGTACTGCCCAAACTCCAGCACCTTAGAGTGGCGGCAAAAGCTCTCAAGAACTATGATATTCCAACACATCTACGTGGATTCTGGAGGTACCTCCACGCAGCATACACACACCCAGTCTTCATTCGCACGTGTCCCTGCGACCAGGAGGTGACACTGCACTGGCACGATCGCCCTGAGACGCCCAAACTGTCCCACAAACGCCACGCAGCCATCGCAAAGGAGAAGCCTAAATATTCCTTTGATGTGCCTGTTCATGCCTCTGTGGTGACTGTTGTTGATGAGTGA

MGSVGHSPALPTPSPAAPTLTPDSMGVVGDGSEGGDAVCVYVKAGVDGERMGACPFCQRIFMVLLIKANHGLLRFKVITTNPAKPPPEFKMLGLKHLPALIHGEEGFDALDDIIQYLDTRFPGGGLEYNHPHADLATKDFFSRFCFYIKAVNLDSTKLDQALCSLDAFLQRPVFPSPDAGLTNGQHEAPESPGEGPQPPQYLCGDSLTHLDCEVLPKLQHLRVAAKALKNYDIPTHLRGFWRYLHAAYTHPVFIRTCPCDQEVTLHWHDRPETPKLSHKRHAAIAKEKPKYSFDVPVHASVVTVVDE

transcript_transcript/2234_Pt_Mix_transcript2234/f6p0/4172

ATGCCTGAGTACAAGCTGGTCTATCTTACCCTCCGTGGCCGCGCGGAGCCCATTCGCTGGATACTGGCCGTCACGGATCAGCCCTACGATGACGTGCGCTACATCAGAGAGACGGAGTGGCCCTTTAAAAAGCCAGAGATGCCATACGGACACGTGCCTGTGCTGTTCGTGGACGGGAAGCCGCTGTGTCAGAGCGTCAGTATCTGCCGGTACCTGGGACGCATGCACGGCCTCTTCGTGGACGACCCTTGGGAGGCCGCCTTAGGGGACGAGGTGGCTGACGCCGTGAACGACCTTATTCAACCCGCCGGTCAGATCGCGTATGCCAGACTTGCGAACGAGACCGAGAAATACAAGATGCTGGCCACGGAGTTCCACACCACCACGCTGCCGCCAACCATAAGGGAACTGGACCGCAGACTGGACGGACGGGATTGGTTCTGCGGCTCCAAGATGACGTGGGTGGACCTGTTCGCCGCCTGTTACCTGAAGGAGATCACCTTGCAACACGAAGGCTCCTTGGACGCCGTCCCGCGCCTGAAGAAACACGTGGAGAAGATTGCCAAGCTGCCACAGATCGAGAAATGGCTAAAAGAGCGGCCAGATTCCATGCTGTAG

MPEYKLVYLTLRGRAEPIRWILAVTDQPYDDVRYIRETEWPFKKPEMPYGHVPVLFVDGKPLCQSVSICRYLGRMHGLFVDDPWEAALGDEVADAVNDLIQPAGQIAYARLANETEKYKMLATEFHTTTLPPTIRELDRRLDGRDWFCGSKMTWVDLFAACYLKEITLQHEGSLDAVPRLKKHVEKIAKLPQIEKWLKERPDSML

transcript_transcript/22683_Pt_Mix_transcript22683/f2p0/1714

ATGGCGTGGCTGAGCACTGAATTTTACTTCTACAAGGGGAGCGCTCCGTGTCGCGCCGTGTGGATGACGCTCAAAATGCTCAATGTGGAGTACGAGGCTAAGGAAGTGGATCTCCTGAAGGCTGAGAACAAGCGGCCATGGTTTTTGAGGCTCAACCCGCAGCACACTGTTCCCACTCTGACCGAGGGAGATTTCGCTCTATGGGAGAGCCGCGCCATTATGATGTACCTAATGAACAAATACGCCACGGAGGAGAACCAGTATCTGTACCCGAAGGAGCCTGAGGAGAGGGCGAAGGTGGACCGCATGTTATTCTTCGACATGGGAACGCTCTACCACTCGATCAAGGAGTACTTTGCCCCCAAGATATACGACGGCCTTCCCCCGGACCCAGAGAAGGAGAACCTGCTGAAGACAAGCCTGGATCACCTGGACCACTTCCTGGAGATCGGTGGCGTGCCTTACCTGTGCGGGGAGAGGATCACTCTCGCTGACGTGGCTGTCCTCGCCTCCGTCACTGAGCTGGACGCTATGGAATACAACTACAGGTGTTACGGGGAGTTCAACAGATGGGTGGAGCGAGTGAAGGCCGCCATCAAGCCTTACAAGGAGTGCTGCGAGGAGGGCGTGCGGATGACCAAAGACCGCACCAAGTTCCTGGAGACTGAGGCCAAGAAGCAGATGGAGAAGGGATGTTATTTCTATTGA

MAWLSTEFYFYKGSAPCRAVWMTLKMLNVEYEAKEVDLLKAENKRPWFLRLNPQHTVPTLTEGDFALWESRAIMMYLMNKYATEENQYLYPKEPEERAKVDRMLFFDMGTLYHSIKEYFAPKIYDGLPPDPEKENLLKTSLDHLDHFLEIGGVPYLCGERITLADVAVLASVTELDAMEYNYRCYGEFNRWVERVKAAIKPYKECCEEGVRMTKDRTKFLETEAKKQMEKGCYFY

transcript_transcript/24745_Pt_Mix_transcript24745/f2p0/1590

ATGACCTACCGCCTGCTGTACCTGAACATCCGGGCCCGCGGGGAGCCCATCAGGTGGGTGCTGCGGGCGCTGGAGGTGGAATTCGAGGAAGAGCGGATCGATATGTTCTCCGAGTGGGGGGCCAAGAAGCAAGACTTCAAGTGGGGCCAGACGCCGGTGCTGGAGGTGGACGGGGAACAGCTGGGACAGACGACGGTGATTTGCCGCTATCTTGGAGAAAAACACAACATGGCGGTGGAGGATCCCTGGATCGCCAGTCGCCTGCAGGAGGCCGCCGAGTACCTCCATGATGTGTCCACCCTCGCTGCCTTCGCCTTCTACTTCAGGATCAAGAATGATGAGGAGCAGAAGGAGTTCTTCATGGGCAGAGTCCGGGAACGTGCGGCGGTGGTGGTTAAGAATCTGGAGAATTTAGTCACCGACGAGGCGGGCTGGCTCCTGTCGCCAAAGATGACGTGGGTGGACGTGTTCACAGCAGCCTATCTAGACCAGTACGTGGACATGATAGATGGCCTGCTGGAGGAAGCCCCCAAACTGCAGGAGATATTAGGGCGGGTGCGATCCCTGCCGGCTATCCAGGAGTGGATCGAGGCGCGCCCTCCCCTGCACGAATTCGAGACCAACGAGGGACTATGA

MTYRLLYLNIRARGEPIRWVLRALEVEFEEERIDMFSEWGAKKQDFKWGQTPVLEVDGEQLGQTTVICRYLGEKHNMAVEDPWIASRLQEAAEYLHDVSTLAAFAFYFRIKNDEEQKEFFMGRVRERAAVVVKNLENLVTDEAGWLLSPKMTWVDVFTAAYLDQYVDMIDGLLEEAPKLQEILGRVRSLPAIQEWIEARPPLHEFETNEGL

transcript_transcript/27235_Pt_Mix_transcript27235/f5p0/1356

ATGGCCCCTACACTGTACTACTTCTTGATGTCGCCTTACTCCCGCTCTGTGTTGCTGGCGGTGCGGGCTCTGGGTCTGGACGTGGAGCTGAAAACCATCGACTTGAAGAAAAAGGAACAGCTCAACCCGGATTTTGTGGCAATCAACCCGCAGCACACCGTTCCTACCCTGGTGGACGGCGACCTTGTGCTGACTGAGAGCCGCGCCATCAGCACCTATTTGGTCTCTCGCTACGGAAAGGACGACTCTCTCTACCCGAAGGACGTCGTTACAAGGGCTAAGGTGGATGGACTTCTCTACTTCGACTGCGCCACCCTCTGCATTCGTTGGCGCGCTGTCGTGCACCCAGTCATGAAGGACGGAGCAGCCAAACCCAGCGAGGCAGCAATGAAGAACCTAGACGAGGCTTTGAAGTGGCTGAACGACATACTTGCCCAACATCCTGACCGCTACCTGGCCGGCACCACGCACCCCACCGTCGCGGACCTCTCGATCGCTGCCTGGGCTTGTACATATGTTGCTCTGAACTTCCCTCTGGCCCAGCACCCGGACATCACGCGCTGGCTGGCTCGCTGCAGGGAGAATCTTGTTGGCTTCGACGAGTGTGAGAAGGGCGCGGCGCAGTTTGGGGAGATGTTCGCAGCTATTCTGAAGTCAAGAGCTTGA

MAPTLYYFLMSPYSRSVLLAVRALGLDVELKTIDLKKKEQLNPDFVAINPQHTVPTLVDGDLVLTESRAISTYLVSRYGKDDSLYPKDVVTRAKVDGLLYFDCATLCIRWRAVVHPVMKDGAAKPSEAAMKNLDEALKWLNDILAQHPDRYLAGTTHPTVADLSIAAWACTYVALNFPLAQHPDITRWLARCRENLVGFDECEKGAAQFGEMFAAILKSRA

transcript_transcript/28810_Pt_Mix_transcript28810/f7p0/1259

ATGGCCCCTACACTGTACTACTTCTTGATGTCGCCTTACTCCCGCTCTGTGTTGCTGGCGGTGCGGGCTCTGGGTCTGGACGTGGAGCTGAAAACCATCGACTTGAAGAAAAAGGAACAGCTCAACCCGGATTTTGTGGCAATCAACCCGCAGCACACCGTTCCTACCCTGGTGGACGGCGACCTTGTGCTGACTGAGAGCCGCGCCATCAGCACCTATTTGGTCTCTCGCTACGGAAAGGACGACTCTCTCTACCCGAAGGACGTCGTTACAAGGGCTAAGGTGGATGGACTTCTCTACTTCGACTGCGCCACCCTCTGCATTCGTTGGCGCGCTGTCGTGCACCCAGTCATGAAGGACGGAGCAGCCAAACCCAGCGAGGCAGCAATGGAGAACCTAGACGAGGCTTTGAAGTGGCTGAACGACATACTTGCCCAACATCCTGACCGCTACCTGGCCGGCACCACGCACCCCACCGTCGCGGACCTCTCGATCGCTGCCTGGGCTTGTACATATGTTGCTCTGAACTTCCCTCTGGCTCAGCACCCGGACATCACGCGCTGGCTGGCTCGCTGCAGGGAGAATCTTGTTGGCTTCGACGAGTGTGAGAAGGGCGCGGCGCAGTTTGGGGAGATGTTCGCAGCTATTCTGAAGTCAAGAGCTTGA

MAPTLYYFLMSPYSRSVLLAVRALGLDVELKTIDLKKKEQLNPDFVAINPQHTVPTLVDGDLVLTESRAISTYLVSRYGKDDSLYPKDVVTRAKVDGLLYFDCATLCIRWRAVVHPVMKDGAAKPSEAAMENLDEALKWLNDILAQHPDRYLAGTTHPTVADLSIAAWACTYVALNFPLAQHPDITRWLARCRENLVGFDECEKGAAQFGEMFAAILKSRA

transcript_transcript/29982_Pt_Mix_transcript29982/f2p0/1240

ATGGCGTGGCTGAGCACTGAATTTTACTTCTATAAGGGGAGCGCTCCGTGTCGCGCCGTGTGGATGACGCTCAAGATGCTCAATGTGGAGTACGAGCCTAAGGAAGTGGATCTCCTGAAGGCTGAGAACAAGCGGCCATGGTTTTTGAGGCTCAACCCGCAGCACACTGTTCCCACTCTGACCGAGGGAGATTTCGCTCTATGGGAGAGCCGCGCCATTATGATGTACCTAATGAACAAATACGCCACGGAGGAGAACCAGTATCTGTACCCGAAGGAGCCTGAGGAGAGGGCGAAGGTGGACCGCATGTTATTCTTCGACATGGGAACGCTCTACCACTCGATCAAGGAGTACTTTGCCCCCAAGATATACGACGGCCTGCCCCCGGACCCAGAGAAGGAGAACCTGCTGAAGACAAGCCTGGATCACCTGGACCACTTCCTGGAGATCGGTGGCGTGCCTTACCTGTGCGGGGAGAGGATCACTCTCGCTGACGTGGCTGTCCTCGCCTCCGTCACTGAGCTGGACGCTATGGAATACAACTACAGGTGTTACGGGGAGTTCAACAGATGGGTGGAGCGAGTGAAGGCCGCCATCAAGCCTTACAAGGAGTGCTGCGAGGAGGGCGTGCGGATGACCAAAGACCGCACCAAGTTCCTGGAGACTGAGGCCAAGAAGCAGATGGAGAAGGAACGCAAGGCTTCCCTCCCCAAGAAGTAA

MAWLSTEFYFYKGSAPCRAVWMTLKMLNVEYEPKEVDLLKAENKRPWFLRLNPQHTVPTLTEGDFALWESRAIMMYLMNKYATEENQYLYPKEPEERAKVDRMLFFDMGTLYHSIKEYFAPKIYDGLPPDPEKENLLKTSLDHLDHFLEIGGVPYLCGERITLADVAVLASVTELDAMEYNYRCYGEFNRWVERVKAAIKPYKECCEEGVRMTKDRTKFLETEAKKQMEKERKASLPKK

transcript_transcript/32846_Pt_Mix_transcript32846/f18p0/959

ATGCCCATTGACTTTTACTACCTGCTCATCTCCCCGCCCTGCCGCGCTGTGATGCTGACGGCCGAGGCGGTGGGTGTGAAGCTCAACATGAAGGAGCTGGACATTTTCAAGGGGGAGCAGATGAAGCCAGAGTTTGTGGCCCTCAATCCTCAGCACTGCATCCCCACACTGGTGGATGGCGACTTTGTCATCTGGGAGAGCCGACCCACATGCTCCTACCTGGCATCAAAGTATGGCAAGGATGACTCGCTCTTCCCAAATGATCCTCGTGCCAGAGCTGAAGTGGAACGACTCAATTACTTTGACATGGGCACACTCTTCCATCGCTTCGGGGAGTATGTGTTCCCAGTAATGTTCAGAGGTGAGAAGGAATTCAACCCAGACAAGCTGGAACGTTTACAGGAAGCTCTTGGTTGGCTGGATGGCTTCCTCTCGGGCAACAAGTTTGCTGTTGGTGACAATATTACCATTGCTGACCACACTCTCTTGGCCACTGTCTCTACCATAAAGGAGGCAAATGTGGACTTGAGCAAGCATGCCAACATCCTTGCCTGGCTGGAGAAGTGCAAGGCTGAGGTGCCAGGCTATGAGACCAATCAGAAGGGTGCTGAGGATTGGGGAAAGTTCTTCAAGTCTCGTTGCAACCTGTAA

MPIDFYYLLISPPCRAVMLTAEAVGVKLNMKELDIFKGEQMKPEFVALNPQHCIPTLVDGDFVIWESRPTCSYLASKYGKDDSLFPNDPRARAEVERLNYFDMGTLFHRFGEYVFPVMFRGEKEFNPDKLERLQEALGWLDGFLSGNKFAVGDNITIADHTLLATVSTIKEANVDLSKHANILAWLEKCKAEVPGYETNQKGAEDWGKFFKSRCNL

transcript_transcript/32963_Pt_Mix_transcript32963/f4p0/981

ATGCGCCCTGTGCTCGGCTACTGGAAAACTCGCTGTCTCGCTCAGCCCATTCGCCTCCTACTGGCGTACAAGAGGGTAAATTACGAAGACAAGCAGTACGAGACAGGAGACCCTCCGGAGTACGACAAGACGTGCTGGTTCTCCGTCAAGTTCAATCTCGGCCTTGACTTCCCAAATCTTCCTTACTACATAGACGGCGACGTGAAGCTCAGTCAGACCCTCGCTATTCTCCGCTACCTCGGCCGCAAGTATGGCCTGGAGGGGAAGACAGAGGAGCAGATGAGGCGAATCGACATCTTAGTGAACGATGCAATGGATTTTGAGATGCAGTTCGTGGATGTCGTCTATTACCATTACGACCGGAAGCCAGAGTACTTGAAAAAACTACCTGAAAAGATTAAAGAATATTCAGATTTCCTTGGTCATCATTCTTGGTTTGCCGGAGAAGAGTTGACATTCGCGGATTTCCTGATTTACGAATTCCTAGATCAACACCGTGTCGTTTTTCCCTCTTGTCTGGACGCCACCCCGCCCCTGCAACGCTTCATGACTCGATTTGAAAGTCTGGAATCTATCCGCGAGTACATGGCAGGTCCCACCTTCATGACGGCGCCGCTTTTCAGTAAGTATTCCGCTTATGAGATCGAGCAGCGGGCGAAGAAAATGACTTGA

MRPVLGYWKTRCLAQPIRLLLAYKRVNYEDKQYETGDPPEYDKTCWFSVKFNLGLDFPNLPYYIDGDVKLSQTLAILRYLGRKYGLEGKTEEQMRRIDILVNDAMDFEMQFVDVVYYHYDRKPEYLKKLPEKIKEYSDFLGHHSWFAGEELTFADFLIYEFLDQHRVVFPSCLDATPPLQRFMTRFESLESIREYMAGPTFMTAPLFSKYSAYEIEQRAKKMT

transcript_transcript/33102_Pt_Mix_transcript33102/f2p0/971

ATGAGGGTCGCAGCGGCCAGAGCAGCGGTTCACGCCGCCAAAGAAAAAGTGAAGATTGATTTGTTCTATGATGTGGTATCTCCCTATGCATGGCTTGGATTTGAGGCTCTCAATAGGTACAAGACACACTGGAACATTGATCTACACCTGAAGCCTGTGCTTCTTGCTGGCATTGGAAAGCATGCAGGGACTATTCCACCAGCTATGAAACCCAACAGAGCTCCCTACTTAGTGAAGGATTTGCTTCGCTCAGCAACCTACTTTAATGTACCTCTCAAGTTTCCGAGCGACGTTTTTGAGTTGATGTTTGGTAAGGGTTCTCTCATCCCTTCTCGCTTCCTGACTGCCATCGGCCTGTTGTATCCTGCTCATCTGGAGGCTGCAAGCCGCCAGTTGTGGTTGGCAGCTTGGAGTAGGGATGAGGACATCACATCGCCTGAGGTGTTAGCAGCAGCAGGCTTAGCAGCAGGTCTGACACCAGAAACTCTTGCGGAGGTGAAGGAACAGATGGTTCAGCCAGTCATCAAGCAACGTCTGAAAGCCCACACTGATGAGGCTGTTGGATATGGGGCTTTTGGAGTGCCCACTATAGTTGCCCATGTCGGGGAAAAACCAGCACTTTTCTTTGGGTCTGACAGGTTCCCCATTCTGGCTCAAGAAATCAATGAGACCTGGATGGGTCCTGAACCAGGTGTAGAAAATGCAAAGCTTTGA

MRVAAARAAVHAAKEKVKIDLFYDVVSPYAWLGFEALNRYKTHWNIDLHLKPVLLAGIGKHAGTIPPAMKPNRAPYLVKDLLRSATYFNVPLKFPSDVFELMFGKGSLIPSRFLTAIGLLYPAHLEAASRQLWLAAWSRDEDITSPEVLAAAGLAAGLTPETLAEVKEQMVQPVIKQRLKAHTDEAVGYGAFGVPTIVAHVGEKPALFFGSDRFPILAQEINETWMGPEPGVENAKL

transcript_transcript/3324_Pt_Mix_transcript3324/f4p0/3790

ATGGCGTGGCTGAGCACTGAATTTTACTTCTACAAGGGGAGCGCTCCGTGTCGCGCCGTGTGGATGACGCTCAAGATGCTCAATGTGGAGTACGAGGCTAAGGAAGTGGATCTCCTGAAGGCTGAGAACAAGCGGCCATGGTTTTTGAGGCTCAACCCGCAGCACACTGTTCCCACTCTGACCGAGGGAGATTTCGCTCTATGGGAGAGCCGCGCCATTATGATGTACCTAATGAACAAATACGCCACGGAGGAGAACCAGTATCTGTACCCGAAGGAGCCTGAGGAGAGGGCGAAGGTGGACCGCATGTTATTCTTCGACATGGGAACGCTCTACCACTCGATCAAGGAGTACTTTGCCCCCAAGATATACGACGGCCTTCCCCCGGACCCAGAGAAGGAGAACCTGCTGAAGACAAGCCTGGATCACCTGGACCACTTCCTGGAGATCGGTGGCGTGCCTTACCTGTGCGGGGAGAGGATCACTCTCGCTGACGTGGCTGTCCTCGCCTCCGTCACTGAGCTGGACGCTATGGAATACAACTACAGGTGTTACGGGGAGTTCAACAGATGGGTGGAGCGAGTGAAGGCCGCCATCAAGCCTTACAAGGAGTGCTGCGAGGAGGGCGTGCGGATGACCAAAGACCGCACCAAGTTCCTGGAGACTGAGGCCAAGAAGCAGATGGAGAAGGAACGCAAGGCTTCCCTCCCCAAGAAGTAA

MAWLSTEFYFYKGSAPCRAVWMTLKMLNVEYEAKEVDLLKAENKRPWFLRLNPQHTVPTLTEGDFALWESRAIMMYLMNKYATEENQYLYPKEPEERAKVDRMLFFDMGTLYHSIKEYFAPKIYDGLPPDPEKENLLKTSLDHLDHFLEIGGVPYLCGERITLADVAVLASVTELDAMEYNYRCYGEFNRWVERVKAAIKPYKECCEEGVRMTKDRTKFLETEAKKQMEKERKASLPKK

transcript_transcript/34265_Pt_Mix_transcript34265/f3p0/870

ATGTCCGCCAGCCTTACTCTCTACATTGACTACGTGTCCCAGCCTTCCCGTTCCCTAGTGTTTCTGTGTAGGGCTATTAAGGCGCCCCACCAGGAGAAGGTAGTCAGTCTGGTGAAAGGGGAGCACCAGAACAAGGCTTACGCCGACGTCAACCCCTTCAAGAAAGTCCCGGCGGTGCAGGATGGAGACTTGCTTATCCTAGAGAGTTGCTCTGCCCTGCGGTACATCGCTAGCAAGTATGACGCGGCCGGCACCTGGTACCCCTCTGACCTGAAGACGCGCTGCAAGGTGGATGAGTACCTGGACTGGCAACACCTCAACACCAGAGCTCACGGCGTCGGCTACTACATGAATAAGGTGTTGGTACCCATGATGAAGGGCTCACCACCAGACATGGCATTGGTGGAGAAACACGAGAAGGAGATGGGCCGCGTGGAGGAGCTCTTTGCCAGCTACTTCCTGGGCGACAAGCCGTTCATCACTGGCAACACGCCCACCATCGCGGATCTGCAGGCGGCCACGGAGTTTGAGCAGCCCCAAGCAGCAGGGTACACGTTGGCCAAGACCACGAAGGAGTACCTGGAGAGGGTGAGGGAGGCGGTGGGCGCAGAGATGTACGACTCACTGCACGAGGCGCCCAAGGAACTGGCGAAGAAGGCGCTGCAGTGA

MSASLTLYIDYVSQPSRSLVFLCRAIKAPHQEKVVSLVKGEHQNKAYADVNPFKKVPAVQDGDLLILESCSALRYIASKYDAAGTWYPSDLKTRCKVDEYLDWQHLNTRAHGVGYYMNKVLVPMMKGSPPDMALVEKHEKEMGRVEELFASYFLGDKPFITGNTPTIADLQAATEFEQPQAAGYTLAKTTKEYLERVREAVGAEMYDSLHEAPKELAKKALQ

transcript_transcript/22646_Pt_Mix_transcript22646/f41p0/1649

ATGTTGCCTCGACGTCTGTCCCGCCTGGTGCAACCCGTGAGGCGCCTGGGCACCTGCACCACCTCCAGGGCCTCCTTCACCGTACAGGACGAGGAGGACTTCAGAAACCGGGTGCTGTCCTCCTCCACTCCCGTCGTCGTCGATTTTCATGCCCAGTGGTGTGGGCCGTGCAAGCTGCTGGGACCTCGGCTGGAAACTATCATTGGTGGAAAGGGCGAGAAGGTCCACCTGGCGAAGGTTGACATTGATAACGTGTCAGACTTGGCACTTGACTATGGGGTGTCAGCTGTACCCTCAGTCCTGGCGGTGAAGGATGGCAAGGTGGTGGACAAGTTCGTTGGCCTTCAAGAGGAGTCGAGGATTGAGGCCTTCGTTAATCGGCTCATTGGGGAGTAG

MLPRRLSRLVQPVRRLGTCTTSRASFTVQDEEDFRNRVLSSSTPVVVDFHAQWCGPCKLLGPRLETIIGGKGEKVHLAKVDIDNVSDLALDYGVSAVPSVLAVKDGKVVDKFVGLQEESRIEAFVNRLIGE

transcript_transcript/26350_Pt_Mix_transcript26350/f7p0/1479

ATGCTTAACAACCCAGCAGTTCGATCTCTGCTACAGCAGGTAGTGGCAAAACCTGTTGTCGGGCGACATGCGCAACAGCTTGCATGCACTGTGGGCAGGAATTTCCACAGTAGTGAGCCTGCCATGGAAATTTTTATGGTAAATAGTGAAGATGATTTTAAGAACAAAGTCATGCGAAGTCCTGTTCCAGTCATAGTGAACTTTCATGCCGAGTGGTGTGAGCCATGTCATTCCCTCAAGCCTCTGTTGGAGAAGATTGCTCAAGAAAATGAAGGTCACCTCCACTTGGCTGAGGTGGCAGTGGATGAACACCTTGATCTGCTGCATGCCTTTGAGGTTACTGCAGTGCCAGCAGTGTTGGGTATTCATCGGGGGATGGTGGTTGAGAAGTTTGTTGGTCTAGTATCTCACAAAGAAGTTAAAGGTTTTGTTGATAAATTGCTAAATAAGTAG

MLNNPAVRSLLQQVVAKPVVGRHAQQLACTVGRNFHSSEPAMEIFMVNSEDDFKNKVMRSPVPVIVNFHAEWCEPCHSLKPLLEKIAQENEGHLHLAEVAVDEHLDLLHAFEVTAVPAVLGIHRGMVVEKFVGLVSHKEVKGFVDKLLNK

transcript_transcript/30081_Pt_Mix_transcript30081/f69p0/1146

ATGGTCTACCAAGTGAAGGATAAGGAAGACTTTGACCAGCAGTTGAAGAATGCTGGACAGAAGCTTGTGGTGGTAGACTTCTATGCCACCTGGTGCGGACCCTGCAAGATCATCGCCCCCAAGATTCAGGAGATGAGTGAGCAGATGAGTGACGTTGTGTTCCTGAAGGTGGACGTGGATGAGAATGACGAGGTTGCCGTGACCTATAAGGTGTCTTGCATGCCTACCTTCGTTTTCTTCAAGGCGGAGAAGAAAGTGGACAGTTTCTCAGGAGCAAGTGAAGACAAGCTCCGTGAATTCATTGCAAAGCTGAAGTAA

MVYQVKDKEDFDQQLKNAGQKLVVVDFYATWCGPCKIIAPKIQEMSEQMSDVVFLKVDVDENDEVAVTYKVSCMPTFVFFKAEKKVDSFSGASEDKLREFIAKLK

transcript_transcript/35181_Pt_Mix_transcript35181/f29p0/758

ATGGCTGGTATGCTGAGAAGGTTCGTGCCTCTGTTTGACCGTGTGCTGGTGCAGAAGGCTGAGGTGGTCACCAAGACAGCCTCAGGCATCTTCATCCCGGAGAAGTCCCAGGCGAAGGTGCTGACGGGCAAAGTGGTGGCGGTGGGTGAGGGGCTGCGCACAGAGAGTGGCAGCGTAGTGGCCCCAGCAGTGTCAGTCGGGGACGAGGTGCTGCTGCCTGAGTTTGGAGGCACCAAGGTGACACTAGAGGAGAAGGACTACTTCCTCTTCAGGGACTCTGACATCCTGGCCAAGATGAAGAGCGAGTAA

MAGMLRRFVPLFDRVLVQKAEVVTKTASGIFIPEKSQAKVLTGKVVAVGEGLRTESGSVVAPAVSVGDEVLLPEFGGTKVTLEEKDYFLFRDSDILAKMKSE

transcript_transcript/20595_Pt_Mix_transcript20595/f22p0/1779

ATGGGAAAGGACTACTACAAGATCCTGGGGCTGTCTAAAGGGGCCTCTGAGGAAGACATAAAGAAGGCCTACAGGAAGATGGCCCTCAAATACCATCCCGACAAAAACAAATCAGCAGATGCCGAGGAAAAGTTTAAGGCAGTGGCAGAGGCTTACGAGGTGCTGAGTGACAAAAAGAAGCGGGACATCTACGACCAGTATGGCGAGGAGGGGCTGAAGGGCGGTGTGCCTGGCGGCCCCGGGGAGGATGGCACACACTTCACCTACACCTTCTCCGGGGACCCGCGTGCCACCTTCCACGAGTTCTTTGGAACCAATGATCCCTTCGCTCACTTCTTCAACATGGGTGTGGACGGACATGGGGGTCACGGAGTGTTTGAAGACATGGACGTGGAGGACGACCCCTTTATTACCATGATGGGCGGCGGCGGGCCTCGCTTGGGCGGCACTCGAAGGGCGTTCAGCTTCAACCCGCACGATGCTACTAGGCCACAGGGCGGGAAGGGGCGCCACCAGGATCCTGCCGTCACCAGAGACCTGTACGTCACTTTAGAGGAGGTGTCGAAGGGGGTCACGAAGAAGATGAAGATCACTAGGAATGTGTTGGCGAGTGACGGGCGCTCTACACGTCGAGAGGAGAAGATATTGACAATTGAAGTGAAACCTGGGTGGAAGGAAGGCACCAAGATTACTTTCGAGCGAGAGGGTGACCAGACCCCGGGCAAGATTCCCGCCGACATTATCTTCATGATCAGAGACAAACCTCACCACCACTTCAAGCGTGACGGCGCCAACCTGATCTATACAGCCAAGGTGGCTCTCCGAGATGCATTGTGTGGTACCAGGGTGTCGGTGCCCACTCTGGATAATCAGAGAGTGGCACTCAATCTAACAAATGAGGTGGTGCGGCCCCAGACCACTAAACGCCTTCAGGGCTACGGCTTGCCTTACCCTAAGGACACCCAGAGGAAGGGTGACATCGTCGTTCACTTCGACATTCAGTTTCCTTCTACGTTGACTGAAAGTGCGAAAGAAATTTTGTCTGAAGTCCTCCCTCCGTAA

MGKDYYKILGLSKGASEEDIKKAYRKMALKYHPDKNKSADAEEKFKAVAEAYEVLSDKKKRDIYDQYGEEGLKGGVPGGPGEDGTHFTYTFSGDPRATFHEFFGTNDPFAHFFNMGVDGHGGHGVFEDMDVEDDPFITMMGGGGPRLGGTRRAFSFNPHDATRPQGGKGRHQDPAVTRDLYVTLEEVSKGVTKKMKITRNVLASDGRSTRREEKILTIEVKPGWKEGTKITFEREGDQTPGKIPADIIFMIRDKPHHHFKRDGANLIYTAKVALRDALCGTRVSVPTLDNQRVALNLTNEVVRPQTTKRLQGYGLPYPKDTQRKGDIVVHFDIQFPSTLTESAKEILSEVLPP

transcript_transcript/3641_Pt_Mix_transcript3641/f14p0/3701

ATGGTGAAGGAAACGGGTTACTATGACATGCTTGGGGTGAAACCCACTGCATCACAGGATGAACTCAAAAAAGCATACAGGAAATTGGCCCTCAAGTACCATCCTGACAAAAATCCAAATGAAGGAGAAAAGTTCAAGTTGATCTCACAGGCATACGAAGTCCTTAGCAATGAAGAAAAGCGCAAAATTTATGATCAGGGAGGAGAACAGGCCCTCAAAGAAGGAGGTAGTGGAGGTGGAAGCTTCTCCTCTCCCATGGACATCTTTGATATGTTCTTCGGTGGAGGAATGAGGCGAGGACGAGAAAAGAGAGTGAAGGATGTCATTCACCAAATGAGTGTTTCATTAGAGGAACTTTACAACGGAGCTACTCGAAGATTGGCTCTGCAGAAGCATGTTATCTGCAACAAATGCGAAGGCCAGGGCGGAAAGAAACCCCCGGAGAAGTGCCCTTCATGCAGAGGAACTGGAATGCAGGTCCGAATTCAGCAGCTAGGGCCAGGCATGGTATCCCAGGTGCAGAGTATGTGCGGAGAATGTCGAGGGCAGGGTGAACGTATCAATCCTAAGGACAGGTGTAAGGCATGTGAGGGCAGAAAGGTGGTCAAAGAGAGAAAGATCTTAGAGGTTCACGTGGACAAGGGCATGGAAGATGGACAGAAGATTGTGTTCTCAGGTGAAGGGGACCAAGAACCAGGCCTTGAGCCAGGAGATATTATTATTGTATTAGATGAGAAAGAGCACCCAACTTTCAGACGTGTCGTCAATGATCTCACAATGCAGATGCACATAACTCTTGTGGAAGCACTGTGCGGCTTCCAGAAACCCATTAAAACTTTAGATGATAGGACTATTGTTGTTAGCACCATTCCTGGTGAAGTGATCAAGAATGCTGAAATCAAGGCTGTGTTAGGTGAGGGCATGCCTCAATACAAGAATCCATTTGAAAAGGGACGTTTGCTCCTCCAGTTCCTCGTAGACTTCCCATCACAAATTCCTCTAGATCGAATCCGAAAGCTAGAGAAGATTCTCCCAACCAGACCTGAGGTAATAGTCCCAGATGATGGCGAGGAAGTGAACCTTGTGGAGTTGGACCACAATCAGCGAGGAAGGCGTCACAACCACATCTATGATGATGATGATGATGACCATCATGGAGGACGGCAACATGTCCAGTGCCAGACAAGCTAG

MVKETGYYDMLGVKPTASQDELKKAYRKLALKYHPDKNPNEGEKFKLISQAYEVLSNEEKRKIYDQGGEQALKEGGSGGGSFSSPMDIFDMFFGGGMRRGREKRVKDVIHQMSVSLEELYNGATRRLALQKHVICNKCEGQGGKKPPEKCPSCRGTGMQVRIQQLGPGMVSQVQSMCGECRGQGERINPKDRCKACEGRKVVKERKILEVHVDKGMEDGQKIVFSGEGDQEPGLEPGDIIIVLDEKEHPTFRRVVNDLTMQMHITLVEALCGFQKPIKTLDDRTIVVSTIPGEVIKNAEIKAVLGEGMPQYKNPFEKGRLLLQFLVDFPSQIPLDRIRKLEKILPTRPEVIVPDDGEEVNLVELDHNQRGRRHNHIYDDDDDDHHGGRQHVQCQTS

transcript_transcript/9745_Pt_Mix_transcript9745/f4p0/2746

ATGTACCGCGCTGCCTCCCTGCTCCGGCTGCCCGCGAGTCGCCAGGTCGCCCAACGGCTGGCGACGAGGAGCTACGCCAAGGATGTCAAGTTTGGGTCAGAGGTGCGGGCCATGATGCTGCAGGGCGTGGACGTGCTCACTGATGCTGTGGCCGTCACTATGGGCCCCAAGGGTCGCAATGTGATAATAGAGCAGAGCTGGGGCAGCCCTAAAATCACCAAGGATGGCGTGACGGTGGCCAAGGCGGTGGAGCTGAAGGACAAGTTCCAGAACATTGGTGCCAAGCTGGTGCAGGACGTGGCCAACAACACCAATGAGGAGGCCGGCGATGGCACCACCACTGCCACCGTGCTGGCCCGCACTATTGCCAAGGAGGGCTTCGACAAGATCAGCAAGGGAGCTAACCCTATTGAAATCAGGCGTGGTGTGATGCTGGCAGTGGAGGCTGTCATCGACCACCTCCGCTCACTGTCCCGCCAGGTGACCACCCCTGCTGAGATCACCCAGGTGGCCACCATCTCTGCTAATGGGGACAGCGAAGTGGGAGAGCTCATCTCAGCTGCCATGGAGAAGGTTGGGCGCAATGGAGTGATCACGGTGAAGGACGGCAAGACACTGAAGGACGAGCTAGAGGTGATCGAGGGCATGAAGTTTGATAGGGGTTACATCTCCCCGTACTTCATCAACACAGCTAAGGGGGCCAAGGTGGAGTACCAGGATGCCTTGGTGCTCCTCTCAGAAAAGAAGATCTCCTCCATTCAGTCCATCATCCCAGCGCTGGAGATTGCCAACGCTCAGAGGAAGCCACTGCTCATTATTGCTGAGGACGTGGATGGGGAAGCACTGAGCACCCTGGTGGTGAACCGGCTCAAGATTGGCCTGCAGATTGCTGCTGTCAAGGCCCCAGGCTTTGGTGACAACCGCAAGAACACTATCCAGGACATTGCCATCGCCACCGGGGCCCTCGTCTTCAATGATGAGGCCAGCATGGTGAAGATTGAGGATGTGCAGGCACACGACCTTGGCATGGTGGGTGAGGTGCAGATCACCAAGGACGATACGTTGCTGCTGAAGGGCAAGGGCAAGTCATCAGATATTGAGAGGAGGATTGGCCAGATCAGGGAGCAGATTGAGGACAGCAACTCTGAGTATGAGAAAGAGAAGATGCAGGAGCGAATGGCACGACTCAGCAACGGAGTGGCGGTGGTGAAGGTGGGCGGCTCCTCTGAGGTGGAGGTCAATGAGAAGAAGGATCGTGTAAATGATGCCCTGTGTGCCACACGTGCCGCCATTGAAGAGGGCATCGTGCCTGGTGGTGGTGTGGCACTGCTGCGCTGCTTGCCTGCCCTGGATGCTGTCAAGGCTGCCAATGAGGACCAGAAGATTGGCGTGGACATCATCCGTAAGGCCATTAGGACGCCCTGCTACACCATTGTCACCAACGCTGGCATTGATGCCGCCGTCATCGTCAACAAGGTGGAGGAAGCGACGGGCGACTATGGGTACGATGCTGCCAACGGCACCTTTGTCAATCTAGTGGAGGCCGGCATCATTGACCCCACCAAGGTGGTGCGGTCTGCCCTGACTGATGCTGCTGGTGTGGCCTCTCTCCTCACCACTGCCGAGTCTGTCATCACTGAGATCCCCAAGGAGGAGCCAGCAGGCGGCGGCATGGGTGGCATGGGCGGTATGGGAGGTATGGGAGGAATGGGTGGCATGGGAGGCATGATGTGA

MYRAASLLRLPASRQVAQRLATRSYAKDVKFGSEVRAMMLQGVDVLTDAVAVTMGPKGRNVIIEQSWGSPKITKDGVTVAKAVELKDKFQNIGAKLVQDVANNTNEEAGDGTTTATVLARTIAKEGFDKISKGANPIEIRRGVMLAVEAVIDHLRSLSRQVTTPAEITQVATISANGDSEVGELISAAMEKVGRNGVITVKDGKTLKDELEVIEGMKFDRGYISPYFINTAKGAKVEYQDALVLLSEKKISSIQSIIPALEIANAQRKPLLIIAEDVDGEALSTLVVNRLKIGLQIAAVKAPGFGDNRKNTIQDIAIATGALVFNDEASMVKIEDVQAHDLGMVGEVQITKDDTLLLKGKGKSSDIERRIGQIREQIEDSNSEYEKEKMQERMARLSNGVAVVKVGGSSEVEVNEKKDRVNDALCATRAAIEEGIVPGGGVALLRCLPALDAVKAANEDQKIGVDIIRKAIRTPCYTIVTNAGIDAAVIVNKVEEATGDYGYDAANGTFVNLVEAGIIDPTKVVRSALTDAAGVASLLTTAESVITEIPKEEPAGGGMGGMGGMGGMGGMGGMGGMM

transcript_transcript/9817_Pt_Mix_transcript9817/f4p0/2723

ATGTACCGCGCTGCCTCCCTGCTCCGGCTGCCCGCGAGTCGCCAGGTCGCCCAACAGCTGGCGACGAGGAGCTACGCCAAGGATGTCAAGTTTGGGTCAGAGGTGCGGGCCATGATGCTGCAGGGCGTGGACGTTCTCACTGATGCTGTAGCCGTTACTATGGGCCCCAAGGGTCGCAATGTGATAATAGAGCAGAGCTGGGGCAGCCCCAAAATCACCAAGGATGGCGTGACGGTGGCCAAGGCGGTGGAGCTGAAGGACAAGTTCCAGAACATTGGTGCCAAGCTGGTGCAGGACGTGGCCAACAACACCAATGAGGAGGCCGGCGATGGCACCACCACTGCCACCGTGCTGGCCCGCACTATTGCCAAGGAGGGCTTCGACAAGATCAGCAAGGGAGCTAACCCCATTGAAATCAGGCGTGGTGTGATGCTGGCAGTGGAGGCTGTCATCGACCACCTCCGCTCACTGTCCCGCCAGGTGACCACCCCTGCTGAGATCACCCAGGTGGCCACCATCTCTGCTAATGGGGACAGCGAAGTGGGAGAGCTCATCTCAGCTGCCATGGAGAAGGTTGGGCGCAATGGAGTGATCACGGTGAAGGACGGCAAGACACTGAAGGACGAGCTAGAGGTGATCGAGGGCATGAAGTTTGATAGGGGTTACATCTCCCCGTACTTCATCAACACAGCTAAGGGGGCCAAGGTGGAGTACCAGGATGCCTTGGTGCTCCTCTCAGAAAAGAAGATCTCCTCCATTCAGTCCATCATCCCAGCGCTGGAGATTGCCAATGCTCAGAGGAAGCCACTGCTCATTATTGCTGAGGACGTGGATGGGGAAGCACTGAGCACCCTGGTGGTGAACCGGCTCAAGATTGGCCTGCAGATTGCTGCTGTCAAGGCCCCAGGCTTTGGTGACAACCGCAAGAACACTATCCAGGACATTGCCATCGCCACCGGGGCCCTCGTCTTCAATGATGAGGCCAGCATGGTGAAGATTGAGGATGTGCAGGCACACGACCTTGGCATGGTGGGTGAGGTGCAGATCACCAAGGACGATACGTTGCTGCTGAAGGGCAAGGGCAAGTCATCAGATATTGAGAGGAGGATTGGCCAGATCAGGGAGCAGATTGAGGACAGCAACTCTGAGTATGAGAAAGAGAAGATGCAGGAGCGAATGGCACGACTCAGCAACGGAGTGGCGGTGGTGAAGGTGGGCGGCTCCTCTGAGGTGGAGGTCAATGAGAAGAAGGATCGTGTAAATGATGCCCTGTGTGCCACACGTGCCGCCATTGAAGAGGGCATCGTGCCTGGTGGTGGTGTGGCACTGCTGCGCTGCTTGCCTGCCCTGGATGCTGTCAAGGCTGCCAATGAGGACCAGAAGATTGGCGTGGACATCATCCGTAAGGCCATTAGGACGCCCTGCTACACCATTGTCACCAATGCTGGCATTGATGCCGCCGTCATCGTCAACAAGGTGGAGGAAGCGACGGGCGACTATGGGTACGATGCTGCCAACGGCACCTTTGTCAATCTAGTGGAGGCCGGCATCATTGACCCCACCAAGGTGGTGCGGTCTGCCCTGACTGATGCTGCTGGTGTGGCCTCTCTCCTCACCACTGCCGAGTCTGTCATCACTGAGATCCCCAAGGAGGAGCCAGCAGGCGGCGGCATGGGTGGCATGGGCGGTATGGGAGGTATGGGAGGAATGGGTGGCATGGGAGGCATGATGTGA

MYRAASLLRLPASRQVAQQLATRSYAKDVKFGSEVRAMMLQGVDVLTDAVAVTMGPKGRNVIIEQSWGSPKITKDGVTVAKAVELKDKFQNIGAKLVQDVANNTNEEAGDGTTTATVLARTIAKEGFDKISKGANPIEIRRGVMLAVEAVIDHLRSLSRQVTTPAEITQVATISANGDSEVGELISAAMEKVGRNGVITVKDGKTLKDELEVIEGMKFDRGYISPYFINTAKGAKVEYQDALVLLSEKKISSIQSIIPALEIANAQRKPLLIIAEDVDGEALSTLVVNRLKIGLQIAAVKAPGFGDNRKNTIQDIAIATGALVFNDEASMVKIEDVQAHDLGMVGEVQITKDDTLLLKGKGKSSDIERRIGQIREQIEDSNSEYEKEKMQERMARLSNGVAVVKVGGSSEVEVNEKKDRVNDALCATRAAIEEGIVPGGGVALLRCLPALDAVKAANEDQKIGVDIIRKAIRTPCYTIVTNAGIDAAVIVNKVEEATGDYGYDAANGTFVNLVEAGIIDPTKVVRSALTDAAGVASLLTTAESVITEIPKEEPAGGGMGGMGGMGGMGGMGGMGGMM

transcript_transcript/12237_Pt_Mix_transcript12237/f59p0/2463

ATGATGTCCAAAGCAAGGTGTCTGGCCACTAATCTCGGTGCCCTTAGTGCTAGGAAGAATGTGGCGCAGCTCCTACAACAGAGCTCAGTGCAAGGGGTGCTGGCCCAACACAATGCCCTCAACATGCAGACCAGATACAAGTCCGATGGGGTGAAGGGAGCAGTGATCGGCATTGACCTGGGAACAACAAACTCCTGCGTGGCCGTCATGGAGGGCAAGACTGCAAAGGTGATCACCAATGCAGAGGGAGACCGCACCACTCCTTCTGTCATTGCATTCACTAAGGATGGGGAGCGTCTGGTGGGCATTCAGGCCAAGCGTCAGGCAGTCACCAATGCAGGGAACACCTTCTATGCCACAAAGCGACTCATTGGCCGGAGATACGATGACGAGGAAATCGTCAAGGACAGGGAGACAGTGCCTTTCAAGATAGTGAAGGCCAGTAATGGAGATGCCTGGGTACAGACAGACGATGGCAAGATGTACTCCCCATCTCAGGTTGGAGCCTTTGTCCTCACCAAGATGAAGGAGACCGGTGAGGCATATCTCAACACCCCCGTGAAAAATGCTGTCATCACTGTGCCGGCTTACTTCAATGACTCCCAGAGACAGGCCACCAAGGACGCTGGTCAGATTTCTGGGCTGAATGTGCTGCGAGTCATCAATGAGCCCACTGCTGCAGCACTGGCCTATGGCATGGACAAGACTGAAGACAAGATTATTGGAGTGTATGATCTTGGTGGTGGAACCTTTGATATTTCCATTCTTGAGATCCAGAAGGGAGTGTTTGAGGTGAAGTCTACCAATGGTGATACATTCCTGGGAGGTGAGGACTTTGACAGGCATCTTGTCACCTTCCTTGCTGGTGAATTCAAGAAGGAGCAAGGTGTGGATGTGACCAAGGACACTATGGCCATGCAGAGGCTGAAGGAAGCTGCTGAGAAGGCCAAGATTGAACTGTCCTCCTCCAACCAGACTGACATTAACCTCCCCTACCTGACCATGGATGCATCTGGACCCAAGCACATGATGTATAAGCTGACAAGAGCCAAGTTTGAAAGCATTGTGGACAAGCTGATCAAACGCACCGTTGACCCATGCCTCAAGGCGATCAAGGATGCAGAGTGCAACAAGTCAGAGATAGGCGAGGTGATCCTGGTTGGTGGAATGTCTCGTATGCCCAAGGTCATCTCAATGGTGCAGGAAATATTTGGCCGTGCTCCAAGCAAGGCGGTGAACCCCGATGAGGCAGTGGCTGTGGGCGCTGCCATCCAGGGCGGTGTATTGGCAGGCGATGTGACAGATGTGTTGCTGCTGGATGTCACGCCGCTGTCACTTGGTATTGAGACCCTGGGAGGTGTGATGACTAAGCTCATCAACAGAAACACGACCATTCCCACCAGGAAGTCACAAGTGTTCTCCACAGCTGCTGATGGCCAGACACAGGTAGAGATTAAGGTTCACCAGGGAGAGAGAGAAATGGCTGGTGATAACAAGCTTCTGGGACAGTTCAGCTTGGTTGGCATTCCTCCTGCTCCTCGTGGAGTCCCACAGATTGAAGTCACCTTTGACATTGATGCCAACGGCATTGTTCATGTCTCTGCTCGGGACAAGGGCACCGGCAAGGAACAGCAGATTGTGATCCAGTCTTCTGGAGGTCTTAGCAAAGATGAGATTGAGAACATGATCAAGAAAGCAGAACAGTATGCTGAGGAGGATAAGAAGAAGAAGGAAGTGGTGGAGGCCATCAACCAGGGAGAGAGTATCATTCATGACACTGAAAGCAAAATGGAGGAGTTCAAAGACCAGCTTCCTGCAGAAGAGAGTGACAAGTTAAAGGAAAGGCTTACAAAGGTGAAGGATCTTCTGGCGAACAAGGACTCTGCCGATCCAGAAGAAATCAAGAAGGAAGTCGGTGAACTCCAGCAGGCATCACTGAAGCTGTTTGAGATGGCATACAAGAAGATGGCTTCAGAAAGAGAAAGCAGTGGATCATCAGGTACTGAAGACAAGGATAAAGAGAAGAAGGAAGAAAGTCAGCATTAA

MMSKARCLATNLGALSARKNVAQLLQQSSVQGVLAQHNALNMQTRYKSDGVKGAVIGIDLGTTNSCVAVMEGKTAKVITNAEGDRTTPSVIAFTKDGERLVGIQAKRQAVTNAGNTFYATKRLIGRRYDDEEIVKDRETVPFKIVKASNGDAWVQTDDGKMYSPSQVGAFVLTKMKETGEAYLNTPVKNAVITVPAYFNDSQRQATKDAGQISGLNVLRVINEPTAAALAYGMDKTEDKIIGVYDLGGGTFDISILEIQKGVFEVKSTNGDTFLGGEDFDRHLVTFLAGEFKKEQGVDVTKDTMAMQRLKEAAEKAKIELSSSNQTDINLPYLTMDASGPKHMMYKLTRAKFESIVDKLIKRTVDPCLKAIKDAECNKSEIGEVILVGGMSRMPKVISMVQEIFGRAPSKAVNPDEAVAVGAAIQGGVLAGDVTDVLLLDVTPLSLGIETLGGVMTKLINRNTTIPTRKSQVFSTAADGQTQVEIKVHQGEREMAGDNKLLGQFSLVGIPPAPRGVPQIEVTFDIDANGIVHVSARDKGTGKEQQIVIQSSGGLSKDEIENMIKKAEQYAEEDKKKKEVVEAINQGESIIHDTESKMEEFKDQLPAEESDKLKERLTKVKDLLANKDSADPEEIKKEVGELQQASLKLFEMAYKKMASERESSGSSGTEDKDKEKKEESQH

transcript_transcript/12947_Pt_Mix_transcript12947/f2p0/2448

ATGTCTAAGGGAGCAGCAGTGGGTATTGACTTGGGGACAACCTACTCCTGCGTAGGTGTCTTCCAGCATGGCAAGGTGGAGATCATCGCTAACGATCAGGGCAACAGAACTACGCCCTCTTACGTGGCCTTCACCGACACTGAGCGTCTCATCGGAGATGCTGCCAAGAACCAGGTGGCCATGAATCCAAACAACACTGTTTTTGATGCCAAGAGGCTGATTGGCAGGAAGTTCACTGATCATCATGTACAGTCAGACATGAAGCACTGGCCCTTCGAGGTCATTGAGGACAGCACGAAACCAAAGATCAGGGTAGAGTACAAAGGAGAAAAGAAGTCTTTCTACCCTGAGGAGATCTCCTCAATGGTGCTCATGAAGATGAAGGAAACAGCAGAAGCATACCTTGGTGCTGCCGTGAAGGATGCTGTTATCACTGTCCCAGCGTACTTTAATGACTCCCAGCGTCAGGCCACCAAAGACGCAGGCACCATCTCTGGTGTCAATGTGCTGCGTATCATTAATGAACCCACCGCTGCTGCCATCGCCTATGGTTTGGACAAGAAAGTAGGTGGTGAGCGCAATGTCCTCATCTTCGATCTTGGCGGTGGGACCTTTGATGTATCTATCCTGACCATCGAGGATGGCATCTTTGAGGTGAAGTCAACTGCAGGAGACACACACTTGGGTGGAGAAGACTTTGACAACAGAATGGTAAACCATTTCCTTCAGGAATTCAAGCGAAAGTACAAGAAGGACCCAACTGAGAGCAAGCGAGCCCTGAGGCGCCTGCGTACTGCCTGTGAGCGTGCAAAGCGTACCCTCTCCTCCTCCACCCAGGCCAGTGTGGAAATAGATTCTCTCTTTGAGGGCATCGACTTCTACACTTCTGTTACCCGTGCCCGCTTTGAGGAACTATGTGCTGACCTGTTCCGTGGCACACTGGAGCCCGTGGAGAAGGCTCTACGTGATGCCAAGCTGGACAAGGCCCAGATTCATGACATTGTTCTTGTGGGAGGCTCCACCCGTATCCCTAAGATCCAGAAACTCCTGCAGGACTTCTTCAATGGAAAGGAGCTGAACAAGTCCATAAACCCTGATGAAGCTGTGGCCTATGGTGCAGCTGTGCAGGCTGCCATCCTTTGTGGTGATAAGTCTGAGGCTGTCCAGGACTTGCTGCTGTTGGATGTGACTCCTCTCTCACTGGGTATTGAAACTGCAGGTGGTGTGATGACTGCCCTCATCAAACGTAACACCACCATCCCCACCAAGCAAACCCAGACCTTCACCACTTATTCTGATAACCAGCCTGGTGTGCTCATCCAGGTGTACGAGGGAGAGCGAGCTATGACTAAGGATAACAACCTCCTTGGGAAGTTTGAGTTGACTGGCATTCCCCCAGCTCCACGAGGAGTTCCTCAGATTGAGGTGACCTTTGACATTGATGCCAATGGTATTCTCAATGTGTCTGCAGTAGACAAATCCACTGGAAAGGAGAACAAGATCACCATCACTAATGACAAGGGTCGCCTGAGCAAGGAGGAAATTGAACGCATGGTGCAAGATGCAGAGAAGTACAAGGCAGAGGATGATAAGCAGAGGGACCGCATTGGTGCCAAGAATGCCTTGGAGTCCTACTGCTTCAACATGAAGTCCACTGTGGAAGAAGAGAAGTTCAAGGACAAAGTGAGTGAAGAGGATCGTAACAAGATCCTGGAGGCTTGCAATGAGGCTATCAAATGGCTTGATGCCAACCAGCTGGGAGAGAAAGACGAGTATGAACACAAGCAGAAGGAACTGGAGCAGATCTGCAACCCTATCATCACCAAGATGTACCAAGCTGCTGGTGGTGCTCCCCCAGGTGGCATGCCTGGTGGCTTCCCTGGTGCTGGTGGTGCTCCAGGTGGTGCCCCTGGCGGTGGCTCCTCTGGTCCTACCATCGAGGAGGTTGATTAA

MSKGAAVGIDLGTTYSCVGVFQHGKVEIIANDQGNRTTPSYVAFTDTERLIGDAAKNQVAMNPNNTVFDAKRLIGRKFTDHHVQSDMKHWPFEVIEDSTKPKIRVEYKGEKKSFYPEEISSMVLMKMKETAEAYLGAAVKDAVITVPAYFNDSQRQATKDAGTISGVNVLRIINEPTAAAIAYGLDKKVGGERNVLIFDLGGGTFDVSILTIEDGIFEVKSTAGDTHLGGEDFDNRMVNHFLQEFKRKYKKDPTESKRALRRLRTACERAKRTLSSSTQASVEIDSLFEGIDFYTSVTRARFEELCADLFRGTLEPVEKALRDAKLDKAQIHDIVLVGGSTRIPKIQKLLQDFFNGKELNKSINPDEAVAYGAAVQAAILCGDKSEAVQDLLLLDVTPLSLGIETAGGVMTALIKRNTTIPTKQTQTFTTYSDNQPGVLIQVYEGERAMTKDNNLLGKFELTGIPPAPRGVPQIEVTFDIDANGILNVSAVDKSTGKENKITITNDKGRLSKEEIERMVQDAEKYKAEDDKQRDRIGAKNALESYCFNMKSTVEEEKFKDKVSEEDRNKILEACNEAIKWLDANQLGEKDEYEHKQKELEQICNPIITKMYQAAGGAPPGGMPGGFPGAGGAPGGAPGGGSSGPTIEEVD

transcript_transcript/14389_Pt_Mix_transcript14389/f5p0/2319

ATGAGGTGTTGGGTAGCCCTAGGCCTGGTGGCCACCGTGGCCGTATTGGTCACAGCCAAGGAGTCGAAGAAGGAAGAGGTGGGAACTGTCATCGGTATTGACCTTGGCACCACTTACTCATGTGTGGGTGTGTTCAAAAATGGGAGAGTTGAGATCATTGCTAATGACCAGGGCAACAGGATCACACCCTCCTACGTAGCATTCACTGCTGATGGGGAGCGACTCATTGGTGACTCTGCCAAGAACCAGCTCACCACCAACCCTGAGAATACCATCTTTGATGCCAAGCGGCTCATTGGCAGGGAATGGACCGACAAATCTGTCCAGCATGACATCCAGTTCTTCCCCTTCAAGGTCATCAAGAAGAACGAAAAGCCCCACATTCAGGTTTCCACTTCACAGGGAGAGAAGGTGTTTGCTGCAGAAGAGGTCTCTGCCATGGTCCTTGGAAAGATGAAGGAAGTTGCTGAGGCTTATCTCGGAAAGACAGTCACTCATGCTGTCGTCACTGTCCCTGCCTACTTCAACGATGCCCAGCGACAGGCAACAAAGGATGCCGGCACAATTGCTGGATTGACAGTCATGAGGATCATCAATGAGCCTACAGCTGCTGCCATTGCCTATGGTATTGACAAGAAGGAAGGAGAGAAGAACATTCTTGTGTTTGATCTCGGTGGTGGCACCTTTGACGTCTCCCTGCTCACTATTGATTCCGGCGTGTTTGAAGTGGTGGCCACAAATGGCGACACTCATCTTGGTGGTGAGGACTTTGACCAGCGTGTCATGGACCACTTCATCAAGCTGTACAAGAAGAAGAAAGGCAAGGACATCAGAAAGGACAACCGTGCTGTTCAGAAGCTCCGTCGTGAGGTTGAGAAGGCAAAGAGGTCCCTGTCTGCTAGCCACCAGGTCAGGATTGAAATTGAATCCTTCTTTGAAGGAGAGGACTTCTCAGAGACACTCACCCGTGCCAAGTTTGAGGAGCTGAACATGGATCTCTTCAGGTCTACCATGAAGCCAGTGCAGAAGGTGCTTGAGGACTCTGACCTGCAGAAGAAGGAAATCGACGAGATCGTGTTGGTGGGCGGCTCCACTCGTATTCCCAAGATCCAGCAGCTGGTGAAGGAATTCTTCAATGGCAAGGAGCCATCCCGAGGTATCAATCCTGATGAGGCTGTTGCGTATGGTGCTGCTGTCCAGGCTGGTGTGCTGTCTGGTGAGGACGACACCAATGACCTTGTGCTGCTTGATGTCAACCCTCTGACTCTTGGAATTGAGACTGTGGGTGGAGTCATGACCAAGCTCATTTCCCGTAACACTGTCATCCCCACCAAGAAGTCCCAGATCTTCTCCACTGCCTCTGACAACCAGCACACTGTCACTATCCAGGTATTCGAGGGTGAGCGACCCATGACCAAGGATAACCACATCCTTGGAAAGTTTGACCTGACCGGCATCCCCCCAGCTCCTCGTGGTGTGCCTCAGATTGAAGTGACCTTTGAGATCGATGCTAATGGTATCCTCCAGGTATCTGCTGAAGACAAGGGCACTGGCAATAAGGAAAAGATTGTCATCACCAACGACCAGAACCGCCTCACTCCAGAGGATATCGAACGCATGATCAAGGATGCTGAGGTGTTTGCTGACGAGGACAAGAAGCTGAAGGAGCGTGTTGAGTCCAGGAATGAGCTGGAGTCGTATGCCTACAGCCTGAAGAACCAGGTGAATGACAAGGAAAAGCTTGGAGCCAAGCTTACCGATGAGGACAAGGAAAAGATCGAGGAAGCCATTGATGAGAAGATCAAGTGGCTGGAGGACAACCCTGATTCTGAGGCTGAAGATTATAAGGCTCAGAAGAAGGAACTGGAGGACATTGTGCAGCCGATCATTGCCAAGCTGTACCAGGGTGCTGGTGGCACTCCTCCCACTGGTGAAGAGGAGTTTGATAAGGACGAATTGTAA

MRCWVALGLVATVAVLVTAKESKKEEVGTVIGIDLGTTYSCVGVFKNGRVEIIANDQGNRITPSYVAFTADGERLIGDSAKNQLTTNPENTIFDAKRLIGREWTDKSVQHDIQFFPFKVIKKNEKPHIQVSTSQGEKVFAAEEVSAMVLGKMKEVAEAYLGKTVTHAVVTVPAYFNDAQRQATKDAGTIAGLTVMRIINEPTAAAIAYGIDKKEGEKNILVFDLGGGTFDVSLLTIDSGVFEVVATNGDTHLGGEDFDQRVMDHFIKLYKKKKGKDIRKDNRAVQKLRREVEKAKRSLSASHQVRIEIESFFEGEDFSETLTRAKFEELNMDLFRSTMKPVQKVLEDSDLQKKEIDEIVLVGGSTRIPKIQQLVKEFFNGKEPSRGINPDEAVAYGAAVQAGVLSGEDDTNDLVLLDVNPLTLGIETVGGVMTKLISRNTVIPTKKSQIFSTASDNQHTVTIQVFEGERPMTKDNHILGKFDLTGIPPAPRGVPQIEVTFEIDANGILQVSAEDKGTGNKEKIVITNDQNRLTPEDIERMIKDAEVFADEDKKLKERVESRNELESYAYSLKNQVNDKEKLGAKLTDEDKEKIEEAIDEKIKWLEDNPDSEAEDYKAQKKELEDIVQPIIAKLYQGAGGTPPTGEEEFDKDEL

transcript_transcript/15607_Pt_Mix_transcript15607/f3p0/2226

ATGGCCATAGCACGTGCAGTAGGCATCGATCTGGGCACCACCTACTCCTGCGTAGGGGTGTTCCAGCATGGCAAGGTGGAGATCATCGCCAATGACCAGGGCAACCGGACCACTCCCTCCTACGTGGCCTTCACAGACACCGAGCGGCTGATTGGAGACGCCGCGAAGAACCAGGTGGCCATGAACCCCAATAACACAGTCTTTGATGCCAAGCGACTCATCGGCAGGAAGTTTGAGGATGCCACAGTACAGAGCGACATGAAGCAGTGGCCCTTCACTGTGATCTCAGATGGAGGCAAGCCAAAGATCAGCGTGGAATACAAAGGAGAGGCCAAGAAGTTCTTCCCCGAGGAGATCTCTTCCATGGTCCTCATCAAGATGAAGGAGACTGCCGAAGCTTACTTGGGCACTACCGTCAAGGATGCCGTGGTCACTGTCCCCGCTTACTTCAACGACTCCCAGCGCCAGGCCACCAAGGACGCCGGCACCATCTCAGGAATGAACGTTCTCAGGATTATCAACGAGCCCACAGCGGCTGCCATCGCCTACGGCCTCGACAAGAGGGTTGGAGGCGAACGCAATGTCCTCATCTTTGACTTGGGTGGTGGAACTTTCGATGTGTCCATTCTTACCATCGAGGACGGAATCTTCGAGGTGAAGTCCACTGCTGGGGACACGCATCTTGGTGGCGAAGACTTCGATAACAGAATGGTCAACTTCTTCATCCAGGAGTTTAAGAGGAAGTACAAGAAGGATCTCTCTACTAATAAGCGTGCCATCCGCCGCCTGAGGACAGCATGTGAACGCGCCAAGAGAACCTTATCCTCCTCCACCCAGGCCAGCATTGAGATCGACTCTTTGTTTGAGGGCATTGATTTCTACACATCTGTCACACGTGCTCGCTTTGAAGAACTGTGCTCTGATCTGTTCCGTGGCACGCTGGAGCCCGTGGAGAAGTCTCTGCGTGACGCCAAGCTGGATAAGGCTCAGATCCATGACATCGTGCTCGTCGGTGGATCCACCAGGATTCCCAAGATTCAGAAACTGCTGCAAGACTTCTTCAATGGCAAGGAATTGAACAAGTCTATCAACCCCGACGAGGCAGTAGCATATGGCGCAGCAGTGCAGGCAGCCATCCTTTGTGGTGACAAGTCTGAGGAGGTCCAGGACCTGCTGCTGCTGGACGTGACGCCACTGTCCCTCGGCATCGAGACAGCAGGCGGCGTGATGACGGCACTCATCAAGCGTAACACCACCATCCCCACCAAGCAAACTCAGACCTTCACCACTTACTCAGACAACCAGCCCGGTGTGCTAATCCAGGTCTATGAAGGCGAACGAGCCATGACCCGCGACAACAACCTGCTGGGCAAGTTCGAGCTGACAGGCATCCCTCCCGCGCCTCGCGGCGTGCCTCAGATCGAAGTGACCTTCGACATCGATGCTAACGGAATCCTGAATGTGTCTGCCGTGGACAAGTCCACAGGCAAGGAGAACAAGATCACCATCACCAATGACAAGGGTCGCCTCAGCAAGGAGGAGATCGAGCGTATGGTGAACGAGGCAGATAAATATCGCGCCGAGGATGAGCAGCAACGTGAGCGAATCAGCGCCAAGAACAACCTGGAGTCGTACTGCTTCAACATGAAGTCCACAGTGGACGACGAGAAGTTCAAGGACAAGATCCCCGAGAGTGACCGCACCGCCATCTTGGACAAGTGCAACGAGACCATCCAGTGGCTGGACGCCAACCAGCTGGCAGAGAAGGACGAGTACGAGCACCGCCAGAAGGAACTAGAGAAGGTATGCAACCCCATCATCACCAAGATGTATGCGGCGGCGGGGGGCGCACCAGGCGGTATGCCCGGCGGTATGCCCGGTGGGATGCCCGGTGGCCCTCAGGGCGGCAGCACCGGCGGCGGCAGCTCCGGACCAACCATTGAGGAGGTGGATTAA

MAIARAVGIDLGTTYSCVGVFQHGKVEIIANDQGNRTTPSYVAFTDTERLIGDAAKNQVAMNPNNTVFDAKRLIGRKFEDATVQSDMKQWPFTVISDGGKPKISVEYKGEAKKFFPEEISSMVLIKMKETAEAYLGTTVKDAVVTVPAYFNDSQRQATKDAGTISGMNVLRIINEPTAAAIAYGLDKRVGGERNVLIFDLGGGTFDVSILTIEDGIFEVKSTAGDTHLGGEDFDNRMVNFFIQEFKRKYKKDLSTNKRAIRRLRTACERAKRTLSSSTQASIEIDSLFEGIDFYTSVTRARFEELCSDLFRGTLEPVEKSLRDAKLDKAQIHDIVLVGGSTRIPKIQKLLQDFFNGKELNKSINPDEAVAYGAAVQAAILCGDKSEEVQDLLLLDVTPLSLGIETAGGVMTALIKRNTTIPTKQTQTFTTYSDNQPGVLIQVYEGERAMTRDNNLLGKFELTGIPPAPRGVPQIEVTFDIDANGILNVSAVDKSTGKENKITITNDKGRLSKEEIERMVNEADKYRAEDEQQRERISAKNNLESYCFNMKSTVDDEKFKDKIPESDRTAILDKCNETIQWLDANQLAEKDEYEHRQKELEKVCNPIITKMYAAAGGAPGGMPGGMPGGMPGGPQGGSTGGGSSGPTIEEVD

transcript_transcript/16659_Pt_Mix_transcript16659/f4p0/2135

ATGTCTAAGGGAGCAGCAGTGGGTATTGACTTGGGGACAACCTACTCCTGCGTAGGTGTCTTCCAGCATGGCAAGGTGGAGATCATCGCTAACGATCAGGGCAACAGAACTACGCCCTCTTACGTGGCCTTCACCGACACTGAGCGTCTCATCGGAGATGCTGCCAAGAACCAGGTGGCCATGAATCCAAACAACACTGTTTTTGATGCCAAGAGGCTGATTGGCAGGAAGTTCACTGATCATCATGTACAGTCAGACATGAAGCACTGGCCCTTCGAGGTCATTGAGGACAGCACGAAACCAAAGATCAGGGTAGAGTACAAAGGAGAAAAGAAGTCTTTCTACCCTGAGGAGATCTCCTCAATGGTGCTCATGAAGATGAAGGAAACAGCAGAAGCATACCTTGGTGCTGCCGTGAAGGATGCTGTTATCACTGTCCCAGCGTACTTTAATGACTCCCAGCGTCAGGCCACCAAAGACGCAGGCACCATCTCTGGTGTCAATGTGCTGCGTATCATTAATGAACCCACCGCTGCTGCCATCGCCTATGGTTTGGACAAGAAAGTAGGTGGTGAGCGCAATGTCCTCACCATCGAGGATGGCATCTTTGAGGTGAAATCAACTGCAGGAGACACACACTTGGGTGGAGAAGACTTTGACAACAGAATGGTAAACCATTTCCTTCAGGAATTCAAGCGAAAGTACAAGAAGGACCCAACTGAGAGCAAGCGAGCCCTGAGGCGCCTGCGTACTGCCTGTGAGCGTGCAAAGCGTACCCTCTCCTCCTCCACCCAGGCCAGTGTGGAAATAGATTCTCTCTTTGAGGGCATCGACTTCTACACTTCTGTTACCCGTGCCCGCTTTGAGGAACTATGTGCTGACCTGTTCCGTGGCACACTGGAGCCCGTGGAGAAGGCTCTACGTGATGCCAAGCTGGACAAGGCCCAGATTCATGACATTGTTCTTGTGGGAGGCTCCACCCGTATCCCTAAGATCCAGAAACTCCTGCAGGACTTCTTCAATGGAAAGGAGCTGAACAAGTCCATAAACCCTGATGAAGCTGTGGCCTATGGTGCAGCTGTGCAGGCTGCCATCCTTTGTGGTGATAAGTCTGAGGCTGTCCAGGACTTGCTGCTGTTGGATGTGACTCCTCTCTCACTGGGTATTGAAACTGCAGGTGGTGTGATGACTGCCCTCATCAAACGTAACACCACCATCCCCACCAAGCAAACCCAGACCTTCACCACTTATTCTGATAACCAGCCTGGTGTGCTCATCCAGGTGTACGAGGGAGAGCGAGCTATGACTAAGGATAACAACCTCCTTGGGAAGTTTGAGTTGACTGGCATTCCCCCAGCTCCACGAGGAGTTCCTCAGATTGAGGTGACCTTTGACATTGATGCCAATGGTATCCTCAATGTGTCTGCAGTAGACAAATCCACTGGAAAGGAGAACAAGATCACCATCACTAATGACAAGGGTCGCCTGAGCAAGGAGGAAATTGAACGCATGGTGCAAGATGCAGAGAAGTACAAGGCAGAGGATGATAAGCAGAGGGACCGCATTGGTGCCAAGAATGCCTTGGAGTCCTACTGCTTCAACATGAAGTCCACTGTGGAAGAAGAGAAGTTCAAGGACAAAGTGAGTGAAGAGGATCGTAACAAGATCCTAGAGGCTTGCAATGAGGCTATCAAATGGCTTGATGCCAACCAGCTGGGAGAGAAAGACGAGTATGAACACAAGCAGAAGGAACTGGAGCAGATCTGCAACCCTATCATCACCAAGATGTACCAAGCTGCTGGTGGTGCTCCCCCAGGTGGCATGCCTGGTGGCTTCCCTGGTGCTGGTGGTGCTCCAGGTGGTGCCCCTGGCGGTGGCTCCTCTGGTCCTACCATCGAGGAGGTTGATTAA

MSKGAAVGIDLGTTYSCVGVFQHGKVEIIANDQGNRTTPSYVAFTDTERLIGDAAKNQVAMNPNNTVFDAKRLIGRKFTDHHVQSDMKHWPFEVIEDSTKPKIRVEYKGEKKSFYPEEISSMVLMKMKETAEAYLGAAVKDAVITVPAYFNDSQRQATKDAGTISGVNVLRIINEPTAAAIAYGLDKKVGGERNVLTIEDGIFEVKSTAGDTHLGGEDFDNRMVNHFLQEFKRKYKKDPTESKRALRRLRTACERAKRTLSSSTQASVEIDSLFEGIDFYTSVTRARFEELCADLFRGTLEPVEKALRDAKLDKAQIHDIVLVGGSTRIPKIQKLLQDFFNGKELNKSINPDEAVAYGAAVQAAILCGDKSEAVQDLLLLDVTPLSLGIETAGGVMTALIKRNTTIPTKQTQTFTTYSDNQPGVLIQVYEGERAMTKDNNLLGKFELTGIPPAPRGVPQIEVTFDIDANGILNVSAVDKSTGKENKITITNDKGRLSKEEIERMVQDAEKYKAEDDKQRDRIGAKNALESYCFNMKSTVEEEKFKDKVSEEDRNKILEACNEAIKWLDANQLGEKDEYEHKQKELEQICNPIITKMYQAAGGAPPGGMPGGFPGAGGAPGGAPGGGSSGPTIEEVD

transcript_transcript/17243_Pt_Mix_transcript17243/f3p0/2121

ATGTCTAAGGGAGCAGCAGTGGGTATTGACTTGGGGACAACCTACTCCTGCGTAGGTGTCTTCCAGCATGGCAAGGTGGAGATCATCGCTAACGATCAGGGCAACAGAACTACGCCCTCTTACGTGGCCTTCACCGACACTGAGCGTCTCATCGGAGATGCTGCCAAGAACCAGGTGGCCATGAATCCAAACAACACTGTTTTTGATGCCAAGAGGCTGATTGGCAGGAAGTTCACTGATCATCATGTACAGTCAGACATGAAGCACTGGCCCTTCGAGGTCATTGAGGACAGCACGAAACCAAAGATCAGGGTAGAGTACAAAGGAGAAAAGAAGTCTTTCTACCCTGAGGAGATCTCCTCAATGGTGCTCATGAAGATGAAGGAAACAGCAGAAGCATACCTTGGTGCTGCCGTGAAGGATGCTGTTATCACTGTCCCAGCGTACTTTAATGACTCCCAGCGTCAGGCCACCAAAGACGCAGGCACCATCTCTGGTGTCAATGTGCTGCGTATCATTAATGAACCCACCGCTGCTGCCATCGCCTATGGTTTGGACAAGAAAGTAGGTGGTGAGCGCAATGTCCTCATCTTCGATCTTGGCGGTGGGACCTTTGATGTATCTATCCTGACCATCGAGGATGGCATCTTTGAGGTGAAATCAACTGCAGGAGACACACACTTGGGTGGAGAAGACTTTGACAACAGAATGGTAAACCATTTCCTTCAGGAATTCAAGCGAAAGTACAAGAAGGACCCAACTGAGAGCAAGCGAGCCCTGAGGCGCCTGCGTACTGCCTGTGAGCGTGCAAAGCGTACCCTCTCCTCCTCCACCCAGGCCAGTGTGGAAATAGATTCTCTCTTTGAGGGCATCGACTTCTACACTTCTGTTACCCGTGCCCGCTTTGAGGAACTATGTGCTGACCTGTTCCGTGGCACACTGGAGCCCGTGGAGAAGGCTCTACGTGATGCCAAGCTGGACAAGGCCCAGATTCATGACATTGTTCTTGTGGGAGGCTCCACCCGTATCCCTAAGATCCAGAAACTCCTGCAGGACTTCTTCAATGGAAAGGAGCTGAACAAGTCCATAAACCCTGATGAAGCTGTGGCCTATGGTGCAGCTGTGCAGGCTGCCATCCTTTGTGGTGATAAGTCTGAGGCTGTCCAGGACTTGCTGCTGTTGGATGTGACTCCTCTCTCACTGGGTATTGAAACTGCAGGTGGTGTGATGACTGCCCTCATCAAACGTAACACCACCATCCCCACCAAGCAAACCCAGACCTTCACCACTTATTCTGATAACCAGCCTGGTGTGCTCATCCAGGTGTACGAGGGAGAGCGAGCTATGACTAAGGATAACAACCTCCTTGGGAAGTTTGAGTTGACTGGCATTCCCCCAGCTCCACGAGGAGTTCCTCAGATTGAGGTGACCTTTGACATTGATGCCAATGGTATCCTCAATGTGTCTGCAGTAGACAAATCCACTGGAAAGGAGAACAAGATCACCATCACTAATGACAAGGGTCGCCTGAGCAAGGAGGAAATTGAACGCATGGTGCAAGATGCAGAGAAGTACAAGGCAGAGGATGATAAGCAGAGGGACCGCATTGGTGCCAAGAATGCCTTGGAGTCCTACTGCTTCAACATGAAGTCCACTGTGGAAGAAGAGAAGTTCAAGGACAAAGTGAGTGAAGAGGATCGTAACAAGATCCTAGAGGCTTGCAATGAGGCTATCAAATGGCTTGATGCCAACCAGCTGGGAGAGAAAGACGAGTATGAACACAAGCAGAAGGAACTGGAGCAGATCTGCAACCCTATCATCACCAAGATGTACCAAGCTGCTGGTGGTGCCCCCTGGCGGTGGCTCCTCTGGTCCTACCATCGAGGAGGTTGA

MSKGAAVGIDLGTTYSCVGVFQHGKVEIIANDQGNRTTPSYVAFTDTERLIGDAAKNQVAMNPNNTVFDAKRLIGRKFTDHHVQSDMKHWPFEVIEDSTKPKIRVEYKGEKKSFYPEEISSMVLMKMKETAEAYLGAAVKDAVITVPAYFNDSQRQATKDAGTISGVNVLRIINEPTAAAIAYGLDKKVGGERNVLIFDLGGGTFDVSILTIEDGIFEVKSTAGDTHLGGEDFDNRMVNHFLQEFKRKYKKDPTESKRALRRLRTACERAKRTLSSSTQASVEIDSLFEGIDFYTSVTRARFEELCADLFRGTLEPVEKALRDAKLDKAQIHDIVLVGGSTRIPKIQKLLQDFFNGKELNKSINPDEAVAYGAAVQAAILCGDKSEAVQDLLLLDVTPLSLGIETAGGVMTALIKRNTTIPTKQTQTFTTYSDNQPGVLIQVYEGERAMTKDNNLLGKFELTGIPPAPRGVPQIEVTFDIDANGILNVSAVDKSTGKENKITITNDKGRLSKEEIERMVQDAEKYKAEDDKQRDRIGAKNALESYCFNMKSTVEEEKFKDKVSEEDRNKILEACNEAIKWLDANQLGEKDEYEHKQKELEQICNPIITKMYQAAGGAPWRWLLWSYHRGG

transcript_transcript/7098_Pt_Mix_transcript7098/f56p0/3046

ATGTTGCAAATAAGATACCGGTACTGCTTCCTGCTGCTCGCCCTGCTGTTCACCGCAGGCGGCGTGAGGGCAGCGGAGGACGACGATGATGTGGCAGGGGAGGGTACGGTGGAGAGTGACCTGGGCGCTGGAAGGGAAGGCTCTCGGACTGACGACGGGGTGGTGGCCAGGGAGGAGGAGGCAATCAAGCTGGACGGACTGAATGTGGCCCAGATGAAAGAGCTGCGGGAGAAGGCGGAGAAACACGCTTTCCAGGCCGAGGTTAACCGCATGATGAAGTTGATCATTAACTCGCTCTACAGGAACAAGGAGATCTTCCTGAGAGAGTTGATCAGTAACGCCTCGGATGCCCTGGACAAGATCCGCCTGCTGTCCCTCACTGACAAGGAGCAACTCGACTCCAACCCAGAAATGGTCATCAGGATCAAGGCAGACAAGGACAACCACATCCTTCACATCACTGACACGGGTATTGGCATGACCCGCAATGATCTGGTGAACAACCTGGGCACCATCGCCAAGTCTGGCACCTCTGAGTTCTTCTCCAAGCTGCAGGATTCTGACAGCCCTGACCAGGCCAATGACCTGATTGGTCAGTTCGGCGTGGGCTTCTACTCTGCCTTCTTGGTGGCAGACCGTGTGGTGGTGACGTCCAAGAACAACGCAGACCAGCAGCACATCTGGGAGTCTGATTCTGCTGAGTTCTCTGTGGTGGAGGACCCTCGTGGGGACACCCTGAAGAGAGGAACGACGGTGTCTCTGCATCTGAAGGAGGAAGCGTACGACTTTGTGGAGGTGGACACTGTGAAGACTTTGGTCAAGAAATACTCACAGTTCATCAACTTCCCCATCTACCTGTGGGAGTCCAAGACTGAAGAGGTGGAGGAGCCTCTGGATGAGGATGAGGTGGAAGAAGAAAAGGAGGAGGACAAGGTTGAGGAAGACGAGGAAGGAAAGGTAGAGGAGGAAAAAGAGGACAAACCCAAGACAAAGAAGGTTTCCAAGACAACATGGGACTGGACTCTGGTGAATGATGCCAAGCCCATCTGGACCAGGAAGCCAGCGGAGATTGAGGACGAAGAGTACAACGAATTCTACAAGACGCTGAGCAAGGACTCCAAGGATCCGTTGGCCAAGACTCACTTCATTGCCGAGGGTGAGGTGACCTTCAAGTCCCTGTTGTTTGTCCCAGAAGTGCAGCCCAGCGAGTCCTTCAACAAGTACGGCACACGCACCGACAACATCAAGCTGTATGTGCGTCGTGTCTTCATCACCGACGACTTCCAGGACATGATGCCAAACTACCTCAACTTTGTGCGGGGCGTGGTGGACTCTGACGACCTGCCCCTCAACGTTTCCCGAGAGAACCTGCAGCAGCACAAGCTCCTGAAGGTGATCAAGAAGAAGCTGGTGCGCAAGACTCTGGACATGATCAAGAAGCTGGAGCCTGAGGAGTATGAGAAGTTCTGGAAGGAGTACTCCACCAACCTCAAGCTTGGCACCATTGAAGACTCGGCCAACCGTACTCGCCTTGCCAAGCTGCTACGCTTCCTCTCCTCCTCCTCTGGGGACAAGATGATCTCTCTGTCTGAGTATGTGGAGCGCATGAAGGAGAAGCAGGAACACATCTACTACATGGCTGGCTCTTCCAAGGCCGAGGTTGAGAACTCTCCCTTCGTGGAGCGTCTGCTGAAGAAGGGATATGAGGTGCTATTCCTGACGGAGGCCATCGATGAATACGCTGTCAATGCCATCCCAGAGTTTGAGGGCAAGAAGTTCCAGAATGTGGCCAAGGAAGGACTGACCATTGACGAGGGTGAAGGAGCCAAGGAGCGCCTGGAGGAGCTGAAGAAGGTGTTTGAACCTCTCACCAAGTGGCTCAGTGAAGACGCACTCAAGGATGAGGTCTCCAAGGCTGTTGTTTCTGAGAGGCTCTCGGATTCTCCCTGTGCCCTGGTGGCCAGCATGTTTGGGTGGACAGGAAACATGGAACGCTTGGCTATCTCTAATGCTCACCAAAAGACCCACGACTCTCATAGGGACTACTACCTGAGCCAGAAGAAGACCCTGGAGATCAACCCTCGCCATCCTCTTGTCAAGGAATTGCTGCGCCGTGTGGAGTCTGACCCTGCTGATGAGAAAGCCAAGAACATTGCTGAGATGATGTTCCACACAGCCACCCTGCGCTCTGGCTACATGCTGAGGAACACTGTTGATTTCTCAAAGTCTGTGGAGGAGATGATGCGACAGACACTGGGCATTGCTGTGGACGAGCCAGTGGAAGAGGAGCCAGAGTATGAAGAGGCAGATGAGGATGAGGAGCAGCTGCTGGAGGAGGATGAAGATGAGGATGGAGGTGAGGTGGAAGAAATGCACGACGAATTGTAA

MLQIRYRYCFLLLALLFTAGGVRAAEDDDDVAGEGTVESDLGAGREGSRTDDGVVAREEEAIKLDGLNVAQMKELREKAEKHAFQAEVNRMMKLIINSLYRNKEIFLRELISNASDALDKIRLLSLTDKEQLDSNPEMVIRIKADKDNHILHITDTGIGMTRNDLVNNLGTIAKSGTSEFFSKLQDSDSPDQANDLIGQFGVGFYSAFLVADRVVVTSKNNADQQHIWESDSAEFSVVEDPRGDTLKRGTTVSLHLKEEAYDFVEVDTVKTLVKKYSQFINFPIYLWESKTEEVEEPLDEDEVEEEKEEDKVEEDEEGKVEEEKEDKPKTKKVSKTTWDWTLVNDAKPIWTRKPAEIEDEEYNEFYKTLSKDSKDPLAKTHFIAEGEVTFKSLLFVPEVQPSESFNKYGTRTDNIKLYVRRVFITDDFQDMMPNYLNFVRGVVDSDDLPLNVSRENLQQHKLLKVIKKKLVRKTLDMIKKLEPEEYEKFWKEYSTNLKLGTIEDSANRTRLAKLLRFLSSSSGDKMISLSEYVERMKEKQEHIYYMAGSSKAEVENSPFVERLLKKGYEVLFLTEAIDEYAVNAIPEFEGKKFQNVAKEGLTIDEGEGAKERLEELKKVFEPLTKWLSEDALKDEVSKAVVSERLSDSPCALVASMFGWTGNMERLAISNAHQKTHDSHRDYYLSQKKTLEINPRHPLVKELLRRVESDPADEKAKNIAEMMFHTATLRSGYMLRNTVDFSKSVEEMMRQTLGIAVDEPVEEEPEYEEADEDEEQLLEEDEDEDGGEVEEMHDEL

transcript_transcript/9552_Pt_Mix_transcript9552/f318p0/2655

ATGCCTGAGGATGCTGCCATGGAAGATGTGGAGACCTTCGCCTTCCAGGCGGAGATCGCCCAGCTTATGTCCCTCATCATCAACACTTTCTACAGCAACAAAGAAATCTTCCTGCGAGAGATAATCTCCAACAGCTCTGATGCCCTGGACAAGATCCGATACGAGTCCCTCACTGACCCCTCAAAGCTGGAGAGTGGCAAGGAGCTTTTCATCAAGCTCATCCCAGACAAGAATGACCGCACCCTCACCATCATTGACAGTGGTATTGGCATGACCAAGGCTGACCTGGTGAACAACTTGGGTACCATCGCCAAGTCTGGCACCAAGGCTTTCATGGAGGCACTGCAGGCCGGTGCCGACATCTCCATGATTGGTCAGTTCGGCGTGGGCTTCTACTCGGCCTACCTGGTGGCTGACAAGGTCACAGTGGTGTCAAAGAACAACGATGATGAGCAGTACGTGTGGGAGTCCTCCGCTGGCGGGTCCTTCACTGTACGCACTGACCATGGTGAGCCACTGGGCCGAGGTACCAGGATCACCCTTCACCTGAAGGAAGACCAAACAGAGTACCTTGAGGAGCGCCGTATCAGGGAGATTGTCAAGAAGCACTCTCAGTTCATTGGCTACCCCATCAGGCTCCTTGTTGAGAAGGAGAGGGATAAGGAAGTGTCTGATGATGAGGAGGAGGAAAAGGAAGAAGAGAAGGAGAAGAAAGAAGAGGAAGATGATGACAAGCCAAAGATTGAGGATGTAGGTGAAGATGAAGATGCAGATAAGAAGGATGGTGACAAGAAGAAAAAGAAGACTGTGAAGGAGAAGTACACTGAGGATGAGGAGCTGAACAAGACCAAGCCCCTCTGGACCCGCAACCCTGATGACATCTCCCAGGAGGAGTATGGAGAGTTCTACAAATCCCTTACCAATGACTGGGAGGATCATCTAGCAGTCAAGCACTTCAGCGTTGAGGGACAGCTGGAGTTCAGGGCTCTGCTGTTCCTGCCTCGCCGTGCTCCCTTTGACCTCTTTGAAAACCGCAAGCAAAAGAACAAGATCAAGCTGTACGTGCGTCGTGTCTTCATCATGGAGAACTGTGAGGAACTGATTCCTGAATACCTTAACTTCCTCAATGGTGTTGTGGATTCAGAGGATCTTCCTCTCAACATCTCCCGAGAAATGTTGCAGCAGAACAAGATCCTTAAGGTCATCCGCAAGAATTTGGTAAAGAAGGCCATGGAGCTTTTCGAGGAGCTGGTGGAGGACAAGGATAACTACAAGAAGTTCTACGAGAACTTCTCAAAGAACATCAAGCTGGGTATCCACGAAGACTCCACCAACCGCAAGAAACTGGCCGAGTTCCTGCGGTACCACACCTCTGCCTCTGGTGATGAGATGTCCTCCCTCAAGGACTATGTGTCCCGTATGAAGGAGAACCAGAAGCAGATCTACTACATCACTGGTGAGAGCCGTGAACAGGTTCACAACTCGGCCTTCGTGGAGAGAGTGAAGAAGCGTGGCTTCGAGGTGGTGTACATGGTTGAACCCATTGATGAGTACTGTGTCCAGCAGCTGAAGGAATATGACGGCAAGCAGCTGGTGTCCGTCACCAAGGAGGGTCTGGAACTCCCTGAGGATGAGGACGAGAAGAAGAAATTGGAAGAACAGAAGACCAAGTTCGAGAACTTGTGCAAGGTTGTCAAGGACATCCTAGACAAGCGTGTTGAGAAGGTGGTGGTGAGCAACAGGCTGGTGACCTCGCCATGCTGCATTGTCACCTCCCAGTACGGCTGGACCGCAAACATGGAGCGCATCATGAAGGCGCAGGCCCTCAGGGACACCTCCACCATGGGATATATGGCTGCCAAGAAGCACCTTGAGATCAACCCAGATCACAGCATCATCGAGACTCTTCGCCAGAAGGCTGATGCTGACAAGAATGACAAGTCTGTCAAGGATCTTGTCATGTTGCTCTTTGAGAGTGCCCTTCTATCTTCTGGCTTCACCCTGGAGGACCCTGGTGTCCATGCTGGCCGTATCTACAGAATGATCAAGCTTGGCCTTGGTATCGATGAGGATGACGCCCCTGCCGAGGACAACACCGAGAGTGTGGAGGAAATGCCACCCCTGGAGGATGAAGAGGACACTTCCCGCATGGAGGAAGTGGACTAA

MPEDAAMEDVETFAFQAEIAQLMSLIINTFYSNKEIFLREIISNSSDALDKIRYESLTDPSKLESGKELFIKLIPDKNDRTLTIIDSGIGMTKADLVNNLGTIAKSGTKAFMEALQAGADISMIGQFGVGFYSAYLVADKVTVVSKNNDDEQYVWESSAGGSFTVRTDHGEPLGRGTRITLHLKEDQTEYLEERRIREIVKKHSQFIGYPIRLLVEKERDKEVSDDEEEEKEEEKEKKEEEDDDKPKIEDVGEDEDADKKDGDKKKKKTVKEKYTEDEELNKTKPLWTRNPDDISQEEYGEFYKSLTNDWEDHLAVKHFSVEGQLEFRALLFLPRRAPFDLFENRKQKNKIKLYVRRVFIMENCEELIPEYLNFLNGVVDSEDLPLNISREMLQQNKILKVIRKNLVKKAMELFEELVEDKDNYKKFYENFSKNIKLGIHEDSTNRKKLAEFLRYHTSASGDEMSSLKDYVSRMKENQKQIYYITGESREQVHNSAFVERVKKRGFEVVYMVEPIDEYCVQQLKEYDGKQLVSVTKEGLELPEDEDEKKKLEEQKTKFENLCKVVKDILDKRVEKVVVSNRLVTSPCCIVTSQYGWTANMERIMKAQALRDTSTMGYMAAKKHLEINPDHSIIETLRQKADADKNDKSVKDLVMLLFESALLSSGFTLEDPGVHAGRIYRMIKLGLGIDEDDAPAEDNTESVEEMPPLEDEEDTSRMEEVD

transcript_transcript/19331_Pt_Mix_transcript19331/f3p0/1962

ATGTTGAAGCTCCTCTTGATGGTAGTGGCGGCAGTGGCGGTGTCATCGTGGGTCGGCGGCGGCGAGGTGATGGTGGCGGCCCAGTCTGGTGGTGGTGCCCGCGTCGTTTTCACCGATGAACTGCCGATGCCCGAGCTGATCTCCAAGACGCGCCCTCCCTGCGTGGACTCCAATAATATCGGGGGGGACCTGTGCGAAGAAGACCCAGAGTACGATGACAACATCAAGCGGCGCGTCTCCATGCTCATGGAAAGGGACAGAGGAGTGAACGCCTTGTTGAGCGACCCTTCTCTCAGAGGCCTTCTTGTTGACGTGGATCCGGAGCCGAGGGTGAACGTTCGGTTCGGCGGGGACTCAGAGACACCTGTATGTTCCGCCCAGGAGACACTCATCTACCCAAAACGCGCCAAGACACCCAATGATGAGTGGGTGTTTGTATTGAACCAGGAAGGCGTGCAGCAGGCCTTGAGGGTGGAGAAGTGCACCAGCGATGGCTCGGCGTGCTTGGGGATGGTGCTGCCTAACGGGGGCACAGCCACCTGCCGCCAGAAGCATATCTATCGCAGACTGCTGGTCCTCGGCACCAACAAAATTGAGCCAGAAGAGGTGTTGATGCCCTCCTGCTGCGTCTGTTACACCACATATCAAGACTTGGTGACTCGTATAGGAAACAGCACAAGGGGCCGCAAGCCCCCTTCTTCCACCCCGCCCCGTGACGATCCTGTCGCTCCATCAATGCCCACCTCGTCTGATCCTGTTGCTGCACCAGTGGCCGGCCATGCTCACTTCCTTCCGCTGAGATTCAACAGATCAAACAGGAGGTCTGCAAGTGTTTTGTACCCATTCGGCGGCAAACGCTAA

MLKLLLMVVAAVAVSSWVGGGEVMVAAQSGGGARVVFTDELPMPELISKTRPPCVDSNNIGGDLCEEDPEYDDNIKRRVSMLMERDRGVNALLSDPSLRGLLVDVDPEPRVNVRFGGDSETPVCSAQETLIYPKRAKTPNDEWVFVLNQEGVQQALRVEKCTSDGSACLGMVLPNGGTATCRQKHIYRRLLVLGTNKIEPEEVLMPSCCVCYTTYQDLVTRIGNSTRGRKPPSSTPPRDDPVAPSMPTSSDPVAAPVAGHAHFLPLRFNRSNRRSASVLYPFGGKR

transcript_transcript/19430_Pt_Mix_transcript19430/f2p0/1941

ATGTTGAAGCTCCTCTTGATGGTAGTGGCGGCAGTGGCGGTGTCATCGTGGGTCGGCGGCGGCGAGGTGATGGTGGCGGCCCAGTCTGGTGGTGGTGCCCGCGTCGTTTTCACCGATGAAGTGAGTTGGCGTGGCTACATGCGAGGCCTTGGTCACATGCGAGGCCTTGGTCACATGCGAGGCCTTGGCTACATGAGGGCCGCGCAGTCTTCCGAAGAGGATATTCGAAGAATCAACTATCTGCCGATGCCCGAGCTGATCTCCAAGACGCGCCCTCCCTGCGTGGACTCCAATAATATCGGGGGGGACCTGTGCGAAGAAGACCCAGAGTACGATGACAACATCAAGCGGCGCGTCTCCATGCTCATGGAAAGGGACAGAGGAGTGAACGCCTTGTTGAGCGACCCTTCTCTCAGAGGCCTTCTTGTTGACGTGGATCCGGAGCCGAGGGTGAACGTTCGGTTCGGCGGGGACTCAGAGACACCTGTATGTTCCGCCCAGGAGACACTCATCTACCCAAAACGCGCCAAGACACCCAATGATGAGTGGGTGTTTGTATTGAACCAGGAAGGCGTGCAGCAGGCCTTGAGGGTGGAGAAGTGCACCAGCGATGGCTCGGCGTGCTTGGGGATGGTGCTGCCTAACGGGGGCACAGCCACCTGCCGCCAGAAGCATATCTATCGCAGACTGCTGGTCCTCGGCACCAACAAAATTGAGCCAGAAGAGGTGTTGATGCCCTCCTGCTGCGTCTGTTACACCACATATCAAGACTTGGTGACTCGTATAGGAAACAGCACAAGGGGCCGCAAGCCCCCTTCTTCCACCCCGCCCCGTGACGATCCTGTCGCTCCATCAATGCCCACCTCGTCTGATCCTGTTGCTGCACCAGTGGCCGGCCATGCTCACTTCCTTCCGCTGAGATTCAACAGATCAAACAGGAGGTCTGCAAGTGTTTTGTACCCATTCGGCGGCAAACGCTAA

MLKLLLMVVAAVAVSSWVGGGEVMVAAQSGGGARVVFTDEVSWRGYMRGLGHMRGLGHMRGLGYMRAAQSSEEDIRRINYLPMPELISKTRPPCVDSNNIGGDLCEEDPEYDDNIKRRVSMLMERDRGVNALLSDPSLRGLLVDVDPEPRVNVRFGGDSETPVCSAQETLIYPKRAKTPNDEWVFVLNQEGVQQALRVEKCTSDGSACLGMVLPNGGTATCRQKHIYRRLLVLGTNKIEPEEVLMPSCCVCYTTYQDLVTRIGNSTRGRKPPSSTPPRDDPVAPSMPTSSDPVAAPVAGHAHFLPLRFNRSNRRSASVLYPFGGKR

transcript_transcript/19587_Pt_Mix_transcript19587/f36p0/1919

ATGTTGAAGCTCCTCTTGATGGTAGTGGCGGCAGTGGCGGTGTCATCGTGGGTCGGCGGCGGCGAGGTGATGGTGGCGGCCCAGTCTGGTGGTGGTGCCCGCGTCGTTTTCACCGATGAACTGCCGATGCCCGAGCTGATCTCCAAGACGCGCCCTCCCTGCGTGGACTCCAATAATATCGGGGGGGACCTGTGCGAAGAAGACCCAGAGTACGATGACAACATCAAGCGGCGCGTCTCCATGCTCATGGAAAGGGACAGAGGAGTGAACGCCTTGTTGAGCGACCCTTCTCTCAGAGGCCTTCTTGTTGACGGACGACCAACCCTTCAAGAACTCAACAGATCACGCAGGGACGCCACAGAACTCATGACTTTTGATCTTTCTAAGATTTCTGACTCCGACAGAGAAAACTTAGTGGATCCGGAGCCGAGGGTGAACGTTCGGTTCGGCGGGGACTCAGAGACACCTGTATGTTCCGCCCAGGAGACACTCATCTACCCAAAACGCGCCAAGACACCCAATGATGAGTGGGTGTTTGTATTGAACCAGGAAGGCGTGCAGCAGGCCTTGAGGGTGGAGAAGTGCACCAGCGATGGCTCGGCGTGCTTGGGGATGGTGCTGCCTAACGGGGGCACAGCCACCTGCCGCCAGAAGCATATCTATCGCAGACTGCTGGTCCTCGGCACCAACAAAATTGAGCCAGAAGAGGTGTTGATGCCCTCCTGCTGCGTCTGTTACACCACATATCAAGACTTGGTGACTCGTATAGGAAACAGCACAAGGGGCCGCAAGCCCCCTTCTTCCACCCCGCCCCGTGACGATCCTGTCGCTCCATCAATGCCCACCTCGTCTGATCCTGTTGCTGCACCAGTGGCCGGCCATGCTCACTTCCTTCCGCTGAGATTCAACAGATCAAACAGGAGGTCTGCAAGTGTTTTGTACCCATTCGGCGGCAAACGCTAA

MLKLLLMVVAAVAVSSWVGGGEVMVAAQSGGGARVVFTDELPMPELISKTRPPCVDSNNIGGDLCEEDPEYDDNIKRRVSMLMERDRGVNALLSDPSLRGLLVDGRPTLQELNRSRRDATELMTFDLSKISDSDRENLVDPEPRVNVRFGGDSETPVCSAQETLIYPKRAKTPNDEWVFVLNQEGVQQALRVEKCTSDGSACLGMVLPNGGTATCRQKHIYRRLLVLGTNKIEPEEVLMPSCCVCYTTYQDLVTRIGNSTRGRKPPSSTPPRDDPVAPSMPTSSDPVAAPVAGHAHFLPLRFNRSNRRSASVLYPFGGKR

transcript_transcript/22268_Pt_Mix_transcript22268/f2p0/1752

ATGCCCGAGCTGATCTCCAAGACGCGCCCTCCCTGCGTGGACTCCAATAATATCGGGGGGGACCTGTGCGAAGAAGACCCAGAGTACGATGACAACATCAAGCGGCGCGTCTCCATGCTCATGGAAAGGGACAGAGGAGTGAACGCCTTGTTGAGCGACCCTTCTCTCAGAGGCCTTCTTGTTGACGTGGATCCGGAGCCGAGGGTGAACGTTCGGTTCGGCGGGGACTCAGAGACACCTGTATGTTCCGCCCAGGAGACACTCATCTACCCAAAACGCGCCAAGACACCCAATGATGAGTGGGTGTTTGTATTGAACCAGGAAGGCGTGCAGCAGGCCTTGAGGGTGGAGAAGTGCACCAGCGATGGCTCGGCGTGCTTGGGGATGGTGCTGCCTAACGGGGGCACAGCCACCTGCCGCCAGAAGCATATCTATCGCAGACTGCTGGTCCTCGGCACCAACAAAATTGAGCCAGAAGAGGTGTTGATGCCCTCCTGCTGCGTCTGTTACACCACATATCAAGACTTGGTGACTCGTATAGGAAACAGCACAAGGGGCCGCAAGCCCCCTTCTTCCACCCCGCCCCGTGACGATCCTGTCGCTCCATCAATGCCCACCTCGTCTGATCCTGTTGCTGCACCAGTGGCCGGCCATGCTCACTTCCTTCCGCTGAGATTCAACAGATCAAACAGGAGGTCTGCAAGTGTTTTGTACCCATTCGGCGGCAAACGCTAA

MPELISKTRPPCVDSNNIGGDLCEEDPEYDDNIKRRVSMLMERDRGVNALLSDPSLRGLLVDVDPEPRVNVRFGGDSETPVCSAQETLIYPKRAKTPNDEWVFVLNQEGVQQALRVEKCTSDGSACLGMVLPNGGTATCRQKHIYRRLLVLGTNKIEPEEVLMPSCCVCYTTYQDLVTRIGNSTRGRKPPSSTPPRDDPVAPSMPTSSDPVAAPVAGHAHFLPLRFNRSNRRSASVLYPFGGKR

transcript_transcript/22874_Pt_Mix_transcript22874/f2p0/1726

ATGTTGAAGCTCCTCTTGATGGTAGTGGCGGCAGTGGCGGTGTCATCGTGGGTCGGCGGCGGCGAGGTGATGGTGGCGGCCCAGTCTGGTGGTGGTGCCCGCGTCGTTTTCACCGATGAACTGCCGATGCCCGAGCTGATCTCCATGCTCATGGAAAGGGACAGAGGAGTGAACGCCTTGTTGAGCGACCCTTCTCTCAGAGGCCTTCTTGTTGACGTGGATCCGGAGCCGAGGGTGAACGTTCGGTTCGGCGGGGACTCAGAGACACCTGTATGTTCCGCCCAGGAGACACTCATCTACCCAAAACGCGCCAAGACACCCAATGATGAGTGGGTGTTTGTATTGAACCAGGAAGGCGTGCAGCAGGCCTTGAGGGTGGAGAAGTGCACCAGCGATGGCTCGGCGTGCTTGGGGATGGTGCTGCCTAACGGGGGCACAGCCACCTGCCGCCAGAAGCATATCTATCGCAGACTGCTGGTCCTCGGCACCAACAAAATTGAGCCAGAAGAGGTGTTGATGCCCTCCTGCTGCGTCTGTTACACCACATATCAAGACTTGGTGACTCGTATAGGAAACAGCACAAGGGGCCGCAAGCCCCCTTCTTCCACCCCGCCCCGTGACGATCCTGTCGCTCCATCAATGCCCACCTCGTCTGATCCTGTTGCTGCACCAGTGGCCGGCCATGCTCACTTCCTTCCGCTGAGATTCAACAGATCAAACAGGAGGTCTGCAAGTGTTTTGTACCCATTCGGCGGCAAACGCTAA

MLKLLLMVVAAVAVSSWVGGGEVMVAAQSGGGARVVFTDELPMPELISMLMERDRGVNALLSDPSLRGLLVDVDPEPRVNVRFGGDSETPVCSAQETLIYPKRAKTPNDEWVFVLNQEGVQQALRVEKCTSDGSACLGMVLPNGGTATCRQKHIYRRLLVLGTNKIEPEEVLMPSCCVCYTTYQDLVTRIGNSTRGRKPPSSTPPRDDPVAPSMPTSSDPVAAPVAGHAHFLPLRFNRSNRRSASVLYPFGGKR

transcript_transcript/26209_Pt_Mix_transcript26209/f2p0/1610

ATGCTCATGGAAAGGGACAGAGGAGTGAACGCCTTGTTGAGCGACCCTTCTCTCAGAGGCCTTCTTGTTGACGTGGATCCGGAGCCGAGGGTGAACGTTCGGTTCGGCGGGGACTCAGAGACACCTGTATGTTCCGCCCAGGAGACACTCATCTACCCAAAACGCGCTAAGACACCCAATGATGAGTGGGTGTTTGTATTGAACCAGGAAGGCGTGCAGCAGGCCTTGAGGGTGGAGAAGTGCACCAGCGATGGCTCGGCGTGCTTGGGGATGGTGCTGCCTAACGGGGGCACAGCCACCTGCCGCCAGAAGCATATCTATCGCAGACTGCTGGTCCTCGGCACCAACAAAATTGAGCCAGAAGAGGTGTTGATGCCCTCCTGCTGCGTCTGTTACACCACATATCAAGACTTGGTGACTCGTATAGGAAACAGCACAAGGGGCCGCAAGCCCCCTTCTTCCACCCCGCCCCGTGACGATCCTGTCGCTCCATCAATGCCCACCTCGTCTGATCCTGTTGCTGCACCAGTGGCCGGCCATGCTCACTTCCTTCCGCTGAGATTCAACAGATCAAACAGGAGGTCTGCAAGTGTTTTGTACCCATTCGGCGGCAAACGCTAA

MLMERDRGVNALLSDPSLRGLLVDVDPEPRVNVRFGGDSETPVCSAQETLIYPKRAKTPNDEWVFVLNQEGVQQALRVEKCTSDGSACLGMVLPNGGTATCRQKHIYRRLLVLGTNKIEPEEVLMPSCCVCYTTYQDLVTRIGNSTRGRKPPSSTPPRDDPVAPSMPTSSDPVAAPVAGHAHFLPLRFNRSNRRSASVLYPFGGKR

transcript_transcript/29178_Pt_Mix_transcript29178/f2p0/1315

ATGCCCTCCTGCTGCGTCTGTTACACCACATATCAAGACTTGGTGACTCGTATAGGAAACAGCACAAGGGGCCGCAAGCCCCCTTCTTCCACCCCGCCCCGTGACGATCCTGTCGCTCCATCAATGCCCACCTCGTCTGATCCTGTTGCTGCACCAGTGGCCGGCCATGCTCACTTCCTTCCGCTGAGATTCAACAGATCAAACAGGAGGCCTTCTCTCAGAGGCCTTCTTGTTGACGTGGATCCGGAGCCGAGGGTGAACGTTCGGTTCGGCGTGGACTCAGAGACACCTGTATGTTCCGCCCAGGAGACACTCATCTACCCAAAACGCGCCAAGACACCCAATGATGAGTGGGTGTTTGTATTGAACCAGGAAGGCGTGCAGCAGGCCTTGAGGGTGGAGAAGTGTCCGCCATTAGTGTTTCCAGGAGCGTCACTCTATCCACTGGGCCATGGTGTGAGTGTATGTGTGTGTATGGTATGCTAG

MPSCCVCYTTYQDLVTRIGNSTRGRKPPSSTPPRDDPVAPSMPTSSDPVAAPVAGHAHFLPLRFNRSNRRPSLRGLLVDVDPEPRVNVRFGVDSETPVCSAQETLIYPKRAKTPNDEWVFVLNQEGVQQALRVEKCPPLVFPGASLYPLGHGVSVCVCMVC

transcript_transcript/32420_Pt_Mix_transcript32420/f7p0/1039

ATGGTTTTCTCACTGTCGGTATTTCTGGCGTGTGCCGGAGTGGCTCTCGGCTCGCTGCCCCCAGTGCACAGGACACCTACCAGGCAGCCCTACGGTGCCCACCCCACCCACAGGGCTGGCTACTGCGACCCCACCACGCCTCCTACCTGTGCCTATGGTAGCGACGTCTCCTTCTGCCTGGAAGACCCCGAGTACCCCGAGTACGACATCAAGGGCGCCATCGAGGCCGACAAGCTCTTCGCCAAGAAGTATGCTGACGTGGCCGACCAATCCGCCGACGACCTGGTTGAACACGTGACCAAGTACCAGGAGGAGGCGTTCGACTACTCCTACTACACCGGCGCCTCTACCGGCTACTCCCCCTACGACGTGACCCACTGGACGGGCCCCGAGGGCTACATCTGTCCCTCGGACGTGGCCTACGCCCGCCCCAGACGCGCCCGCAACGTGGAGGGCAAGTGGAGGGTGATCGTGAACGACGTGCACTACTATACCCAGACGGCACGCCTGGAGACGTGCCTGTTCCCCGAGGCCGCCTGCCGCGCCTTGGCGCCATGCTTCAAGAGTCACTGCACCCAAAAGTATGTGTACCACCGCCTGCTCTCCTACGACCCCTGCGATCCCTACAAGGGCCTCTTCATCGACATCTACAAGCTGCCCTCCGCCTGCTCCTGCCACGTCGCCCCTCCCCCTCCTTCTTACGCCTAA

MVFSLSVFLACAGVALGSLPPVHRTPTRQPYGAHPTHRAGYCDPTTPPTCAYGSDVSFCLEDPEYPEYDIKGAIEADKLFAKKYADVADQSADDLVEHVTKYQEEAFDYSYYTGASTGYSPYDVTHWTGPEGYICPSDVAYARPRRARNVEGKWRVIVNDVHYYTQTARLETCLFPEAACRALAPCFKSHCTQKYVYHRLLSYDPCDPYKGLFIDIYKLPSACSCHVAPPPPSYA

transcript_transcript/346_Pt_Mix_transcript346/f2p0/5853

ATGGCGAGGTGGTGTCCCGCAAGGCGTCTCCTGGGACTGACCGTGTTTGTGGCGGTGGTGGCTTGGTGTTCAGGAAATTACATTATCACCACGCCGAGGAAGTGGGCAGAAGCGAGCGCCGCCCAAGTGTGCCTGTTCAACGTGGGCGGAGGCGTGGAGGAGGTGGAGGCGCCCGTTGAGGGAGTGGAGGTGAAGGTGGAAGGCGAGCACAGGAGTGCGGATGTGCTGGTTCCTCCTCAGTTTGTCTCCATTCCTCCGGGCCAGACGGAGGTGTGTCGGTCCATCCCAGTACCGGCGGTGGATCACATGAGGGGCAAACTCCACATCAACGGGACACTGGGAGGCAAGTCAGTCACCCACACTGAAACCATCAAGTTCGCCGCAGCCTCTCAGACATTCGTACAGACAGACAAGTTCCTTTACGCCCCGGGACAGAAGGTGCAGTTCAGGATCCTCACCGTTTACGGGCCTTACCTTAGAGTTTTCACAGGCATGTACCCGGAGATGTGGATCGAGTCCCCGTCCGGCAGCCGCATCGCCCAGTGGGTCAGAGTGGAGAGCTCCGGGGGTCTGATCCACCAGGAGTTCCAACTGATTAACGAGCCCGAGGAGGGGACGTATAAGATCCATGTGGAGTCGCCAGTGGGAGGATCCAAGGCAGTGCAGACATTCAAGATAGAGGATTTTGTGTTGCCGCGTTTTGAAGTCACCCTGCAGTCACCACCCTACATTCTGGCTACTACCAAGAGTCTGCACTACCGAGTTTGTGCTATGTACACGTATGGCCAGCCAGTAAAGGGAGAAGTGACCTTTGACATCCGTAAACCTGACCACTACAAAGTAACAAGACCGATCACCGGCTGTGAGAACTTCACCATTGCCATGAGCACCTCCCTAAGCACCAGTTATGGCCACTCCACCGTCAATGTGGTGGCCAAGGTGGTGGAGGAAGGCACGGGGGAGGAGGCGCAGGCTACAGGACAGATAGATGTACAGCGGAAGGTTCTTAAATTCAAGCATGTAGGGAAGGAGGAGTATGTCAAGCCTAATCTTCCCTACACTGGCCAGTTCAAGGTGACACTGCCCTCAGATGAACCAGCTGCCAACGAGCTGATGACAATCTGCAAAGGCGACACCTGCAGGAATATCACCACTGACGCTAGAGGGTTAGTGGAATACATCTACCCGAAGCATGAGGATTTCTCCATAGAGATCTCCTCACCAAGATACCCGCGAATTGAAAACCCCGAAAGGTCCTGGAGCCCAATTATGTACAAGTCAGCCGACAGTCACAGAGTCAAGACCTACTACTCCCCTTCAAACTCCTCCCTTGTGATCAAAACCCCGGAGGTCCAGCTGAGGTGCCACGCTGGGGAGACACAAAACTTCACGGTCTCTGTCATGTATGCTGGCACGAGGGACTCTAAGGCTAACTTCACCATACAGCTTGTCTCTAGAGGTCAAATCCAGTTCACTCACACTGAGGAACACACCCTTACTGACACCGACCTCCCCATTGACTCCTCCCTGCTGCTGGTGCCCCTGCCGCCCGCCACTGAGGGTGTAGCAAGGGGTGTGCTCAGCCTGCCCCTCACCATTCACCTCACAGCCTCCCCATCTGCTAAGGTGCTGGTCTGGTACACACAGCCAGACGGTGAGGTGGTGTCAGCAATGCAGGAGATCCGAATCAAGAAGTGCCTGACCAACTTGGTGAGTCTTGCCTGGTCCACTAATAAAGCTGAGCCTGGCGAGGAGGTCCACCTTGACCTTAATGCTGAACCAAACTCTCTCTGCAGTCTGGGTGTGGTGGACAAGAGTGTGGAGCTTCTGCAGTCAAATGAGGACCACCTAACACTGGAGGGTGTGTTTAAGGTGGTCAAGAAGGCAATAGTTGGTGACACAGAGAACTCACAGATTAACGATAATGAATACTGCCAAAAAAAGAGGGAGGAAGATGAAGCAGACACCACTGATCCTCTACGACCAGGAATGCCCATACCCTTTGCTGAGGAGGAGCCTGCTGGAGTGGTGAAGAGGTCCATTTGGCATCCCTACGGTCACTATACTGAGGGTGTGGATGCCATTAAAATGTTTGAGAAATCTGGACTATTTGTGTTTACGGATCTGAAGGTGGAGAACAGACCCTGCCACTTTCGTGCTTTGGTGGTTAGGGTTGCATATCGTGCTCCAGCAAGTGTTGCAGGAATGACCCTTTTGAGATCCTCCAGTGCTGGAGTGTCTCCCGATACTGACATCAGATCAATTTTCAAAACTGGACAGGAAAGAGAAGTGGCAATAGAACAAGCCTCCCTCACTACCTTAACCAAACAGTCTGAGGAGTCGACCATTCGCTCCCACTTCCCCGAGACCTGGCTGTGGTCGCTGTCTCTTCTGACAGCTGCTGGGAACAGCAGCCAGGCGCTCACCCTACCACACACCATCACTGAGTGGATTGGCAAGGCAGTGTGCGTCCACCCAGAGAAAGGTGTCGGCTTGTCATCAAAAGAGTCCATTACCACCTTCACCCCCTTCTTTGTTGACCTGACCCTGCCTCCCTCTGTGCAGCGCGGCGAGATCCTCCCTGTCAAGATATCAGTGTTTAACTATCTAGAAGGAGCACTGCCGGTCAAAGTAGTGTTATCCGAAAGCCCAGAATATGAGATCTTAGAGGACCCTCTGGCCACGACTGTCAAGGGTAGTGCTTCTTCCTGCATTCCATCAAAGGAGAAGGTTGTGTTCACCGTCAAGATCCGCCCCAATGCTCTAGGGGATGTGAATCTGAATGTGGAGGCTTTCGTGGATGAGTTATTCCCTGAGGATTGTGGATCTGAGTATGTCATCAGTAAGAGGGACCACATCATCAAGCCCATCCGTGTTGAGCTTGAAGGATTCCCACAGGAGAAGACCTGGACCAAGTATATGTGTACTGATGGTGTGGACAACGAGGAGCAACTAGTGTCCTGGCATCTGGAGGCACCATCAAACATTGTCCCTGACTCAACAAGGGGATGGATCACTGCTGTTGGAGACCTTCTGGGACCAACACTTGATAACCTTGGCTCGCTGGTGCGGATGCCATACGGGTGCGGAGAGCAGAACATGGTCAACTTTGCTCCGAATATCTTTGTCATGCAGTACTTAGAGGCGGCAGAGAAGACCACGGCAGACATCGCAACAAAGGCCATTGAGTTCATGAAGAGTGGATACCAGAGGGAGCTACGCTATCGCCACAAGGACGGATCATTCAGCGCCTTCGGTCCCAGTGATGAGTCAGGCTCTACTTGGCTAACTGCATTTGTACTAAAGTCCTTCGCCCAGGCCCAGCAGTTCATCCCGATTGACACGGGCGACATTGACATGAGTCGGGAGTGGCTCAAGAGAGACCAGATGGAGAATGGCTGCTTCCTCTCCAAGGGCAAAGTCTTCCATAAGTCCATGAAGGGAGGCATAGCGGGTAATGAGTCACCAGTACCCCTCACAGCCTACATCCTCACTGCACTGCTGGAGGCTGGAGAGCTTATTGTGAGTCGGCCGATCAGTGAGGCTGCATTCTGTCTGGTCTCCGATAAGAGTGAGGACCCCTACACACTGGCACTCAAGGCTTATGCTCTAGCTCTGGCTGAAGCACCCGAAGCAGCCCAATTCGTCGTTCAACTCAGGGGCAAGGCAACTGTGTCCACAGAAGGCATGTACTGGGAAATGCCTCCAGTTAATGGGAAGAGTGCGGCAGCGGGGGTGGAGACGGCAGCCTATGCCCTCCTAGCTATGGCCACCCTGAGCCCGGTAGACTACCTTAATGACATGCAGAAGTTGGTCAAGTGGATCTCCAGTAAGAGGAACGGACAGGGCGGCTTTGTCTCCACTCAGGACACAGTGGTAGCCCTACAAGCCCTGGCCAAGTTTGAGATGGTGCTGGGCCAGAAACCAGTGGACGTGGCGGTACTTGCGTCCTCCACCAGCCTGGATCACTCCTTCCGCATCACCGAAACCAACAGACTCCTCCTGCAGCGCGTCGACCTGCCCTCCTTCCCCACAACTGTCACCGCTGACCTGGCTGGGGAAGGGTGTGCGTTGGTGCAGGCTGTCCTCCGCTACAACATTCCTGAGGAGGATCCCAGCACGGCCTTCAACCTCACCGCCACCACACAGACGGTCAGAGATGACAAATGCATCACCAAACGCATCAGAGCCTGCGCCTCCTACACTCTGCCGGACCTCAAATCTAACATGGCTGTCATCGAGATTAACCTGGTGTCTGGCTACATACCAGACAAGAATGATCTGAAGCAGGTGGTTGGCTACGGGACGGGTCTGATCAAGAGGTACGAGGTGGACGGCAGGAAGGTCACATTCTATATTGATGAGTTCTCCCCTGAGGATCTCTGTGTGGCCTTCAAGGTGACTCGCGAGGTGGATGTGGAGAACCCAAAGCCAGGAACGGTCAGGGTGTACGACTACTACGACCCGGACCAGTTTGTTAGTACGAGCTACACCTTCCCGCCCAATGAGGAGTGTGCGTCGGGTTCTGATTTGGACGGGGTGGATATCTTGGTGATCCCCAGTGATGACATTGCCGTTGACTACATCGATTATGTTGTCTAA

MARWCPARRLLGLTVFVAVVAWCSGNYIITTPRKWAEASAAQVCLFNVGGGVEEVEAPVEGVEVKVEGEHRSADVLVPPQFVSIPPGQTEVCRSIPVPAVDHMRGKLHINGTLGGKSVTHTETIKFAAASQTFVQTDKFLYAPGQKVQFRILTVYGPYLRVFTGMYPEMWIESPSGSRIAQWVRVESSGGLIHQEFQLINEPEEGTYKIHVESPVGGSKAVQTFKIEDFVLPRFEVTLQSPPYILATTKSLHYRVCAMYTYGQPVKGEVTFDIRKPDHYKVTRPITGCENFTIAMSTSLSTSYGHSTVNVVAKVVEEGTGEEAQATGQIDVQRKVLKFKHVGKEEYVKPNLPYTGQFKVTLPSDEPAANELMTICKGDTCRNITTDARGLVEYIYPKHEDFSIEISSPRYPRIENPERSWSPIMYKSADSHRVKTYYSPSNSSLVIKTPEVQLRCHAGETQNFTVSVMYAGTRDSKANFTIQLVSRGQIQFTHTEEHTLTDTDLPIDSSLLLVPLPPATEGVARGVLSLPLTIHLTASPSAKVLVWYTQPDGEVVSAMQEIRIKKCLTNLVSLAWSTNKAEPGEEVHLDLNAEPNSLCSLGVVDKSVELLQSNEDHLTLEGVFKVVKKAIVGDTENSQINDNEYCQKKREEDEADTTDPLRPGMPIPFAEEEPAGVVKRSIWHPYGHYTEGVDAIKMFEKSGLFVFTDLKVENRPCHFRALVVRVAYRAPASVAGMTLLRSSSAGVSPDTDIRSIFKTGQEREVAIEQASLTTLTKQSEESTIRSHFPETWLWSLSLLTAAGNSSQALTLPHTITEWIGKAVCVHPEKGVGLSSKESITTFTPFFVDLTLPPSVQRGEILPVKISVFNYLEGALPVKVVLSESPEYEILEDPLATTVKGSASSCIPSKEKVVFTVKIRPNALGDVNLNVEAFVDELFPEDCGSEYVISKRDHIIKPIRVELEGFPQEKTWTKYMCTDGVDNEEQLVSWHLEAPSNIVPDSTRGWITAVGDLLGPTLDNLGSLVRMPYGCGEQNMVNFAPNIFVMQYLEAAEKTTADIATKAIEFMKSGYQRELRYRHKDGSFSAFGPSDESGSTWLTAFVLKSFAQAQQFIPIDTGDIDMSREWLKRDQMENGCFLSKGKVFHKSMKGGIAGNESPVPLTAYILTALLEAGELIVSRPISEAAFCLVSDKSEDPYTLALKAYALALAEAPEAAQFVVQLRGKATVSTEGMYWEMPPVNGKSAAAGVETAAYALLAMATLSPVDYLNDMQKLVKWISSKRNGQGGFVSTQDTVVALQALAKFEMVLGQKPVDVAVLASSTSLDHSFRITETNRLLLQRVDLPSFPTTVTADLAGEGCALVQAVLRYNIPEEDPSTAFNLTATTQTVRDDKCITKRIRACASYTLPDLKSNMAVIEINLVSGYIPDKNDLKQVVGYGTGLIKRYEVDGRKVTFYIDEFSPEDLCVAFKVTREVDVENPKPGTVRVYDYYDPDQFVSTSYTFPPNEECASGSDLDGVDILVIPSDDIAVDYIDYVV

transcript_transcript/348_Pt_Mix_transcript348/f26p0/5826

ATGGCGAGGTGGTGTCCCGCAAGGCGTCTCCTGGGACTGACCGTGTTTGTGGCGGTGGTGGCTTGGTGTTCAGGAAATTACATTATCACCACGCCGAGGAAGTGGGCAGAAGCGAGCGCCGCCCAAGTGTGCCTGTTCAACGTGGGCGGAGGCGTGGAGGAGGTGGAGGCGCCCGTTGAGGGAGTGGAGGTGAAGGTGGAAGGCGAGCACAGGAGTGCGGATGTGCTGGTTCCTCCTCAGTTTGTCTCCATTCCTCCGGGCCAGACGGAGGTGTGTCGGTCCATCCCAGTACCGGCGGTGGATCACATGAGGGGCAAACTCCACATCAACGGGACACTGGGAGGCAAGTCAGTCACCCACACTGAAACCATCAAGTTCGCCGCAGCCTCTCAGACATTCGTACAGACAGACAAGTTCCTTTACGCCCCGGGACAGAAGGTGCAGTTCAGGATCCTCACCGTTTACGGGCCTTACCTTAGAGTTTTCACAGGCATGTACCCGGAGATGTGGATCGAGTCCCCGTCCGGCAGCCGCATCGCCCAGTGGGTCAGAGTGGAGAGCCCCGGGGGTCTGATCCACCAGGAGTTCCAACTGATTAACGAGCCCGAGGAGGGGACGTATAAGATCCATGTGGAGTCGCCAGTGGGAGGATCCAAGGCGGTGCAGACATTCAAGATAGAGGATTTTGTGTTGCCGCGTTTTGAAGTCACCCTGCAGTCACCACCCTACATTCTGGCTACTACCAAGAGTCTGCACTACCGAGTTTGTGCTATGTACACGTATGGCCAGCCAGTAAAGGGAGAAGTGACCTTTGACATCCGTAAACCTGACCACTACAAAGTAACAAGACCGATCACCGGCTGTGAGAACTTCACCATTGCCATGAGCACCTCCCTAAGCACCAGTTATGGCCACTCCACCGTCAATGTGGTGGCCAAGGTGGTGGAGGAAGGCACGGGGGAGGAGGCGCAGGCTACAGGACAGATAGATGTACAGCGGAAGGTTCTTAAATTCAAGCATGTAGGGAAGGAGGAGTATGTCAAGCCTAATCTTCCCTACACTGGCCAGTTCAAGGTGACACTGCCCTCAGATGAACCAGCTGCCAACGAGCTGATGACAATCTGCAAAGGCGACACCTGCAGGAATATCACCACTGACGCTAGAGGGTTAGTGGAATACATCTACCCGAAGCATGAGGATTTCTCCATAGAGATCTCCTCACCAAGATACCCGCGAATTGAAAACCCCGAAAGGTCCTGGAGCCCAATTATGTACAAGTCAGCCGACAGTCACAGAGTCAAGACCTACTACTCCCCTTCAAACTCCTCCCTTGTGATCAAAACCCCGGAGGTCCAGCTGAGGTGCCACGCTGGGGAGACACAAAACTTCACGGTCTCTGTCATGTATGCTGGCACGAGGGACTCTAAGGCTAACTTCACCATACAGCTTGTCTCTAGAGGTCAAATCCAGTTCACTCACACTGAGGAACACACCCTTACTGACACCGACCTCCCCATTGACTCCTCCCTGCTGCTGGTGCCCCTGCCGCCCGCCACTGAGGGTGTAGCAAGGGGTGTGCTCAGCCTGCCCCTCACCATTCACCTCACAGCCTCCCCATCTGCTAAGGTGCTGGTCTGGTACACACGGCCAGACGGTGAGGTGGTGTCAGCAATGCAGGAGATCCGAATCAAGAAGTGCCTGACCAACTTGGTGAGTCTTGCCTGGTCCACTAATAAAGCTGAGCCTGGCGAGGAGGTCCACCTTGACCTTAATGCTGAACCAAACTCTCTCTGCAGTCTGGGTGTGGTGGACAAGAGTGTGGAGCTTCTGCAGTCAAATGAGGACCACCTAACACTGGAGGGTGTGTTTAAGGTGGTCAAGAAGGCAATAGTTGGTGACACAGAGAACTCACAGATTAACGATAATGAATACTGCCAAAAAAAGAGGGAGGAAGATGAAGCAGACACCACTGATCCTCTACGACCAGGAATGCCCATACCCTTTGCTGAGGAGGAGCCTGCTGGAGTGGTGAAGAGGTCCATTTGGCATCCCTACGGTCACTATACTGAGGGTGTGGATGCCATTAAAATGTTTGAGAAATCTGGACTATTTGTGTTTACGGATCTGAAGGTGGAGAACAGACCCTGCCACTTTCGTGTTTATCACTACTTAGAAGACCGCATAGTAAGTGATGACATGGCTCTCCTTGACTTGGCAATTCCAGTGTCAATTGTTGGGCCAGTATTTGCAGCACTACCACAACCAGCACCACCATCACCAGAAAGAAGAGGAGAGGGAGGAGAGGAGGGAGAGGCAGTACAGTTGCGTTCTTACTTCCCCGAGACTTGGCTCTGGGAACTCTCTTTCATTCCAGCTGCTGGGAACAGCAGCCAGGCGCTCACCCTACCACACACCATCACTGAGTGGATTGGCAAGGCAGTGTGCGTCCACCCAGAGAAAGGTGTCGGCTTGTCATCAAAAGAGTCCATTACCACCTTCACCCCCTTCTTTGTTGACCTGACCCTGCCTCCCTCTGTGCAGCGCGGCGAGATCCTCCCTGTCAAGATATCAGTGTTTAACTATCTAGAAGGAGCACTGCCGGTCAAAGTAGTGTTATCCGAAAGCCCAGAATATGAGATCTTAGAGGACCCTCTGGCCACGACTGTCAAGGGTAGTGCTTCTTCCTGCATTCCATCAAAGGAGAAGGTTGTGTTCACCGTCAAGATCCGCCCCAATGCTCTAGGGGATGTGAATCTGAATGTGGAGGCTTTCGTGGATGAGTTATTCCCTGAGGATTGTGGATCTGAGTATGTCATCAGTAAGAGGGACCACATCATCAAGCCCATCCGTGTTGAGCTTGAAGGATTTCCACAGGAGAAGACCTGGACCAAGTATATGTGTACTGATGGTGTGGACAACGAGGAGCAACTAGTGTCCTGGCATCTGGAGGCACCATCAAACATTGTCCCTGACTCAACAAGGGGATGGATCACTGCTGTTGGAGACCTTCTGGGACCAACACTTGATAACCTTGGCTCGCTGGTGCGGATGCCATACGGGTGCGGAGAGCAGAACATGGTCAACTTTGCTCCGAATATCTTTGTCATGCAGTACTTAGAGGCGGCAGAGAAGACCACGGCAGACATCGCAACAAAGGCCATTGAGTTCATGAAGAGTGGATACCAGAGGGAGCTACGCTATCGCCACAAGGACGGATCATTCAGCGCCTTCGGTCCCAGTGATGAGTCAGGCTCTACTTGGCTAACTGCATTTGTACTAAAGTCCTTCGCCCAGGCCCAGCAGTTCATCCCGATTGACACGGGCGACATTGACATGAGTCGGGAGTGGCTCAAGAGAGACCAGATGGAGAATGGCTGCTTCCTCTCCAAGGGCAAAGTCTTCCATAAGTCCATGAAGGGAGGCATAGCGGGTAATGAGTCACCAGTACCCCTCACAGCCTACATCCTCACTGCACTGCTGGAGGCTGGAGAGCTTATTGTGAGTCGGCCGATCAGTGAGGCTGCATTCTGTCTGGTCTCCGATAAGAGTGAGGACCCCTACACACTGGCACTCAAGGCTTATGCTCTAGCTCTGGCTGAAGCACCCGAAGCAGCCCAATTCGTCGTTCAACTCAGGGGCAAGGCAACTGTGTCCACAGAAGGCATGTACTGGGAAATGCCTCCAGTTAATGGGAAGAGTGCGGCAGCGGGGGTGGAGACGGCAGCCTATGCCCTCCTAGCTATGGCCACCCTGAGCCCGGTAGACTACCTTAATGACATGCAGAAGTTGGTCAAGTGGATCTCCAGTAAGAGGAACGGACAGGGCGGCTTTGTCTCCACTCAGGACACAGTGGTAGCCCTACAAGCCCTGGCCAAGTTTGAGATGGTGCTGGGCCAGAAACCAGTGGACGTGGCGGTACTTGCGTCCTCCACCAGCCTGGATCACTCCTTCCGCATCACCGAAACCAACAGACTCCTCCTGCAGCGCGTCGACCTGCCCTCCTTCCCCACAACTGTCACCGCTGACCTGGCTGGGGAAGGGTGTGCGTTGGTGCAGGCTGTCCTCCGCTACAACATTCCTGAGGAGGATCCCAGCACGGCCTTCAACCTCACCGCCACCACACAGACGGTCAGAGATGACAAATGCATCACCAAACGCATCAGAGCCTGCGCCTCCTACACTCTGCCGGACCTCAAATCTAACATGGCTGTCATCGAGATTAACCTGGTGTCTGGCTACATACCAGACAAGAATGATCTGAAGCAGGTGGTTGGCTACGGGACGGGTCTGATCAAGAGGTACGAGGTGGACGGCAGGAAGGTCACATTCTATATTGATGAGTTCTCCCCTGAGGATCTCTGTGTGGCCTTCAAGGTGACTCGCGAGGTGGATGTGGAGAACCCAAAGCCAGGAACGGTCAGGGTGTACGACTACTACGACCCGGACCAGTTTGTTAGTACGAGCTACACCTTCCCGCCCAATGAGGAGTGTGCGTCGGGTTCTGATTTGGACGGGGTGGATATCTTGGTGATCCCCAGTGATGACATTGCCGTTGACTACATCGATTATGTTGTCTAA

MARWCPARRLLGLTVFVAVVAWCSGNYIITTPRKWAEASAAQVCLFNVGGGVEEVEAPVEGVEVKVEGEHRSADVLVPPQFVSIPPGQTEVCRSIPVPAVDHMRGKLHINGTLGGKSVTHTETIKFAAASQTFVQTDKFLYAPGQKVQFRILTVYGPYLRVFTGMYPEMWIESPSGSRIAQWVRVESPGGLIHQEFQLINEPEEGTYKIHVESPVGGSKAVQTFKIEDFVLPRFEVTLQSPPYILATTKSLHYRVCAMYTYGQPVKGEVTFDIRKPDHYKVTRPITGCENFTIAMSTSLSTSYGHSTVNVVAKVVEEGTGEEAQATGQIDVQRKVLKFKHVGKEEYVKPNLPYTGQFKVTLPSDEPAANELMTICKGDTCRNITTDARGLVEYIYPKHEDFSIEISSPRYPRIENPERSWSPIMYKSADSHRVKTYYSPSNSSLVIKTPEVQLRCHAGETQNFTVSVMYAGTRDSKANFTIQLVSRGQIQFTHTEEHTLTDTDLPIDSSLLLVPLPPATEGVARGVLSLPLTIHLTASPSAKVLVWYTRPDGEVVSAMQEIRIKKCLTNLVSLAWSTNKAEPGEEVHLDLNAEPNSLCSLGVVDKSVELLQSNEDHLTLEGVFKVVKKAIVGDTENSQINDNEYCQKKREEDEADTTDPLRPGMPIPFAEEEPAGVVKRSIWHPYGHYTEGVDAIKMFEKSGLFVFTDLKVENRPCHFRVYHYLEDRIVSDDMALLDLAIPVSIVGPVFAALPQPAPPSPERRGEGGEEGEAVQLRSYFPETWLWELSFIPAAGNSSQALTLPHTITEWIGKAVCVHPEKGVGLSSKESITTFTPFFVDLTLPPSVQRGEILPVKISVFNYLEGALPVKVVLSESPEYEILEDPLATTVKGSASSCIPSKEKVVFTVKIRPNALGDVNLNVEAFVDELFPEDCGSEYVISKRDHIIKPIRVELEGFPQEKTWTKYMCTDGVDNEEQLVSWHLEAPSNIVPDSTRGWITAVGDLLGPTLDNLGSLVRMPYGCGEQNMVNFAPNIFVMQYLEAAEKTTADIATKAIEFMKSGYQRELRYRHKDGSFSAFGPSDESGSTWLTAFVLKSFAQAQQFIPIDTGDIDMSREWLKRDQMENGCFLSKGKVFHKSMKGGIAGNESPVPLTAYILTALLEAGELIVSRPISEAAFCLVSDKSEDPYTLALKAYALALAEAPEAAQFVVQLRGKATVSTEGMYWEMPPVNGKSAAAGVETAAYALLAMATLSPVDYLNDMQKLVKWISSKRNGQGGFVSTQDTVVALQALAKFEMVLGQKPVDVAVLASSTSLDHSFRITETNRLLLQRVDLPSFPTTVTADLAGEGCALVQAVLRYNIPEEDPSTAFNLTATTQTVRDDKCITKRIRACASYTLPDLKSNMAVIEINLVSGYIPDKNDLKQVVGYGTGLIKRYEVDGRKVTFYIDEFSPEDLCVAFKVTREVDVENPKPGTVRVYDYYDPDQFVSTSYTFPPNEECASGSDLDGVDILVIPSDDIAVDYIDYVV

transcript_transcript/363_Pt_Mix_transcript363/f55p0/5800

ATGGCGAGGTGGTGTCCCGCAAGGCGTCTCCTGGGACTGACCGTGTTTGTGGCGGTGGTGGCTTGGTGTTCAGGAAATTACATTATCACCACGCCGAGGAAGTGGGCAGAAGCGAGCGCCGCCCAAGTGTGCCTGTTCAACGTGGGCGGAGGCGTGGAGGAGGTGGAGGCGCCCGTTGAGGGAGTGGAGGTGAAGGTGGAAGGCGAGCACAGGAGTGCGGATGTGCTGGTTCCTCCTCAGTTTGTCTCCATTCCTCCGGGCCAGACGGAGGTGTGTCGGTCCATCCCAGTACCGGCGGTGGATCACATGAGGGGCAAACTCCACATCAACGGGACACTGGGAGGCAAGTCAGTCACCCACACTGAAACCATCAAGTTCGCCGCAGCCTCTCAGACATTCGTACAGACAGACAAGTTCCTTTACGCCCCGGGACAGAAGGTGCAGTTCAGGATCCTCACCGTTTACGGGCCTTACCTTAGAGTTTTCACAGGCATGTACCCGGAGATGTGGATCGAGTCCCCGTCCGGCAGCCGCATCGCCCAGTGGGTCAGAGTGGAGAGCTCCGGGGGTCTGATCCACCAGGAGTTCCAACTGATTAACGAGCCCGAGGAGGGGACGTATAAGATCCATGTGGAGTCGCCAGTGGGAGGATCCAAGGCGGTGCAGACATTCAAGATAGAGGATTTTGTGTTGCCGCGTTTTGAAGTCACCCTGCAGTCACCACCCTACATTCTGGCTACTACCAAGAGTCTGCACTACCGAGTTTGTGCTATGTACACGTATGGCCAGCCAGTAAAGGGAGAAGTGACCTTTGACATCCGTAAACCTGACCACTACAAAGTAACAAGACCGATCACCGGCTGTGAGAACTTCACCATTGCCATGAGCACCTCCCTAAGCACCAGTTATGGCCACTCCACCGTCAATGTGGTGGCCAAGGTGGTGGAGGAAGGCACGGGGGAGGAGGCGCAGGCTACAGGACAGATAGATGTACAGCGGAAGGTTCTTAAATTCAAGCATGTAGGGAAGGAGGAGTATGTCAAGCCTAATCTTCCCTACACTGGCCAGTTCAAGGTGACACTGCCCTCAGATGAACCAGCTGCCAACGAGCTGATGACAATCTGCAAAGGCGACACCTGCAGGAATATCACCACTGACGCTAGAGGGTTAGTGGAATACATCTACCCGAAGCATGAGGATTTCTCCATAGAGATCTCCTCACCAAGATACCCGCGAATTGAAAACCCCGAAAGGTCCTGGAGCCCAATTATGTACAAGTCAGCCGACAGTCACAGAGTCAAGACCTACTACTCCCCTTCAAACTCCTCCCTTGTGATCAAAACCCCGGAGGTCCAGCTGAGGTGCCACGCTGGGGAGACACAAAACTTCACGGTCTCTGTCATGTATGCTGGCACGAGGGACTCTAAGGCTAACTTCACCATACAGCTTGTCTCTAGAGGTCAAATCCAGTTCACTCACACTGAGGAACACACCCTTACTGACACCGACCTCCCCATTGACTCCTCCCTGCTGCTGGTGCCCCTGCCGCCCGCCACTGAGGGTGTAGCAAGGGGTGTGCTCAGCCTGCCCCTCACCATTCACCTCACAGCCTCCCCATCTGCTAAGGTGCTGGTCTGGTACACACGGCCAGACGGTGAGGTGGTGTCAGCAATGCAGGAGATCCGAATCAAGAAGTGCCTGACCAACTTGGTGAGTCTTGCCTGGTCCACTAATAAAGCTGAGCCTGGCGAGGAGGTCCACCTTGACCTTAATGCTGAACCAAACTCTCTCTGCAGTCTGGGTGTGGTGGACAAGAGTGTGGAGCTTCTGCAGTCAAATGAGGACCACCTAACACTGGAGGGTGTGTTTAAGGTGGTCAAGAAGGCAATAGTTGGTGACACAGAGAACTCACAGATTAACGATAATGAATACTGCCAAAAAAAGAGGGAGGAAGATGAAGCAGACACCACTGATCCTCTACGACCAGGAATGCCCATACCCTTTGCTGAGGAGGAGCCTGCTGGAGTGGTGAAGAGGTCCATTTGGCATCCCTACGGTCACTATACTGAGGGTGTGGATGCCATTAAAATGTTTGAGAAATCTGGACTATTTGTGTTTACGGATCTGAAGGTGGAGAACAGACCCTGCCACTTTCGTGGGCCAGTGTTTTACGACGATTACCCAGTGATGGCAATGAAAGTCCATTATGTAGATATGATGTTGGAAGCAAGTGAAATTCCGTCCATTATCCATCGCCATCCCCACACCACACAGCACTCCTCCCTGGAAACCACACGAACTTACTTCCCGGAAACATGGCTTTGGGAAATTATTATTATGAATTCAGCTGCTGGGAACAGCAGCCAGGCGCTCACCCTACCACACACCATCACTGAGTGGATTGGCAAGGCAGTGTGCGTCCACCCAGAGAAAGGTGTCGGCTTGTCATCAAAAGAGTCCATTACCACCTTCACCCCCTTCTTTGTTGACCTGACCCTGCCTCCCTCTGTGCAGCGCGGCGAGATCCTCCCTGTCAAGATATCAGTGTTTAACTATCTAGAAGGAGCACTGCCGGTCAAAGTAGTGTTATCCGAAAGCCCAGAATATGAGATCTTAGAGGACCCTCTGGCCACGACTGTCAAGGGTAGTGCTTCTTCCTGCATTCCATCAAAGGAGAAGGTTGTGTTCACCGTCAAGATCCGCCCCAATGCTCTAGGGGATGTGAATCTGAATGTGGAGGCTTTCGTGGATGAGTTATTCCCTGAGGATTGTGGATCTGAGTATGTCATCAGTAAGAGGGACCACATCATCAAGCCCATCCGTGTTGAGCTTGAAGGATTCCCACAGGAGAAGACCTGGACCAAGTATATGTGTACTGATGGTGTGGACAACGAGGAGCAACTAGTGTCCTGGCATCTGGAGGCACCATCAAACATTGTCCCTGACTCAACAAGGGGATGGATCACTGCTGTTGGAGACCTTCTGGGACCAACACTTGATAACCTTGGCTCGCTGGTGCGGATGCCATACGGGTGCGGAGAGCAGAACATGGTCAACTTTGCTCCGAATATCTTTGTCATGCAGTACTTAGAGGCGGCAGAGAAGACCACGGCAGACATCGCAACAAAGGCCATTGAGTTCATGAAGAGTGGATACCAGAGGGAGCTACGCTATCGCCACAAGGACGGATCATTCAGCGCCTTCGGTCCCAGTGATGAGTCAGGCTCTACTTGGCTAACTGCATTTGTACTAAAGTCCTTCGCCCAGGCCCAGCAGTTCATCCCGATTGACACGGGCGACATTGACATGAGTCGGGAGTGGCTCAAGAGAGACCAGATGGAGAATGGCTGCTTCCTCTCCAAGGGCAAAGTCTTCCATAAGTCCATGAAGGGAGGCATAGCGGGTAATGAGTCACCAGTACCCCTCACAGCCTACATCCTCACTGCACTGCTGGAGGCTGGAGAGCTTATTGTGAGTCGGCCGATCAGTGAGGCTGCATTCTGTCTGGTCTCCGATAAGAGTGAGGACCCCTACACACTGGCACTCAAGGCTTATGCTCTAGCTCTGGCTGAAGCACCCGAAGCAGCCCAATTCGTCGTTCAACTCAGGGGCAAGGCAACTGTGTCCACAGAAGGCATGTACTGGGAAATGCCTCCAGTTAATGGGAAGAGTGCGGCAGCGGGGGTGGAGACGGCAGCCTATGCCCTCCTAGCTATGGCCACCCTGAGCCCGGTAGACTACCTTAATGACATGCAGAAGTTGGTCAAGTGGATCTCCAGTAAGAGGAACGGACAGGGCGGCTTTGTCTCCACTCAGGACACAGTGGTAGCCCTACAAGCCCTGGCCAAGTTTGAGATGGTGCTGGGCCAGAAACCAGTGGACGTGGCGGTACTTGCGTCCTCCACCAGCCTGGATCACTCCTTCCGCATCACCGAAACCAACAGACTCCTCCTGCAGCGCGTCGACCTGCCCTCCTTCCCCACAACTGTCACCGCTGACCTGGCTGGGGAAGGGTGTGCGTTGGTGCAGGCTGTCCTCCGCTACAACATTCCTGAGGAGGATCCCAGCACGGCCTTCAACCTCACCGCCACCACACAGACGGTCAGAGATGACAAATGCATCACCAAACGCATCAGAGCCTGCGCCTCCTACACTCTGCCGGACCTCAAATCTAACATGGCTGTCATCGAGATTAACCTGGTGTCTGGCTACATACCAGACAAGAATGATCTGAAGCAGGTGGTTGGCTACGGGACGGGTCTGATCAAGAGGTACGAGGTGGACGGCAGGAAGGTCACATTCTATATTGATGAGTTCTCCCCTGAGGATCTCTGTGTGGCCTTCAAGGTGACTCGCGAGGTGGATGTGGAGAACCCAAAGCCAGGAACGGTCAGGGTGTACGACTACTACGACCCGGACCAGTTTGTTAGTACGAGCTACACCTTCCCGCCCAATGAGGAGTGTGCGTCGGGTTCTGATTTGGACGGGGTGGATATCTTGGTGATCCCCAGTGATGACATTGCCGTTGACTACATCGATTATGTTGTCTAA

MARWCPARRLLGLTVFVAVVAWCSGNYIITTPRKWAEASAAQVCLFNVGGGVEEVEAPVEGVEVKVEGEHRSADVLVPPQFVSIPPGQTEVCRSIPVPAVDHMRGKLHINGTLGGKSVTHTETIKFAAASQTFVQTDKFLYAPGQKVQFRILTVYGPYLRVFTGMYPEMWIESPSGSRIAQWVRVESSGGLIHQEFQLINEPEEGTYKIHVESPVGGSKAVQTFKIEDFVLPRFEVTLQSPPYILATTKSLHYRVCAMYTYGQPVKGEVTFDIRKPDHYKVTRPITGCENFTIAMSTSLSTSYGHSTVNVVAKVVEEGTGEEAQATGQIDVQRKVLKFKHVGKEEYVKPNLPYTGQFKVTLPSDEPAANELMTICKGDTCRNITTDARGLVEYIYPKHEDFSIEISSPRYPRIENPERSWSPIMYKSADSHRVKTYYSPSNSSLVIKTPEVQLRCHAGETQNFTVSVMYAGTRDSKANFTIQLVSRGQIQFTHTEEHTLTDTDLPIDSSLLLVPLPPATEGVARGVLSLPLTIHLTASPSAKVLVWYTRPDGEVVSAMQEIRIKKCLTNLVSLAWSTNKAEPGEEVHLDLNAEPNSLCSLGVVDKSVELLQSNEDHLTLEGVFKVVKKAIVGDTENSQINDNEYCQKKREEDEADTTDPLRPGMPIPFAEEEPAGVVKRSIWHPYGHYTEGVDAIKMFEKSGLFVFTDLKVENRPCHFRGPVFYDDYPVMAMKVHYVDMMLEASEIPSIIHRHPHTTQHSSLETTRTYFPETWLWEIIIMNSAAGNSSQALTLPHTITEWIGKAVCVHPEKGVGLSSKESITTFTPFFVDLTLPPSVQRGEILPVKISVFNYLEGALPVKVVLSESPEYEILEDPLATTVKGSASSCIPSKEKVVFTVKIRPNALGDVNLNVEAFVDELFPEDCGSEYVISKRDHIIKPIRVELEGFPQEKTWTKYMCTDGVDNEEQLVSWHLEAPSNIVPDSTRGWITAVGDLLGPTLDNLGSLVRMPYGCGEQNMVNFAPNIFVMQYLEAAEKTTADIATKAIEFMKSGYQRELRYRHKDGSFSAFGPSDESGSTWLTAFVLKSFAQAQQFIPIDTGDIDMSREWLKRDQMENGCFLSKGKVFHKSMKGGIAGNESPVPLTAYILTALLEAGELIVSRPISEAAFCLVSDKSEDPYTLALKAYALALAEAPEAAQFVVQLRGKATVSTEGMYWEMPPVNGKSAAAGVETAAYALLAMATLSPVDYLNDMQKLVKWISSKRNGQGGFVSTQDTVVALQALAKFEMVLGQKPVDVAVLASSTSLDHSFRITETNRLLLQRVDLPSFPTTVTADLAGEGCALVQAVLRYNIPEEDPSTAFNLTATTQTVRDDKCITKRIRACASYTLPDLKSNMAVIEINLVSGYIPDKNDLKQVVGYGTGLIKRYEVDGRKVTFYIDEFSPEDLCVAFKVTREVDVENPKPGTVRVYDYYDPDQFVSTSYTFPPNEECASGSDLDGVDILVIPSDDIAVDYIDYVV

transcript_transcript/366_Pt_Mix_transcript366/f16p0/5783

ATGGCGAGGTGGTGTCCCGCAAGGCGTCTCCTGGGACTGACCGTGTTTGTGGCGGTGGTGGCTTGGTGTTCAGGAAATTACATTATCACCACGCCGAGGAAGTGGGCAGAAGCGAGCGCCGCCCAAGTGTGCCTGTTCAACGTGGGCGGAGGCGTGGAGGAGGTGGAGGCGCCCGTTGAGGGAGTGGAGGTGAAGGTGGAAGGCGAGCACAGGAGTGCGGATGTGCTGGTTCCTCCTCAGTTTGTCTCCATTCCTCCGGGCCAGACGGAGGTGTGTCGGTCCATCCCAGTACCGGCGGTGGATCACATGAGGGGCAAACTCCACATCAACGGGACACTGGGAGGCAAGTCAGTCACCCACACTGAAACCATCAAGTTCGCCGCAGCCTCTCAGACATTCGTACAGACAGACAAGTTCCTTTACGCCCCGGGACAGAAGGTGCAGTTCAGGATCCTCACCGTTTACGGGCCTTACCTTAGAGTTTTCACAGGCATGTACCCGGAGATGTGGATCGAGTCCCCGTCCGGCAGCCGCATCGCCCAGTGGGTCAGAGTGGAGAGCCCCGGGGGTCTGATCCACCAGGAGTTCCAACTGATTAACGAGCCCGAGGAGGGGACGTATAAGATCCATGTGGAGTCGCCAGTGGGAGGATCCAAGGCGGTGCAGACATTCAAGATAGAGGATTTTGTGTTGCCGCGTTTTGAAGTCACCCTGCAGTCACCACCCTACATTCTGGCTACTACCAAGAGTCTGCACTACCGAGTTTGTGCTATGTACACGTATGGCCAGCCAGTAAAGGGAGAAGTGACCTTTGACATCCGTAAACCTGACCACTACAAAGTAACAAGACCGATCACCGGCTGTGAGAACTTCACCATTGCCATGAGCACCTCCCTAAGCACCAGTTATGGCCACTCCACCGTCAATGTGGTGGCCAAGGTGGTGGAGGAAGGCACGGGGGAGGAGGCGCAGGCTACAGGACAGATAGATGTACAGCGGAAGGTTCTTAAATTCAAGCATGTAGGGAAGGAGGAGTATGTCAAGCCTAATCTTCCCTACACTGGCCAGTTCAAGGTGACACTGCCCTCAGATGAACCAGCTGCCAACGAGCTGATGACAATCTGCAAAGGCGACACCTGCAGGAATATCACCACTGACGCTAGAGGGTTAGTGGAATACATCTACCCGAAGCATGAGGATTTCTCCATAGAGATCTCCTCACCAAGATACCCGCGAATTGAAAACCCCGAAAGGTCCTGGAGCCCAATTATGTACAAGTCAGCCGACAGTCACAGAGTCAAGACCTACTACTCCCCTTCAAACTCCTCCCTTGTGATCAAAACCCCGGAGGTCCAGCTGAGGTGCCACGCTGGGGAGACACAAAACTTCACGGTCTCTGTCATGTATGCTGGCACGAGGGACTCTAAGGCTAACTTCACCATACAGCTTGTCTCTAGAGGTCAAATCCAGTTCACTCACACTGAGGAACACACCCTTACTGACACCGACCTCCCCATTGACTCCTCCCTGCTGCTGGTGCCCCTGCCGCCCGCCACTGAGGGTGTAGCAAGGGGTGTGCTCAGCCTGCCCCTCACCATTCACCTCACAGCCTCCCCATCTGCTAAGGTGCTGGTCTGGTACACACGGCCAGACGGTGAGGTGGTGTCAGCAATGCAGGAGATCCGAATCAAGAAGTGCCTGACCAACTTGGTGAGTCTTGCCTGGTCCACTAATAAAGCTGAGCCTGGCGAGGAGGTCCACCTTGACCTTAATGCTGAACCAAACTCTCTCTGCAGTCTGGGTGTGGTGGACAAGAGTGTGGAGCTTCTGCAGTCAAATGAGGACCACCTAACACTGGAGGGTGTGTTTAAGGTGGTCAAGAAGGCAATAGTTGGTGACACAGAGAACTCACAGATTAACGATAATGAATACTGCCAAAAAAAGAGGGAGGAAGATGAAGCAGACACCACTGATCCTCTACGACCAGGAATGCCCATACCCTTTGCTGAGGAGGAGCCTGCTGGAGTGGTGAAGAGGTCCATTTGGCATCCCTACGGTCACTATACTGAGGGTGTGGATGCCATTAAAATGTTTGAGAAATCTGGACTATTTGTGTTTACGGATCTGAAGGTGGAGAACAGACCCTGCCACTTTCGTGATTGGCGCATACCCTATGCAGCCCATGGAAATGGAGTGGCTGTGGCAGCAGGAGTTGACTATATGATACGAGGACCTCCAATGGGAAGGCCAGCCCCTCATTATCATGGGATTCCCACTCGATCTTACTTCCCTGAGACATGGTTGTGGAAACTGCTATCCTTGCCAGCTGCTGGGAACAGCAGCCAGGCGCTCACCCTACCACACACCATCACTGAGTGGATTGGCAAGGCAGTGTGCGTCCACCCAGAGAAAGGTGTCGGCTTGTCATCAAAAGAGTCCATTACCACCTTCACCCCCTTCTTTGTTGACCTGACCCTGCCTCCCTCTGTGCAGCGCGGCGAGATCCTCCCTGTCAAGATATCAGTGTTTAACTATCTAGAAGGAGCACTGCCGGTCAAAGTAGTGTTATCCGAAAGCCCAGAATATGAGATCTTAGAGGACCCTCTGGCCACGACTGTCAAGGGTAGTGCTTCTTCCTGCATTCCATCAAAGGAGAAGGTTGTGTTCACCGTCAAGATCCGCCCCAATGCTCTAGGGGATGTGAATCTGAATGTGGAGGCTTTCGTGGATGAGTTATTCCCTGAGGATTGTGGATCTGAGTATGTCATCAGTAAGAGGGACCACATCATCAAGCCCATCCGTGTTGAGCTTGAAGGATTTCCACAGGAGAAGACCTGGACCAAGTATATGTGTACTGATGGTGTGGACAACGAGGAGCAACTAGTGTCCTGGCATCTGGAGGCACCATCAAACATTGTCCCTGACTCAACAAGGGGATGGATCACTGCTGTTGGAGACCTTCTGGGACCAACACTTGATAACCTTGGCTCGCTGGTGCGGATGCCATACGGGTGCGGAGAGCAGAACATGGTCAACTTTGCTCCGAATATCTTTGTCATGCAGTACTTAGAGGCGGCAGAGAAGACCACGGCAGACATCGCAACAAAGGCCATTGAGTTCATGAAGAGTGGATACCAGAGGGAGCTACGCTATCGCCACAAGGACGGATCATTCAGCGCCTTCGGTCCCAGTGATGAGTCAGGCTCTACTTGGCTAACTGCATTTGTACTAAAGTCCTTCGCCCAGGCCCAGCAGTTCATCCCGATTGACACGGGCGACATTGACATGAGTCGGGAGTGGCTCAAGAGAGACCAGATGGAGAATGGCTGCTTCCTCTCCAAGGGCAAAGTCTTCCATAAGTCCATGAAGGGAGGCATAGCGGGTAATGAGTCACCAGTACCCCTCACAGCCTACATCCTCACTGCACTGCTGGAGGCTGGAGAGCTTATTGTGAGTCGGCCGATCAGTGAGGCTGCATTCTGTCTGGTCTCCGATAAGAGTGAGGACCCCTACACACTGGCACTCAAGGCTTATGCTCTAGCTCTGGCTGAAGCACCCGAAGCAGCCCAATTCGTCGTTCAACTCAGGGGCAAGGCAACTGTGTCCACAGAAGGCATGTACTGGGAAATGCCTCCAGTTAATGGGAAGAGTGCGGCAGCGGGGGTGGAGACGGCAGCCTATGCCCTCCTAGCTATGGCCACCCTGAGCCCGGTAGACTACCTTAATGACATGCAGAAGTTGGTCAAGTGGATCTCCAGTAAGAGGAACGGACAGGGCGGCTTTGTCTCCACTCAGGACACAGTGGTAGCCCTACAAGCCCTGGCCAAGTTTGAGATGGTGCTGGGCCAGAAACCAGTGGACGTGGCGGTACTTGCGTCCTCCACCAGCCTGGATCACTCCTTCCGCATCACCGAAACCAACAGACTCCTCCTGCAGCGCGTCGACCTGCCCTCCTTCCCCACAACTGTCACCGCTGACCTGGCTGGGGAAGGGTGTGCGTTGGTGCAGGCTGTCCTCCGCTACAACATTCCTGAGGAGGATCCCAGCACGGCCTTCAACCTCACCGCCACCACACAGACGGTCAGAGATGACAAATGCATCACCAAACGCATCAGAGCCTGCGCCTCCTACACTCTGCCGGACCTCAAATCTAACATGGCTGTCATCGAGATTAACCTGGTGTCTGGCTACATACCAGACAAGAATGATCTGAAGCAGGTGGTTGGCTACGGGACGGGTCTGATCAAGAGGTACGAGGTGGACGGCAGGAAGGTCACATTCTATATTGATGAGTTCTCCCCTGAGGATCTCTGTGTGGCCTTCAAGGTGACTCGCGAGGTGGATGTGGAGAACCCAAAGCCAGGAACGGTCAGGGTGTACGACTACTACGACCCGGACCAGTTTGTTAGTACGAGCTACACCTTCCCGCCCAATGAGGAGTGTGCGTCGGGTTCTGATTTGGACGGGGTGGATATCTTGGTGATCCCCAGTGATGACATTGCCGTTGACTACATCGATTATGTTGTCTAA

MARWCPARRLLGLTVFVAVVAWCSGNYIITTPRKWAEASAAQVCLFNVGGGVEEVEAPVEGVEVKVEGEHRSADVLVPPQFVSIPPGQTEVCRSIPVPAVDHMRGKLHINGTLGGKSVTHTETIKFAAASQTFVQTDKFLYAPGQKVQFRILTVYGPYLRVFTGMYPEMWIESPSGSRIAQWVRVESPGGLIHQEFQLINEPEEGTYKIHVESPVGGSKAVQTFKIEDFVLPRFEVTLQSPPYILATTKSLHYRVCAMYTYGQPVKGEVTFDIRKPDHYKVTRPITGCENFTIAMSTSLSTSYGHSTVNVVAKVVEEGTGEEAQATGQIDVQRKVLKFKHVGKEEYVKPNLPYTGQFKVTLPSDEPAANELMTICKGDTCRNITTDARGLVEYIYPKHEDFSIEISSPRYPRIENPERSWSPIMYKSADSHRVKTYYSPSNSSLVIKTPEVQLRCHAGETQNFTVSVMYAGTRDSKANFTIQLVSRGQIQFTHTEEHTLTDTDLPIDSSLLLVPLPPATEGVARGVLSLPLTIHLTASPSAKVLVWYTRPDGEVVSAMQEIRIKKCLTNLVSLAWSTNKAEPGEEVHLDLNAEPNSLCSLGVVDKSVELLQSNEDHLTLEGVFKVVKKAIVGDTENSQINDNEYCQKKREEDEADTTDPLRPGMPIPFAEEEPAGVVKRSIWHPYGHYTEGVDAIKMFEKSGLFVFTDLKVENRPCHFRDWRIPYAAHGNGVAVAAGVDYMIRGPPMGRPAPHYHGIPTRSYFPETWLWKLLSLPAAGNSSQALTLPHTITEWIGKAVCVHPEKGVGLSSKESITTFTPFFVDLTLPPSVQRGEILPVKISVFNYLEGALPVKVVLSESPEYEILEDPLATTVKGSASSCIPSKEKVVFTVKIRPNALGDVNLNVEAFVDELFPEDCGSEYVISKRDHIIKPIRVELEGFPQEKTWTKYMCTDGVDNEEQLVSWHLEAPSNIVPDSTRGWITAVGDLLGPTLDNLGSLVRMPYGCGEQNMVNFAPNIFVMQYLEAAEKTTADIATKAIEFMKSGYQRELRYRHKDGSFSAFGPSDESGSTWLTAFVLKSFAQAQQFIPIDTGDIDMSREWLKRDQMENGCFLSKGKVFHKSMKGGIAGNESPVPLTAYILTALLEAGELIVSRPISEAAFCLVSDKSEDPYTLALKAYALALAEAPEAAQFVVQLRGKATVSTEGMYWEMPPVNGKSAAAGVETAAYALLAMATLSPVDYLNDMQKLVKWISSKRNGQGGFVSTQDTVVALQALAKFEMVLGQKPVDVAVLASSTSLDHSFRITETNRLLLQRVDLPSFPTTVTADLAGEGCALVQAVLRYNIPEEDPSTAFNLTATTQTVRDDKCITKRIRACASYTLPDLKSNMAVIEINLVSGYIPDKNDLKQVVGYGTGLIKRYEVDGRKVTFYIDEFSPEDLCVAFKVTREVDVENPKPGTVRVYDYYDPDQFVSTSYTFPPNEECASGSDLDGVDILVIPSDDIAVDYIDYVV

transcript_transcript/490_Pt_Mix_transcript490/f2p0/5596

ATGAGGGGCAAACTCCACATCAACGGGACACTGGGAGGCAAGTCAGTCACCCACACTGAAACCATCAAGTTCGCCGCAGCCTCTCAGACATTCGTACAGACAGACAAGTTCCTTTACGCCCCGGGACAGAAGGTGCAGTTCAGGATCCTCACCGTTTACGGGCCTTACCTTAGAGTTTTCACAGGCATGTACCCGGAGATGTGGATCGAGTCCCCGTCCGGCAGCCGCATCGCCCAGTGGGTCAGAGTGGAGAGCCCCGGGGGTCTGATCCACCAGGAGTTCCAACTGATTAACGAGCCCGAGGAGGGGACGTATAAGATCCATGTGGAGTCGCCAGTGGGAGGATTCAAGGCGGTGCAGACATTCAAGATAGAGGATTTTGTGTTGCCGCGTTTTGAAGTCACCCTGCAGTCACCACCCTACATTCTGGCTACTACCAAGAGTCTGCACTACCGAGTTTGTGCTATGTACACGTATGGCCAGCCAGTAAAGGGAGAAGTGACCTTTGACATCCGTAAACCTGACCACTACAAAGTAACAAGACCGATCACCGGCTGTGAGAACTTCACCATTGCCATGAGCACCTCCCTAAGCACCAGTTATGGCCACTCCACCGTCAATGTGGTGGCCAAGGTGGTGGAGGAAGGCACGGGGGAGGAGGCGCAGGCTACAGGACAGATAGATGTACAGCGGAAGGTTCTTAAATTCAAGCATGTAGGGAAGGAGGAGTATGTCAAGCCTAATCTTCCCTACACTGGCCAGTTCAAGGTGACACTGCCCTCAGATGAACCAGCTGCCAACGAGCTGATGACAATCTGCAAAGGCGACACCTGCAGGAATATCACCACTGACGCTAGAGGGTTAGTGGAATACATCTACCCGAAGCATGAGGATTTCTCCATAGAGATCTCCTCACCAAGATACCCGCGAATTGAAAACCCCGAAAGGTCCTGGAGCCCAATTATGTACAAGTCAGCCGACAGTCACAGAGTCAAGACCTACTACTCCCCTTCAAACTCCTCCCTTGTGATCAAAACCCCGGAGGTCCAGCTGAGGTGCCACGCTGGGGAGACACAAAACTTCACGGTCTCTGTCATGTATGCTGGCACGAGGGACTCTAAGGCTAACTTCACCATACAGCTTGTCTCTAGAGGTCAAATCCAGTTCACTCACACTGAGGAACACACCCTTACTGACACCGACCTCCCCATTGACTCCTCCCTGCTGCTGGTGCCCCTGCCGCCCGCCACTGAGGGTGTAGCAAGGGGTGTGCTCAGCCTGCCCCTCACCATTCACCTCACAGCCTCCCCATCTGCTAAGGTGCTGGTCTGGTACACACGGCCAGACGGTGAGGTGGTGTCAGCAATGCAGGAGATCCGAATCAAGAAGTGCCTGACCAACTTGGTGAGTCTTGCCTGGTCCACTAATAAAGCTGAGCCTGGCGAGGAGGTCCACCTTGACCTTAATGCTGAACCAAACTCTCTCTGCAGTCTGGGTGTGGTGGACAAGAGTGTGGAGCTTCTGCAGTCAAATGAGGACCACCTAACACTGGAGGGTGTGTTTAAGGTGGTCAAGAAGGCAATAGTTGGTGACACAGAGAACTCACAGATTAACGATAATGAATACTGCCAAAAAAAGAGGGAGGAAGATGAAGCAGACACCACTGATCCTCTACGACCAGGAATGCCCATACCCTTTGCTGAGGAGGAGCCTGCTGGAGTGGTGAAGAGGTCCATTTGGCATCCCTACGGTCACTATACTGAGGGTGTGGATGCCATTAAAATGTTTGAGAAATCTGGACTATTTGTGTTTACGGATCTGAAGGTGGAGAACAGACCCTGCCACTTTCGTGTTTATCACTACTTAGAAGACCGCATAGTAAGTGATGACATGGCTCTCCTTGACTTGGCAATTCCAGTGTCAATTGTTGGGCCAGTATTTGCAGCACTACCACAACCAGCACCACCATCACCAGAAAGAAGAGGAGAGGGAGGAGAGGAGGGAGAGGCAGTACAGTTGCGTTCTTACTTCCCCGAGACTTGGCTCTGGGAACTCTCTTTCATTCCAGCTGCTGGGAACAGCAGCCAGGCGCTCACCCTACCACACACCATCACTGAGTGGATTGGCAAGGCAGTGTGCGTCCACCCAGAGAAAGGTGTCGGCTTGTCATCAAAAGAGTCCATTACCACCTTCACCCCCTTCTTTGTTGACCTGACCCTGCCTCCCTCTGTGCAGCGCGGCGAGATCCTCCCTGTCAAGATATCAGTGTTTAACTATCTAGAAGGAGCACTGCCGGTCAAAGTAGTGTTATCCGAAAGCCCAGAATATGAGATCTTAGAGGACCCTCTGGCCACGACTGTCAAGGGTAGTGCTTCTTCCTGCATTCCATCAAAGGAGAAGGTTGTGTTCACCGTCAAGATCCGCCCCAATGCTCTAGGGGATGTGAATCTGAATGTGGAGGCTTTCGTGGATGAGTTATTCCCTGAGGATTGTGGATCTGAGTATGTCATCAGTAAGAGGGACCACATCATCAAGCCCATCCGTGTTGAGCTTGAAGGATTCCCACAGGAGAAGACCTGGACCAAGTATATGTGTACTGATGGTGTGGACAACGAGGAGCAACTAGTGTCCTGGCATCTGGAGGCACCATCAAACATTGTCCCTGACTCAACAAGGGGATGGATCACTGCTGTTGGAGACCTTCTGGGACCAACACTTGATAACCTTGGCTCGCTGGTGCGGATGCCATACGGGTGCGGAGAGCAGAACATGGTCAACTTTGCTCCGAATATCTTTGTCATGCAGTACTTAGAGGCGGCAGAGAAGACCACGGCAGACATCGCAACAAAGGCCATTGAGTTCATGAAGAGTGGATACCAGAGGGAGCTACGCTATCGCCACAAGGACGGATCATTCAGCGCCTTCGGTCCCAGTGATGAGTCAGGCTCTACTTGGCTAACTGCATTTGTACTAAAGTCCTTCGCCCAGGCCCAGCAGTTCATCCCGATTGACACGGGCGACATTGACATGAGTCGGGAGTGGCTCAAGAGAGACCAGATGGAGAATGGCTGCTTCCTCTCCAAGGGCAAAGTCTTCCACAAGTCCATGAAGGGAGGCATAGCGGGTAATGAGTCACCAGTACCCCTCACAGCCTACATCCTCACTGCACTGCTGGAGGCTGGAGAGCTTATTGTGAGTCGGCCGATCAGTGAGGCTGCATTCTGTCTGGTCTCCGATAAGAGTGAGGACCCCTACACACTGGCACTCAAGGCTTATGCTCTAGCTCTGGCTGAAGCACCCGAAGCAGCCCAATTCGTCGTTCAACTCAGGGGCAAGGCAACTGTGTCCACAGAAGGCATGTACTGGGAAATGCCTCCAGTTAATGGGAAGAGTGCGGCAGCGGGGGTGGAGACGGCAGCCTATGCCCTCCTAGCTATGGCCACCCTGAGCCCGGTAGACTACCTTAATGACATGCAGAAGTTGGTCAAGTGGATCTCCAGTAAGAGGAACGGACAGGGCGGCTTTGTCTCCACTCAGGACACAGTGGTAGCCCTACAAGCCCTGGCCAAGTTTGAGATGGTGCTGGGCCAGAAACCAGTGGACGTGGCGGTACTTGCGTCCTCCGCCAGCCTGGATCACTCCTTCCGCATCACCGAAACCAACAGACTCCTCCTGCAGCGTGTCGACCTGCCCTCCTTCCCCACAACTGTCACCGCTGACCTGGCTGGGGAAGGGTGTGCGTTGGTGCAGGCTGTCCTCCGCTACAACATTCCTGAGGAGGATCCCAGCACGGCCTTCAACCTCACCGCCACCACACAGACGGTCAGAGATGACAAATGCATCACCAAACGCATCAGAGCCTGCGCCTCCTACACTCTGCCGGACCTCAAATCTAACATGGCTGTCATCGAGATTAACCTGGTGTCTGGCTACATACCAGACAAGAATGATCTGAAGCAGGTGGTTGGCTACGGGACGGGTCTGATCAAGAGGTACGAGGTGGACGGCAGGAAGGTCACATTCTATATTGATGAGTTCTCCCCTGAGGATCTCTGTGTGGCCTTCAAGGTGACTCGCGAGGTGGATGTGGAGAACCCAAAGCCAGGAACGGTCAGGGTGTACGACTACTACGACCCGGACCAGTTTGTTAGTACGAGCTACACCTTCCCGCCCAATGAGGAGTGTGCGTCGGGTTCTGATTTGGACGGGGTGGATATCTTGGTGATCCCCAGTGATGACATTGCCGTTGACTACATCGATTATCTTGTCTAA

MRGKLHINGTLGGKSVTHTETIKFAAASQTFVQTDKFLYAPGQKVQFRILTVYGPYLRVFTGMYPEMWIESPSGSRIAQWVRVESPGGLIHQEFQLINEPEEGTYKIHVESPVGGFKAVQTFKIEDFVLPRFEVTLQSPPYILATTKSLHYRVCAMYTYGQPVKGEVTFDIRKPDHYKVTRPITGCENFTIAMSTSLSTSYGHSTVNVVAKVVEEGTGEEAQATGQIDVQRKVLKFKHVGKEEYVKPNLPYTGQFKVTLPSDEPAANELMTICKGDTCRNITTDARGLVEYIYPKHEDFSIEISSPRYPRIENPERSWSPIMYKSADSHRVKTYYSPSNSSLVIKTPEVQLRCHAGETQNFTVSVMYAGTRDSKANFTIQLVSRGQIQFTHTEEHTLTDTDLPIDSSLLLVPLPPATEGVARGVLSLPLTIHLTASPSAKVLVWYTRPDGEVVSAMQEIRIKKCLTNLVSLAWSTNKAEPGEEVHLDLNAEPNSLCSLGVVDKSVELLQSNEDHLTLEGVFKVVKKAIVGDTENSQINDNEYCQKKREEDEADTTDPLRPGMPIPFAEEEPAGVVKRSIWHPYGHYTEGVDAIKMFEKSGLFVFTDLKVENRPCHFRVYHYLEDRIVSDDMALLDLAIPVSIVGPVFAALPQPAPPSPERRGEGGEEGEAVQLRSYFPETWLWELSFIPAAGNSSQALTLPHTITEWIGKAVCVHPEKGVGLSSKESITTFTPFFVDLTLPPSVQRGEILPVKISVFNYLEGALPVKVVLSESPEYEILEDPLATTVKGSASSCIPSKEKVVFTVKIRPNALGDVNLNVEAFVDELFPEDCGSEYVISKRDHIIKPIRVELEGFPQEKTWTKYMCTDGVDNEEQLVSWHLEAPSNIVPDSTRGWITAVGDLLGPTLDNLGSLVRMPYGCGEQNMVNFAPNIFVMQYLEAAEKTTADIATKAIEFMKSGYQRELRYRHKDGSFSAFGPSDESGSTWLTAFVLKSFAQAQQFIPIDTGDIDMSREWLKRDQMENGCFLSKGKVFHKSMKGGIAGNESPVPLTAYILTALLEAGELIVSRPISEAAFCLVSDKSEDPYTLALKAYALALAEAPEAAQFVVQLRGKATVSTEGMYWEMPPVNGKSAAAGVETAAYALLAMATLSPVDYLNDMQKLVKWISSKRNGQGGFVSTQDTVVALQALAKFEMVLGQKPVDVAVLASSASLDHSFRITETNRLLLQRVDLPSFPTTVTADLAGEGCALVQAVLRYNIPEEDPSTAFNLTATTQTVRDDKCITKRIRACASYTLPDLKSNMAVIEINLVSGYIPDKNDLKQVVGYGTGLIKRYEVDGRKVTFYIDEFSPEDLCVAFKVTREVDVENPKPGTVRVYDYYDPDQFVSTSYTFPPNEECASGSDLDGVDILVIPSDDIAVDYIDYLV

transcript_transcript/208_Pt_Mix_transcript208/f2p0/6308

ATGAAGTGGGCGGCGGCGGTGGCGGTGGTGGTGGTGGCCGTGGGCGTGATGGTGGGCGGCGCTGAGGCCAAAGGGACCTACAGCGTGGTGGCCTCCAGAGTGCTCCGGCCCAACCAGGTTTTCCACGTAGCGGTGTCTAGCCACGGCACGGAGGGGGATGTGCAAGTGAGCGCAGAGGTCGGAGGGGAGCAGGATTCAGGCAATATCATTCTGCTCAGACAGATTGCCGACCTCCAGCCTGACTCTACCCAAGTTTTGAAGTTTGAGATAGGAGACATAGGGCCTGGCCAGTACAACCTCTCCGTGTCTGGCTTCGGGGCCCTCACCTTCAGGAACACCACGAAGCTCGACTACGTGCACAAATCCTACTCTGCCTTCATCCAGACTGACAAGACGATCTACAAACCCGGCGACCTGATGCGCTTCCGTGTGGTTGTAGTGAACCCATTGCTGAGACCCTTCGTTACTGGTTCCATCGATGTTTATGTGACGGACGGAGCAGGACACAGGGTAAGGCAGTGGAAACGAGTTTTCACCAACAAGGGGGTGTGGTCAGGGGAGCTGCAGCTGGCGAAGGAGCCTGTGCTAGGGGATTGGAACATCACGGTGGAGGTGCTGGGCCAGAAGACTTCCAGGGCGGTGCAGGTGGCTTACTACGTCCTCCCAAAGTTCGAGGTTATAGTGACGCTGCCTGAATATGTGACGTTTGACCAGGGCGAGATGGTGGCGACGGTGGAGGCAAAATACACGTACGGGAGGGCAGTTAAGGGCGAGGTCACACTGCAGGTCACCCCCACCTACAAGTATGGCTACCTGCAGGCGCCCTACGATGACCCTATCCGCGTGGTCAAGGCTATCAAGGGCAAGACTGATGTTACCATTGACCTGCTGAGTGACGCGAGACTCAAGGGAGACTACGCCAGGGAACTCGAGGTCACAGCGTACGTGAAGGAGGAGCTGACGGAGAGGGTTCAGAATGCCACCTCGCACGTCACAGTTTACCGCTACCCGTACCGCCTTTCTCTCATCCGTACCTCCGATTCCTTCAAGCCGGGCCTCACCTACACCGCCTTTCTCAAAGTGTCCTACCAAGACGACACCCCGGTCACTTCTGGCGAGGTTACCGTGAGACACTCCTTCACACGGGACCCCAGTGGCTTTACGGAGGAGGTTCACACTATAGAACCTTCAGGAATTGTGACTCTACAGTTCACCCCGCCCCTGGATGAGAGTGTGGTGAGCCTGGCGCTGGAGGCACGCTACAAGGACCTGACGCAGTGGCTAGGGGACATCGTGAGGGCGCAGAGTCCCAGTAATTCCTTCCTGCAGGCGACTCTACAGACGGAGAATCCAAGAGTTGGTGGCGAGATACTGATTGGCCTGAATGCAACCCAGCCGCTGGTGTATTTTGTCTATCAAGTGCTGGGGCGAGGAGATGTGGTGTTCAGTAATACCCTGCAGGCTCACCAGGGCACCACTCACACCTTCAGGTTCCTGGCCACCCGAGACATGGCCCCACGTGCTCGCCTCCTGATGTACTACGTGAGGGATGATGGGGAGGTGGTGGCTGACTCACTCCACTTCACCGTGTCTGGGGCAATTCAGAATGAGGTGACAGTTAACCTCAACCCGAATGTGGTGGATGCAGCAGGTGAGGTAGACATCACCGTCACCACCAAACCCAATGCCTTTGTCGGAGTGTTGGCGGTGGACCAGCGTGCCCTCATGCTCGGCACCCACAATCACTTCTCCCAGCATGAGGTGATTGATGAGCTGGAGACATACGACCCTGGCAGAAAGACCTTGGAGGCGCCCTGGCACGCCCTTAGCAGAAAGAAGCGAGCTCTCTTCAACTGGATGGGCACCACAACCACCAGTGATGTCTTCAAGAACGCTGGCATTGTGGTGCTGACTAATGGTTACGTCCACGACTTCAATCCATTCCCCACAGCCAATCAACCTCCCAGCGACATCACAGGCGAGATTCCCTTCCACCGACAAACCGCCGTCAATGGATCCTTTGGCCGCGAGTTTGCCCCCATCGATGCCTCCACCCTGCGTCCTGACCTTGGTCCCGGCCTTGCCTACAATGCCCCCACACGTCCCCCGCTGGCCGGCCCCTATGCCTTCTCCTACCTGCCTCCACCGCCTGACTCCAGGCCACGTCTCTACCTCAACCAGCATGTTCCTCCCACCTGGCTCTTCCTTGATGCTGAGACAAAGTTCAATGGTGTGGTGAGGATGCGGGAGAAGGCACCAGACGCCATCACCTCCTACATGATCTCAGCCTTTGCCATTGATGATCTCTACGGCCTTGGGGTGACAGAGCGCCCTACCAAGCTGCGAGTGTTCCGGCCATTCTTTGTGTCAATGAACCTCCCTGCGGCGGGCGTGGTGCGCGGGGAGGCCGTGGCGGTGGAGATGGTCATTTTCAACTATGGGGATCAACAGGCCACAGCGCAGGTCACTCTGGAGAATCCCAATGAGGACTTCCTGTTTGCTGACTTTGCCAATGAGATCGACCAAGGCTCCCCAGCAGCAATGAAGAGCAGAGAGGTTGTGGTGGCATCAGGTACTGGCATGAGTGTCAGCTTCATGGTGGTGCCACAGACTATCGGCAACATCCCCATCACCGTGAGGGCCACCACTGCCTCCGCTGCTGATGTGGTCACCAAGCAGCTTCTTGTCAAGCCCGAAGGAACTAGACAGACGGTGAACAGAGCCATGCTGATTGACCTCCGCTCCGAGACTTCCTTCTCCGCTACTGTCAACATCACCACTCCGCCCAACGTTGTCAATGAATCCAAGGCAATCTCCGTCACTCTCGTTGGTGATGTGCTTGGTCCTGCTGTGAGTGACCTGCAGAGTCTGGTGGAGCTGCCAACAGGGTGTGGTGAGCAGAACATGGCCAAGTTGGTGCCCAACATTGTGGTGACGGAGTACCTCAAGAACAAGAACCAGCTGGATGAGTCTCTGCTGGGGCGTGCCAAGAGACACCTGGAGACTGGCTACCAGCAGCAGCTCAACTACCGTCACCCTGATGGCTCCTTCAGTGCCTTTGGGAGTCGGGACAAGTCGGGCAGCACCTGGCTGACGGCATTCGTGGCCAAGTCACTGGCGGAGGCTGGGCGGCACATCGAGGTGGAGGCTGAGGTGGTAGATAAAGCAGTGGAGTGGTTGAGGAGCCAGCAGGTAGCGGACGGCAGTTTTCCCGAGGTGGGGACAGTGAACAACAAGGCCATGCAGGGCGGAGCGGCCTTTGGGTCAGCACTCACAGCTTATGTGGTCATGGCGCTGCTCACCACTCAGAACCCACCCAAAGCAATTGTCAGGAACAGCATCAACAGAGGCCTGGACTTCCTGGCCAAGCGACTGGATGACATAGAGGACTTGTACAGCCTTGCCCTCACCACCTATGTACTGCACCTCGCTGACCACACACACAAGGATGCTGCCTTCTTCAAGCTGGAGAGCAAAGCTAAAATACAAGAGAATGAGAAGTGGTGGGAGTCTGGGCCACTGAACAGGACAGTGGAGACAATGGTGGACACGCGGTCTGTAGACGTGGAGATGACGGGATATGGCCTGATGACCTATGTGCGCCGTGGCTTGGTGCAGGATGCCCTACCCATCATGCGCTGGTTGGTGAGGCGACGCAACACACACGGCGGCTTCATGAGCACACAGGACACTGTGGTGGCGTTGGCGGGACTGGCGGGGCTGGCTGAGCGACTTACTGCTGCTAATTCTCGAGTCAACCTGAGACTGATCTATGGCGCACGAGGCAAGAACCTTCAGGTGAACAGCGGCAATACAATGCTGCTGCAGCGTGTGGAGCTCCCCAGCGACACGGAGCGGATAGAGCTGAGTGCGAGTGGGACAGGTGTGGCGGTGGTGCAGGTGACCTACCACTACAACCTGAAGGTGACTGGACCCAAGCCGGCCTTCTCCTTGGATCCTCAGCTGGACTTCTCTATGTCTGACACTAATCGCCTCAGACTCACTTCCTGCATTGGGTACACTGGCGGCAACAATAGCAACATGGCAGTGATGGACGTGTCTCTGCCCTCAGGTTACATCGTAGACAATGATCTTATCCCAGGGCTGTACGACTACGAGGGCGTCAAATTGGTGGAGAAGAAGCCAGACAGCTCCGGTGTGCTGGTGTATTTTGACCACCTGACGCCCGTTGAGGTGTGCCCCACTGTGGCAGCCTACAGGATCAACAAGGTGGCCTTCCAGAAGCCCTCTGCTGTCAGGGTGTACGACTACTATGACACCTCTCGACAGGCCCGCCAGTTCTACCGCCCACTACCAGCCAAGCTGTGCGACATCTGTGACCTGGACGAGTGTGACCCCCGCCAGTGTGAGGAGCAGATCATTGAGCTCAACAGACAGCTTCAGGACCCCGAGGACATAGAGCGGCCTGCCACAGTGACGGAGATCTCCTCAGGTTCTGCTCTTGTCCCTGCCCTGACCACCACCTGCTTGGCTCTTCTCGCTGCTTTCCTTCATCACTAG

MKWAAAVAVVVVAVGVMVGGAEAKGTYSVVASRVLRPNQVFHVAVSSHGTEGDVQVSAEVGGEQDSGNIILLRQIADLQPDSTQVLKFEIGDIGPGQYNLSVSGFGALTFRNTTKLDYVHKSYSAFIQTDKTIYKPGDLMRFRVVVVNPLLRPFVTGSIDVYVTDGAGHRVRQWKRVFTNKGVWSGELQLAKEPVLGDWNITVEVLGQKTSRAVQVAYYVLPKFEVIVTLPEYVTFDQGEMVATVEAKYTYGRAVKGEVTLQVTPTYKYGYLQAPYDDPIRVVKAIKGKTDVTIDLLSDARLKGDYARELEVTAYVKEELTERVQNATSHVTVYRYPYRLSLIRTSDSFKPGLTYTAFLKVSYQDDTPVTSGEVTVRHSFTRDPSGFTEEVHTIEPSGIVTLQFTPPLDESVVSLALEARYKDLTQWLGDIVRAQSPSNSFLQATLQTENPRVGGEILIGLNATQPLVYFVYQVLGRGDVVFSNTLQAHQGTTHTFRFLATRDMAPRARLLMYYVRDDGEVVADSLHFTVSGAIQNEVTVNLNPNVVDAAGEVDITVTTKPNAFVGVLAVDQRALMLGTHNHFSQHEVIDELETYDPGRKTLEAPWHALSRKKRALFNWMGTTTTSDVFKNAGIVVLTNGYVHDFNPFPTANQPPSDITGEIPFHRQTAVNGSFGREFAPIDASTLRPDLGPGLAYNAPTRPPLAGPYAFSYLPPPPDSRPRLYLNQHVPPTWLFLDAETKFNGVVRMREKAPDAITSYMISAFAIDDLYGLGVTERPTKLRVFRPFFVSMNLPAAGVVRGEAVAVEMVIFNYGDQQATAQVTLENPNEDFLFADFANEIDQGSPAAMKSREVVVASGTGMSVSFMVVPQTIGNIPITVRATTASAADVVTKQLLVKPEGTRQTVNRAMLIDLRSETSFSATVNITTPPNVVNESKAISVTLVGDVLGPAVSDLQSLVELPTGCGEQNMAKLVPNIVVTEYLKNKNQLDESLLGRAKRHLETGYQQQLNYRHPDGSFSAFGSRDKSGSTWLTAFVAKSLAEAGRHIEVEAEVVDKAVEWLRSQQVADGSFPEVGTVNNKAMQGGAAFGSALTAYVVMALLTTQNPPKAIVRNSINRGLDFLAKRLDDIEDLYSLALTTYVLHLADHTHKDAAFFKLESKAKIQENEKWWESGPLNRTVETMVDTRSVDVEMTGYGLMTYVRRGLVQDALPIMRWLVRRRNTHGGFMSTQDTVVALAGLAGLAERLTAANSRVNLRLIYGARGKNLQVNSGNTMLLQRVELPSDTERIELSASGTGVAVVQVTYHYNLKVTGPKPAFSLDPQLDFSMSDTNRLRLTSCIGYTGGNNSNMAVMDVSLPSGYIVDNDLIPGLYDYEGVKLVEKKPDSSGVLVYFDHLTPVEVCPTVAAYRINKVAFQKPSAVRVYDYYDTSRQARQFYRPLPAKLCDICDLDECDPRQCEEQIIELNRQLQDPEDIERPATVTEISSGSALVPALTTTCLALLAAFLHH
