# Supplementary material for: Full-Length Transcriptome Analysis Provides New Insights Into the Diversity of Immune-Related Genes in Portunus trituberculatus
Source: Front Immunol. 2022 Apr 7;13:843347. doi: 10.3389/fimmu.2022.843347 (PMC9021376; doi:10.3389/fimmu.2022.843347)
Supplement: Supplementary file 7 [file Table_1.doc]

| Sequence types | Polymerase read | Sub-read | CCS | FLNC | Polished consensus | Unigene |
| --- | --- | --- | --- | --- | --- | --- |
| Sequence number | 1,172,590 | 64,159,133 | 1,061,265 | 668,918 | 43,748 | 17,433 |
| Mean length | 75,948 | 1,314 | 2,006 | 1,760 | 1,917 | 2,271 |
| N50 length | 141,113 | 1,900 | 2,471 | 2,266 | 2,522 | 2,841 |
