# Supplementary material for: Full-Length Transcriptome Analysis Provides New Insights Into the Diversity of Immune-Related Genes in Portunus trituberculatus
Source: Front Immunol. 2022 Apr 7;13:843347. doi: 10.3389/fimmu.2022.843347 (PMC9021376; doi:10.3389/fimmu.2022.843347)
Supplement: Supplementary file 8 [file Table_2.doc]

| Databases | NR | NT | Pfam | SwissProt | GO | KOG | KEGG |
| --- | --- | --- | --- | --- | --- | --- | --- |
| Annotation number | 12,972 | 6,371 | 10,172 | 11,296 | 10,172 | 10,126 | 12,343 |
